# Evaluation of the yeast surface display system for screening of functional nanobodies

**DOI:** 10.1186/s13568-020-00983-y

**Published:** 2020-03-16

**Authors:** Kaho Kajiwara, Wataru Aoki, Mitsuyoshi Ueda

**Affiliations:** 1 Division of Applied Life Sciences, Graduate School of Agriculture, Kyoto University, Kitashirakawa Oiwake-cho, Sakyo-ku, Kyoto, 606-8502 Japan; 2 Core Research for Evolutional Science and Technology (CREST), Japan Science and Technology Agency (JST), 7 Goban-cho, Chiyoda-ku, Tokyo, 102-0076 Japan

**Keywords:** Cell surface display, Screening platform, Protein engineering, Library screening, Nanobody, *Saccharomyces cerevisiae*

## Abstract

Yeast surface display is a powerful technology used to isolate and engineer proteins to improve their activity, specificity, and stability. In this method, gene expression is regulated by promoters, and secretion efficiency is affected by secretion signals. Furthermore, both the accessibility and activity of the displayed proteins are affected by the length of anchor proteins. The ideal promoter, secretion signal, and anchor protein combination depend on the proteins of interest. In this study, we optimized a yeast surface display suitable for nanobody evaluation. We designed five display systems that used different combinations of promoters, secretion signals, and anchor proteins. Anti-hen egg-white lysozyme nanobody was used as the model nanobody. The amount of nanobodies displayed on yeast cells, the number of antigens bound to the displayed nanobodies, and the display efficiency were quantified. Overall, we improved the yeast display system for nanobody engineering and proposed its optimization.

## Introduction

Camelid single-domain antibody fragments (“nanobodies”) are being increasingly utilized in various applications (Salvador et al. 2019). Their most notable advantage is their small size; the molecular weight of one nanobody is approximately 15 kDa, i.e., one-tenth that of conventional IgG (Hamers-Casterman et al. 1993). Moreover, nanobodies exhibit high stability and solubility due to structural differences such as hallmark soluble amino acids in their framework regions and a disulfide bond between complementary-determining regions via extra cysteine residues (Vu et al. 1997; Govaert et al. 2012).

Several in vitro display methods have been developed to select nanobodies with high affinity and/or high thermostability (Yau et al. 2003; Ryckaert et al. 2010; Koide and Koide 2012; Fleetwood et al. 2013; Doshi et al. 2014; Moutel et al. 2016). Among them, yeast surface display is a potent screening platform used for protein engineering (Boder and Wittrup 1997; McMahon et al. 2018; Ueda 2019). This technology allows for the cell surface tethering of target proteins at the cell wall. In the yeast surface display system, a gene encoding a protein of interest with a secretion signal peptide is fused with a gene encoding an anchor protein. This fusion gene is then introduced into yeast cells and expressed under the control of any promoter. For instance, fusion proteins can be covalently linked to the cell wall via a glycosylphosphatidylinositol attachment signal (Ueda 2019). The most significant features of the yeast surface display system are its eukaryotic protein quality control and posttranslational modification machinery (Ueda 2019). As such, yeast can produce various eukaryotic proteins as its native biologically functional form. Furthermore, yeast cells are compatible with flow cytometry, allowing for quantitative screening (Ueda 2019).

The effects of promoters, secretion signals, and anchor proteins on display efficiencies of heterologous proteins have been studied. Selecting an appropriate promoter is important to control the expression levels of a gene of interest. In a previous study, β-glucosidase was displayed on a yeast cell surface under various promoters, and enzyme activities of β-glucosidase were compared, demonstrating that the enzymatic activities of the displayed proteins depended on the promoter strength (Inokuma et al. 2016).

The appropriate expression levels vary in every experiment, so it is necessary to determine an optimal promoter for each. To ensure that the fusion protein is properly directed to the cell surface, it is important to introduce a signal peptide to the fusion protein’s N-terminus. Two commonly used sequences for yeast surface display are the α pre-pro sequence derived from *Saccharomyces cerevisiae* and the glucoamylase secretion signal derived from *Rhizopus oryzae* (Kuroda et al. 2009; Ueda 2019). With regard to the α pre-pro signal sequence, directed evolution has been conducted to improve protein production levels (Rakestraw et al. 2009). Moreover, yeast display systems utilize various host cell wall proteins or synthetic tethers, and these anchor proteins have different lengths (Schreuder et al. 1996; Boder and Wittrup 1997; Van der Vaart et al. 1997; Ueda 2019). Both the accessibility and activity of a displayed protein have been shown to be affected by the length of the anchor protein used (Sato et al. 2002; McMahon et al. 2018). Therefore, it is important to select the appropriate anchor protein for a particular target protein because no universal anchor protein exists.

In this study, we evaluate various promoters, secretion signals, and anchor proteins to establish a yeast surface display suitable for nanobodies. We evaluate the effects of each parameter on the yeast surface display and propose an optimal screening platform for nanobody engineering.

## Materials and methods

### Construction of plasmids and yeast strains

DNA fragments of improved α-factor secretion signal (Rakestraw et al. 2009), anti-hen egg-white lysozyme nanobody cAbLys3 (Lauwereys et al. 1998), and 649-stalk (649 amino acids) (McMahon et al. 2018) were synthesized using gBlocks Gene Fragment (Integrated DNA Technologies, Coralville, IA, USA). The secretion signal of glucoamylase from *R. oryzae* and the C-terminal 320 amino acids of α-agglutinin were amplified from pULD1 (Kuroda et al. 2009) via PCR. These genes were cloned using an In-Fusion Cloning Kit (Takara Bio USA Inc., Shiga, Japan) and competent *Escherichia coli* DH5α (*F*^*−*^*Φ80lacZ*Δ*M*15 Δ(*lacZYA*-*argF*) *U169 endA1 recA1 hsdR17*(r_K_^−^, m_K_^+^) *deoR supE44 thi*-*1* λ^−^*gyrA96 relA1*). The transformed *E. coli* was cultured in Luria–Bertani media (1% [*w/v*] tryptone [Becton, Dickinson and Company, Franklin Lakes, NJ, USA], 0.5% [*w/v*] yeast extract [Becton, Dickinson and Company], and 1% [*w/v*] sodium chloride [Nacalai Tesque, Kyoto, Japan]) containing 100 µg/mL ampicillin (Meiji Seika, Tokyo, Japan). The full sequences of the plasmids used in this study are shown in Additional file 1: Figure S1.

The *Saccharomyces cerevisiae* strain BY4741 (*MATa*, *his3*Δ*1*, *leu2*Δ*0*, *met15*Δ*0*, *ura3*Δ*0*) was used as a host for the cell surface display of nanobodies. Yeast cells were transformed with the constructed plasmids via a Frozen EZ Yeast Transformation II Kit (Zymo Research, Irvine, CA, USA). The transformants were selected on synthetic dextrose solid medium (SDC glucose + HML) (0.67% [*w/v*] yeast nitrogen base without amino acids [Becton, Dickinson and Company], 2% [*w/v*] glucose [Nacalai Tesque], 0.5% [*w/v*] casamino acids [Becton, Dickinson and Company], 0.002% [*w/v*] l-histidine [Nacalai Tesque], 0.003% [*w/v*] l-leucine [Nacalai Tesque], 0.003% [*w/v*] l-methionine [Nacalai Tesque], adjusted to pH 6.0 with 1 N NaOH and 2% [*w/v*] agar [Nacalai Tesque]). The obtained colonies were precultured in liquid SDC glucose + HML media at 30 °C and 250 rpm for 24 h. Following the preculture, the optical density at a wavelength of 600 nm was measured, and the main cultures were started at an OD_600_ of 0.1.

### Immunofluorescence labeling of yeast cells for microscopic analysis

To confirm nanobody production on the cell surface display, immunofluorescence labeling of the cells was performed. The OD_600_ was measured in each sampling point, and approximately 4.5 × 10^6^ cells (OD_600_ of 1 ≈ 1.5 × 10^7^ yeast cells/mL) were subjected to immunofluorescence labeling. After centrifugation at 1000×*g* for 5 min, the cells were washed with phosphate-buffered saline (PBS, pH 7.2), resuspended in PBS containing 1% bovine serum albumin (Sigma-Aldrich, MO, USA), and incubated for 30 min at room temperature. Mouse monoclonal anti-HA tag antibody (Nacalai Tesque) or mouse monoclonal anti-FLAG M2 antibody (Sigma-Aldrich) was added at a dilution ratio of 1:500, and the solutions were incubated at room temperature with gentle shaking on a rotary shaker (WKN-2210, Wakenyaku, Kyoto, Japan) for 1 h. Following this, the cells were washed with PBS and incubated with Alexa Fluor^®^ 488 (AF488)-conjugated goat anti-mouse IgG secondary antibody (Invitrogen, CA, USA) diluted 1:1000 at room temperature with gentle shaking on a rotary shaker (WKN-2210, Wakenyaku) for 1.5 h. The cells were then used for further analysis after being washed with PBS.

After the immunofluorescence labeling, the cells were observed via an inverted microscope (IX71, Olympus, Tokyo, Japan). Green fluorescence from the AF488 was detected through a U-MNIBA2 mirror unit with a BP-470-490 excitation filter, DM505 dichroic mirror, and BA 510-550 emission filter (Olympus).

### Immunofluorescence labeling of yeast cells for flow cytometry

To quantify the amounts of displayed nanobodies and compare the five display systems, the fluorescence intensity was evaluated via flow cytometry. In addition to the immunofluorescent labeling described previously, Alexa Fluor^®^ 647 (AF647)-labeled lysozyme was incubated with the cells to quantify the relative amount of functional nanobodies. The fluorescence labeling of the lysozyme was performed using an Alexa Fluor^®^ 647 Microscale Protein Labeling Kit (Invitrogen Corporation, Carlsbad, CA, USA). In this labeling procedure, the AF647-labeled lysozyme was added at a dilution ratio of 1:500 with anti-mouse IgG secondary antibodies. After being washed with PBS, the cells were suspended in PBS and analyzed via a flow cytometer (JSAN, Bay Bioscience, Kobe, Japan). The fluorescence of AF488 was detected with an excitation at 488 nm and emission at 535 ± 23 nm, while that of AF647 was detected with an excitation at 640 nm and emission at 661 ± 10 nm. Then, the fluorescence intensity of 20,000 yeast cells was displayed as a density plot. The right upper region of the plot, which represented both AF488- and AF647-positive cells, was the Q2 region, and the ratio and mean fluorescence intensity of the yeast cells in the Q2 region were quantified. The experiments were performed in biological triplicate for each sample, and Tukey’s test was used for the statistical analysis.

## Results

### Plasmid design for the cell surface display of nanobodies

To optimize the cell surface display of nanobodies, five plasmids expected to be suitable based on previous studies were designed (Kuroda et al. 2009; Rakestraw et al. 2009; McMahon et al. 2018). In yeast surface display, gene expression levels are regulated by promoters, and secretion efficiency is influenced by secretion signals. Moreover, the accessibility and activity of the displayed proteins are affected by the length of anchor proteins. To analyze the effects of these three parameters, five display systems with different promoter, secretion signal, and anchor protein combinations were constructed (Table 1, Additional file 1). The constitutive *GAP* promoter and galactose-inducible *GAL1* promoter were used as the candidate promoters. For the secretion signals, the glucoamylase secretion signal and improved α-factor signal sequence (an engineered mating factor α leader sequence used to increase protein production) were selected (Rakestraw et al. 2009). With regard to the anchor proteins, 3′-half of α-agglutinin (320 amino acids) and 649-stalk (649 amino acids) were used. A HA-tag and anchor protein were fused to the C-terminus of the nanobody sequence because the complementary-determining regions of nanobodies exist at their N-terminus. The control plasmids had no nanobody sequence and a FLAG-tag instead of a HA-tag. Both the HA-tag and FLAG-tag were available for the detection of proteins displayed on the yeast cells (Kuroda et al. 2009; McMahon et al. 2018). The control strains were used to examine the nonspecific absorption during staining for the flow cytometry analysis.

**Table 1:** List of constructed display systems

Name	Promoter	Secretion signal	Anchor protein
System 1	GAP promoter	Glucoamylase secretion signal	3′-Half of α-agglutinin
System 2	GAP promoter	Improved α-factor secretion signal	3′-Half of α-agglutinin
System 3	GAP promoter	Improved α-factor secretion signal	649-stalk
System 4	GAL1 promoter	Improved α-factor secretion signal	3′-Half of α-agglutinin
System 5	GAL1 promoter	Improved α-factor secretion signal	649-stalk

### Cell surface display of a nanobody using the five display systems

**Fig. 1:**
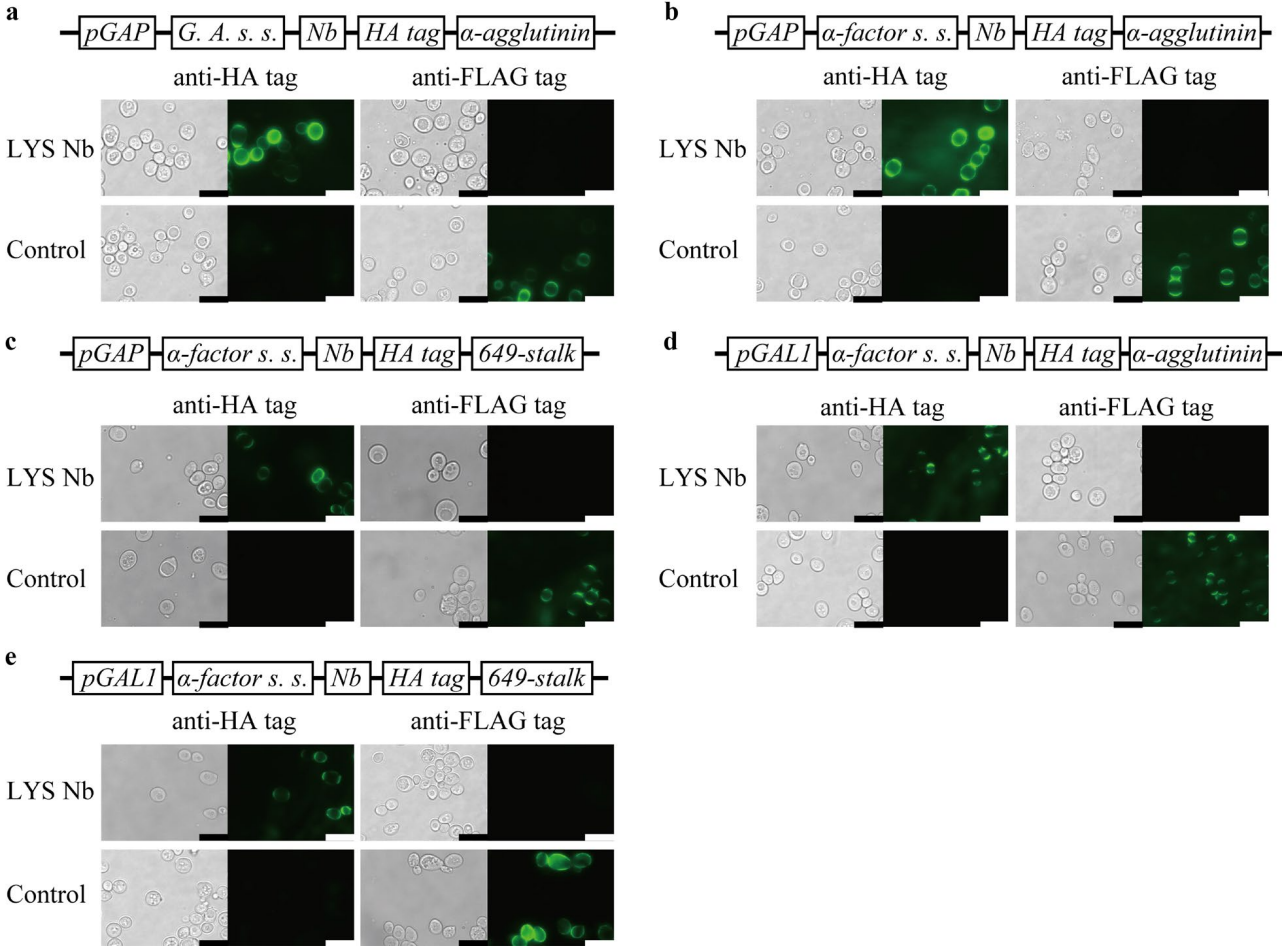
Immunofluorescence labeling of yeast cells. Anti-hen egg-white lysozyme nanobody (LYS Nb) was displayed using five different plasmids. The yeast cells were stained using either anti-HA tag mouse monoclonal antibody or anti-FLAG tag mouse monoclonal antibody as the primary antibody and Alexa Fluor^®^ 488 (AF488)-conjugated anti-mouse monoclonal antibody as the secondary antibody. The gene cassettes in Table I for each display system of LYS Nb are described above micrographs. LYS Nb: yeast cells with an LYS Nb-encoding plasmid. Control: yeast cells with a control plasmid that had no nanobody sequence and a FLAG-tag instead of an HA tag. Micrographs of **a** display system 1, **b** display system 2, **c** display system 3, **d** display system 4, and **e** display system 5. *pGAP*, glyceraldehyde-3-phosphate dehydrogenase promoter; *pGAL1*, galactokinase promoter; *G. A. s. s.*, secretion signal of glucoamylase from *Rhizopus oryzae*; *α*-*factor s. s.*, improved secretion signal of mating factor α preprotein from *Saccharomyces serevisiae*; *Nb*, anti-hen egg-white lysozyme nanobody; *α*-*agglutinin*, 320 C-terminal amino acids of α-agglutinin gene; *649*-*stalk*, a synthetic anchor protein consisting of 649 amino acids. Scale bars, 10 μm

To confirm nanobody production on the cell surface, the yeast cells were stained against the epitope tags and observed via fluorescence microscopy (Fig. 1). The yeast cells displaying the anti-hen egg-white lysozyme nanobody were successfully stained by the anti-HA tag antibody. Moreover, the control strains were successfully labeled with the anti-FLAG tag antibody, not the anti-HA tag antibody. These results indicate that all five designed plasmids (Table 1) can be used for nanobody display.

### Determination of the optimum culture conditions of the five display systems

**Fig. 2:**
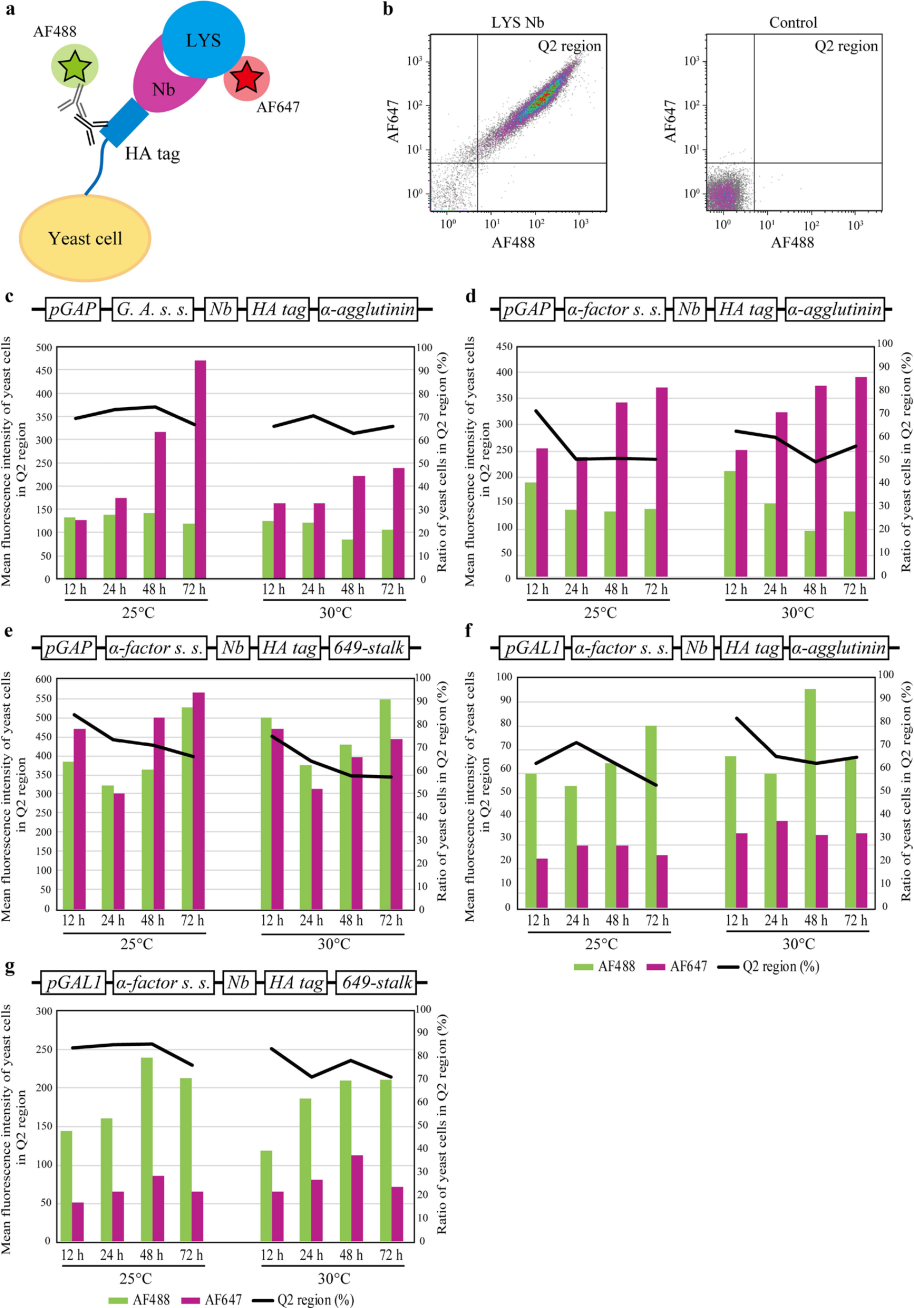
The effects of culture conditions on display efficiencies. **a** Schematic representation of the experimental scheme. Yeast cells were cultured at 25 °C or 30 °C for 72 h. Each yeast sample was stained using anti-HA tag mouse monoclonal antibody and AF488-conjugated anti-mouse monoclonal antibody as a secondary antibody to quantify the relative amount of displayed nanobodies. Alexa Fluor^®^ 647 (AF647)-labeled lysozyme was also used to quantify the relative amount of functional nanobodies. NB, anti-hen egg-white lysozyme nanobody; LYS, hen egg white-lysozyme. **b** Representative images of the flow cytometry analysis of the yeast cells with display system 1. The Q2 region (%) shows the ratio of yeast cells with strong AF488 and AF647 signals (see Materials and Methods). The display efficiencies of **c** display system 1, **d** display system 2, **e** display system 3, **f** display system 4, and **g** display system 5. The bar graphs display the mean fluorescence intensity of yeast cells in the Q2 region, and the black lines show the ratio of yeast cells in the Q2 region

To compare the display systems, we attempted to determine their optimum culture conditions. Both the anti-lysozyme nanobody strains and control strains were cultured at 25 °C or 30 °C for 24 h. At each sampling point, yeast cells were stained and subjected to flow cytometry analysis (Fig. 2a). The control strains showed little nonspecific absorption against the anti-HA tag antibody and AF647-labeled lysozyme (Fig. 2b). In the time-course analysis, almost all systems’ highest ratio of yeast cells in the Q2 region was within 12 h (Fig. 2c–g). Thus, the five display systems were compared after 12 h of culture.

### Evaluation of the effects of promoters, secretion signals, and anchor proteins

**Fig. 3:**
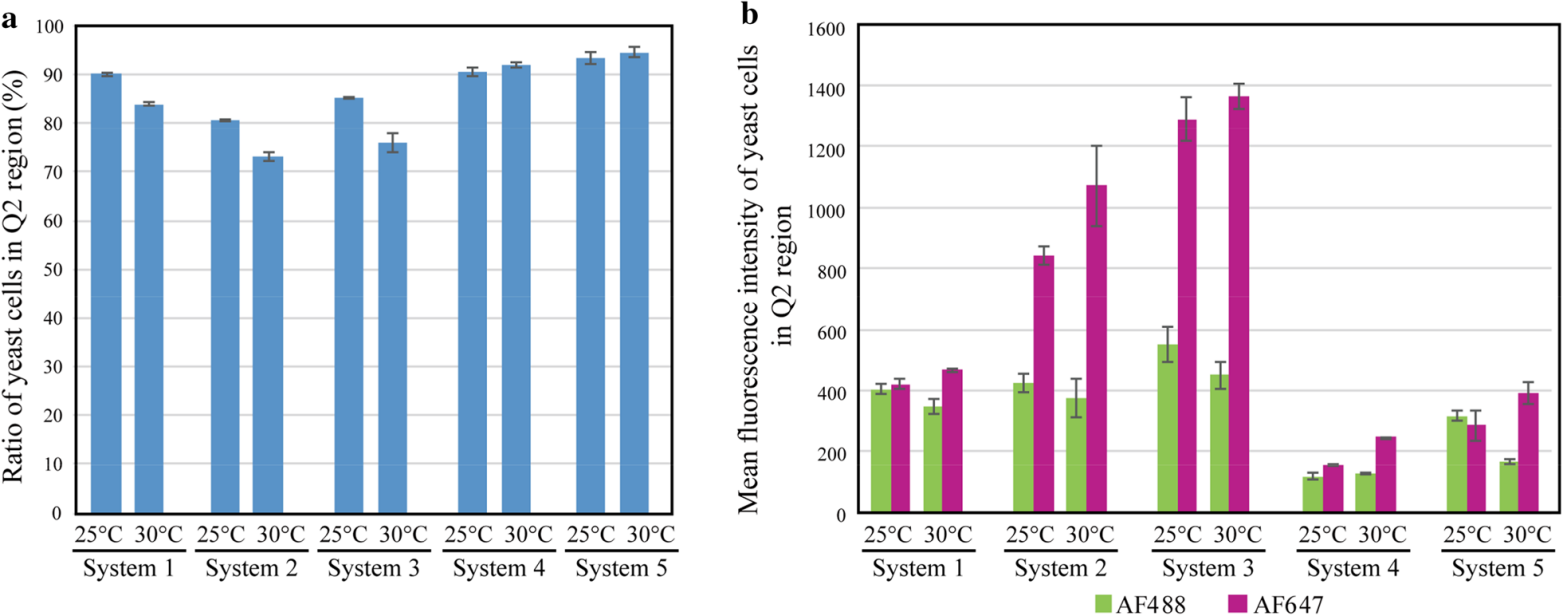
Comparison of the display efficiencies after 12-h culture. Each yeast sample was stained using anti-HA tag monoclonal antibody and AF488-conjugated anti-mouse secondary antibody and AF647-labeled lysozyme. **a** The ratio of yeast cells in the Q2 region, which represents yeast cells with strong AF488 and AF647 signals (see “Materials and methods”). **b** The mean fluorescence intensity of yeast cells in the Q2 region. The fluorescence intensity of AF488 represents the relative amount of displayed nanobodies on a yeast cell, while that of AF647 represents the relative amount of functional nanobodies. The bars indicate the means ± standard deviations of the three biological replicates

**Fig. 4:**
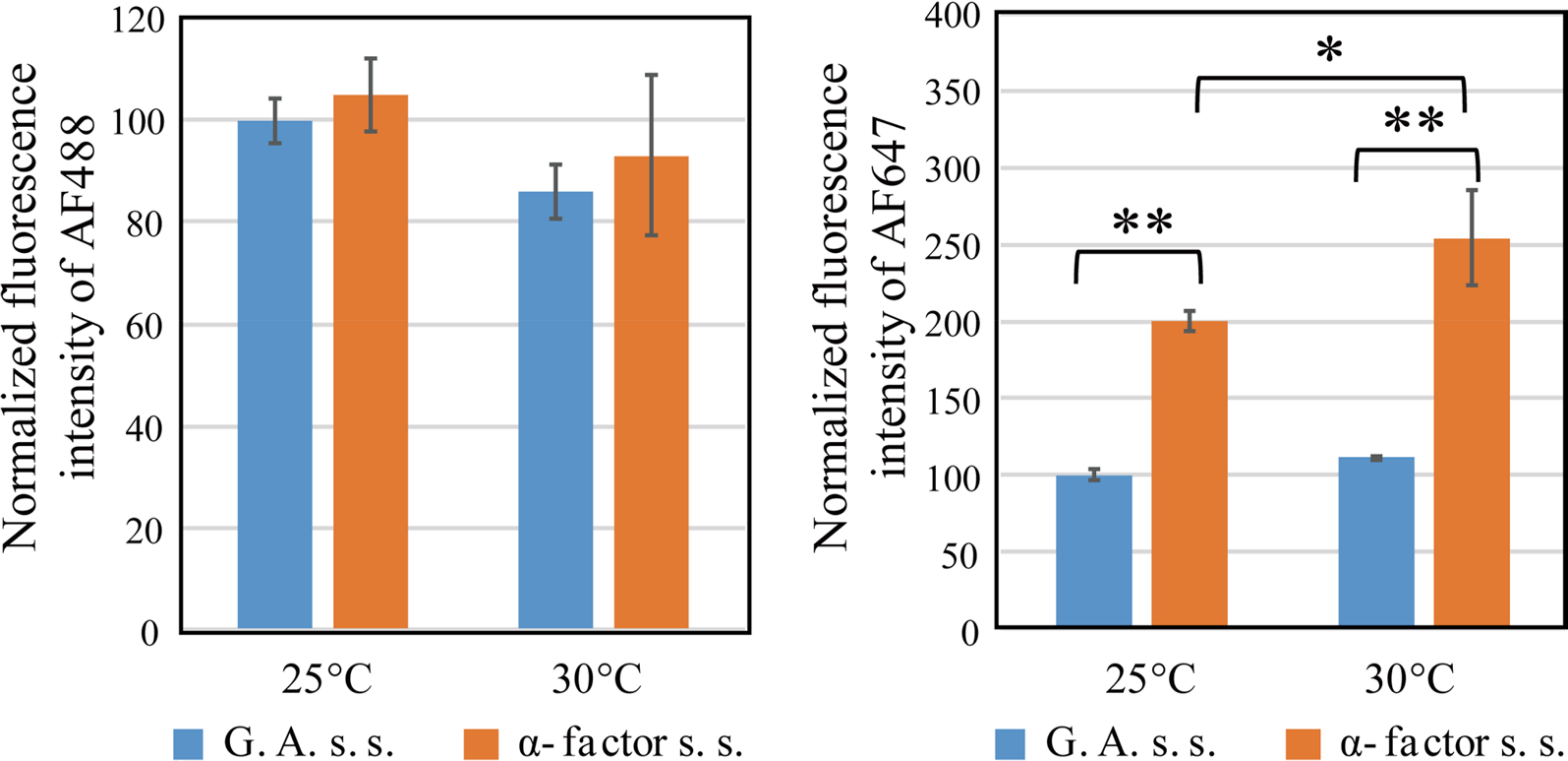
The effect of secretion signals on display efficiencies. To analyze the difference between the glucoamylase secretion signal (G. A. s. s.) and improved α-factor secretion signal (α-factor s. s.), the fluorescence intensities of display system 1 and display system 2 were compared. The data were extracted from Fig. 3. The fluorescence intensity of AF488 represents the relative amount of displayed nanobodies on a yeast cell, while that of AF647 represents the relative amount of functional nanobodies. The bars indicate the means ± standard deviations of the three biological replicates. The data were normalized against the fluorescence intensity of display system 1 at 25 °C. *P < 0.05, **P < 0.01, Tukey’s test

**Fig. 5:**
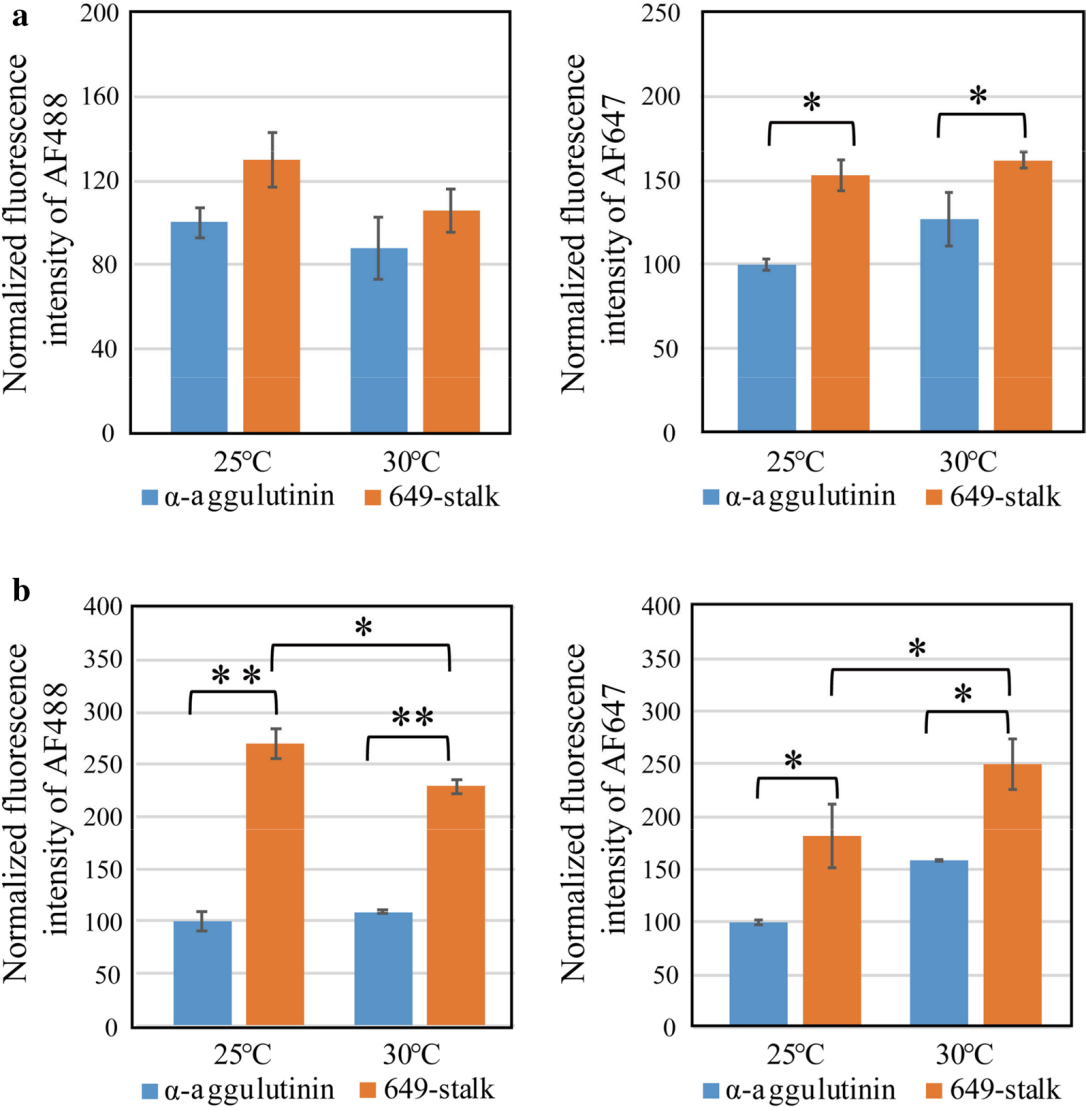
The effect of anchor proteins on display efficiencies. To analyze the difference between α-agglutinin and 649-stalk, **a** display systems 2 and 3 and **b** display systems 4 and 5 were compared. The data were extracted from Fig. 3. The fluorescence intensity of AF488 represents the relative amount of displayed nanobodies on a yeast cell, while that of AF647 represents the relative amount of functional nanobodies. **a** The influences of the anchor proteins under the *GAP* promoter in display systems 2 and 3. The data were normalized against the fluorescence intensity of display system 2 at 25 °C. **b** The influences of the anchor proteins under the *GAL1* promoter in display systems 4 and 5. The data were normalized against the fluorescence intensity of display system 4 at 25 °C. The bars indicate the means ± standard deviations of the three biological replicates. *P < 0.05, **P < 0.01, Tukey’s test

**Fig. 6:**
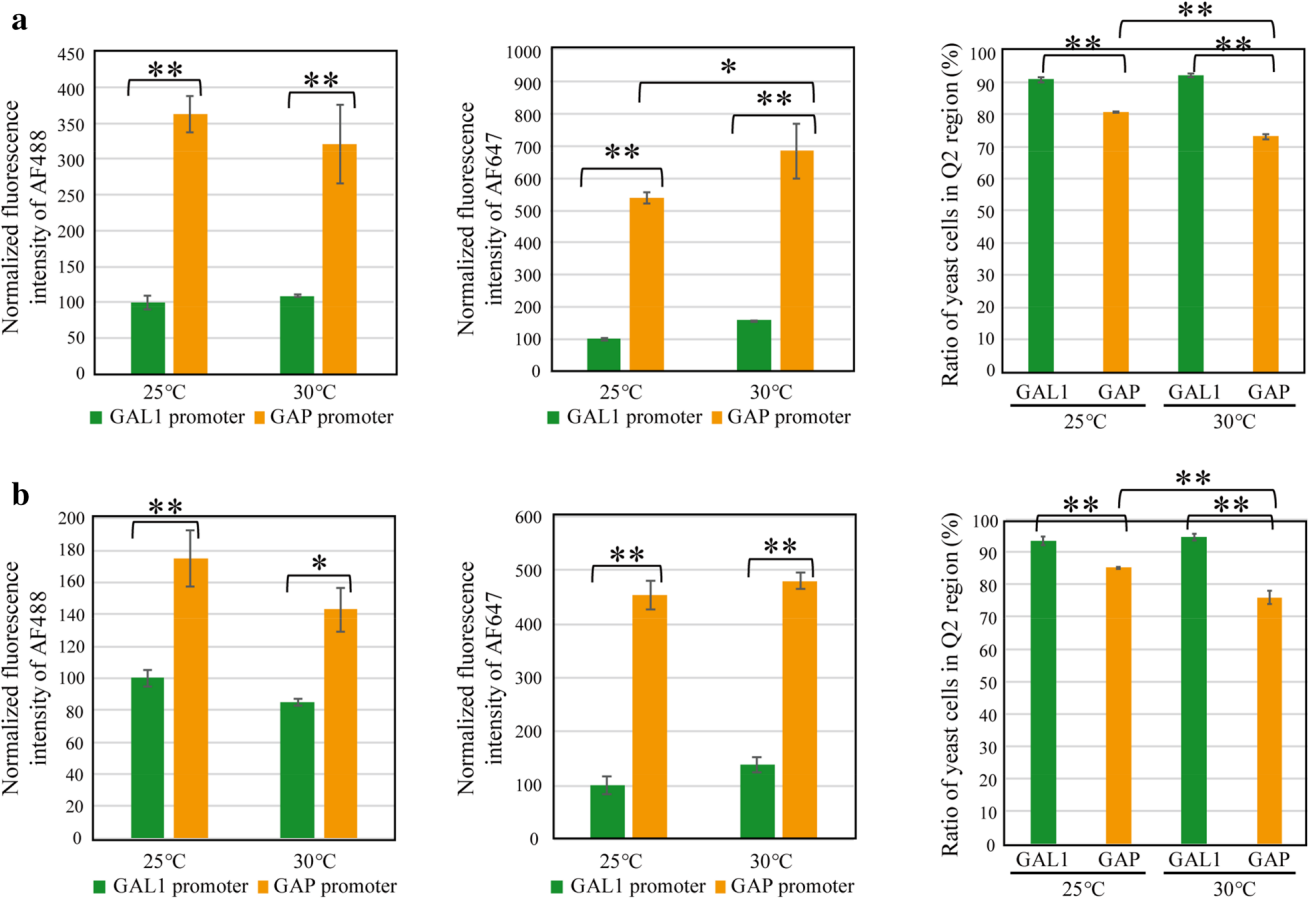
The effect of promoters on display efficiencies. The differences between the *GAP* promoter and *GAL1* promoter in **a** display systems 2 and 4 and **b** display systems 3 and 5 were compared. The data were extracted from Fig. 3. The fluorescence intensity of AF488 represents the relative amount of displayed nanobodies on a yeast cell, while that of AF647 represents the relative amount of functional nanobodies. The Q2 region represents yeast cells with strong AF488 and AF647 signals (see “Materials and methods”). **a** The influence of the promoters when the anchor protein was α-agglutinin. Display systems 2 and 4 were compared. The fluorescence intensity was normalized against that of display system 2 at 25 °C. **b** The influence of the promoters when the anchor protein was 649-stalk. Display systems 3 and 5 were compared. The fluorescence intensity was normalized against that of display system 3 at 25 °C. The bars indicate the means ± standard deviations of the three biological replicates. *P < 0.05, **P < 0.01, Tukey’s test

To evaluate the effects of promoters, secretion signals, and anchor proteins, the amount of nanobodies displayed on yeast cells and the ratio of yeast cells in the Q2 region were compared among the five display systems. The ratio of yeast cells in the Q2 region of the five display systems is shown in Fig. 3a, and the relative fluorescence intensities of AF488 and AF647 are shown in Fig. 3b. The fluorescence intensity of AF647 was higher at 30 °C than at 25 °C in all systems, indicating that the nanobodies were more active at the former temperature. These data are analyzed in further detail for each parameter in Figs. 4, 5 and 6.

The effects of secretion signals in systems 1 and 2 were compared (Fig. 4). The normalized fluorescence intensity of AF647 was higher in system 2 than in system 1, indicating that the improved α-factor secretion signal increased the amount of displayed functional nanobodies in comparison with the glucoamylase secretion signal.

The effects of anchor proteins in systems 2 and 3 and systems 4 and 5 were also compared (Fig. 5). With regard to 649-stalk, the amount of nanobodies that bound to the lysozyme increased about 1.5-fold compared with α-agglutinin. This is likely because the nanobodies were displayed more distant from the yeast surface, and the accessibility of the nanobodies to the antigens was improved.

Lastly, the effect of promoters in systems 2 and 4 and systems 3 and 5 was compared (Fig. 6). The fluorescence intensities of AF488 and AF647 were higher under the *GAP* promoter than under the *GAL1* promoter (Fig. 6a, b [left and middle panels]). These data indicate that the *GAP* promoter was stronger than the *GAL1* promoter; however, the ratio of yeast cells in the Q2 region was higher under the *GAL1* promoter than under the *GAP* promoter (Fig. 6a, b [right panels]).

## Discussion

In this study, we attempted to establish a yeast display system suitable for nanobodies. We designed five display systems to investigate the effects of different promoters, secretion signals, anchor proteins, and culture temperatures. We quantified the amount of nanobodies displayed on yeast cells, the number of antigens bound to displayed nanobodies, and the display efficiency and observed that the values of these primary endpoints largely varied depending on the parameters.

With regard to the effect of promoters, the *GAP* promoter was stronger than the *GAL1* promoter, and more nanobodies were displayed on the yeast cell surface under the former (Fig. 6). The tendency of differences in protein production between promoters was consistent with the results of previous studies (Piruzian et al. 2002; Partow et al. 2010). However, the display efficiency, i.e., the ratio of yeast cells in the Q2 region, was higher under the *GAL1* promoter than the *GAP* promoter (Fig. 6). This likely occurred because the production of heterologous proteins by the *GAL1* promoter may have been appreciably repressed before induction, avoiding possible negative effects on cell growth.

With regard to the secretion signals used in this study, the α pre-pro signal was most suitable for the yeast surface display of nanobodies. No significant difference in the amount of displayed nanobodies was observed between the two secretion signals tested, but there was a twofold difference in the amount of functional nanobodies (Fig. 4). The fluorescence intensity of AF488 represented the relative amount of displayed nanobodies on a yeast cell, while that of AF647 represented the relative amount of functional nanobodies. These data suggested that the nanobodies produced in display system 2 were more functional than those produced in display system 1. This was probably because the nanobodies were better folded in display system 2 due to the complicated secretory process of the α-mating factor. In glucoamylase processing, the signal peptidase cleavage occurs in one step after alanine at position 25 (Innis et al. 1985). In contrast, α-mating factor processing occurs in two steps: first, the pre-signal is removed by the signal peptidase in the endoplasmic reticulum, and second, KEX2 endopeptidase cleaves the pro-leader sequence between the arginine and lysine (Brake et al. 1984). The longer transition time in the ER may provide additional time for proper protein folding (Kjeldsen et al. 1997).

Concerning the anchor proteins, we confirmed that their length affected the accessibility of antigens to the nanobodies. As a synthetic tether mimicking the low-complexity sequence of yeast cell wall proteins, 649-stalk has been shown to enhance this accessibility compared with shorter synthetic tethers (McMahon et al. 2018). In this study, we compared this synthetic anchor protein with a widely used anchor protein derived from α-agglutinin, a cell adhesion glycoprotein (Lipke and Kurjan 1992). Overall, 649-stalk showed a higher fluorescence intensity of AF488 and AF647 than α-agglutinin (Fig. 5). With the longer anchor protein, there was less congestion around the nanobodies and HA-tag because the nanobodies were displayed more distant from the cell surface. Therefore, 649-stalk improved the accessibility of the anti-HA tag antibody to the HA-tag and the accessibility of the nanobodies to their antigen, lysozyme. This effect of the long anchor protein was consistent with previously reported data (Sato et al. 2002).

In addition to the effects of these three parameters, this study revealed that the nanobodies were more functional at 30 °C than at 25 °C. The fluorescence intensity of AF488 was higher at 25 °C than at 30 °C in almost all systems, whereas that of AF647 was highest at 30 °C. In general, a low temperature effectively improves protein expression levels and aids protein folding because it reduces the cell growth rate and allows for protein folding without rate limiting (Hong et al. 2002; Camarero et al. 2012). Moreover, here, there were more functional displayed nanobodies at 30 °C than at 25 °C. It has been stated that thermal stability improves protein folding (Vogt et al. 1997). The melting temperature (*T*_*m*_) is an index of thermal stability, and a protein with a high *T*_*m*_ is considered to be more stable under physiological conditions. The *T*_*m*_ value of the nanobody cAbLys3 used in this study was relatively high (Govaert et al. 2012); thus, we assumed that protein folding is not a rate limiting process, even at 30 °C. In addition, the nanobodies may have been more functional at 30 °C than at 25 °C because 30 °C is closer to the body temperature of camelids (Zanolari et al. 2010).

In summary, this study examined various key parameters regarding the yeast surface display of nanobodies. When considering conditions to evaluate nanobody library preparation, there are two important points: first, the ratio of yeast producing properly folded nanobodies should be high, and second, the binding ability of each mutant should be finely evaluated. Here, the display efficiency was shown to be high under the *GAL1* promoter, and the amount of displayed nanobodies under the *GAL1* promoter was less than that under the *GAP* promoter (Fig. 6). Thus, it was expected that the avidity effect would be reduced under the *GAL1* promoter. As a result, we could evaluate the affinity, i.e., the strength of a single interaction of a displayed nanobody and its antigens. Furthermore, we confirmed that the antigen-binding ability of the nanobodies was improved using the α pre-pro signal (Fig. 4), and the accessibility was improved by a long anchor protein (Fig. 5). Overall, the knowledge gained in this study will allow us to evaluate the preparation of nanobodies in the future.

## Supplementary information

**Additional file 1: Figure S1.** Plasmid sequences used in this study.

## Data Availability

All relevant data are within the manuscript and its additional information files.

## Abbreviations

AF488: Alexa Fluor^®^ 488
AF647: Alexa Fluor^®^ 647
