# Supplementary material for: Evaluation of the yeast surface display system for screening of functional nanobodies
Source: AMB Express. 2020 Mar 16;10:51. doi: 10.1186/s13568-020-00983-y (PMC7076106; doi:10.1186/s13568-020-00983-y)
Supplement: Supplementary file 1 — Additional file 1: Figure S1. Plasmid sequences used in this study. [file 13568_2020_983_MOESM1_ESM.pdf]

System 1 anti-lys Nb strain (9399 bp)

GTTAACGAAGCATCTGTGCTTCATTTTGTAGAACAAAAATGCAACGCGAGAGCGCTAATTTTTCAAACAAAGAATCTGAGCTGCATTTTTACAGAACAGAAATGCAA
CAATTGCTTCGTAGACACGAAGTAAAACATCTTGTTTTTACGTTGCGCTCTCGCGATTAAAAAGTTTGTTTCTTAGACTCGACGTAAAAATGTCTTGTCTTTACGTT
2 micron origin
20 40 60 80 100

CGCGAAAGCGCTATTTTACCAACGAAGAATCTGTGCTTCATTTTTGTAAAACAAAAATGCAACGCGAGAGCGCTAATTTTTCAAACAAAGAATCTGAGCTGCATTTT
GCGCTTTCGCGATAAAATGGTTGCTTCTTAGACACGAAGTAAAAACATTTTGTTTTTACGTTGCGCTCTCGCGATTAAAAAGTTTGTTTCTTAGACTCGACGTAAAA
2 micron origin
120 140 160 180 200

TACAGAACAGAAATGCAACGCGAGAGCGCTATTTTACCAACAAAGAATCTATACTTCTTTTTTGTTCTACAAAAATGCATCCCGAGAGCGCTATTTTTCTAACAAAG
ATGTCTTGTCTTTACGTTGCGCTCTCGCGATAAAATGGTTGTTTCTTAGATATGAAGAAAAAACAAGATGTTTTTACGTAGGGCTCTCGCGATAAAAAGATTGTTTC
2 micron origin
220 240 260 280 300 320

CATCTTAGATTACTTTTTTTCTCCTTTGTGCGCTCTATAATGCAGTCTCTTGATAACTTTTTGCACTGTAGGTCCGTTAAGGTTAGAAGAAGGCTACTTTGGTGTCT
GTAGAATCTAATGAAAAAAAGAGGAAACACGCGAGATATTACGTCAGAGAACTATTGAAAAACGTGACATCCAGGCAATTCCAATCTTCTTCCGATGAAACCACAGA
2 micron origin
340 360 380 400 420

ATTTTCTCTTCCATAAAAAAAGCCTGACTCCACTTCCCGCGTTTACTGATTACTAGCGAAGCTGCGGGTGCATTTTTTCAAGATAAAGGCATCCCCGATTATATTCT
TAAAAGAGAAGGTATTTTTTTCGGACTGAGGTGAAGGGCGCAAATGACTAATGATCGCTTCGACGCCCACGTAAAAAAGTTCTATTTCCGTAGGGGCTAATATAAGA
2 micron origin
440 460 480 500 520

ATACCGATGTGGATTGCGCATACTTTGTGAACAGAAAGTGATAGCGTTGATGATTCTTCATTGGTCAGAAAATTATGAACGGTTTCTTCTATTTTGTCTCTATATAC
TATGGCTACACCTAACGCGTATGAAACACTTGTCTTTCACTATCGCAACTACTAAGAAGTAACCAGTCTTTTAATACTTGCCAAAGAAGATAAAACAGAGATATATG
2 micron origin
540 560 580 600 620 640

TACGTATAGGAAATGTTTACATTTTCGTATTGTTTTCGATTCACTCTATGAATAGTTCTTACTACAATTTTTTTGTCTAAAGAGTAATACTAGAGATAAACATAAAA
ATGCATATCCTTTACAAATGTAAAAGCATAACAAAAGCTAAGTGAGATACTTATCAAGAATGATGTTAAAAAAACAGATTTCTCATTATGATCTCTATTTGTATTTT
2 micron origin
660 680 700 720 740

AATGTAGAGGTCGAGTTTAGATGCAAGTTCAAGGAGCGAAAGGTGGATGGGTAGGTTATATAGGGATATAGCACAGAGATATATAGCAAAGAGATACTTTTGAGCAA
TTACATCTCCAGCTCAAATCTACGTTCAAGTTCCTCGCTTTCCACCTACCCATCCAATATATCCCTATATCGTGTCTCTATATATCGTTTCTCTATGAAAACTCGTT
2 micron origin
760 780 800 820 840

TGTTTGTGGAAGCGGTATTCGCAATATTTTAGTAGCTCGTTACAGTCCGGTGCGTTTTTGGTTTTTTGAAAGTGCGTCTTCAGAGCGCTTTTGGTTTTCAAAAGCGC
ACAAACACCTTCGCCATAAGCGTTATAAAATCATCGAGCAATGTCAGGCCACGCAAAAACCAAAAAACTTTCACGCAGAAGTCTCGCGAAAACCAAAAGTTTTCGCG
2 micron origin
860 880 900 920 940 960

TCTGAAGTTCCTATACTTTCTAGCTAGAGAATAGGAACTTCGGAATAGGAACTTCAAAGCGTTTCCGAAAACGAGCGCTTCCGAAAATGCAACGCGAGCTGCGCACA
AGACTTCAAGGATATGAAAGATCGATCTCTTATCCTTGAAGCCTTATCCTTGAAGTTTCGCAAAGGCTTTTGCTCGCGAAGGCTTTTACGTTGCGCTCGACGCGTGT
2 micron origin
980 1,000 1,020 1,040 1,060

TACAGCTCACTGTTCACGTCGCACCTATATCTGCGTGTTGCCTGTATATATATATACATGAGAAGAACGGCATAGTGCGTGTTTATGCTTAAATGCGTACTTATATG
ATGTCGAGTGACAAGTGCAGCGTGGATATAGACGCACAACGGACATATATATATATGTACTCTTCTTGCCGTATCACGCACAAATACGAATTTACGCATGAATATAC
2 micron origin
1,080 1,100 1,120 1,140 1,160

CGTCTATTTATGTAGGATGAAAGGTAGTCTAGTACCTCCTGTGATATTATCCCATTCCATGCGGGGTATCGTATGCTTCCTTCAGCACTACCCTTTAGCTGTTCTAT
GCAGATAAATACATCCTACTTTCCATCAGATCATGGAGGACACTATAATAGGGTAAGGTACGCCCCATAGCATACGAAGGAAGTCGTGATGGGAAATCGACAAGATA
2 micron origin
1,180 1,200 1,220 1,240 1,260 1,280

ATGCTGCCACTCCTCAATTGGATTAGTCTCATCCTTCAATGCTATCATTTCCTTTGATATTGGATCGATCCGATGATAAGCTGTCAAACATGAGAATTGGGTAATAA
TACGACGGTGAGGAGTTAACCTAATCAGAGTAGGAAGTTACGATAGTAAAGGAAACTATAACCTAGCTAGGCTACTATTCGACAGTTTGTACTCTTAACCCATTATT
2 micron origin URA3
1,300 1,320 1,340 1,360 1,380

CTGATATAATTAAATTGAAGCTCTAATTTGTGAGTTTAGTATACATGCATTTACTTATAATACAGTTTTTTAGTTTTGCTGGCCGCATCTTCTCAAATATGCTTCCC
GACTATATTAATTTAACTTCGAGATTAAACACTCAAATCATATGTACGTAAATGAATATTATGTCAAAAAATCAAAACGACCGGCGTAGAAGAGTTTATACGAAGGG
URA3
1,400 1,420 1,440 1,460 1,480

AGCCTGCTTTTCTGTAACGTTCACCCTCTACCTTAGCATCCCTTCCCTTTGCAAATAGTCCTCTTCCAACAATAATAATGTCAGATCCTGTAGAGACCACATCATCC
TCGGACGAAAAGACATTGCAAGTGGGAGATGGAATCGTAGGGAAGGGAAACGTTTATCAGGAGAAGGTTGTTATTATTACAGTCTAGGACATCTCTGGTGTAGTAGG
URA3
1,500 1,520 1,540 1,560 1,580 1,600

ACGGTTCTATACTGTTGACCCAATGCGTCTCCCTTGTCATCTAAACCCACACCGGGTGTCATAATCAACCAATCGTAACCTTCATCTCTTCCACCCATGTCTCTTTG
TGCCAAGATATGACAACTGGGTTACGCAGAGGGAACAGTAGATTTGGGTGTGGCCCACAGTATTAGTTGGTTAGCATTGGAAGTAGAGAAGGTGGGTACAGAGAAAC
URA3
1,620 1,640 1,660 1,680 1,700

AGCAATAAAGCCGATAACAAAATCTTTGTCGCTCTTCGCAATGTCAACAGTACCCTTAGTATATTCTCCAGTAGATAGGGAGCCCTTGCATGACAATTCTGCTAACA
TCGTTATTTCGGCTATTGTTTTAGAAACAGCGAGAAGCGTTACAGTTGTCATGGGAATCATATAAGAGGTCATCTATCCCTCGGGAACGTACTGTTAAGACGATTGT

URA3

1,720 1,740 1,760 1,780 1,800

TCAAAAGGCCTCTAGGTTCCTTTGTTACTTCTTCTGCCGCCTGCTTCAAACCGCTAACAATACCTGGGCCCACCACACCGTGTGCATTCGTAATGTCTGCCCATTCT
AGTTTTCCGGAGATCCAAGGAAACAATGAAGAAGACGGCGGACGAAGTTTGGCGATTGTTATGGACCCGGGTGGTGTGGCACACGTAAGCATTACAGACGGGTAAGA

URA3

1,820 1,840 1,860 1,880 1,900 1,920

GCTATTCTGTATACACCCGCAGAGTACTGCAATTTGACTGTATTACCAATGTCAGCAAATTTTCTGTCTTCGAAGAGTAAAAAATTGTACTTGGCGGATAATGCCTT
CGATAAGACATATGTGGGCGTCTCATGACGTTAAACTGACATAATGGTTACAGTCGTTTAAAAGACAGAAGCTTCTCATTTTTTAACATGAACCGCCTATTACGGAA

URA3

1,940 1,960 1,980 2,000 2,020

TAGCGGCTTAACTGTGCCCTCCATGGAAAAATCAGTCAAGATATCCACATGTGTTTTTAGTAAACAAATTTTGGGACCTAATGCTTCAACTAACTCCAGTAATTCCT
ATCGCCGAATTGACACGGGAGGTACCTTTTTAGTCAGTTCTATAGGTGTACACAAAAATCATTTGTTTAAAACCCTGGATTACGAAGTTGATTGAGGTCATTAAGGA

URA3

2,040 2,060 2,080 2,100 2,120 2,140

TGGTGGTACGAACATCCAATGAAGCACACAAGTTTGTTTGCTTTTCGTGCATGATATTAAATAGCTTGGCAGCAACAGGACTAGGATGAGTAGCAGCACGTTCCTTA
ACCACCATGCTTGTAGGTTACTTCGTGTGTTCAAACAAACGAAAAGCACGTACTATAATTTATCGAACCGTCGTTGTCCTGATCCTACTCATCGTCGTGCAAGGAAT

URA3

2,160 2,180 2,200 2,220 2,240

TATGTAGCTTTCGACATGATTTATCTTCGTTTCCTGCATGTTTTTGTTCTGTGCAGTTGGGTTAAGAATACTGGGCAATTTCATGTTTCTTCAACACTACATATGCG
ATACATCGAAAGCTGTACTAAATAGAAGCAAAGGACGTACAAAAACAAGACACGTCAACCCAATTCTTATGACCCGTTAAAGTACAAAGAAGTTGTGATGTATACGC

URA3

2,260 2,280 2,300 2,320 2,340

TATATATACCAATCTAAGTCTGTGCTCCTTCCTTCGTTCTTCCTTCTGTTCGGAGATTACCGAATCAAAAAAATTTCAAAGAAACCGAAATCAAAAAAAAGAATAAA
ATATATATGGTTAGATTCAGACACGAGGAAGGAAGCAAGAAGGAAGACAAGCCTCTAATGGCTTAGTTTTTTTAAAGTTTCTTTGGCTTTAGTTTTTTTTCTTATTT

URA3

2,360 2,380 2,400 2,420 2,440 2,460

AAAAAAATGATGAATTGAATTGAAAAGCTAATTCTTGAAGACGAAAGGGCCTCGTGATACGCCTATTTTTATAGGTTAATGTCATGATAATAATGGTTTCTTAGACG
TTTTTTTACTACTTAACTTAACTTTTCGATTAAGAACTTCTGCTTTCCCGGAGCACTATGCGGATAAAAATATCCAATTACAGTACTATTATTACCAAAGAATCTGC

URA3

2,480 2,500 2,520 2,540 2,560

TCAGGTGGCACTTTTCGGGGAAATGTGCGCGGAACCCCTATTTGTTTATTTTTCTAAATACATTCAAATATGTATCCGCTCATGAGACAATAACCCTGATAAATGCT
AGTCCACCGTGAAAAGCCCCTTTACACGCGCCTTGGGGATAAACAAATAAAAAGATTTATGTAAGTTTATACATAGGCGAGTACTCTGTTATTGGGACTATTTACGA

2,580 2,600 2,620 2,640 2,660

TCAATAATATTGAAAAAGGAAGAGTATGAGTATTCAACATTTCCGTGTCGCCCTTATTCCCTTTTTTGCGGCATTTTGCCTTCCTGTTTTTGCTCACCCAGAAACGC
AGTTATTATAACTTTTTCCTTCTCATACTCATAAGTTGTAAAGGCACAGCGGGAATAAGGGAAAAAACGCCGTAAAACGGAAGGACAAAAACGAGTGGGTCTTTGCG

2,680 2,700 2,720 2,740 2,760 2,780

TGGTGAAAGTAAAAGATGCTGAAGATCAGTTGGGTGCACGAGTGGGTTACATCGAACTGGATCTCAACAGCGGTAAGATCCTTGAGAGTTTTCGCCCCGAAGAACGT
ACCACTTTCATTTTCTACGACTTCTAGTCAACCCACGTGCTCACCCAATGTAGCTTGACCTAGAGTTGTCGCCATTCTAGGAACTCTCAAAAGCGGGGCTTCTTGCA

2,800 2,820 2,840 2,860 2,880

TTTCCAATGATGAGCACTTTTAAAGTTCTGCTATGTGGCGCGGTATTATCCCGTATTGACGCCGGGCAAGAGCAACTCGGTCGCCGCATACACTATTCTCAGAATGA
AAAGGTTACTACTCGTGAAAATTTCAAGACGATACACCGCGCCATAATAGGGCATAACTGCGGCCCGTTCTCGTTGAGCCAGCGGCGTATGTGATAAGAGTCTTACT

AmpR

2,900 2,920 2,940 2,960 2,980

CTTGGTTGAGTACTCACCAGTCACAGAAAAGCATCTTACGGATGGCATGACAGTAAGAGAATTATGCAGTGCTGCCATAACCATGAGTGATAACACTGCGGCCAACT
GAACCAACTCATGAGTGGTCAGTGTCTTTTCGTAGAATGCCTACCGTACTGTCATTCTCTTAATACGTCACGACGGTATTGGTACTCACTATTGTGACGCCGGTTGA

AmpR

3,000 3,020 3,040 3,060 3,080 3,100

TACTTCTGACAACGATCGGAGGACCGAAGGAGCTAACCGCTTTTTTGCACAACATGGGGGATCATGTAACTCGCCTTGATCGTTGGGAACCGGAGCTGAATGAAGCC
ATGAAGACTGTTGCTAGCCTCCTGGCTTCCTCGATTGGCGAAAAAACGTGTTGTACCCCCTAGTACATTGAGCGGAACTAGCAACCCTTGGCCTCGACTTACTTCGG

AmpR

3,120 3,140 3,160 3,180 3,200

ATACCAAACGACGAGCGTGACACCACGATGCCTGTAGCAATGGCAACAACGTTGCGCAAACTATTAACTGGCGAACTACTTACTCTAGCTTCCCGGCAACAATTAAT
TATGGTTTGCTGCTCGCACTGTGGTGCTACGGACATCGTTACCGTTGTTGCAACGCGTTTGATAATTGACCGCTTGATGAATGAGATCGAAGGGCCGTTGTTAATTA

AmpR

3,220 3,240 3,260 3,280 3,300

AGACTGGATGGAGGCGGATAAAGTTGCAGGACCACTTCTGCGCTCGGCCCTTCCGGCTGGCTGGTTTATTGCTGATAAATCTGGAGCCGGTGAGCGTGGGTCTCGCG
TCTGACCTACCTCCGCCTATTTCAACGTCCTGGTGAAGACGCGAGCCGGGAAGGCCGACCGACCAAATAACGACTATTTAGACCTCGGCCACTCGCACCCAGAGCGC

AmpR

3,320 3,340 3,360 3,380 3,400 3,420

GTATCATTGCAGCACTGGGGCCAGATGGTAAGCCCTCCCGTATCGTAGTTATCTACACGACGGGGAGTCAGGCAACTATGGATGAACGAAATAGACAGATCGCTGAG
CATAGTAACGTCGTGACCCCGGTCTACCATTCGGGAGGGCATAGCATCAATAGATGTGCTGCCCCTCAGTCCGTTGATACCTACTTGCTTTATCTGTCTAGCGACTC

AmpR

3,440 3,460 3,480 3,500 3,520

ATAGGTGCCTCACTGATTAAGCATTGGTAACTGTCAGACCAAGTTTACTCATATATACTTTAGATTGATTTAAAACTTCATTTTTAATTTAAAAGGATCTAGGTGAA
TATCCACGGAGTGACTAATTCGTAACCATTGACAGTCTGGTTCAAATGAGTATATATGAAATCTAACTAAATTTTGAAGTAAAAATTAAATTTTCCTAGATCCACTT

AmpR

3,540 3,560 3,580 3,600 3,620

GATCCTTTTTGATAATCTCATGACCAAAATCCCTTAACGTGAGTTTTCGTTCCACTGAGCGTCAGACCCCGTAGAAAAGATCAAAGGATCTTCTTGAGATCCTTTTT
CTAGGAAAAACTATTAGAGTACTGGTTTTAGGGAATTGCACTCAAAAGCAAGGTGACTCGCAGTCTGGGGCATCTTTTCTAGTTTCCTAGAAGAACTCTAGGAAAAA
ColE1 origin
3,640 3,660 3,680 3,700 3,720 3,740

TTCTGCGCGTAATCTGCTGCTTGCAAACAAAAAAACCACCGCTACCAGCGGTGGTTTGTTTGCCGGATCAAGAGCTACCAACTCTTTTTCCGAAGGTAACTGGCTTC
AAGACGCGCATTAGACGACGAACGTTTGTTTTTTTGGTGGCGATGGTCGCCACCAAACAAACGGCCTAGTTCTCGATGGTTGAGAAAAAGGCTTCCATTGACCGAAG
ColE1 origin
3,760 3,780 3,800 3,820 3,840

AGCAGAGCGCAGATACCAAATACTGTCCTTCTAGTGTAGCCGTAGTTAGGCCACCACTTCAAGAACTCTGTAGCACCGCCTACATACCTCGCTCTGCTAATCCTGTT
TCGTCTCGCGTCTATGGTTTATGACAGGAAGATCACATCGGCATCAATCCGGTGGTGAAGTTCTTGAGACATCGTGGCGGATGTATGGAGCGAGACGATTAGGACAA
ColE1 origin
3,860 3,880 3,900 3,920 3,940

ACCAGTGGCTGCTGCCAGTGGCGATAAGTCGTGTCTTACCGGGTTGGACTCAAGACGATAGTTACCGGATAAGGCGCAGCGGTCGGGCTGAACGGGGGGTTCGTGCA
TGGTCACCGACGACGGTCACCGCTATTCAGCACAGAATGGCCCAACCTGAGTTCTGCTATCAATGGCCTATTCCGCGTCGCCAGCCCGACTTGCCCCCCAAGCACGT
ColE1 origin
3,960 3,980 4,000 4,020 4,040 4,060

CACAGCCCAGCTTGGAGCGAACGACCTACACCGAACTGAGATACCTACAGCGTGAGCTATGAGAAAGCGCCACGCTTCCCGAAGGGAGAAAGGCGGACAGGTATCCG
GTGTCGGGTCGAACCTCGCTTGCTGGATGTGGCTTGACTCTATGGATGTCGCACTCGATACTCTTTCGCGGTGCGAAGGGCTTCCCTCTTTCCGCCTGTCCATAGGC
ColE1 origin
4,080 4,100 4,120 4,140 4,160

GTAAGCGGCAGGGTCGGAACAGGAGAGCGCACGAGGGAGCTTCCAGGGGGAAACGCCTGGTATCTTTATAGTCCTGTCGGGTTTCGCCACCTCTGACTTGAGCGTCG
CATTCGCCGTCCCAGCCTTGTCCTCTCGCGTGCTCCCTCGAAGGTCCCCCTTTGCGGACCATAGAAATATCAGGACAGCCCAAAGCGGTGGAGACTGAACTCGCAGC
ColE1 origin
4,180 4,200 4,220 4,240 4,260 4,280

ATTTTTGTGATGCTCGTCAGGGGGGCGGAGCCTATGGAAAAACGCCAGCAACGCGGCCTTTTTACGGTTCCTGGCCTTTTGCTGGCCTTTTGCTCACATGTTCTTTC
TAAAAACACTACGAGCAGTCCCCCCGCCTCGGATACCTTTTTGCGGTCGTTGCGCCGGAAAAATGCCAAGGACCGGAAAACGACCGGAAAACGAGTGTACAAGAAAG
ColE1 origin
4,300 4,320 4,340 4,360 4,380

CTGCGTTATCCCCTGATTCTGTGGATAACCGTATTACCGCCTTTGAGTGAGCTGATACCGCTCGCCGCAGCCGAACGACCGAGCGCAGCGAGTCAGTGAGCGAGGAA
GACGCAATAGGGGACTAAGACACCTATTGGCATAATGGCGGAAACTCACTCGACTATGGCGAGCGGCGTCGGCTTGCTGGCTCGCGTCGCTCAGTCACTCGCTCCTT
4,400 4,420 4,440 4,460 4,480

GCGGAAGAGCGCCCAATACGCAAACCGCCTCTCCCCGCGCGTTGGCCGATTCATTAATGCAGCTGGCACGACAGGTTTCCCGACTGGAAAGCGGGCAGTGAGCGCAA
CGCCTTCTCGCGGGTTATGCGTTTGGCGGAGAGGGGCGCGCAACCGGCTAAGTAATTACGTCGACCGTGCTGTCCAAAGGGCTGACCTTTCGCCCGTCACTCGCGTT
4,500 4,520 4,540 4,560 4,580 4,600

CGCAATTAATGTGAGTTAGCTCACTCATTAGGCACCCCAGGCTTTACACTTTATGCTTCCGGCTCGTATGTTGTGTGGAATTGTGAGCGGATAACAATTTCACACAG
GCGTTAATTACACTCAATCGAGTGAGTAATCCGTGGGGTCCGAAATGTGAAATACGAAGGCCGAGCATACAACACACCTTAACACTCGCCTATTGTTAAAGTGTGTC

lac promoter LacO

4,620 4,640 4,660 4,680 4,700

GAAACAGCTATGACCATGATTACGCCAAGCTTACCAGTTCTCACACGGAACACCACTAATGGACACAAATTCGAAATACTTTGACCCTATTTTCGAGGACCTTGTCA
CTTTGTCGATACTGGTACTAATGCGGTTCGAATGGTCAAGAGTGTGCCTTGTGGTGATTACCTGTGTTTAAGCTTTATGAAACTGGGATAAAAGCTCCTGGAACAGT

M13-rev GAPDH promoter

4,720 4,740 4,760 4,780 4,800

CCTTGAGCCCAAGAGAGCCAAGATTTAAATTTTCCTATGACTTGATGCAAATTCCCAAAGCTAATAACATGCAAGACACGTACGGTCAAGAAGACATATTTGACCTC
GGAACTCGGGTTCTCTCGGTTCTAAATTTAAAAGGATACTGAACTACGTTTAAGGGTTTCGATTATTGTACGTTCTGTGCATGCCAGTTCTTCTGTATAAACTGGAG

GAPDH promoter

4,820 4,840 4,860 4,880 4,900 4,920

TTAACAGGTTCAGACGCGACTGCCTCATCAGTAAGACCCGTTGAAAAGAACTTACCTGAAAAAAACGAATATATACTAGCGTTGAATGTTAGCGTCAACAACAAGAA
AATTGTCCAAGTCTGCGCTGACGGAGTAGTCATTCTGGGCAACTTTTCTTGAATGGACTTTTTTTGCTTATATATGATCGCAACTTACAATCGCAGTTGTTGTTCTT

GAPDH promoter

4,940 4,960 4,980 5,000 5,020

GTTTAATGACGCGGAGGCCAAGGCAAAAAGATTCCTTGATTACGTAAGGGAGTTAGAATCATTTTGAATAAAAAACACGCTTTTTCAGTTCGAGTTTATCATTATCA
CAAATTACTGCGCCTCCGGTTCCGTTTTTCTAAGGAACTAATGCATTCCCTCAATCTTAGTAAAACTTATTTTTTGTGCGAAAAAGTCAAGCTCAAATAGTAATAGT

GAPDH promoter

5,040 5,060 5,080 5,100 5,120

ATACTGCCATTTCAAAGAATACGTAAATAATTAATAGTAGTGATTTTCCTAACTTTATTTAGTCAAAAAATTAGCCTTTTAATTCTGCTGTAACCCGTACATGCCCA
TATGACGGTAAAGTTTCTTATGCATTTATTAATTATCATCACTAAAAGGATTGAAATAAATCAGTTTTTTAATCGGAAAATTAAGACGACATTGGGCATGTACGGGT

GAPDH promoter

5,140 5,160 5,180 5,200 5,220 5,240

AAATAGGGGGCGGGTTACACAGAATATATAACATCGTAGGTGTCTGGGTGAACAGTTTATTCCTGGCATCCACTAAATATAATGGAGCCCGCTTTTTAAGCTGGCAT
TTTATCCCCCGCCCAATGTGTCTTATATATTGTAGCATCCACAGACCCACTTGTCAAATAAGGACCGTAGGTGATTTATATTACCTCGGGCGAAAAATTCGACCGTA

GAPDH promoter

5,260 5,280 5,300 5,320 5,340

CCAGAAAAAAAAAGAATCCCAGCACCAAAATATTGTTTTCTTCACCAACCATCAGTTCATAGGTCCATTCTCTTAGCGCAACTACAGAGAACAGGGGCACAAACAGG
GGTCTTTTTTTTTCTTAGGGTCGTGGTTTTATAACAAAAGAAGTGGTTGGTAGTCAAGTATCCAGGTAAGAGAATCGCGTTGATGTCTCTTGTCCCCGTGTTTGTCC

GAPDH promoter

5,360 5,380 5,400 5,420 5,440

CAAAAAACGGGCACAACCTCAATGGAGTGATGCAACCTGCCTGGAGTAAATGATGACACAAGGCAATTGACCCACGCATGTATCTATCTCATTTTCTTACACCTTCT
GTTTTTTGCCCGTGTTGGAGTTACCTCACTACGTTGGACGGACCTCATTTACTACTGTGTTCCGTTAACTGGGTGCGTACATAGATAGAGTAAAAGAATGTGGAAGA

GAPDH promoter

5,460 5,480 5,500 5,520 5,540 5,560

ATTACCTTCTGCTCTCTCTGATTTGGAAAAAGCTGAAAAAAAAGGTTGAAACCAGTTCCCTGAAATTATTCCCCTACTTGACTAATAAGTATATAAAGACGGTAGGT
TAATGGAAGACGAGAGAGACTAAACCTTTTTCGACTTTTTTTTCCAACTTTGGTCAAGGGACTTTAATAAGGGGATGAACTGATTATTCATATATTTCTGCCATCCA

GAPDH promoter

5,580 5,600 5,620 5,640 5,660

ATTGATTGTAATTCTGTAAATCTATTTCTTAAACTTCTTAAATTCTACTTTTATAGTTAGTCTTTTTTTTAGTTTTAAAACACCAAGAACTTAGTTTCGAATAAACA
TAACTAACATTAAGACATTTAGATAAAGAATTTGAAGAATTTAAGATGAAAATATCAATCAGAAAAAAAATCAAAATTTTGTGGTTCTTGAATCAAAGCTTATTTGT

GAPDH promoter

5,680 5,700 5,720 5,740 5,760

CACATAAACAGAATTCATGCAACTGTTCAATTTGCCATTGAAAGTTTCATTCTTTCTCGTCCTCTCTTACTTTTCTTTGCTCGTTTCTGCCagatctggcggccgcg
GTGTATTTGTCTTAAGTACGTTGACAAGTTAAACGGTAACTTTCAAAGTAAGAAAGAGCAGGAGAGAATGAAAAGAAACGAGCAAAGACGGtctagaccgccggcgc

1 2 3 4 5 6 7 8 9 10 11 12 13 14 15 16 17 18 19 20 21 22 23 24 25
M Q L F N L P L K V S F F L V L S Y F S L L V S A

Glucoamylase secretion signal

5,780 5,800 5,820 5,840 5,860 5,880

catgcgctagcctcgagGATGTGCAGTTGCAAGCATCCGGCGGCGGTTCTGTTCAGGCTGGTGGTTCTCTAAGATTAAGTTGTGCTGCTTCAGGTTATACCATCGGC
gtacgcgatcggagctcCTACACGTCAACGTTCGTAGGCCGCCGCCAAGACAAGTCCGACCACCAAGAGATTCTAATTCAACACGACGAAGTCCAATATGGTAGCCG

2 4 6 8 10 12 14 16 18 20 22 24 26 28 30
D V Q L Q A S G G G S V Q A G G S L R L S C A A S G Y T I G

anti-lys Nb

5,900 5,920 5,940 5,960 5,980

CCATACTGCATGGGTTGGTTTCGTCAAGCTCCAGGCAAAGAAAGGGAGGGAGTCGCAGCCATTAACATGGGCGGCGGTATCACATATTATGCAGACTCCGTAAAAGG
GGTATGACGTACCCAACCAAAGCAGTTCGAGGTCCGTTTCTTTCCCTCCCTCAGCGTCGGTAATTGTACCCGCCGCCATAGTGTATAATACGTCTGAGGCATTTTCC

32 34 36 38 40 42 44 46 48 50 52 54 56 58 60 62 64 66
P Y C M G W F R Q A P G K E R E G V A A I N M G G G I T Y Y A D S V K G

anti-lys Nb

6,000 6,020 6,040 6,060 6,080

TAGATTTACTATCTCTCAGGACAATGCTAAGAACACGGTCTATCTGTTAATGAATTCATTGGAGCCCGAGGACACTGCCATATACTACTGTGCCGCAGATTCAACCA
ATCTAAATGATAGAGAGTCCTGTTACGATTCTTGTGCCAGATAGACAATTACTTAAGTAACCTCGGGCTCCTGTGACGGTATATGATGACACGGCGTCTAAGTTGGT

68 70 72 74 76 78 80 82 84 86 88 90 92 94 96 98 100
R F T I S Q D N A K N T V Y L L M N S L E P E D T A I Y Y C A A D S T

anti-lys Nb

6,100 6,120 6,140 6,160 6,180 6,200

```
TTTACGCTTCCTATTACGAATGCGGACACGGATTAAGTACTGGAGGTTACGGTTATGATTCCTGGGGTCAAGGCACACAAGTCACGGTATCATCAgaatccTACCCA
AAATGCGAAGGATAATGCTTACGCCTGTGCCTAATTCATGACCTCCAATGCCAATACTAAGGACCCCAGTTCCGTGTGTTCAGTGCCATAGTAGTcttaggATGGGT
102  104  106  108  110  112  114  116  118  120  122  124  126  128  130  132
I Y A S Y Y E C G H G L S T G G Y G Y D S W G Q G T Q V T V S S
anti-lys Nb
6,220  6,240  6,260  6,280  6,300

TACGATGTTCCAGATTACGCTGCTGGAGGTTCGAGCGCCAAAAGCTCTTTTATCTCAACCACTACTACTGATTTAACAAGTATAAACACTAGTGCGTATTCCACTGG
ATGCTACAAGGTCTAATGCGACGACCTCCAAGCTCGCGGTTTTCGAGAAAATAGAGTTGGTGATGATGACTAAATTGTTCATATTTGTGATCACGCATAAGGTGACC
2  4  6  8  10  12  14  16  18  20  22  24
S A K S S F I S T T T T D L T S I N T S A Y S T G
HA tag
3' Half of α-agglutinin
6,320  6,340  6,360  6,380  6,400  6,420

ATCCATTTCCACAGTAGAAACAGGCAATCGAACTACATCAGAAGTGATCAGTCATGTGGTGACTACCAGCACAAAACTGTCTCCAACTGCTACTACCAGCCTGACAA
TAGGTAAAGGTGTCATCTTTGTCCGTTAGCTTGATGTAGTCTTCACTAGTCAGTACACCACTGATGGTCGTGTTTTGACAGAGGTTGACGATGATGGTCGGACTGTT
26  28  30  32  34  36  38  40  42  44  46  48  50  52  54  56  58  60
S I S T V E T G N R T T S E V I S H V V T T S T K L S P T A T T S L T
3' Half of α-agglutinin
6,440  6,460  6,480  6,500  6,520

TTGCACAAACCAGTATCTATTCTACTGACTCATATATCACAGTAGGAACAGATATTCACACCACATCAGAAGTGATTAGTGATGTGGAAACCATTAGCAGAGAAACA
AACGTGTTTGGTCATAGATAAGATGACTGAGTATATAGTGTCATCCTTGTCTATAAGTGTGGTGTAGTCTTCACTAATCACTACACCTTTGGTAATCGTCTCTTTGT
62  64  66  68  70  72  74  76  78  80  82  84  86  88  90  92  94  96
I A Q T S I Y S T D S Y I T V G T D I H T T S E V I S D V E T I S R E T
3' Half of α-agglutinin
6,540  6,560  6,580  6,600  6,620

GCTTCGACCGTTGTAGCCGCTCCAACCTCAACAACTGGATGGACAGGCGCTATGAATACTTACATCTCGCAATTTACATCCTCTTCTTTCGCAACAATCAACAGCAC
CGAAGCTGGCAACATCGGCGAGGTTGGAGTTGTTGACCTACCTGTCCGCGATACTTATGAATGTAGAGCGTTAAATGTAGGAGAAGAAAGCGTTGTTAGTTGTCGTG
98  100  102  104  106  108  110  112  114  116  118  120  122  124  126  128  130  132
A S T V V A A P T S T T G W T G A M N T Y I S Q F T S S S F A T I N S T
3' Half of α-agglutinin
6,640  6,660  6,680  6,700  6,720  6,740

ACCAATAATCTCTTCATCAGCAGTATTTGAAACCTCAGATGCTTCAATTGTCAATGTGCACACTGAAAATATCACGAATACTGCTGCTGTTCCATCTGAAGAGCCCA
TGGTTATTAGAGAAGTAGTCGTCATAAACTTTGGAGTCTACGAAGTTAACAGTTACACGTGTGACTTTTATAGTGCTTATGACGACGACAAGGTAGACTTCTCGGGT
134  136  138  140  142  144  146  148  150  152  154  156  158  160  162  164  166
P I I S S S A V F E T S D A S I V N V H T E N I T N T A A V P S E E P
3' Half of α-agglutinin
6,760  6,780  6,800  6,820  6,840
```

```
CTTTTGTAAATGCCACGAGAAACTCCTTAAATTCCTTCTGCAGCAGCAAACAGCCATCCAGTCCCTCATCTTATACGTCTTCCCCACTCGTATCGTCCCTCTCCGTA
GAAAACATTTACGGTGCTCTTTGAGGAATTTAAGGAAGACGTCGTCGTTTGTCGGTAGGTCAGGGAGTAGAATATGCAGAAGGGGTGAGCATAGCAGGGAGAGGCAT
168 170 172 174 176 178 180 182 184 186 188 190 192 194 196 198 200 202
T F V N A T R N S L N S F C S S K Q P S S P S S Y T S S P L V S S L S V
3' Half of α-agglutinin
6,860 6,880 6,900 6,920 6,940

AGCAAAACATTACTAAGCACCAGTTTTACGCCTTCTGTGCCAACATCTAATACATATATCAAAACGAAAAATACGGGTTACTTTGAGCACACGGCTTTGACAACATC
TCGTTTTGTAATGATTCGTGGTCAAAATGCGGAAGACACGGTTGTAGATTATGTATATAGTTTTGCTTTTTATGCCCAATGAAACTCGTGTGCCGAAACTGTTGTAG
204 206 208 210 212 214 216 218 220 222 224 226 228 230 232 234 236 238
S K T L L S T S F T P S V P T S N T Y I K T K N T G Y F E H T A L T T S
3' Half of α-agglutinin
6,960 6,980 7,000 7,020 7,040 7,060

TTCAGTTGGCCTTAATTCTTTTAGTGAAACAGCAGTCTCATCTCAGGGAACGAAAATTGACACCTTTTTAGTGTCATCCTTGATCGCATATCCTTCTTCTGCATCAG
AAGTCAACCGGAATTAAGAAAATCACTTTGTCGTCAGAGTAGAGTCCCTTGCTTTTAACTGTGGAAAAATCACAGTAGGAACTAGCGTATAGGAAGAAGACGTAGTC
240 242 244 246 248 250 252 254 256 258 260 262 264 266 268 270 272 274
S V G L N S F S E T A V S S Q G T K I D T F L V S S L I A Y P S S A S
3' Half of α-agglutinin
7,080 7,100 7,120 7,140 7,160

GAAGCCAATTGTCCGGTATCCAACAGAATTTCACATCAACTTCTCTCATGATTTCAACCTATGAAGGTAAAGCGTCTATATTTTTCTCAGCTGAGCTCGGTTCGATC
CTTCGGTTAACAGGCCATAGGTTGTCTTAAAGTGTAGTTGAAGAGAGTACTAAAGTTGGATACTTCCATTTCGCAGATATAAAAAGAGTCGACTCGAGCCAAGCTAG
276 278 280 282 284 286 288 290 292 294 296 298 300 302 304 306 308 310
G S Q L S G I Q Q N F T S T S L M I S T Y E G K A S I F F S A E L G S I
3' Half of α-agglutinin
7,180 7,200 7,220 7,240 7,260

ATTTTTCTGCTTTTGTCGTACCTGCTATTCTAAAACGGGTACTGTACAGTTAGTACATTGAGTCGAAATATACGAAATTATTGTTCATAATTTTCATCCTGGCTCTT
TAAAAAGACGAAAACAGCATGGACGATAAGATTTTGCCCATGACATGTCAATCATGTAACTCAGCTTTATATGCTTTAATAACAAGTATTAAAAGTAGGACCGAGAA
312 314 316 318 320
I F L L L S Y L L F *
3' Half of α-agglutinin
7,280 7,300 7,320 7,340 7,360 7,380

TTTTTCTTCAACCATAGTTAAATGGACAGTTCATATCTTAACTCTAATAATACTTTTCTAGTTCTTATCCTTTTCCGTCTCACCGCAGATTTTATCATAGTATTAAA
AAAAAGAAGTTGGTATCAATTTACCTGTCAAGTATAGAATTGAGATTATTATGAAAAGATCAAGAATAGGAAAAGGCAGAGTGGCGTCTAAAATAGTATCATAATTT
3' Half of α-agglutinin
7,400 7,420 7,440 7,460 7,480

TTTATATTTTGTTCGTAAAAAGAAAAATTTGTGAGCGTTACCGCTCGTTTCATTACCCGAAGGCTGTTTCAGTAGACCACTGATTAAGTAAGTAGATGAAAAAATTT
AAATATAAAACAAGCATTTTTCTTTTTAAACACTCGCAATGGCGAGCAAAGTAATGGGCTTCCGACAAAGTCATCTGGTGACTAATTCATTCATCTACTTTTTTAAA
3' Half of α-agglutinin
7,500 7,520 7,540 7,560 7,580
```

CATCACCATGAAAGAGTTCGATGAGAGCTACTTTTTCAAATGCTTAACAGCTAACCGCCATTCAATAATGTTACGCTCTCTTCATTCTGCGGCTACGTTATCTAACA
GTAGTGGTACTTTCTCAAGCTACTCTCGATGAAAAAGTTTACGAATTGTCGATTGGCGGTAAGTTATTACAATGCGAGAGAAGTAAGACGCCGATGCAATAGATTGT

3' Half of α-agglutinin

7,600 7,620 7,640 7,660 7,680 7,700

AGAGGTTTTACTCTCTCATATCTCATTCAAATAGAAAGAACATAATCAAAGGTACCGCGATGTAGTAAAACTAGCTAGACCGAGAAAGAGACTAGAAATGCAAAAGG
TCTCCAAAATGAGAGAGTATAGAGTAAGTTTATCTTTCTTGTATTAGTTTCCATGGCGCTACATCATTTTGATCGATCTGGCTCTTTCTCTGATCTTTACGTTTTCC

3' Half of α-agglutinin

7,720 7,740 7,760 7,780 7,800

CACTTCTACAATGGCTGCCATCATTATTATCCGATGTGACGCTGCATTTTTTTTTTTTTTTTTTTTTTTTTTTTTTTTTTTTTTTTTTTTTTTTTTTGTACAAATATCATA
GTGAAGATGTTACCGACGGTAGTAATAATAGGCTACACTGCGACGTAAAAAAAAAAAAAAAAAAAAAAAAAAAAAAAAAAAAAAAAAAAAAAAAAAACATGTTTATAGTAT

7,820 7,840 7,860 7,880 7,900

AAAAAAGAGAATCTTTTTAAGCAAGGATTTTCTTAACTTCTTCGGCGACAGCATCACCGACTTCGGTGGTACTGTTGGAACCACCTAAATCACCAGTTCTGATACCT
TTTTTTCTCTTAGAAAAATTCGTTCCTAAAAGAATTGAAGAAGCCGCTGTCGTAGTGGCTGAAGCCACCATGACAACCTTGGTGGATTTAGTGGTCAAGACTATGGA

7,920 7,940 7,960 7,980 8,000 8,020

GCATCCAAAACCTTTTTAACTGCATCTTCAATGGCCTTACCTTCTTCAGGCAAGTTCAATGACAATTTCAACATCATTGCAGCAGACAAGATAGTGGCGATAGGGTC
CGTAGGTTTTGGAAAAATTGACGTAGAAGTTACCGGAATGGAAGAAGTCCGTTCAAGTTACTGTTAAAGTTGTAGTAACGTCGTCTGTTCTATCACCGCTATCCCAG

8,040 8,060 8,080 8,100 8,120

AACCTTATTCTTTGGCAAATCTGGAGCAGAACCGTGGCATGGTTCGTACAAACCAAATGCGGTGTTCTTGTCTGGCAAAGAGGCCAAGGACGCAGATGGCAACAAAC
TTGGAATAAGAAACCGTTTAGACCTCGTCTTGGCACCGTACCAAGCATGTTTGGTTTACGCCACAAGAACAGACCGTTTCTCCGGTTCCTGCGTCTACCGTTGTTTG

8,140 8,160 8,180 8,200 8,220

CCAAGGAACCTGGGATAACGGAGGCTTCATCGGAGATGATATCACCAAACATGTTGCTGGTGATTATAATACCATTTAGGTGGGTTGGGTTCTTAACTAGGATCATG
GGTTCCTTGGACCCTATTGCCTCCGAAGTAGCCTCTACTATAGTGGTTTGTACAACGACCACTAATATTATGGTAAATCCACCCAACCCAAGAATTGATCCTAGTAC

8,240 8,260 8,280 8,300 8,320 8,340

GCGGCAGAATCAATCAATTGATGTTGAACCTTCAATGTAGGAAATTCGTTCTTGATGGTTTCCTCCACAGTTTTTCTCCATAATCTTGAAGAGGCCAAAACATTAGC
CGCCGTCTTAGTTAGTTAACTACAACTTGGAAGTTACATCCTTTAAGCAAGAACTACCAAAGGAGGTGTCAAAAAGAGGTATTAGAACTTCTCCGGTTTTGTAATCG

8,360 8,380 8,400 8,420 8,440

TTTATCCAAGGACCAAATAGGCAATGGTGGCTCATGTTGTAGGGCCATGAAAGCGGCCATTCTTGTGATTCTTTGCACTTCTGGAACGGTGTATTGTTCACTATCCC
AAATAGGTTCCTGGTTTATCCGTTACCACCGAGTACAACATCCCGGTACTTTCGCCGGTAAGAACACTAAGAAACGTGAAGACCTTGCCACATAACAAGTGATAGGG

8,460 8,480 8,500 8,520 8,540 8,560

AAGCGACACCATCACCATCGTCTTCCTTTCTCTTACCAAAGTAAATACCTCCCACTAATTCTCTGACAACAACGAAGTCAGTACCTTTAGCAAATTGTGGCTTGATT
TTCGCTGTGGTAGTGGTAGCAGAAGGAAAGAGAATGGTTTCATTTATGGAGGGTGATTAAGAGACTGTTGTTGCTTCAGTCATGGAAATCGTTTAACACCGAACTAA

8,580 8,600 8,620 8,640 8,660

GGAGATAAGTCTAAAAGAGAGTCGGATGCAAAGTTACATGGTCTTAAGTTGGCGTACAATTGAAGTTCTTTACGGATTTTTAGTAAACCTTGTTCAGGTCTAACACT
CCTCTATTCAGATTTTCTCTCAGCCTACGTTTCAATGTACCAGAATTCAACCGCATGTTAACTTCAAGAAATGCCTAAAAATCATTTGGAACAAGTCCAGATTGTGA

8,680 8,700 8,720 8,740 8,760

ACCTGTACCCCATTTAGGACCACCCACAGCACCTAACAAAACGGCATCAACCTTCTTGGAGGCTTCCAGCGCCTCATCTGGAAGTGGGACACCTGTAGCATCGATAG
TGGACATGGGGTAAATCCTGGTGGGTGTCGTGGATTGTTTTGCCGTAGTTGGAAGAACCTCCGAAGGTCGCGGAGTAGACCTTCACCCTGTGGACATCGTAGCTATC

8,780 8,800 8,820 8,840 8,860 8,880

CAGCACCACCAATTAAATGATTTTCGAAATCGAACTTGACATTGGAACGAACATCAGAAATAGCTTTAAGAACCTTAATGGCTTCGGCTGTGATTTCTTGACCAACG
GTCGTGGTGGTTAATTTACTAAAAGCTTTAGCTTGAACTGTAACCTTGCTTGTAGTCTTTATCGAAATTCTTGGAATTACCGAAGCCGACACTAAAGAACTGGTTGC

8,900 8,920 8,940 8,960 8,980

TGGTCACCTGGCAAAACGACGATCTTCTTAGGGGCAGACATTACAATGGTATATCCTTGAAATATATATAAAAAAAAAAAAAAAAAAAAAAAAAAAAAAATGCAGCT
ACCAGTGGACCGTTTTGCTGCTAGAAGAATCCCCGTCTGTAATGTTACCATATAGGAACTTTATATATATTTTTTTTTTTTTTTTTTTTTTTTTTTTTTTACGTCGA

9,000 9,020 9,040 9,060 9,080

TCTCAATGATATTCGAATACGCTTTGAGGAGATACAGCCTAATATCCGACAAACTGTTTTACAGATTTACGATCGTACTTGTTACCCATCATTGAATTTTGAACATC
AGAGTTACTATAAGCTTATGCGAAACTCCTCTATGTCGGATTATAGGCTGTTTGACAAAATGTCTAAATGCTAGCATGAACAATGGGTAGTAACTTAAAACTTGTAG

9,100 9,120 9,140 9,160 9,180 9,200

CGAACCTGGGAGTTTTCCCTGAAACAGATAGTATATTTGAACCTGTATAATAATATATAGTCTAGCGCTTTACGGAAGACAATGTATGTATTTCGGTTCCTGGAGAA
GCTTGGACCCTCAAAAGGGACTTTGTCTATCATATAAACTTGGACATATTATTATATATCAGATCGCGAAATGCCTTCTGTTACATACATAAAGCCAAGGACCTCTT

9,220 9,240 9,260 9,280 9,300

ACTATTGCATCTATTGCATAGGTAATCTTGCACGTCGCATCCCCGGTTCATTTTCTGCGTTTCCATCTTGCACTTCAATAGCATATCTTT
TGATAACGTAGATAACGTATCCATTAGAACGTGCAGCGTAGGGGCCAAGTAAAAGACGCAAAGGTAGAACGTGAAGTTATCGTATAGAAA

9,310 9,320 9,330 9,340 9,350 9,360 9,370 9,380 9,390

System 1 control strain (9000 bp)

GTTAACGAAGCATCTGTGCTTCATTTTGTAGAACAAAAATGCAACGCGAGAGCGCTAATTTTTCAAACAAAGAATCTGAGCTGCATTTTTACAGAACAGAAATGCAA
CAATTGCTTCGTAGACACGAAGTAAAACATCTTGTTTTTACGTTGCGCTCTCGCGATTAAAAAGTTTGTTTCTTAGACTCGACGTAAAAATGTCTTGTCTTTACGTT

2 micron origin

20 40 60 80 100

CGCGAAAGCGCTATTTTACCAACGAAGAATCTGTGCTTCATTTTTGTAAAACAAAAATGCAACGCGAGAGCGCTAATTTTTCAAACAAAGAATCTGAGCTGCATTTT
GCGCTTTCGCGATAAAATGGTTGCTTCTTAGACACGAAGTAAAAACATTTTGTTTTTACGTTGCGCTCTCGCGATTAAAAAGTTTGTTTCTTAGACTCGACGTAAAA

2 micron origin

120 140 160 180 200

TACAGAACAGAAATGCAACGCGAGAGCGCTATTTTACCAACAAAGAATCTATACTTCTTTTTTGTTCTACAAAAATGCATCCCGAGAGCGCTATTTTTCTAACAAAG
ATGTCTTGTCTTTACGTTGCGCTCTCGCGATAAAATGGTTGTTTCTTAGATATGAAGAAAAAACAAGATGTTTTTACGTAGGGCTCTCGCGATAAAAAGATTGTTTC

2 micron origin

220 240 260 280 300 320

CATCTTAGATTACTTTTTTTCTCCTTTGTGCGCTCTATAATGCAGTCTCTTGATAACTTTTTGCACTGTAGGTCCGTTAAGGTTAGAAGAAGGCTACTTTGGTGTCT
GTAGAATCTAATGAAAAAAAGAGGAAACACGCGAGATATTACGTCAGAGAACTATTGAAAAACGTGACATCCAGGCAATTCCAATCTTCTTCCGATGAAACCACAGA

2 micron origin

340 360 380 400 420

ATTTTCTCTTCCATAAAAAAAGCCTGACTCCACTTCCCGCGTTTACTGATTACTAGCGAAGCTGCGGGTGCATTTTTTCAAGATAAAGGCATCCCCGATTATATTCT
TAAAAGAGAAGGTATTTTTTTCGGACTGAGGTGAAGGGCGCAAATGACTAATGATCGCTTCGACGCCCACGTAAAAAAGTTCTATTTCCGTAGGGGCTAATATAAGA

2 micron origin

440 460 480 500 520

ATACCGATGTGGATTGCGCATACTTTGTGAACAGAAAGTGATAGCGTTGATGATTCTTCATTGGTCAGAAAATTATGAACGGTTTCTTCTATTTTGTCTCTATATAC
TATGGCTACACCTAACGCGTATGAAACACTTGTCTTTCACTATCGCAACTACTAAGAAGTAACCAGTCTTTTAATACTTGCCAAAGAAGATAAAACAGAGATATATG

2 micron origin

540 560 580 600 620 640

TACGTATAGGAAATGTTTACATTTTCGTATTGTTTTCGATTCACTCTATGAATAGTTCTTACTACAATTTTTTTGTCTAAAGAGTAATACTAGAGATAAACATAAAA
ATGCATATCCTTTACAAATGTAAAAGCATAACAAAAGCTAAGTGAGATACTTATCAAGAATGATGTTAAAAAAACAGATTTCTCATTATGATCTCTATTTGTATTTT

2 micron origin

660 680 700 720 740

AATGTAGAGGTCGAGTTTAGATGCAAGTTCAAGGAGCGAAAGGTGGATGGGTAGGTTATATAGGGATATAGCACAGAGATATATAGCAAAGAGATACTTTTGAGCAA
TTACATCTCCAGCTCAAATCTACGTTCAAGTTCCTCGCTTTCCACCTACCCATCCAATATATCCCTATATCGTGTCTCTATATATCGTTTCTCTATGAAAACTCGTT

2 micron origin

760 780 800 820 840

TGTTTGTGGAAGCGGTATTCGCAATATTTTAGTAGCTCGTTACAGTCCGGTGCGTTTTTGGTTTTTTGAAAGTGCGTCTTCAGAGCGCTTTTGGTTTTCAAAAGCGC
ACAAACACCTTCGCCATAAGCGTTATAAAATCATCGAGCAATGTCAGGCCACGCAAAAACCAAAAAACTTTCACGCAGAAGTCTCGCGAAAACCAAAAGTTTTCGCG

2 micron origin

860 880 900 920 940 960

TCTGAAGTTCCTATACTTTCTAGCTAGAGAATAGGAACTTCGGAATAGGAACTTCAAAGCGTTTCCGAAAACGAGCGCTTCCGAAAATGCAACGCGAGCTGCGCACA
AGACTTCAAGGATATGAAAGATCGATCTCTTATCCTTGAAGCCTTATCCTTGAAGTTTCGCAAAGGCTTTTGCTCGCGAAGGCTTTTACGTTGCGCTCGACGCGTGT

2 micron origin

980 1,000 1,020 1,040 1,060

TACAGCTCACTGTTCACGTCGCACCTATATCTGCGTGTTGCCTGTATATATATATACATGAGAAGAACGGCATAGTGCGTGTTTATGCTTAAATGCGTACTTATATG
ATGTCGAGTGACAAGTGCAGCGTGGATATAGACGCACAACGGACATATATATATATGTACTCTTCTTGCCGTATCACGCACAAATACGAATTTACGCATGAATATAC

2 micron origin

1,080 1,100 1,120 1,140 1,160

CGTCTATTTATGTAGGATGAAAGGTAGTCTAGTACCTCCTGTGATATTATCCCATTCCATGCGGGGTATCGTATGCTTCCTTCAGCACTACCCTTTAGCTGTTCTAT
GCAGATAAATACATCCTACTTTCCATCAGATCATGGAGGACACTATAATAGGGTAAGGTACGCCCCATAGCATACGAAGGAAGTCGTGATGGGAAATCGACAAGATA

2 micron origin

1,180 1,200 1,220 1,240 1,260 1,280

ATGCTGCCACTCCTCAATTGGATTAGTCTCATCCTTCAATGCTATCATTTCCTTTGATATTGGATCGATCCGATGATAAGCTGTCAAACATGAGAATTGGGTAATAA
TACGACGGTGAGGAGTTAACCTAATCAGAGTAGGAAGTTACGATAGTAAAGGAAACTATAACCTAGCTAGGCTACTATTCGACAGTTTGTACTCTTAACCCATTATT

2 micron origin URA3

1,300 1,320 1,340 1,360 1,380

CTGATATAATTAAATTGAAGCTCTAATTTGTGAGTTTAGTATACATGCATTTACTTATAATACAGTTTTTTAGTTTTGCTGGCCGCATCTTCTCAAATATGCTTCCC
GACTATATTAATTTAACTTCGAGATTAAACACTCAAATCATATGTACGTAAATGAATATTATGTCAAAAAATCAAAACGACCGGCGTAGAAGAGTTTATACGAAGGG

URA3

1,400 1,420 1,440 1,460 1,480

AGCCTGCTTTTCTGTAACGTTCACCCTCTACCTTAGCATCCCTTCCCTTTGCAAATAGTCCTCTTCCAACAATAATAATGTCAGATCCTGTAGAGACCACATCATCC
TCGGACGAAAAGACATTGCAAGTGGGAGATGGAATCGTAGGGAAGGGAAACGTTTATCAGGAGAAGGTTGTTATTATTACAGTCTAGGACATCTCTGGTGTAGTAGG

URA3

1,500 1,520 1,540 1,560 1,580 1,600

ACGGTTCTATACTGTTGACCCAATGCGTCTCCCTTGTCATCTAAACCCACACCGGGTGTCATAATCAACCAATCGTAACCTTCATCTCTTCCACCCATGTCTCTTTG
TGCCAAGATATGACAACTGGGTTACGCAGAGGGAACAGTAGATTTGGGTGTGGCCCACAGTATTAGTTGGTTAGCATTGGAAGTAGAGAAGGTGGGTACAGAGAAAC

URA3

1,620 1,640 1,660 1,680 1,700

AGCAATAAAGCCGATAACAAAATCTTTGTCGCTCTTCGCAATGTCAACAGTACCCTTAGTATATTCTCCAGTAGATAGGGAGCCCTTGCATGACAATTCTGCTAACA
TCGTTATTTCGGCTATTGTTTTAGAAACAGCGAGAAGCGTTACAGTTGTCATGGGAATCATATAAGAGGTCATCTATCCCTCGGGAACGTACTGTTAAGACGATTGT
URA3
1,720 1,740 1,760 1,780 1,800

TCAAAAGGCCTCTAGGTTCCTTTGTTACTTCTTCTGCCGCCTGCTTCAAACCGCTAACAATACCTGGGCCCACCACACCGTGTGCATTCGTAATGTCTGCCCATTCT
AGTTTTCCGGAGATCCAAGGAAACAATGAAGAAGACGGCGGACGAAGTTTGGCGATTGTTATGGACCCGGGTGGTGTGGCACACGTAAGCATTACAGACGGGTAAGA
URA3
1,820 1,840 1,860 1,880 1,900 1,920

GCTATTCTGTATACACCCGCAGAGTACTGCAATTTGACTGTATTACCAATGTCAGCAAATTTTCTGTCTTCGAAGAGTAAAAAATTGTACTTGGCGGATAATGCCTT
CGATAAGACATATGTGGGCGTCTCATGACGTTAAACTGACATAATGGTTACAGTCGTTTAAAAGACAGAAGCTTCTCATTTTTTAACATGAACCGCCTATTACGGAA
URA3
1,940 1,960 1,980 2,000 2,020

TAGCGGCTTAACTGTGCCCTCCATGGAAAAATCAGTCAAGATATCCACATGTGTTTTTAGTAAACAAATTTTGGGACCTAATGCTTCAACTAACTCCAGTAATTCCT
ATCGCCGAATTGACACGGGAGGTACCTTTTTAGTCAGTTCTATAGGTGTACACAAAAATCATTTGTTTAAAACCCTGGATTACGAAGTTGATTGAGGTCATTAAGGA
URA3
2,040 2,060 2,080 2,100 2,120 2,140

TGGTGGTACGAACATCCAATGAAGCACACAAGTTTGTTTGCTTTTCGTGCATGATATTAAATAGCTTGGCAGCAACAGGACTAGGATGAGTAGCAGCACGTTCCTTA
ACCACCATGCTTGTAGGTTACTTCGTGTGTTCAAACAAACGAAAAGCACGTACTATAATTTATCGAACCGTCGTTGTCCTGATCCTACTCATCGTCGTGCAAGGAAT
URA3
2,160 2,180 2,200 2,220 2,240

TATGTAGCTTTCGACATGATTTATCTTCGTTTCCTGCATGTTTTTGTTCTGTGCAGTTGGGTTAAGAATACTGGGCAATTTCATGTTTCTTCAACACTACATATGCG
ATACATCGAAAGCTGTACTAAATAGAAGCAAAGGACGTACAAAAACAAGACACGTCAACCCAATTCTTATGACCCGTTAAAGTACAAAGAAGTTGTGATGTATACGC
URA3
2,260 2,280 2,300 2,320 2,340

TATATATACCAATCTAAGTCTGTGCTCCTTCCTTCGTTCTTCCTTCTGTTCGGAGATTACCGAATCAAAAAAATTTCAAAGAAACCGAAATCAAAAAAAAGAATAAA
ATATATATGGTTAGATTCAGACACGAGGAAGGAAGCAAGAAGGAAGACAAGCCTCTAATGGCTTAGTTTTTTTAAAGTTTCTTTGGCTTTAGTTTTTTTTCTTATTT
URA3
2,360 2,380 2,400 2,420 2,440 2,460

AAAAAAATGATGAATTGAATTGAAAAGCTAATTCTTGAAGACGAAAGGGCCTCGTGATACGCCTATTTTTATAGGTTAATGTCATGATAATAATGGTTTCTTAGACG
TTTTTTTACTACTTAACTTAACTTTTCGATTAAGAACTTCTGCTTTCCCGGAGCACTATGCGGATAAAAATATCCAATTACAGTACTATTATTACCAAAGAATCTGC
URA3
2,480 2,500 2,520 2,540 2,560

TCAGGTGGCACTTTTCGGGGAAATGTGCGCGGAACCCCTATTTGTTTATTTTTCTAAATACATTCAAATATGTATCCGCTCATGAGACAATAACCCTGATAAATGCT
AGTCCACCGTGAAAAGCCCCTTTACACGCGCCTTGGGGATAAACAAATAAAAAGATTTATGTAAGTTTATACATAGGCGAGTACTCTGTTATTGGGACTATTTACGA
2,580 2,600 2,620 2,640 2,660

TCAATAATATTGAAAAAGGAAGAGTATGAGTATTCAACATTTCCGTGTCGCCCTTATTCCCTTTTTTGCGGCATTTTGCCTTCCTGTTTTTGCTCACCCAGAAACGC
AGTTATTATAACTTTTTCCTTCTCATACTCATAAGTTGTAAAGGCACAGCGGGAATAAGGGAAAAAACGCCGTAAAACGGAAGGACAAAAACGAGTGGGTCTTTGCG

2,680 2,700 2,720 2,740 2,760 2,780

TGGTGAAAGTAAAAGATGCTGAAGATCAGTTGGGTGCACGAGTGGGTTACATCGAACTGGATCTCAACAGCGGTAAGATCCTTGAGAGTTTTCGCCCCGAAGAACGT
ACCACTTTCATTTTCTACGACTTCTAGTCAACCCACGTGCTCACCCAATGTAGCTTGACCTAGAGTTGTCGCCATTCTAGGAACTCTCAAAAGCGGGGCTTCTTGCA

2,800 2,820 2,840 2,860 2,880

TTTCCAATGATGAGCACTTTTAAAGTTCTGCTATGTGGCGCGGTATTATCCCGTATTGACGCCGGGCAAGAGCAACTCGGTCGCCGCATACACTATTCTCAGAATGA
AAAGGTTACTACTCGTGAAAATTTCAAGACGATACACCGCGCCATAATAGGGCATAACTGCGGCCCGTTCTCGTTGAGCCAGCGGCGTATGTGATAAGAGTCTTACT

AmpR

2,900 2,920 2,940 2,960 2,980

CTTGGTTGAGTACTCACCAGTCACAGAAAAGCATCTTACGGATGGCATGACAGTAAGAGAATTATGCAGTGCTGCCATAACCATGAGTGATAACACTGCGGCCAACT
GAACCAACTCATGAGTGGTCAGTGTCTTTTCGTAGAATGCCTACCGTACTGTCATTCTCTTAATACGTCACGACGGTATTGGTACTCACTATTGTGACGCCGGTTGA

AmpR

3,000 3,020 3,040 3,060 3,080 3,100

TACTTCTGACAACGATCGGAGGACCGAAGGAGCTAACCGCTTTTTTGCACAACATGGGGGATCATGTAACTCGCCTTGATCGTTGGGAACCGGAGCTGAATGAAGCC
ATGAAGACTGTTGCTAGCCTCCTGGCTTCCTCGATTGGCGAAAAAACGTGTTGTACCCCCTAGTACATTGAGCGGAACTAGCAACCCTTGGCCTCGACTTACTTCGG

AmpR

3,120 3,140 3,160 3,180 3,200

ATACCAAACGACGAGCGTGACACCACGATGCCTGTAGCAATGGCAACAACGTTGCGCAAACTATTAACTGGCGAACTACTTACTCTAGCTTCCCGGCAACAATTAAT
TATGGTTTGCTGCTCGCACTGTGGTGCTACGGACATCGTTACCGTTGTTGCAACGCGTTTGATAATTGACCGCTTGATGAATGAGATCGAAGGGCCGTTGTTAATTA

AmpR

3,220 3,240 3,260 3,280 3,300

AGACTGGATGGAGGCGGATAAAGTTGCAGGACCACTTCTGCGCTCGGCCCTTCCGGCTGGCTGGTTTATTGCTGATAAATCTGGAGCCGGTGAGCGTGGGTCTCGCG
TCTGACCTACCTCCGCCTATTTCAACGTCCTGGTGAAGACGCGAGCCGGGAAGGCCGACCGACCAAATAACGACTATTTAGACCTCGGCCACTCGCACCCAGAGCGC

AmpR

3,320 3,340 3,360 3,380 3,400 3,420

GTATCATTGCAGCACTGGGGCCAGATGGTAAGCCCTCCCGTATCGTAGTTATCTACACGACGGGGAGTCAGGCAACTATGGATGAACGAAATAGACAGATCGCTGAG
CATAGTAACGTCGTGACCCCGGTCTACCATTCGGGAGGGCATAGCATCAATAGATGTGCTGCCCCTCAGTCCGTTGATACCTACTTGCTTTATCTGTCTAGCGACTC

AmpR

3,440 3,460 3,480 3,500 3,520

ATAGGTGCCTCACTGATTAAGCATTGGTAACTGTCAGACCAAGTTTACTCATATATACTTTAGATTGATTTAAAACTTCATTTTTAATTTAAAAGGATCTAGGTGAA
TATCCACGGAGTGACTAATTCGTAACCATTGACAGTCTGGTTCAAATGAGTATATATGAAATCTAACTAAATTTTGAAGTAAAAATTAAATTTTCCTAGATCCACTT

AmpR

3,540 3,560 3,580 3,600 3,620

GATCCTTTTTGATAATCTCATGACCAAAATCCCTTAACGTGAGTTTTCGTTCCACTGAGCGTCAGACCCCGTAGAAAAGATCAAAGGATCTTCTTGAGATCCTTTTT
CTAGGAAAAACTATTAGAGTACTGGTTTTAGGGAATTGCACTCAAAAGCAAGGTGACTCGCAGTCTGGGGCATCTTTTCTAGTTTCCTAGAAGAACTCTAGGAAAAA
ColE1 origin
3,640 3,660 3,680 3,700 3,720 3,740

TTCTGCGCGTAATCTGCTGCTTGCAAACAAAAAAACCACCGCTACCAGCGGTGGTTTGTTTGCCGGATCAAGAGCTACCAACTCTTTTTCCGAAGGTAACTGGCTTC
AAGACGCGCATTAGACGACGAACGTTTGTTTTTTTGGTGGCGATGGTCGCCACCAAACAAACGGCCTAGTTCTCGATGGTTGAGAAAAAGGCTTCCATTGACCGAAG
ColE1 origin
3,760 3,780 3,800 3,820 3,840

AGCAGAGCGCAGATACCAAATACTGTCCTTCTAGTGTAGCCGTAGTTAGGCCACCACTTCAAGAACTCTGTAGCACCGCCTACATACCTCGCTCTGCTAATCCTGTT
TCGTCTCGCGTCTATGGTTTATGACAGGAAGATCACATCGGCATCAATCCGGTGGTGAAGTTCTTGAGACATCGTGGCGGATGTATGGAGCGAGACGATTAGGACAA
ColE1 origin
3,860 3,880 3,900 3,920 3,940

ACCAGTGGCTGCTGCCAGTGGCGATAAGTCGTGTCTTACCGGGTTGGACTCAAGACGATAGTTACCGGATAAGGCGCAGCGGTCGGGCTGAACGGGGGGTTCGTGCA
TGGTCACCGACGACGGTCACCGCTATTCAGCACAGAATGGCCCAACCTGAGTTCTGCTATCAATGGCCTATTCCGCGTCGCCAGCCCGACTTGCCCCCCAAGCACGT
ColE1 origin
3,960 3,980 4,000 4,020 4,040 4,060

CACAGCCCAGCTTGGAGCGAACGACCTACACCGAACTGAGATACCTACAGCGTGAGCTATGAGAAAGCGCCACGCTTCCCGAAGGGAGAAAGGCGGACAGGTATCCG
GTGTCGGGTCGAACCTCGCTTGCTGGATGTGGCTTGACTCTATGGATGTCGCACTCGATACTCTTTCGCGGTGCGAAGGGCTTCCCTCTTTCCGCCTGTCCATAGGC
ColE1 origin
4,080 4,100 4,120 4,140 4,160

GTAAGCGGCAGGGTCGGAACAGGAGAGCGCACGAGGGAGCTTCCAGGGGGAAACGCCTGGTATCTTTATAGTCCTGTCGGGTTTCGCCACCTCTGACTTGAGCGTCG
CATTCGCCGTCCCAGCCTTGTCCTCTCGCGTGCTCCCTCGAAGGTCCCCCTTTGCGGACCATAGAAATATCAGGACAGCCCAAAGCGGTGGAGACTGAACTCGCAGC
ColE1 origin
4,180 4,200 4,220 4,240 4,260 4,280

ATTTTTGTGATGCTCGTCAGGGGGGCGGAGCCTATGGAAAAACGCCAGCAACGCGGCCTTTTTACGGTTCCTGGCCTTTTGCTGGCCTTTTGCTCACATGTTCTTTC
TAAAAACACTACGAGCAGTCCCCCCGCCTCGGATACCTTTTTGCGGTCGTTGCGCCGGAAAAATGCCAAGGACCGGAAAACGACCGGAAAACGAGTGTACAAGAAAG
ColE1 origin
4,300 4,320 4,340 4,360 4,380

CTGCGTTATCCCCTGATTCTGTGGATAACCGTATTACCGCCTTTGAGTGAGCTGATACCGCTCGCCGCAGCCGAACGACCGAGCGCAGCGAGTCAGTGAGCGAGGAA
GACGCAATAGGGGACTAAGACACCTATTGGCATAATGGCGGAAACTCACTCGACTATGGCGAGCGGCGTCGGCTTGCTGGCTCGCGTCGCTCAGTCACTCGCTCCTT
4,400 4,420 4,440 4,460 4,480

GCGGAAGAGCGCCCAATACGCAAACCGCCTCTCCCCGCGCGTTGGCCGATTCATTAATGCAGCTGGCACGACAGGTTTCCCGACTGGAAAGCGGGCAGTGAGCGCAA
CGCCTTCTCGCGGGTTATGCGTTTGGCGGAGAGGGGCGCGCAACCGGCTAAGTAATTACGTCGACCGTGCTGTCCAAAGGGCTGACCTTTCGCCCGTCACTCGCGTT
4,500 4,520 4,540 4,560 4,580 4,600

CGCAATTAATGTGAGTTAGCTCACTCATTAGGCACCCCAGGCTTTACACTTTATGCTTCCGGCTCGTATGTTGTGTGGAATTGTGAGCGGATAACAATTTCACACAG
GCGTTAATTACACTCAATCGAGTGAGTAATCCGTGGGGTCCGAAATGTGAAATACGAAGGCCGAGCATACAACACACCTTAACACTCGCCTATTGTTAAAGTGTGTC

lac promoter LacO

4,620 4,640 4,660 4,680 4,700

GAAACAGCTATGACCATGATTACGCCAAGCTTACCAGTTCTCACACGGAACACCACTAATGGACACAAATTCGAAATACTTTGACCCTATTTTCGAGGACCTTGTCA
CTTTGTCGATACTGGTACTAATGCGGTTCGAATGGTCAAGAGTGTGCCTTGTGGTGATTACCTGTGTTTAAGCTTTATGAAACTGGGATAAAAGCTCCTGGAACAGT

M13-rev GAPDH promoter

4,720 4,740 4,760 4,780 4,800

CCTTGAGCCCAAGAGAGCCAAGATTTAAATTTTCCTATGACTTGATGCAAATTCCCAAAGCTAATAACATGCAAGACACGTACGGTCAAGAAGACATATTTGACCTC
GGAACTCGGGTTCTCTCGGTTCTAAATTTAAAAGGATACTGAACTACGTTTAAGGGTTTCGATTATTGTACGTTCTGTGCATGCCAGTTCTTCTGTATAAACTGGAG

GAPDH promoter

4,820 4,840 4,860 4,880 4,900 4,920

TTAACAGGTTCAGACGCGACTGCCTCATCAGTAAGACCCGTTGAAAAGAACTTACCTGAAAAAAACGAATATATACTAGCGTTGAATGTTAGCGTCAACAACAAGAA
AATTGTCCAAGTCTGCGCTGACGGAGTAGTCATTCTGGGCAACTTTTCTTGAATGGACTTTTTTTGCTTATATATGATCGCAACTTACAATCGCAGTTGTTGTTCTT

GAPDH promoter

4,940 4,960 4,980 5,000 5,020

GTTTAATGACGCGGAGGCCAAGGCAAAAAGATTCCTTGATTACGTAAGGGAGTTAGAATCATTTTGAATAAAAAACACGCTTTTTCAGTTCGAGTTTATCATTATCA
CAAATTACTGCGCCTCCGGTTCCGTTTTTCTAAGGAACTAATGCATTCCCTCAATCTTAGTAAAACTTATTTTTTGTGCGAAAAAGTCAAGCTCAAATAGTAATAGT

GAPDH promoter

5,040 5,060 5,080 5,100 5,120

ATACTGCCATTTCAAAGAATACGTAAATAATTAATAGTAGTGATTTTCCTAACTTTATTTAGTCAAAAAATTAGCCTTTTAATTCTGCTGTAACCCGTACATGCCCA
TATGACGGTAAAGTTTCTTATGCATTTATTAATTATCATCACTAAAAGGATTGAAATAAATCAGTTTTTTAATCGGAAAATTAAGACGACATTGGGCATGTACGGGT

GAPDH promoter

5,140 5,160 5,180 5,200 5,220 5,240

AAATAGGGGGCGGGTTACACAGAATATATAACATCGTAGGTGTCTGGGTGAACAGTTTATTCCTGGCATCCACTAAATATAATGGAGCCCGCTTTTTAAGCTGGCAT
TTTATCCCCCGCCCAATGTGTCTTATATATTGTAGCATCCACAGACCCACTTGTCAAATAAGGACCGTAGGTGATTTATATTACCTCGGGCGAAAAATTCGACCGTA

GAPDH promoter

5,260 5,280 5,300 5,320 5,340

CCAGAAAAAAAAAGAATCCCAGCACCAAAATATTGTTTTCTTCACCAACCATCAGTTCATAGGTCCATTCTCTTAGCGCAACTACAGAGAACAGGGGCACAAACAGG
GGTCTTTTTTTTTCTTAGGGTCGTGGTTTTATAACAAAAGAAGTGGTTGGTAGTCAAGTATCCAGGTAAGAGAATCGCGTTGATGTCTCTTGTCCCCGTGTTTGTCC

GAPDH promoter

5,360 5,380 5,400 5,420 5,440

CAAAAAACGGGCACAACCTCAATGGAGTGATGCAACCTGCCTGGAGTAAATGATGACACAAGGCAATTGACCCACGCATGTATCTATCTCATTTTCTTACACCTTCT
GTTTTTTGCCCGTGTTGGAGTTACCTCACTACGTTGGACGGACCTCATTTACTACTGTGTTCCGTTAACTGGGTGCGTACATAGATAGAGTAAAAGAATGTGGAAGA

GAPDH promoter

5,460 5,480 5,500 5,520 5,540 5,560

ATTACCTTCTGCTCTCTCTGATTTGGAAAAAGCTGAAAAAAAAGGTTGAAACCAGTTCCCTGAAATTATTCCCCTACTTGACTAATAAGTATATAAAGACGGTAGGT
TAATGGAAGACGAGAGAGACTAAACCTTTTTCGACTTTTTTTTCCAACTTTGGTCAAGGGACTTTAATAAGGGGATGAACTGATTATTCATATATTTCTGCCATCCA

GAPDH promoter

5,580 5,600 5,620 5,640 5,660

ATTGATTGTAATTCTGTAAATCTATTTCTTAAACTTCTTAAATTCTACTTTTATAGTTAGTCTTTTTTTTAGTTTTAAAACACCAAGAACTTAGTTTCGAATAAACA
TAACTAACATTAAGACATTTAGATAAAGAATTTGAAGAATTTAAGATGAAAATATCAATCAGAAAAAAAATCAAAATTTTGTGGTTCTTGAATCAAAGCTTATTTGT

GAPDH promoter

5,680 5,700 5,720 5,740 5,760

CACATAAACAGAATTCATGCAACTGTTCAATTTGCCATTGAAAGTTTCATTCTTTCTCGTCCTCTCTTACTTTTCTTTGCTCGTTTCTGCCagatctggcggccgcg
GTGTATTTGTCTTAAGTACGTTGACAAGTTAAACGGTAACTTTCAAAGTAAGAAAGAGCAGGAGAGAATGAAAAGAAACGAGCAAAGACGGtctagaccgccggcgc

Glucoamylase secretion signal

5,780 5,800 5,820 5,840 5,860 5,880

catgcgctagcctcgagGGTGGATCTGATTACAAGGATGACGATGACAAGGCTGGAGGTTCGAGCGCCAAAAGCTCTTTTATCTCAACCACTACTACTGATTTAACA
gtacgcgatcggagctcCCACCTAGACTAATGTTCCTACTGCTACTGTTCCGACCTCCAAGCTCGCGGTTTTCGAGAAAATAGAGTTGGTGATGATGACTAAATTGT

2 4 6 8 10 12 14
S A K S S F I S T T T T D L T

FLAG 3' Half of α-agglutinin

5,900 5,920 5,940 5,960 5,980

AGTATAAACACTAGTGCGTATTCCACTGGATCCATTTCCACAGTAGAAACAGGCAATCGAACTACATCAGAAGTGATCAGTCATGTGGTGACTACCAGCACAAAACT
TCATATTTGTGATCACGCATAAGGTGACCTAGGTAAAGGTGTCATCTTTGTCCGTTAGCTTGATGTAGTCTTCACTAGTCAGTACACCACTGATGGTCGTGTTTTGA

16 18 20 22 24 26 28 30 32 34 36 38 40 42 44 46 48 50
S I N T S A Y S T G S I S T V E T G N R T T S E V I S H V V T T S T K L

3' Half of α-agglutinin

6,000 6,020 6,040 6,060 6,080

GTCTCCAACTGCTACTACCAGCCTGACAATTGCACAAACCAGTATCTATTCTACTGACTCATATATCACAGTAGGAACAGATATTCACACCACATCAGAAGTGATTA
CAGAGGTTGACGATGATGGTCGGACTGTTAACGTGTTTGGTCATAGATAAGATGACTGAGTATATAGTGTCATCCTTGTCTATAAGTGTGGTGTAGTCTTCACTAAT

52 54 56 58 60 62 64 66 68 70 72 74 76 78 80 82 84 86
S P T A T T S L T I A Q T S I Y S T D S Y I T V G T D I H T T S E V I

3' Half of α-agglutinin

6,100 6,120 6,140 6,160 6,180 6,200

GTGATGTGGAAACCATTAGCAGAGAAACAGCTTCGACCGTTGTAGCCGCTCCAACCTCAACAACTGGATGGACAGGCGCTATGAATACTTACATCTCGCAATTTACA
CACTACACCTTTGGTAATCGTCTCTTTGTCGAAGCTGGCAACATCGGCGAGGTTGGAGTTGTTGACCTACCTGTCCGCGATACTTATGAATGTAGAGCGTTAAATGT
88 90 92 94 96 98 100 102 104 106 108 110 112 114 116 118 120 122
S D V E T I S R E T A S T V V A A P T S T T G W T G A M N T Y I S Q F T

3' Half of α-agglutinin

6,220 6,240 6,260 6,280 6,300

TCCTCTTCTTTCGCAACAATCAACAGCACACCAATAATCTCTTCATCAGCAGTATTTGAAACCTCAGATGCTTCAATTGTCAATGTGCACACTGAAAATATCACGAA
AGGAGAAGAAAGCGTTGTTAGTTGTCGTGTGGTTATTAGAGAAGTAGTCGTCATAAACTTTGGAGTCTACGAAGTTAACAGTTACACGTGTGACTTTTATAGTGCTT
124 126 128 130 132 134 136 138 140 142 144 146 148 150 152 154 156 158
S S S F A T I N S T P I I S S S A V F E T S D A S I V N V H T E N I T N

3' Half of α-agglutinin

6,320 6,340 6,360 6,380 6,400 6,420

TACTGCTGCTGTTCCATCTGAAGAGCCCACTTTTGTAAATGCCACGAGAAACTCCTTAAATTCCTTCTGCAGCAGCAAACAGCCATCCAGTCCCTCATCTTATACGT
ATGACGACGACAAGGTAGACTTCTCGGGTGAAAACATTTACGGTGCTCTTTGAGGAATTTAAGGAAGACGTCGTCGTTTGTCGGTAGGTCAGGGAGTAGAATATGCA
160 162 164 166 168 170 172 174 176 178 180 182 184 186 188 190 192
T A A V P S E E P T F V N A T R N S L N S F C S S K Q P S S P S S Y T

3' Half of α-agglutinin

6,440 6,460 6,480 6,500 6,520

CTTCCCCACTCGTATCGTCCCTCTCCGTAAGCAAAACATTACTAAGCACCAGTTTTACGCCTTCTGTGCCAACATCTAATACATATATCAAAACGAAAAATACGGGT
GAAGGGGTGAGCATAGCAGGGAGAGGCATTCGTTTTGTAATGATTCGTGGTCAAAATGCGGAAGACACGGTTGTAGATTATGTATATAGTTTTGCTTTTTATGCCCA
194 196 198 200 202 204 206 208 210 212 214 216 218 220 222 224 226 228
S S P L V S S L S V S K T L L S T S F T P S V P T S N T Y I K T K N T G

3' Half of α-agglutinin

6,540 6,560 6,580 6,600 6,620

TACTTTGAGCACACGGCTTTGACAACATCTTCAGTTGGCCTTAATTCTTTTAGTGAAACAGCAGTCTCATCTCAGGGAACGAAAATTGACACCTTTTTAGTGTCATC
ATGAAACTCGTGTGCCGAAACTGTTGTAGAAGTCAACCGGAATTAAGAAAATCACTTTGTCGTCAGAGTAGAGTCCCTTGCTTTTAACTGTGGAAAAATCACAGTAG
230 232 234 236 238 240 242 244 246 248 250 252 254 256 258 260 262 264
Y F E H T A L T T S S V G L N S F S E T A V S S Q G T K I D T F L V S S

3' Half of α-agglutinin

6,640 6,660 6,680 6,700 6,720 6,740

CTTGATCGCATATCCTTCTTCTGCATCAGGAAGCCAATTGTCCGGTATCCAACAGAATTTCACATCAACTTCTCTCATGATTTCAACCTATGAAGGTAAAGCGTCTA
GAACTAGCGTATAGGAAGAAGACGTAGTCCTTCGGTTAACAGGCCATAGGTTGTCTTAAAGTGTAGTTGAAGAGAGTACTAAAGTTGGATACTTCCATTTCGCAGAT
266 268 270 272 274 276 278 280 282 284 286 288 290 292 294 296 298 300
L I A Y P S S A S G S Q L S G I Q Q N F T S T S L M I S T Y E G K A S

3' Half of α-agglutinin

6,760 6,780 6,800 6,820 6,840

TATTTTTCTCAGCTGAGCTCGGTTCGATCATTTTTCTGCTTTTGTCGTACCTGCTATTCTAAAACGGGTACTGTACAGTTAGTACATTGAGTCGAAATATACGAAAT
ATAAAAAGAGTCGACTCGAGCCAAGCTAGTAAAAAGACGAAAACAGCATGGACGATAAGATTTTGCCCATGACATGTCAATCATGTAACTCAGCTTTATATGCTTTA

302 304 306 308 310 312 314 316 318 320
I F F S A E L G S I I F L L L S Y L L F *

3' Half of α-agglutinin

6,860 6,880 6,900 6,920 6,940

TATTGTTCATAATTTTCATCCTGGCTCTTTTTTTCTTCAACCATAGTTAAATGGACAGTTCATATCTTAACTCTAATAATACTTTTCTAGTTCTTATCCTTTTCCGT
ATAACAAGTATTAAAAGTAGGACCGAGAAAAAAAGAAGTTGGTATCAATTTACCTGTCAAGTATAGAATTGAGATTATTATGAAAAGATCAAGAATAGGAAAAGGCA

3' Half of α-agglutinin

6,960 6,980 7,000 7,020 7,040 7,060

CTCACCGCAGATTTTATCATAGTATTAAATTTATATTTTGTTCGTAAAAAGAAAAATTTGTGAGCGTTACCGCTCGTTTCATTACCCGAAGGCTGTTTCAGTAGACC
GAGTGGCGTCTAAAATAGTATCATAATTTAAATATAAAACAAGCATTTTTCTTTTTAAACACTCGCAATGGCGAGCAAAGTAATGGGCTTCCGACAAAGTCATCTGG

3' Half of α-agglutinin

7,080 7,100 7,120 7,140 7,160

ACTGATTAAGTAAGTAGATGAAAAAATTTCATCACCATGAAAGAGTTCGATGAGAGCTACTTTTTCAAATGCTTAACAGCTAACCGCCATTCAATAATGTTACGCTC
TGACTAATTCATTCATCTACTTTTTTAAAGTAGTGGTACTTTCTCAAGCTACTCTCGATGAAAAAGTTTACGAATTGTCGATTGGCGGTAAGTTATTACAATGCGAG

3' Half of α-agglutinin

7,180 7,200 7,220 7,240 7,260

TCTTCATTCTGCGGCTACGTTATCTAACAAGAGGTTTTACTCTCTCATATCTCATTCAAATAGAAAGAACATAATCAAAGGTACCGCGATGTAGTAAAACTAGCTAG
AGAAGTAAGACGCCGATGCAATAGATTGTTCTCCAAAATGAGAGAGTATAGAGTAAGTTTATCTTTCTTGTATTAGTTTCCATGGCGCTACATCATTTTGATCGATC

3' Half of α-agglutinin

7,280 7,300 7,320 7,340 7,360 7,380

ACCGAGAAAGAGACTAGAAATGCAAAAGGCACTTCTACAATGGCTGCCATCATTATTATCCGATGTGACGCTGCATTTTTTTTTTTTTTTTTTTTTTTTTTTTTTTT
TGGCTCTTTCTCTGATCTTTACGTTTTCCGTGAAGATGTTACCGACGGTAGTAATAATAGGCTACACTGCGACGTAAAAAAAAAAAAAAAAAAAAAAAAAAAAAAAA

7,400 7,420 7,440 7,460 7,480

TTTTTTTTTTTTTTTGTACAAATATCATAAAAAAAGAGAATCTTTTTAAGCAAGGATTTTCTTAACTTCTTCGGCGACAGCATCACCGACTTCGGTGGTACTGTTGG
AAAAAAAAAAAAAAACATGTTTATAGTATTTTTTTCTCTTAGAAAAATTCGTTCCTAAAAGAATTGAAGAAGCCGCTGTCGTAGTGGCTGAAGCCACCATGACAACC

7,500 7,520 7,540 7,560 7,580

AACCACCTAAATCACCAGTTCTGATACCTGCATCCAAAACCTTTTTAACTGCATCTTCAATGGCCTTACCTTCTTCAGGCAAGTTCAATGACAATTTCAACATCATT
TTGGTGGATTTAGTGGTCAAGACTATGGACGTAGGTTTTGGAAAAATTGACGTAGAAGTTACCGGAATGGAAGAAGTCCGTTCAAGTTACTGTTAAAGTTGTAGTAA

7,600 7,620 7,640 7,660 7,680 7,700

GCAGCAGACAAGATAGTGGCGATAGGGTCAACCTTATTCTTTGGCAAATCTGGAGCAGAACCGTGGCATGGTTCGTACAAACCAAATGCGGTGTTCTTGTCTGGCAA
CGTCGTCTGTTCTATCACCGCTATCCCAGTTGGAATAAGAAACCGTTTAGACCTCGTCTTGGCACCGTACCAAGCATGTTTGGTTTACGCCACAAGAACAGACCGTT

7,720 7,740 7,760 7,780 7,800

AGAGGCCAAGGACGCAGATGGCAACAAACCCAAGGAACCTGGGATAACGGAGGCTTCATCGGAGATGATATCACCAAACATGTTGCTGGTGATTATAATACCATTTA
TCTCCGGTTCCTGCGTCTACCGTTGTTTGGGTTCCTTGGACCCTATTGCCTCCGAAGTAGCCTCTACTATAGTGGTTTGTACAACGACCACTAATATTATGGTAAAT

7,820 7,840 7,860 7,880 7,900

GGTGGGTTGGGTTCTTAACTAGGATCATGGCGGCAGAATCAATCAATTGATGTTGAACCTTCAATGTAGGAAATTCGTTCTTGATGGTTTCCTCCACAGTTTTTCTC
CCACCCAACCCAAGAATTGATCCTAGTACCGCCGTCTTAGTTAGTTAACTACAACTTGGAAGTTACATCCTTTAAGCAAGAACTACCAAAGGAGGTGTCAAAAAGAG

7,920 7,940 7,960 7,980 8,000 8,020

CATAATCTTGAAGAGGCCAAAACATTAGCTTTATCCAAGGACCAAATAGGCAATGGTGGCTCATGTTGTAGGGCCATGAAAGCGGCCATTCTTGTGATTCTTTGCAC
GTATTAGAACTTCTCCGGTTTTGTAATCGAAATAGGTTCCTGGTTTATCCGTTACCACCGAGTACAACATCCCGGTACTTTCGCCGGTAAGAACACTAAGAAACGTG

8,040 8,060 8,080 8,100 8,120

TTCTGGAACGGTGTATTGTTCACTATCCCAAGCGACACCATCACCATCGTCTTCCTTTCTCTTACCAAAGTAAATACCTCCCACTAATTCTCTGACAACAACGAAGT
AAGACCTTGCCACATAACAAGTGATAGGGTTCGCTGTGGTAGTGGTAGCAGAAGGAAAGAGAATGGTTTCATTTATGGAGGGTGATTAAGAGACTGTTGTTGCTTCA

8,140 8,160 8,180 8,200 8,220

CAGTACCTTTAGCAAATTGTGGCTTGATTGGAGATAAGTCTAAAAGAGAGTCGGATGCAAAGTTACATGGTCTTAAGTTGGCGTACAATTGAAGTTCTTTACGGATT
GTCATGGAAATCGTTTAACACCGAACTAACCTCTATTCAGATTTTCTCTCAGCCTACGTTTCAATGTACCAGAATTCAACCGCATGTTAACTTCAAGAAATGCCTAA

8,240 8,260 8,280 8,300 8,320 8,340

TTTAGTAAACCTTGTTCAGGTCTAACACTACCTGTACCCCATTTAGGACCACCCACAGCACCTAACAAAACGGCATCAACCTTCTTGGAGGCTTCCAGCGCCTCATC
AAATCATTTGGAACAAGTCCAGATTGTGATGGACATGGGGTAAATCCTGGTGGGTGTCGTGGATTGTTTTGCCGTAGTTGGAAGAACCTCCGAAGGTCGCGGAGTAG

8,360 8,380 8,400 8,420 8,440

TGGAAGTGGGACACCTGTAGCATCGATAGCAGCACCACCAATTAAATGATTTTCGAAATCGAACTTGACATTGGAACGAACATCAGAAATAGCTTTAAGAACCTTAA
ACCTTCACCCTGTGGACATCGTAGCTATCGTCGTGGTGGTTAATTTACTAAAAGCTTTAGCTTGAACTGTAACCTTGCTTGTAGTCTTTATCGAAATTCTTGGAATT

8,460 8,480 8,500 8,520 8,540 8,560

TGGCTTCGGCTGTGATTTCTTGACCAACGTGGTCACCTGGCAAAACGACGATCTTCTTAGGGGCAGACATTACAATGGTATATCCTTGAAATATATATAAAAAAAAA
ACCGAAGCCGACACTAAAGAACTGGTTGCACCAGTGGACCGTTTTGCTGCTAGAAGAATCCCCGTCTGTAATGTTACCATATAGGAACTTTATATATATTTTTTTTT

8,580 8,600 8,620 8,640 8,660

AAAAAAAAAAAAAAAAAAAAAATGCAGCTTCTCAATGATATTCGAATACGCTTTGAGGAGATACAGCCTAATATCCGACAAACTGTTTTACAGATTTACGATCGTAC
TTTTTTTTTTTTTTTTTTTTTTACGTCGAAGAGTTACTATAAGCTTATGCGAAACTCCTCTATGTCGGATTATAGGCTGTTTGACAAAATGTCTAAATGCTAGCATG

8,680 8,700 8,720 8,740 8,760

TTGTTACCCATCATTGAATTTTGAACATCCGAACCTGGGAGTTTTCCCTGAAACAGATAGTATATTTGAACCTGTATAATAATATATAGTCTAGCGCTTTACGGAAG
AACAATGGGTAGTAACTTAAAACTTGTAGGCTTGGACCCTCAAAAGGGACTTTGTCTATCATATAAACTTGGACATATTATTATATATCAGATCGCGAAATGCCTTC

8,780 8,800 8,820 8,840 8,860 8,880

ACAATGTATGTATTTCGGTTCCTGGAGAAACTATTGCATCTATTGCATAGGTAATCTTGCACGTCGCATCCCCGGTTCATTTTCTGCGTTTCCATCTTGCACTTCAA
TGTTACATACATAAAGCCAAGGACCTCTTTGATAACGTAGATAACGTATCCATTAGAACGTGCAGCGTAGGGGCCAAGTAAAAGACGCAAAGGTAGAACGTGAAGTT

8,900 8,920 8,940 8,960 8,980

```
TAGCATATCTTT
ATCGTATAGAAA
 8,990   9,000
```

System 2 anti-lys Nb strain (9582 bp)

GTTAACGAAGCATCTGTGCTTCATTTTGTAGAACAAAAATGCAACGCGAGAGCGCTAATTTTTCAAACAAAGAATCTGAGCTGCATTTTTACAGAACAGAAATGCAA
CAATTGCTTCGTAGACACGAAGTAAAACATCTTGTTTTTACGTTGCGCTCTCGCGATTAAAAAGTTTGTTTCTTAGACTCGACGTAAAAATGTCTTGTCTTTACGTT

2 micron origin

20 40 60 80 100

CGCGAAAGCGCTATTTTACCAACGAAGAATCTGTGCTTCATTTTTGTAAAACAAAAATGCAACGCGAGAGCGCTAATTTTTCAAACAAAGAATCTGAGCTGCATTTT
GCGCTTTCGCGATAAAATGGTTGCTTCTTAGACACGAAGTAAAAACATTTTGTTTTTACGTTGCGCTCTCGCGATTAAAAAGTTTGTTTCTTAGACTCGACGTAAAA

2 micron origin

120 140 160 180 200

TACAGAACAGAAATGCAACGCGAGAGCGCTATTTTACCAACAAAGAATCTATACTTCTTTTTTGTTCTACAAAAATGCATCCCGAGAGCGCTATTTTTCTAACAAAG
ATGTCTTGTCTTTACGTTGCGCTCTCGCGATAAAATGGTTGTTTCTTAGATATGAAGAAAAAACAAGATGTTTTTACGTAGGGCTCTCGCGATAAAAAGATTGTTTC

2 micron origin

220 240 260 280 300 320

CATCTTAGATTACTTTTTTTCTCCTTTGTGCGCTCTATAATGCAGTCTCTTGATAACTTTTTGCACTGTAGGTCCGTTAAGGTTAGAAGAAGGCTACTTTGGTGTCT
GTAGAATCTAATGAAAAAAAGAGGAAACACGCGAGATATTACGTCAGAGAACTATTGAAAAACGTGACATCCAGGCAATTCCAATCTTCTTCCGATGAAACCACAGA

2 micron origin

340 360 380 400 420

ATTTTCTCTTCCATAAAAAAAGCCTGACTCCACTTCCCGCGTTTACTGATTACTAGCGAAGCTGCGGGTGCATTTTTTCAAGATAAAGGCATCCCCGATTATATTCT
TAAAAGAGAAGGTATTTTTTTCGGACTGAGGTGAAGGGCGCAAATGACTAATGATCGCTTCGACGCCCACGTAAAAAAGTTCTATTTCCGTAGGGGCTAATATAAGA

2 micron origin

440 460 480 500 520

ATACCGATGTGGATTGCGCATACTTTGTGAACAGAAAGTGATAGCGTTGATGATTCTTCATTGGTCAGAAAATTATGAACGGTTTCTTCTATTTTGTCTCTATATAC
TATGGCTACACCTAACGCGTATGAAACACTTGTCTTTCACTATCGCAACTACTAAGAAGTAACCAGTCTTTTAATACTTGCCAAAGAAGATAAAACAGAGATATATG

2 micron origin

540 560 580 600 620 640

TACGTATAGGAAATGTTTACATTTTCGTATTGTTTTCGATTCACTCTATGAATAGTTCTTACTACAATTTTTTTGTCTAAAGAGTAATACTAGAGATAAACATAAAA
ATGCATATCCTTTACAAATGTAAAAGCATAACAAAAGCTAAGTGAGATACTTATCAAGAATGATGTTAAAAAAACAGATTTCTCATTATGATCTCTATTTGTATTTT

2 micron origin

660 680 700 720 740

AATGTAGAGGTCGAGTTTAGATGCAAGTTCAAGGAGCGAAAGGTGGATGGGTAGGTTATATAGGGATATAGCACAGAGATATATAGCAAAGAGATACTTTTGAGCAA
TTACATCTCCAGCTCAAATCTACGTTCAAGTTCCTCGCTTTCCACCTACCCATCCAATATATCCCTATATCGTGTCTCTATATATCGTTTCTCTATGAAAACTCGTT

2 micron origin

760 780 800 820 840

TGTTTGTGGAAGCGGTATTCGCAATATTTTAGTAGCTCGTTACAGTCCGGTGCGTTTTTGGTTTTTTGAAAGTGCGTCTTCAGAGCGCTTTTGGTTTTCAAAAGCGC
ACAAACACCTTCGCCATAAGCGTTATAAAATCATCGAGCAATGTCAGGCCACGCAAAAACCAAAAAACTTTCACGCAGAAGTCTCGCGAAAACCAAAAGTTTTCGCG

2 micron origin

860 880 900 920 940 960

TCTGAAGTTCCTATACTTTCTAGCTAGAGAATAGGAACTTCGGAATAGGAACTTCAAAGCGTTTCCGAAAACGAGCGCTTCCGAAAATGCAACGCGAGCTGCGCACA
AGACTTCAAGGATATGAAAGATCGATCTCTTATCCTTGAAGCCTTATCCTTGAAGTTTCGCAAAGGCTTTTGCTCGCGAAGGCTTTTACGTTGCGCTCGACGCGTGT

2 micron origin

980 1,000 1,020 1,040 1,060

TACAGCTCACTGTTCACGTCGCACCTATATCTGCGTGTTGCCTGTATATATATATACATGAGAAGAACGGCATAGTGCGTGTTTATGCTTAAATGCGTACTTATATG
ATGTCGAGTGACAAGTGCAGCGTGGATATAGACGCACAACGGACATATATATATATGTACTCTTCTTGCCGTATCACGCACAAATACGAATTTACGCATGAATATAC

2 micron origin

1,080 1,100 1,120 1,140 1,160

CGTCTATTTATGTAGGATGAAAGGTAGTCTAGTACCTCCTGTGATATTATCCCATTCCATGCGGGGTATCGTATGCTTCCTTCAGCACTACCCTTTAGCTGTTCTAT
GCAGATAAATACATCCTACTTTCCATCAGATCATGGAGGACACTATAATAGGGTAAGGTACGCCCCATAGCATACGAAGGAAGTCGTGATGGGAAATCGACAAGATA

2 micron origin

1,180 1,200 1,220 1,240 1,260 1,280

ATGCTGCCACTCCTCAATTGGATTAGTCTCATCCTTCAATGCTATCATTTCCTTTGATATTGGATCGATCCGATGATAAGCTGTCAAACATGAGAATTGGGTAATAA
TACGACGGTGAGGAGTTAACCTAATCAGAGTAGGAAGTTACGATAGTAAAGGAAACTATAACCTAGCTAGGCTACTATTCGACAGTTTGTACTCTTAACCCATTATT

2 micron origin URA3

1,300 1,320 1,340 1,360 1,380

CTGATATAATTAAATTGAAGCTCTAATTTGTGAGTTTAGTATACATGCATTTACTTATAATACAGTTTTTTAGTTTTGCTGGCCGCATCTTCTCAAATATGCTTCCC
GACTATATTAATTTAACTTCGAGATTAAACACTCAAATCATATGTACGTAAATGAATATTATGTCAAAAAATCAAAACGACCGGCGTAGAAGAGTTTATACGAAGGG

URA3

1,400 1,420 1,440 1,460 1,480

AGCCTGCTTTTCTGTAACGTTCACCCTCTACCTTAGCATCCCTTCCCTTTGCAAATAGTCCTCTTCCAACAATAATAATGTCAGATCCTGTAGAGACCACATCATCC
TCGGACGAAAAGACATTGCAAGTGGGAGATGGAATCGTAGGGAAGGGAAACGTTTATCAGGAGAAGGTTGTTATTATTACAGTCTAGGACATCTCTGGTGTAGTAGG

URA3

1,500 1,520 1,540 1,560 1,580 1,600

ACGGTTCTATACTGTTGACCCAATGCGTCTCCCTTGTCATCTAAACCCACACCGGGTGTCATAATCAACCAATCGTAACCTTCATCTCTTCCACCCATGTCTCTTTG
TGCCAAGATATGACAACTGGGTTACGCAGAGGGAACAGTAGATTTGGGTGTGGCCCACAGTATTAGTTGGTTAGCATTGGAAGTAGAGAAGGTGGGTACAGAGAAAC

URA3

1,620 1,640 1,660 1,680 1,700

AGCAATAAAGCCGATAACAAAATCTTTGTCGCTCTTCGCAATGTCAACAGTACCCTTAGTATATTCTCCAGTAGATAGGGAGCCCTTGCATGACAATTCTGCTAACA
TCGTTATTTCGGCTATTGTTTTAGAAACAGCGAGAAGCGTTACAGTTGTCATGGGAATCATATAAGAGGTCATCTATCCCTCGGGAACGTACTGTTAAGACGATTGT
URA3
1,720 1,740 1,760 1,780 1,800

TCAAAAGGCCTCTAGGTTCCTTTGTTACTTCTTCTGCCGCCTGCTTCAAACCGCTAACAATACCTGGGCCCACCACACCGTGTGCATTCGTAATGTCTGCCCATTCT
AGTTTTCCGGAGATCCAAGGAAACAATGAAGAAGACGGCGGACGAAGTTTGGCGATTGTTATGGACCCGGGTGGTGTGGCACACGTAAGCATTACAGACGGGTAAGA
URA3
1,820 1,840 1,860 1,880 1,900 1,920

GCTATTCTGTATACACCCGCAGAGTACTGCAATTTGACTGTATTACCAATGTCAGCAAATTTTCTGTCTTCGAAGAGTAAAAAATTGTACTTGGCGGATAATGCCTT
CGATAAGACATATGTGGGCGTCTCATGACGTTAAACTGACATAATGGTTACAGTCGTTTAAAAGACAGAAGCTTCTCATTTTTTAACATGAACCGCCTATTACGGAA
URA3
1,940 1,960 1,980 2,000 2,020

TAGCGGCTTAACTGTGCCCTCCATGGAAAAATCAGTCAAGATATCCACATGTGTTTTTAGTAAACAAATTTTGGGACCTAATGCTTCAACTAACTCCAGTAATTCCT
ATCGCCGAATTGACACGGGAGGTACCTTTTTAGTCAGTTCTATAGGTGTACACAAAAATCATTTGTTTAAAACCCTGGATTACGAAGTTGATTGAGGTCATTAAGGA
URA3
2,040 2,060 2,080 2,100 2,120 2,140

TGGTGGTACGAACATCCAATGAAGCACACAAGTTTGTTTGCTTTTCGTGCATGATATTAAATAGCTTGGCAGCAACAGGACTAGGATGAGTAGCAGCACGTTCCTTA
ACCACCATGCTTGTAGGTTACTTCGTGTGTTCAAACAAACGAAAAGCACGTACTATAATTTATCGAACCGTCGTTGTCCTGATCCTACTCATCGTCGTGCAAGGAAT
URA3
2,160 2,180 2,200 2,220 2,240

TATGTAGCTTTCGACATGATTTATCTTCGTTTCCTGCATGTTTTTGTTCTGTGCAGTTGGGTTAAGAATACTGGGCAATTTCATGTTTCTTCAACACTACATATGCG
ATACATCGAAAGCTGTACTAAATAGAAGCAAAGGACGTACAAAAACAAGACACGTCAACCCAATTCTTATGACCCGTTAAAGTACAAAGAAGTTGTGATGTATACGC
URA3
2,260 2,280 2,300 2,320 2,340

TATATATACCAATCTAAGTCTGTGCTCCTTCCTTCGTTCTTCCTTCTGTTCGGAGATTACCGAATCAAAAAAATTTCAAAGAAACCGAAATCAAAAAAAAGAATAAA
ATATATATGGTTAGATTCAGACACGAGGAAGGAAGCAAGAAGGAAGACAAGCCTCTAATGGCTTAGTTTTTTTAAAGTTTCTTTGGCTTTAGTTTTTTTTCTTATTT
URA3
2,360 2,380 2,400 2,420 2,440 2,460

AAAAAAATGATGAATTGAATTGAAAAGCTAATTCTTGAAGACGAAAGGGCCTCGTGATACGCCTATTTTTATAGGTTAATGTCATGATAATAATGGTTTCTTAGACG
TTTTTTTACTACTTAACTTAACTTTTCGATTAAGAACTTCTGCTTTCCCGGAGCACTATGCGGATAAAAATATCCAATTACAGTACTATTATTACCAAAGAATCTGC
URA3
2,480 2,500 2,520 2,540 2,560

TCAGGTGGCACTTTTCGGGGAAATGTGCGCGGAACCCCTATTTGTTTATTTTTCTAAATACATTCAAATATGTATCCGCTCATGAGACAATAACCCTGATAAATGCT
AGTCCACCGTGAAAAGCCCCTTTACACGCGCCTTGGGGATAAACAAATAAAAAGATTTATGTAAGTTTATACATAGGCGAGTACTCTGTTATTGGGACTATTTACGA
2,580 2,600 2,620 2,640 2,660

```
TCAATAATATTGAAAAAGGAAGAGTATGAGTATTCAACATTTCCGTGTCGCCCTTATTCCCTTTTTTGCGGCATTTTGCCTTCCTGTTTTTGCTCACCCAGAAACGC
AGTTATTATAACTTTTTCCTTCTCATACTCATAAGTTGTAAAGGCACAGCGGGAATAAGGGAAAAAACGCCGTAAAACGGAAGGACAAAAACGAGTGGGTCTTTGCG
   2,680               2,700               2,720               2,740               2,760               2,780

TGGTGAAAGTAAAAGATGCTGAAGATCAGTTGGGTGCACGAGTGGGTTACATCGAACTGGATCTCAACAGCGGTAAGATCCTTGAGAGTTTTCGCCCCGAAGAACGT
ACCACTTTCATTTTCTACGACTTCTAGTCAACCCACGTGCTCACCCAATGTAGCTTGACCTAGAGTTGTCGCCATTCTAGGAACTCTCAAAAGCGGGGCTTCTTGCA
                2,800               2,820               2,840               2,860               2,880

TTTCCAATGATGAGCACTTTTAAAGTTCTGCTATGTGGCGCGGTATTATCCCGTATTGACGCCGGGCAAGAGCAACTCGGTCGCCGCATACACTATTCTCAGAATGA
AAAGGTTACTACTCGTGAAAATTTCAAGACGATACACCGCGCCATAATAGGGCATAACTGCGGCCCGTTCTCGTTGAGCCAGCGGCGTATGTGATAAGAGTCTTACT
         AmpR
         2,900               2,920               2,940               2,960               2,980

CTTGGTTGAGTACTCACCAGTCACAGAAAAGCATCTTACGGATGGCATGACAGTAAGAGAATTATGCAGTGCTGCCATAACCATGAGTGATAACACTGCGGCCAACT
GAACCAACTCATGAGTGGTCAGTGTCTTTTCGTAGAATGCCTACCGTACTGTCATTCTCTTAATACGTCACGACGGTATTGGTACTCACTATTGTGACGCCGGTTGA
AmpR
3,000               3,020               3,040               3,060               3,080               3,100

TACTTCTGACAACGATCGGAGGACCGAAGGAGCTAACCGCTTTTTTGCACAACATGGGGGATCATGTAACTCGCCTTGATCGTTGGGAACCGGAGCTGAATGAAGCC
ATGAAGACTGTTGCTAGCCTCCTGGCTTCCTCGATTGGCGAAAAAACGTGTTGTACCCCCTAGTACATTGAGCGGAACTAGCAACCCTTGGCCTCGACTTACTTCGG
AmpR
             3,120               3,140               3,160               3,180               3,200

ATACCAAACGACGAGCGTGACACCACGATGCCTGTAGCAATGGCAACAACGTTGCGCAAACTATTAACTGGCGAACTACTTACTCTAGCTTCCCGGCAACAATTAAT
TATGGTTTGCTGCTCGCACTGTGGTGCTACGGACATCGTTACCGTTGTTGCAACGCGTTTGATAATTGACCGCTTGATGAATGAGATCGAAGGGCCGTTGTTAATTA
AmpR
      3,220               3,240               3,260               3,280               3,300

AGACTGGATGGAGGCGGATAAAGTTGCAGGACCACTTCTGCGCTCGGCCCTTCCGGCTGGCTGGTTTATTGCTGATAAATCTGGAGCCGGTGAGCGTGGGTCTCGCG
TCTGACCTACCTCCGCCTATTTCAACGTCCTGGTGAAGACGCGAGCCGGGAAGGCCGACCGACCAAATAACGACTATTTAGACCTCGGCCACTCGCACCCAGAGCGC
AmpR
3,320               3,340               3,360               3,380               3,400               3,420

GTATCATTGCAGCACTGGGGCCAGATGGTAAGCCCTCCCGTATCGTAGTTATCTACACGACGGGGAGTCAGGCAACTATGGATGAACGAAATAGACAGATCGCTGAG
CATAGTAACGTCGTGACCCCGGTCTACCATTCGGGAGGGCATAGCATCAATAGATGTGCTGCCCCTCAGTCCGTTGATACCTACTTGCTTTATCTGTCTAGCGACTC
AmpR
            3,440               3,460               3,480               3,500               3,520

ATAGGTGCCTCACTGATTAAGCATTGGTAACTGTCAGACCAAGTTTACTCATATATACTTTAGATTGATTTAAAACTTCATTTTTAATTTAAAAGGATCTAGGTGAA
TATCCACGGAGTGACTAATTCGTAACCATTGACAGTCTGGTTCAAATGAGTATATATGAAATCTAACTAAATTTTGAAGTAAAAATTAAATTTTCCTAGATCCACTT
AmpR
    3,540               3,560               3,580               3,600               3,620
```

GATCCTTTTTGATAATCTCATGACCAAAATCCCTTAACGTGAGTTTTCGTTCCACTGAGCGTCAGACCCCGTAGAAAAGATCAAAGGATCTTCTTGAGATCCTTTTT
CTAGGAAAAACTATTAGAGTACTGGTTTTAGGGAATTGCACTCAAAAGCAAGGTGACTCGCAGTCTGGGGCATCTTTTCTAGTTTCCTAGAAGAACTCTAGGAAAAA
ColE1 origin
3,640 3,660 3,680 3,700 3,720 3,740

TTCTGCGCGTAATCTGCTGCTTGCAAACAAAAAAACCACCGCTACCAGCGGTGGTTTGTTTGCCGGATCAAGAGCTACCAACTCTTTTTCCGAAGGTAACTGGCTTC
AAGACGCGCATTAGACGACGAACGTTTGTTTTTTTGGTGGCGATGGTCGCCACCAAACAAACGGCCTAGTTCTCGATGGTTGAGAAAAAGGCTTCCATTGACCGAAG
ColE1 origin
3,760 3,780 3,800 3,820 3,840

AGCAGAGCGCAGATACCAAATACTGTCCTTCTAGTGTAGCCGTAGTTAGGCCACCACTTCAAGAACTCTGTAGCACCGCCTACATACCTCGCTCTGCTAATCCTGTT
TCGTCTCGCGTCTATGGTTTATGACAGGAAGATCACATCGGCATCAATCCGGTGGTGAAGTTCTTGAGACATCGTGGCGGATGTATGGAGCGAGACGATTAGGACAA
ColE1 origin
3,860 3,880 3,900 3,920 3,940

ACCAGTGGCTGCTGCCAGTGGCGATAAGTCGTGTCTTACCGGGTTGGACTCAAGACGATAGTTACCGGATAAGGCGCAGCGGTCGGGCTGAACGGGGGGTTCGTGCA
TGGTCACCGACGACGGTCACCGCTATTCAGCACAGAATGGCCCAACCTGAGTTCTGCTATCAATGGCCTATTCCGCGTCGCCAGCCCGACTTGCCCCCCAAGCACGT
ColE1 origin
3,960 3,980 4,000 4,020 4,040 4,060

CACAGCCCAGCTTGGAGCGAACGACCTACACCGAACTGAGATACCTACAGCGTGAGCTATGAGAAAGCGCCACGCTTCCCGAAGGGAGAAAGGCGGACAGGTATCCG
GTGTCGGGTCGAACCTCGCTTGCTGGATGTGGCTTGACTCTATGGATGTCGCACTCGATACTCTTTCGCGGTGCGAAGGGCTTCCCTCTTTCCGCCTGTCCATAGGC
ColE1 origin
4,080 4,100 4,120 4,140 4,160

GTAAGCGGCAGGGTCGGAACAGGAGAGCGCACGAGGGAGCTTCCAGGGGGAAACGCCTGGTATCTTTATAGTCCTGTCGGGTTTCGCCACCTCTGACTTGAGCGTCG
CATTCGCCGTCCCAGCCTTGTCCTCTCGCGTGCTCCCTCGAAGGTCCCCCTTTGCGGACCATAGAAATATCAGGACAGCCCAAAGCGGTGGAGACTGAACTCGCAGC
ColE1 origin
4,180 4,200 4,220 4,240 4,260 4,280

ATTTTTGTGATGCTCGTCAGGGGGGCGGAGCCTATGGAAAAACGCCAGCAACGCGGCCTTTTTACGGTTCCTGGCCTTTTGCTGGCCTTTTGCTCACATGTTCTTTC
TAAAAACACTACGAGCAGTCCCCCCGCCTCGGATACCTTTTTGCGGTCGTTGCGCCGGAAAAATGCCAAGGACCGGAAAACGACCGGAAAACGAGTGTACAAGAAAG
ColE1 origin
4,300 4,320 4,340 4,360 4,380

CTGCGTTATCCCCTGATTCTGTGGATAACCGTATTACCGCCTTTGAGTGAGCTGATACCGCTCGCCGCAGCCGAACGACCGAGCGCAGCGAGTCAGTGAGCGAGGAA
GACGCAATAGGGGACTAAGACACCTATTGGCATAATGGCGGAAACTCACTCGACTATGGCGAGCGGCGTCGGCTTGCTGGCTCGCGTCGCTCAGTCACTCGCTCCTT
4,400 4,420 4,440 4,460 4,480

GCGGAAGAGCGCCCAATACGCAAACCGCCTCTCCCCGCGCGTTGGCCGATTCATTAATGCAGCTGGCACGACAGGTTTCCCGACTGGAAAGCGGGCAGTGAGCGCAA
CGCCTTCTCGCGGGTTATGCGTTTGGCGGAGAGGGGCGCGCAACCGGCTAAGTAATTACGTCGACCGTGCTGTCCAAAGGGCTGACCTTTCGCCCGTCACTCGCGTT
4,500 4,520 4,540 4,560 4,580 4,600

CGCAATTAATGTGAGTTAGCTCACTCATTAGGCACCCCAGGCTTTACACTTTATGCTTCCGGCTCGTATGTTGTGTGGAATTGTGAGCGGATAACAATTTCACACAG
GCGTTAATTACACTCAATCGAGTGAGTAATCCGTGGGGTCCGAAATGTGAAATACGAAGGCCGAGCATACAACACACCTTAACACTCGCCTATTGTTAAAGTGTGTC
lac promoter LacO
4,620 4,640 4,660 4,680 4,700

GAAACAGCTATGACCATGATTACGCCAAGCTTACCAGTTCTCACACGGAACACCACTAATGGACACAAATTCGAAATACTTTGACCCTATTTTCGAGGACCTTGTCA
CTTTGTCGATACTGGTACTAATGCGGTTCGAATGGTCAAGAGTGTGCCTTGTGGTGATTACCTGTGTTTAAGCTTTATGAAACTGGGATAAAAGCTCCTGGAACAGT
M13-rev GAPDH promoter
4,720 4,740 4,760 4,780 4,800

CCTTGAGCCCAAGAGAGCCAAGATTTAAATTTTCCTATGACTTGATGCAAATTCCCAAAGCTAATAACATGCAAGACACGTACGGTCAAGAAGACATATTTGACCTC
GGAACTCGGGTTCTCTCGGTTCTAAATTTAAAAGGATACTGAACTACGTTTAAGGGTTTCGATTATTGTACGTTCTGTGCATGCCAGTTCTTCTGTATAAACTGGAG
GAPDH promoter
4,820 4,840 4,860 4,880 4,900 4,920

TTAACAGGTTCAGACGCGACTGCCTCATCAGTAAGACCCGTTGAAAAGAACTTACCTGAAAAAAACGAATATATACTAGCGTTGAATGTTAGCGTCAACAACAAGAA
AATTGTCCAAGTCTGCGCTGACGGAGTAGTCATTCTGGGCAACTTTTCTTGAATGGACTTTTTTTGCTTATATATGATCGCAACTTACAATCGCAGTTGTTGTTCTT
GAPDH promoter
4,940 4,960 4,980 5,000 5,020

GTTTAATGACGCGGAGGCCAAGGCAAAAAGATTCCTTGATTACGTAAGGGAGTTAGAATCATTTTGAATAAAAAACACGCTTTTTCAGTTCGAGTTTATCATTATCA
CAAATTACTGCGCCTCCGGTTCCGTTTTTCTAAGGAACTAATGCATTCCCTCAATCTTAGTAAAACTTATTTTTTGTGCGAAAAAGTCAAGCTCAAATAGTAATAGT
GAPDH promoter
5,040 5,060 5,080 5,100 5,120

ATACTGCCATTTCAAAGAATACGTAAATAATTAATAGTAGTGATTTTCCTAACTTTATTTAGTCAAAAAATTAGCCTTTTAATTCTGCTGTAACCCGTACATGCCCA
TATGACGGTAAAGTTTCTTATGCATTTATTAATTATCATCACTAAAAGGATTGAAATAAATCAGTTTTTTAATCGGAAAATTAAGACGACATTGGGCATGTACGGGT
GAPDH promoter
5,140 5,160 5,180 5,200 5,220 5,240

AAATAGGGGGCGGGTTACACAGAATATATAACATCGTAGGTGTCTGGGTGAACAGTTTATTCCTGGCATCCACTAAATATAATGGAGCCCGCTTTTTAAGCTGGCAT
TTTATCCCCCGCCCAATGTGTCTTATATATTGTAGCATCCACAGACCCACTTGTCAAATAAGGACCGTAGGTGATTTATATTACCTCGGGCGAAAAATTCGACCGTA
GAPDH promoter
5,260 5,280 5,300 5,320 5,340

CCAGAAAAAAAAAGAATCCCAGCACCAAAATATTGTTTTCTTCACCAACCATCAGTTCATAGGTCCATTCTCTTAGCGCAACTACAGAGAACAGGGGCACAAACAGG
GGTCTTTTTTTTTCTTAGGGTCGTGGTTTTATAACAAAAGAAGTGGTTGGTAGTCAAGTATCCAGGTAAGAGAATCGCGTTGATGTCTCTTGTCCCCGTGTTTGTCC
GAPDH promoter
5,360 5,380 5,400 5,420 5,440

CAAAAAACGGGCACAACCTCAATGGAGTGATGCAACCTGCCTGGAGTAAATGATGACACAAGGCAATTGACCCACGCATGTATCTATCTCATTTTCTTACACCTTCT
GTTTTTTGCCCGTGTTGGAGTTACCTCACTACGTTGGACGGACCTCATTTACTACTGTGTTCCGTTAACTGGGTGCGTACATAGATAGAGTAAAAGAATGTGGAAGA

GAPDH promoter

5,460 5,480 5,500 5,520 5,540 5,560

ATTACCTTCTGCTCTCTCTGATTTGGAAAAAGCTGAAAAAAAAGGTTGAAACCAGTTCCCTGAAATTATTCCCCTACTTGACTAATAAGTATATAAAGACGGTAGGT
TAATGGAAGACGAGAGAGACTAAACCTTTTTCGACTTTTTTTTCCAACTTTGGTCAAGGGACTTTAATAAGGGGATGAACTGATTATTCATATATTTCTGCCATCCA

GAPDH promoter

5,580 5,600 5,620 5,640 5,660

ATTGATTGTAATTCTGTAAATCTATTTCTTAAACTTCTTAAATTCTACTTTTATAGTTAGTCTTTTTTTTAGTTTTAAAACACCAAGAACTTAGTTTCGAATAAACA
TAACTAACATTAAGACATTTAGATAAAGAATTTGAAGAATTTAAGATGAAAATATCAATCAGAAAAAAAATCAAAATTTTGTGGTTCTTGAATCAAAGCTTATTTGT

GAPDH promoter

5,680 5,700 5,720 5,740 5,760

CACATAAACAGAATTCATGAGATTCCCATCTATCTTCACCGCTGTTTTGTTCGCTGCTTCTTCTGCTTTGGCTGCTCCAGCTAACACCACCACCGAAGACGAAACCG
GTGTATTTGTCTTAAGTACTCTAAGGGTAGATAGAAGTGGCGACAAAACAAGCGACGAAGAAGACGAAACCGACGAGGTCGATTGTGGTGGTGGCTTCTGCTTTGGC

1 2 3 4 5 6 7 8 9 10 11 12 13 14 15 16 17 18 19 20 21 22 23 24 25 26 27 28 29 30
M R F P S I F T A V L F A A S S A L A A P A N T T T E D E T

Mating factor alpha leader

5,780 5,800 5,820 5,840 5,860 5,880

CTCAAATCCCAGCTGAAGCTGTTATCGACTACTCTGACTTGGAAGGTGACTTCGACGCTGCTGCTTTGCCATTGTCTAACTCTACCAACAACGGTTTGTCTTCTACC
GAGTTTAGGGTCGACTTCGACAATAGCTGATGAGACTGAACCTTCCACTGAAGCTGCGACGACGAAACGGTAACAGATTGAGATGGTTGTTGCCAAACAGAAGATGG

31 32 33 34 35 36 37 38 39 40 41 42 43 44 45 46 47 48 49 50 51 52 53 54 55 56 57 58 59 60 61 62 63 64 65 66
A Q I P A E A V I D Y S D L E G D F D A A A L P L S N S T N N G L S S T

Mating factor alpha leader

5,900 5,920 5,940 5,960 5,980

AACACCACCATCGCTTCTATCGCTGCTAAGGAAGAAGGTGTTCAATTGGACAAGAGAGAAagatctggcggccgcgcatgcgctagcctcgagGATGTGCAGTTGCA
TTGTGGTGGTAGCGAAGATAGCGACGATTCCTTCTTCCACAAGTTAACCTGTTCTCTCTTtctagaccgccggcgcgtacgcgatcggagctcCTACACGTCAACGT

67 68 69 70 71 72 73 74 75 76 77 78 79 80 81 82 83 84 85 86
N T T I A S I A A K E E G V Q L D K R E

2 4
D V Q L Q

Mating factor alpha leader

6,000 6,020 6,040 6,060 6,080

AGCATCCGGCGGCGGTTCTGTTCAGGCTGGTGGTTCTCTAAGATTAAGTTGTGCTGCTTCAGGTTATACCATCGGCCCATACTGCATGGGTTGGTTTCGTCAAGCTC
TCGTAGGCCGCCGCCAAGACAAGTCCGACCACCAAGAGATTCTAATTCAACACGACGAAGTCCAATATGGTAGCCGGGTATGACGTACCCAACCAAAGCAGTTCGAG

6 8 10 12 14 16 18 20 22 24 26 28 30 32 34 36 38 40
A S G G G S V Q A G G S L R L S C A A S G Y T I G P Y C M G W F R Q A

anti-lys Nb

6,100 6,120 6,140 6,160 6,180 6,200

CAGGCAAAGAAAGGGAGGGAGTCGCAGCCATTAACATGGGCGGCGGTATCACATATTATGCAGACTCCGTAAAAGGTAGATTTACTATCTCTCAGGACAATGCTAAG
GTCCGTTTCTTTCCCTCCCTCAGCGTCGGTAATTGTACCCGCCGCCATAGTGTATAATACGTCTGAGGCATTTTCCATCTAAATGATAGAGAGTCCTGTTACGATTC
42 44 46 48 50 52 54 56 58 60 62 64 66 68 70 72 74 76
P G K E R E G V A A I N M G G G I T Y Y A D S V K G R F T I S Q D N A K

anti-lys Nb

6,220 6,240 6,260 6,280 6,300

AACACGGTCTATCTGTTAATGAATTCATTGGAGCCCGAGGACACTGCCATATACTACTGTGCCGCAGATTCAACCATTTACGCTTCCTATTACGAATGCGGACACGG
TTGTGCCAGATAGACAATTACTTAAGTAACCTCGGGCTCCTGTGACGGTATATGATGACACGGCGTCTAAGTTGGTAAATGCGAAGGATAATGCTTACGCCTGTGCC
78 80 82 84 86 88 90 92 94 96 98 100 102 104 106 108 110 112
N T V Y L L M N S L E P E D T A I Y Y C A A D S T I Y A S Y Y E C G H G

anti-lys Nb

6,320 6,340 6,360 6,380 6,400 6,420

ATTAAGTACTGGAGGTTACGGTTATGATTCCTGGGGTCAAGGCACACAAGTCACGGTATCATCAgaatccTACCCATACGATGTTCCAGATTACGCTGCTGGAGGTT
TAATTCATGACCTCCAATGCCAATACTAAGGACCCCAGTTCCGTGTGTTCAGTGCCATAGTAGTcttaggATGGGTATGCTACAAGGTCTAATGCGACGACCTCCAA
114 116 118 120 122 124 126 128 130 132
L S T G G Y G Y D S W G Q G T Q V T V S S

anti-lys Nb HA tag

6,440 6,460 6,480 6,500 6,520

CGAGCGCCAAAAGCTCTTTTATCTCAACCACTACTACTGATTTAACAAGTATAAACACTAGTGCGTATTCCACTGGATCCATTTCCACAGTAGAAACAGGCAATCGA
GCTCGCGGTTTTCGAGAAAATAGAGTTGGTGATGATGACTAAATTGTTCATATTTGTGATCACGCATAAGGTGACCTAGGTAAAGGTGTCATCTTTGTCCGTTAGCT
2 4 6 8 10 12 14 16 18 20 22 24 26 28 30 32 34
S A K S S F I S T T T T D L T S I N T S A Y S T G S I S T V E T G N R

3' Half of α-agglutinin

6,540 6,560 6,580 6,600 6,620

ACTACATCAGAAGTGATCAGTCATGTGGTGACTACCAGCACAAAACTGTCTCCAACTGCTACTACCAGCCTGACAATTGCACAAACCAGTATCTATTCTACTGACTC
TGATGTAGTCTTCACTAGTCAGTACACCACTGATGGTCGTGTTTTGACAGAGGTTGACGATGATGGTCGGACTGTTAACGTGTTTGGTCATAGATAAGATGACTGAG
36 38 40 42 44 46 48 50 52 54 56 58 60 62 64 66 68 70
T T S E V I S H V V T T S T K L S P T A T T S L T I A Q T S I Y S T D S

3' Half of α-agglutinin

6,640 6,660 6,680 6,700 6,720 6,740

ATATATCACAGTAGGAACAGATATTCACACCACATCAGAAGTGATTAGTGATGTGGAAACCATTAGCAGAGAAACAGCTTCGACCGTTGTAGCCGCTCCAACCTCAA
TATATAGTGTCATCCTTGTCTATAAGTGTGGTGTAGTCTTCACTAATCACTACACCTTTGGTAATCGTCTCTTTGTCGAAGCTGGCAACATCGGCGAGGTTGGAGTT
72 74 76 78 80 82 84 86 88 90 92 94 96 98 100 102 104 106
Y I T V G T D I H T T S E V I S D V E T I S R E T A S T V V A A P T S

3' Half of α-agglutinin

6,760 6,780 6,800 6,820 6,840

```
CAACTGGATGGACAGGCGCTATGAATACTTACATCTCGCAATTTACATCCTCTTCTTTCGCAACAATCAACAGCACACCAATAATCTCTTCATCAGCAGTATTTGAA
GTTGACCTACCTGTCCGCGATACTTATGAATGTAGAGCGTTAAATGTAGGAGAAGAAAGCGTTGTTAGTTGTCGTGTGGTTATTAGAGAAGTAGTCGTCATAAACTT
  108   110   112   114   116   118   120   122   124   126   128   130   132   134   136   138   140   142
 T  T  G  W  T  G  A  M  N  T  Y  I  S  Q  F  T  S  S  S  F  A  T  I  N  S  T  P  I  I  S  S  S  A  V  F  E
3' Half of α-agglutinin
       6,860       6,880       6,900       6,920       6,940
```

```
ACCTCAGATGCTTCAATTGTCAATGTGCACACTGAAAATATCACGAATACTGCTGCTGTTCCATCTGAAGAGCCCACTTTTGTAAATGCCACGAGAAACTCCTTAAA
TGGAGTCTACGAAGTTAACAGTTACACGTGTGACTTTTATAGTGCTTATGACGACGACAAGGTAGACTTCTCGGGTGAAAACATTTACGGTGCTCTTTGAGGAATTT
   144   146   148   150   152   154   156   158   160   162   164   166   168   170   172   174   176   178
 T  S  D  A  S  I  V  N  V  H  T  E  N  I  T  N  T  A  A  V  P  S  E  E  P  T  F  V  N  A  T  R  N  S  L  N
3' Half of α-agglutinin
   6,960       6,980       7,000       7,020       7,040       7,060
```

```
TTCCTTCTGCAGCAGCAAACAGCCATCCAGTCCCTCATCTTATACGTCTTCCCCACTCGTATCGTCCCTCTCCGTAAGCAAAACATTACTAAGCACCAGTTTTACGC
AAGGAAGACGTCGTCGTTTGTCGGTAGGTCAGGGAGTAGAATATGCAGAAGGGGTGAGCATAGCAGGGAGAGGCATTCGTTTTGTAATGATTCGTGGTCAAAATGCG
    180   182   184   186   188   190   192   194   196   198   200   202   204   206   208   210   212
  S  F  C  S  S  K  Q  P  S  S  P  S  S  Y  T  S  S  P  L  V  S  S  L  S  V  S  K  T  L  L  S  T  S  F  T
3' Half of α-agglutinin
          7,080       7,100       7,120       7,140       7,160
```

```
CTTCTGTGCCAACATCTAATACATATATCAAAACGAAAAATACGGGTTACTTTGAGCACACGGCTTTGACAACATCTTCAGTTGGCCTTAATTCTTTTAGTGAAACA
GAAGACACGGTTGTAGATTATGTATATAGTTTTGCTTTTTATGCCCAATGAAACTCGTGTGCCGAAACTGTTGTAGAAGTCAACCGGAATTAAGAAAATCACTTTGT
214   216   218   220   222   224   226   228   230   232   234   236   238   240   242   244   246   248
 P S  V  P  T  S  N  T  Y  I  K  T  K  N  T  G  Y  F  E  H  T  A  L  T  T  S  S  V  G  L  N  S  F  S  E  T
3' Half of α-agglutinin
        7,180       7,200       7,220       7,240       7,260
```

```
GCAGTCTCATCTCAGGGAACGAAAATTGACACCTTTTTAGTGTCATCCTTGATCGCATATCCTTCTTCTGCATCAGGAAGCCAATTGTCCGGTATCCAACAGAATTT
CGTCAGAGTAGAGTCCCTTGCTTTTAACTGTGGAAAAATCACAGTAGGAACTAGCGTATAGGAAGAAGACGTAGTCCTTCGGTTAACAGGCCATAGGTTGTCTTAAA
250   252   254   256   258   260   262   264   266   268   270   272   274   276   278   280   282   284
 A  V  S  S  Q  G  T  K  I  D  T  F  L  V  S  S  L  I  A  Y  P  S  S  A  S  G  S  Q  L  S  G  I  Q  Q  N  F
3' Half of α-agglutinin
 7,280       7,300       7,320       7,340       7,360       7,380
```

```
CACATCAACTTCTCTCATGATTTCAACCTATGAAGGTAAAGCGTCTATATTTTTCTCAGCTGAGCTCGGTTCGATCATTTTTCTGCTTTTGTCGTACCTGCTATTCT
GTGTAGTTGAAGAGAGTACTAAAGTTGGATACTTCCATTTCGCAGATATAAAAAGAGTCGACTCGAGCCAAGCTAGTAAAAAGACGAAAACAGCATGGACGATAAGA
 286   288   290   292   294   296   298   300   302   304   306   308   310   312   314   316   318   320
  T  S  T  S  L  M  I  S  T  Y  E  G  K  A  S  I  F  F  S  A  E  L  G  S  I  I  F  L  L  L  S  Y  L  L  F
3' Half of α-agglutinin
            7,400       7,420       7,440       7,460       7,480
```

AAAACGGGTACTGTACAGTTAGTACATTGAGTCGAAATATACGAAATTATTGTTCATAATTTTCATCCTGGCTCTTTTTTTCTTCAACCATAGTTAAATGGACAGTT
TTTTGCCCATGACATGTCAATCATGTAACTCAGCTTTATATGCTTTAATAACAAGTATTAAAAGTAGGACCGAGAAAAAAAGAAGTTGGTATCAATTTACCTGTCAA

*

3′ Half of α-agglutinin

7,500 7,520 7,540 7,560 7,580

CATATCTTAACTCTAATAATACTTTTCTAGTTCTTATCCTTTTCCGTCTCACCGCAGATTTTATCATAGTATTAAATTTATATTTTGTTCGTAAAAAGAAAAATTTG
GTATAGAATTGAGATTATTATGAAAAGATCAAGAATAGGAAAAGGCAGAGTGGCGTCTAAAATAGTATCATAATTTAAATATAAAACAAGCATTTTTCTTTTTAAAC

3′ Half of α-agglutinin

7,600 7,620 7,640 7,660 7,680 7,700

TGAGCGTTACCGCTCGTTTCATTACCCGAAGGCTGTTTCAGTAGACCACTGATTAAGTAAGTAGATGAAAAAATTTCATCACCATGAAAGAGTTCGATGAGAGCTAC
ACTCGCAATGGCGAGCAAAGTAATGGGCTTCCGACAAAGTCATCTGGTGACTAATTCATTCATCTACTTTTTTAAAGTAGTGGTACTTTCTCAAGCTACTCTCGATG

3′ Half of α-agglutinin

7,720 7,740 7,760 7,780 7,800

TTTTTCAAATGCTTAACAGCTAACCGCCATTCAATAATGTTACGCTCTCTTCATTCTGCGGCTACGTTATCTAACAAGAGGTTTTACTCTCTCATATCTCATTCAAA
AAAAAGTTTACGAATTGTCGATTGGCGGTAAGTTATTACAATGCGAGAGAAGTAAGACGCCGATGCAATAGATTGTTCTCCAAAATGAGAGAGTATAGAGTAAGTTT

3′ Half of α-agglutinin

7,820 7,840 7,860 7,880 7,900

TAGAAAGAACATAATCAAAGGTACCGCGATGTAGTAAAACTAGCTAGACCGAGAAAGAGACTAGAAATGCAAAAGGCACTTCTACAATGGCTGCCATCATTATTATC
ATCTTTCTTGTATTAGTTTCCATGGCGCTACATCATTTTGATCGATCTGGCTCTTTCTCTGATCTTTACGTTTTCCGTGAAGATGTTACCGACGGTAGTAATAATAG

7,920 7,940 7,960 7,980 8,000 8,020

CGATGTGACGCTGCATTTTTTTTTTTTTTTTTTTTTTTTTTTTTTTTTTTTTTTTTTTTTTTTTTTTGTACAAATATCATAAAAAAAGAGAATCTTTTTAAGCAAGGATTTT
GCTACACTGCGACGTAAAAAAAAAAAAAAAAAAAAAAAAAAAAAAAAAAAAAAAAAAAAAAAAAAAACATGTTTATAGTATTTTTTTCTCTTAGAAAAATTCGTTCCTAAAA

8,040 8,060 8,080 8,100 8,120

CTTAACTTCTTCGGCGACAGCATCACCGACTTCGGTGGTACTGTTGGAACCACCTAAATCACCAGTTCTGATACCTGCATCCAAAACCTTTTTAACTGCATCTTCAA
GAATTGAAGAAGCCGCTGTCGTAGTGGCTGAAGCCACCATGACAACCTTGGTGGATTTAGTGGTCAAGACTATGGACGTAGGTTTTGGAAAAATTGACGTAGAAGTT

8,140 8,160 8,180 8,200 8,220

TGGCCTTACCTTCTTCAGGCAAGTTCAATGACAATTTCAACATCATTGCAGCAGACAAGATAGTGGCGATAGGGTCAACCTTATTCTTTGGCAAATCTGGAGCAGAA
ACCGGAATGGAAGAAGTCCGTTCAAGTTACTGTTAAAGTTGTAGTAACGTCGTCTGTTCTATCACCGCTATCCCAGTTGGAATAAGAAACCGTTTAGACCTCGTCTT

8,240 8,260 8,280 8,300 8,320 8,340

CCGTGGCATGGTTCGTACAAACCAAATGCGGTGTTCTTGTCTGGCAAAGAGGCCAAGGACGCAGATGGCAACAAACCCAAGGAACCTGGGATAACGGAGGCTTCATC
GGCACCGTACCAAGCATGTTTGGTTTACGCCACAAGAACAGACCGTTTCTCCGGTTCCTGCGTCTACCGTTGTTTGGGTTCCTTGGACCCTATTGCCTCCGAAGTAG

8,360 8,380 8,400 8,420 8,440

GGAGATGATATCACCAAACATGTTGCTGGTGATTATAATACCATTTAGGTGGGTTGGGTTCTTAACTAGGATCATGGCGGCAGAATCAATCAATTGATGTTGAACCT
CCTCTACTATAGTGGTTTGTACAACGACCACTAATATTATGGTAAATCCACCCAACCCAAGAATTGATCCTAGTACCGCCGTCTTAGTTAGTTAACTACAACTTGGA

8,460 8,480 8,500 8,520 8,540 8,560

TCAATGTAGGAAATTCGTTCTTGATGGTTTCCTCCACAGTTTTTCTCCATAATCTTGAAGAGGCCAAAACATTAGCTTTATCCAAGGACCAAATAGGCAATGGTGGC
AGTTACATCCTTTAAGCAAGAACTACCAAAGGAGGTGTCAAAAAGAGGTATTAGAACTTCTCCGGTTTTGTAATCGAAATAGGTTCCTGGTTTATCCGTTACCACCG

8,580 8,600 8,620 8,640 8,660

TCATGTTGTAGGGCCATGAAAGCGGCCATTCTTGTGATTCTTTGCACTTCTGGAACGGTGTATTGTTCACTATCCCAAGCGACACCATCACCATCGTCTTCCTTTCT
AGTACAACATCCCGGTACTTTCGCCGGTAAGAACACTAAGAAACGTGAAGACCTTGCCACATAACAAGTGATAGGGTTCGCTGTGGTAGTGGTAGCAGAAGGAAAGA

8,680 8,700 8,720 8,740 8,760

CTTACCAAAGTAAATACCTCCCACTAATTCTCTGACAACAACGAAGTCAGTACCTTTAGCAAATTGTGGCTTGATTGGAGATAAGTCTAAAAGAGAGTCGGATGCAA
GAATGGTTTCATTTATGGAGGGTGATTAAGAGACTGTTGTTGCTTCAGTCATGGAAATCGTTTAACACCGAACTAACCTCTATTCAGATTTTCTCTCAGCCTACGTT

8,780 8,800 8,820 8,840 8,860 8,880

AGTTACATGGTCTTAAGTTGGCGTACAATTGAAGTTCTTTACGGATTTTTAGTAAACCTTGTTCAGGTCTAACACTACCTGTACCCCATTTAGGACCACCCACAGCA
TCAATGTACCAGAATTCAACCGCATGTTAACTTCAAGAAATGCCTAAAAATCATTTGGAACAAGTCCAGATTGTGATGGACATGGGGTAAATCCTGGTGGGTGTCGT

8,900 8,920 8,940 8,960 8,980

CCTAACAAAACGGCATCAACCTTCTTGGAGGCTTCCAGCGCCTCATCTGGAAGTGGGACACCTGTAGCATCGATAGCAGCACCACCAATTAAATGATTTTCGAAATC
GGATTGTTTTGCCGTAGTTGGAAGAACCTCCGAAGGTCGCGGAGTAGACCTTCACCCTGTGGACATCGTAGCTATCGTCGTGGTGGTTAATTTACTAAAAGCTTTAG

9,000 9,020 9,040 9,060 9,080

GAACTTGACATTGGAACGAACATCAGAAATAGCTTTAAGAACCTTAATGGCTTCGGCTGTGATTTCTTGACCAACGTGGTCACCTGGCAAAACGACGATCTTCTTAG
CTTGAACTGTAACCTTGCTTGTAGTCTTTATCGAAATTCTTGGAATTACCGAAGCCGACACTAAAGAACTGGTTGCACCAGTGGACCGTTTTGCTGCTAGAAGAATC

9,100 9,120 9,140 9,160 9,180 9,200

GGGCAGACATTACAATGGTATATCCTTGAAATATATATAAAAAAAAAAAAAAAAAAAAAAAAAAAAAAAAATGCAGCTTCTCAATGATATTCGAATACGCTTTGAGGAG
CCCGTCTGTAATGTTACCATATAGGAACTTTATATATATTTTTTTTTTTTTTTTTTTTTTTTTTTTTTTTTACGTCGAAGAGTTACTATAAGCTTATGCGAAACTCCTC

9,220 9,240 9,260 9,280 9,300

ATACAGCCTAATATCCGACAAACTGTTTTACAGATTTACGATCGTACTTGTTACCCATCATTGAATTTTGAACATCCGAACCTGGGAGTTTTCCCTGAAACAGATAG
TATGTCGGATTATAGGCTGTTTGACAAAATGTCTAAATGCTAGCATGAACAATGGGTAGTAACTTAAAACTTGTAGGCTTGGACCCTCAAAAGGGACTTTGTCTATC

9,320 9,340 9,360 9,380 9,400

TATATTTGAACCTGTATAATAATATATAGTCTAGCGCTTTACGGAAGACAATGTATGTATTTCGGTTCCTGGAGAAACTATTGCATCTATTGCATAGGTAATCTTGC
ATATAAACTTGGACATATTATTATATATCAGATCGCGAAATGCCTTCTGTTACATACATAAAGCCAAGGACCTCTTTGATAACGTAGATAACGTATCCATTAGAACG

9,420 9,440 9,460 9,480 9,500 9,520

ACGTCGCATCCCCGGTTCATTTTCTGCGTTTCCATCTTGCACTTCAATAGCATATCTTT
TGCAGCGTAGGGGCCAAGTAAAAGACGCAAAGGTAGAACGTGAAGTTATCGTATAGAAA

9,530 9,540 9,550 9,560 9,570 9,580

System 2 control strain (9183 bp)

GTTAACGAAGCATCTGTGCTTCATTTTGTAGAACAAAAATGCAACGCGAGAGCGCTAATTTTTCAAACAAAGAATCTGAGCTGCATTTTTACAGAACAGAAATGCAA
CAATTGCTTCGTAGACACGAAGTAAAACATCTTGTTTTTACGTTGCGCTCTCGCGATTAAAAAGTTTGTTTCTTAGACTCGACGTAAAAATGTCTTGTCTTTACGTT
2 micron origin
20 40 60 80 100

CGCGAAAGCGCTATTTTACCAACGAAGAATCTGTGCTTCATTTTTGTAAAACAAAAATGCAACGCGAGAGCGCTAATTTTTCAAACAAAGAATCTGAGCTGCATTTT
GCGCTTTCGCGATAAAATGGTTGCTTCTTAGACACGAAGTAAAAACATTTTGTTTTTACGTTGCGCTCTCGCGATTAAAAAGTTTGTTTCTTAGACTCGACGTAAAA
2 micron origin
120 140 160 180 200

TACAGAACAGAAATGCAACGCGAGAGCGCTATTTTACCAACAAAGAATCTATACTTCTTTTTTGTTCTACAAAAATGCATCCCGAGAGCGCTATTTTTCTAACAAAG
ATGTCTTGTCTTTACGTTGCGCTCTCGCGATAAAATGGTTGTTTCTTAGATATGAAGAAAAAACAAGATGTTTTTACGTAGGGCTCTCGCGATAAAAAGATTGTTTC
2 micron origin
220 240 260 280 300 320

CATCTTAGATTACTTTTTTTCTCCTTTGTGCGCTCTATAATGCAGTCTCTTGATAACTTTTTGCACTGTAGGTCCGTTAAGGTTAGAAGAAGGCTACTTTGGTGTCT
GTAGAATCTAATGAAAAAAAGAGGAAACACGCGAGATATTACGTCAGAGAACTATTGAAAAACGTGACATCCAGGCAATTCCAATCTTCTTCCGATGAAACCACAGA
2 micron origin
340 360 380 400 420

ATTTTCTCTTCCATAAAAAAAGCCTGACTCCACTTCCCGCGTTTACTGATTACTAGCGAAGCTGCGGGTGCATTTTTTCAAGATAAAGGCATCCCCGATTATATTCT
TAAAAGAGAAGGTATTTTTTTCGGACTGAGGTGAAGGGCGCAAATGACTAATGATCGCTTCGACGCCCACGTAAAAAAGTTCTATTTCCGTAGGGGCTAATATAAGA
2 micron origin
440 460 480 500 520

ATACCGATGTGGATTGCGCATACTTTGTGAACAGAAAGTGATAGCGTTGATGATTCTTCATTGGTCAGAAAATTATGAACGGTTTCTTCTATTTTGTCTCTATATAC
TATGGCTACACCTAACGCGTATGAAACACTTGTCTTTCACTATCGCAACTACTAAGAAGTAACCAGTCTTTTAATACTTGCCAAAGAAGATAAAACAGAGATATATG
2 micron origin
540 560 580 600 620 640

TACGTATAGGAAATGTTTACATTTTCGTATTGTTTTCGATTCACTCTATGAATAGTTCTTACTACAATTTTTTTGTCTAAAGAGTAATACTAGAGATAAACATAAAA
ATGCATATCCTTTACAAATGTAAAAGCATAACAAAAGCTAAGTGAGATACTTATCAAGAATGATGTTAAAAAAACAGATTTCTCATTATGATCTCTATTTGTATTTT
2 micron origin
660 680 700 720 740

AATGTAGAGGTCGAGTTTAGATGCAAGTTCAAGGAGCGAAAGGTGGATGGGTAGGTTATATAGGGATATAGCACAGAGATATATAGCAAAGAGATACTTTTGAGCAA
TTACATCTCCAGCTCAAATCTACGTTCAAGTTCCTCGCTTTCCACCTACCCATCCAATATATCCCTATATCGTGTCTCTATATATCGTTTCTCTATGAAAACTCGTT
2 micron origin
760 780 800 820 840

TGTTTGTGGAAGCGGTATTCGCAATATTTTAGTAGCTCGTTACAGTCCGGTGCGTTTTTGGTTTTTTGAAAGTGCGTCTTCAGAGCGCTTTTGGTTTTCAAAAGCGC
ACAAACACCTTCGCCATAAGCGTTATAAAATCATCGAGCAATGTCAGGCCACGCAAAAACCAAAAAACTTTCACGCAGAAGTCTCGCGAAAACCAAAAGTTTTCGCG

2 micron origin

860 880 900 920 940 960

TCTGAAGTTCCTATACTTTCTAGCTAGAGAATAGGAACTTCGGAATAGGAACTTCAAAGCGTTTCCGAAAACGAGCGCTTCCGAAAATGCAACGCGAGCTGCGCACA
AGACTTCAAGGATATGAAAGATCGATCTCTTATCCTTGAAGCCTTATCCTTGAAGTTTCGCAAAGGCTTTTGCTCGCGAAGGCTTTTACGTTGCGCTCGACGCGTGT

2 micron origin

980 1,000 1,020 1,040 1,060

TACAGCTCACTGTTCACGTCGCACCTATATCTGCGTGTTGCCTGTATATATATATACATGAGAAGAACGGCATAGTGCGTGTTTATGCTTAAATGCGTACTTATATG
ATGTCGAGTGACAAGTGCAGCGTGGATATAGACGCACAACGGACATATATATATATGTACTCTTCTTGCCGTATCACGCACAAATACGAATTTACGCATGAATATAC

2 micron origin

1,080 1,100 1,120 1,140 1,160

CGTCTATTTATGTAGGATGAAAGGTAGTCTAGTACCTCCTGTGATATTATCCCATTCCATGCGGGGTATCGTATGCTTCCTTCAGCACTACCCTTTAGCTGTTCTAT
GCAGATAAATACATCCTACTTTCCATCAGATCATGGAGGACACTATAATAGGGTAAGGTACGCCCCATAGCATACGAAGGAAGTCGTGATGGGAAATCGACAAGATA

2 micron origin

1,180 1,200 1,220 1,240 1,260 1,280

ATGCTGCCACTCCTCAATTGGATTAGTCTCATCCTTCAATGCTATCATTTCCTTTGATATTGGATCGATCCGATGATAAGCTGTCAAACATGAGAATTGGGTAATAA
TACGACGGTGAGGAGTTAACCTAATCAGAGTAGGAAGTTACGATAGTAAAGGAAACTATAACCTAGCTAGGCTACTATTCGACAGTTTGTACTCTTAACCCATTATT

2 micron origin URA3

1,300 1,320 1,340 1,360 1,380

CTGATATAATTAAATTGAAGCTCTAATTTGTGAGTTTAGTATACATGCATTTACTTATAATACAGTTTTTTAGTTTTGCTGGCCGCATCTTCTCAAATATGCTTCCC
GACTATATTAATTTAACTTCGAGATTAAACACTCAAATCATATGTACGTAAATGAATATTATGTCAAAAAATCAAAACGACCGGCGTAGAAGAGTTTATACGAAGGG

URA3

1,400 1,420 1,440 1,460 1,480

AGCCTGCTTTTCTGTAACGTTCACCCTCTACCTTAGCATCCCTTCCCTTTGCAAATAGTCCTCTTCCAACAATAATAATGTCAGATCCTGTAGAGACCACATCATCC
TCGGACGAAAAGACATTGCAAGTGGGAGATGGAATCGTAGGGAAGGGAAACGTTTATCAGGAGAAGGTTGTTATTATTACAGTCTAGGACATCTCTGGTGTAGTAGG

URA3

1,500 1,520 1,540 1,560 1,580 1,600

ACGGTTCTATACTGTTGACCCAATGCGTCTCCCTTGTCATCTAAACCCACACCGGGTGTCATAATCAACCAATCGTAACCTTCATCTCTTCCACCCATGTCTCTTTG
TGCCAAGATATGACAACTGGGTTACGCAGAGGGAACAGTAGATTTGGGTGTGGCCCACAGTATTAGTTGGTTAGCATTGGAAGTAGAGAAGGTGGGTACAGAGAAAC

URA3

1,620 1,640 1,660 1,680 1,700

AGCAATAAAGCCGATAACAAAATCTTTGTCGCTCTTCGCAATGTCAACAGTACCCTTAGTATATTCTCCAGTAGATAGGGAGCCCTTGCATGACAATTCTGCTAACA
TCGTTATTTCGGCTATTGTTTTAGAAACAGCGAGAAGCGTTACAGTTGTCATGGGAATCATATAAGAGGTCATCTATCCCTCGGGAACGTACTGTTAAGACGATTGT
URA3
1,720 1,740 1,760 1,780 1,800

TCAAAAGGCCTCTAGGTTCCTTTGTTACTTCTTCTGCCGCCTGCTTCAAACCGCTAACAATACCTGGGCCCACCACACCGTGTGCATTCGTAATGTCTGCCCATTCT
AGTTTTCCGGAGATCCAAGGAAACAATGAAGAAGACGGCGGACGAAGTTTGGCGATTGTTATGGACCCGGGTGGTGTGGCACACGTAAGCATTACAGACGGGTAAGA
URA3
1,820 1,840 1,860 1,880 1,900 1,920

GCTATTCTGTATACACCCGCAGAGTACTGCAATTTGACTGTATTACCAATGTCAGCAAATTTTCTGTCTTCGAAGAGTAAAAAATTGTACTTGGCGGATAATGCCTT
CGATAAGACATATGTGGGCGTCTCATGACGTTAAACTGACATAATGGTTACAGTCGTTTAAAAGACAGAAGCTTCTCATTTTTTAACATGAACCGCCTATTACGGAA
URA3
1,940 1,960 1,980 2,000 2,020

TAGCGGCTTAACTGTGCCCTCCATGGAAAAATCAGTCAAGATATCCACATGTGTTTTTAGTAAACAAATTTTGGGACCTAATGCTTCAACTAACTCCAGTAATTCCT
ATCGCCGAATTGACACGGGAGGTACCTTTTTAGTCAGTTCTATAGGTGTACACAAAAATCATTTGTTTAAAACCCTGGATTACGAAGTTGATTGAGGTCATTAAGGA
URA3
2,040 2,060 2,080 2,100 2,120 2,140

TGGTGGTACGAACATCCAATGAAGCACACAAGTTTGTTTGCTTTTCGTGCATGATATTAAATAGCTTGGCAGCAACAGGACTAGGATGAGTAGCAGCACGTTCCTTA
ACCACCATGCTTGTAGGTTACTTCGTGTGTTCAAACAAACGAAAAGCACGTACTATAATTTATCGAACCGTCGTTGTCCTGATCCTACTCATCGTCGTGCAAGGAAT
URA3
2,160 2,180 2,200 2,220 2,240

TATGTAGCTTTCGACATGATTTATCTTCGTTTCCTGCATGTTTTTGTTCTGTGCAGTTGGGTTAAGAATACTGGGCAATTTCATGTTTCTTCAACACTACATATGCG
ATACATCGAAAGCTGTACTAAATAGAAGCAAAGGACGTACAAAAACAAGACACGTCAACCCAATTCTTATGACCCGTTAAAGTACAAAGAAGTTGTGATGTATACGC
URA3
2,260 2,280 2,300 2,320 2,340

TATATATACCAATCTAAGTCTGTGCTCCTTCCTTCGTTCTTCCTTCTGTTCGGAGATTACCGAATCAAAAAAATTTCAAAGAAACCGAAATCAAAAAAAAGAATAAA
ATATATATGGTTAGATTCAGACACGAGGAAGGAAGCAAGAAGGAAGACAAGCCTCTAATGGCTTAGTTTTTTTAAAGTTTCTTTGGCTTTAGTTTTTTTTCTTATTT
URA3
2,360 2,380 2,400 2,420 2,440 2,460

AAAAAAATGATGAATTGAATTGAAAAGCTAATTCTTGAAGACGAAAGGGCCTCGTGATACGCCTATTTTTATAGGTTAATGTCATGATAATAATGGTTTCTTAGACG
TTTTTTTACTACTTAACTTAACTTTTCGATTAAGAACTTCTGCTTTCCCGGAGCACTATGCGGATAAAAATATCCAATTACAGTACTATTATTACCAAAGAATCTGC
URA3
2,480 2,500 2,520 2,540 2,560

TCAGGTGGCACTTTTCGGGGAAATGTGCGCGGAACCCCTATTTGTTTATTTTTCTAAATACATTCAAATATGTATCCGCTCATGAGACAATAACCCTGATAAATGCT
AGTCCACCGTGAAAAGCCCCTTTACACGCGCCTTGGGGATAAACAAATAAAAAGATTTATGTAAGTTTATACATAGGCGAGTACTCTGTTATTGGGACTATTTACGA
2,580 2,600 2,620 2,640 2,660

TCAATAATATTGAAAAAGGAAGAGTATGAGTATTCAACATTTCCGTGTCGCCCTTATTCCCTTTTTTGCGGCATTTTGCCTTCCTGTTTTTGCTCACCCAGAAACGC
AGTTATTATAACTTTTTCCTTCTCATACTCATAAGTTGTAAAGGCACAGCGGGAATAAGGGAAAAAACGCCGTAAAACGGAAGGACAAAAACGAGTGGGTCTTTGCG

2,680 2,700 2,720 2,740 2,760 2,780

TGGTGAAAGTAAAAGATGCTGAAGATCAGTTGGGTGCACGAGTGGGTTACATCGAACTGGATCTCAACAGCGGTAAGATCCTTGAGAGTTTTCGCCCCGAAGAACGT
ACCACTTTCATTTTCTACGACTTCTAGTCAACCCACGTGCTCACCCAATGTAGCTTGACCTAGAGTTGTCGCCATTCTAGGAACTCTCAAAAGCGGGGCTTCTTGCA

2,800 2,820 2,840 2,860 2,880

TTTCCAATGATGAGCACTTTTAAAGTTCTGCTATGTGGCGCGGTATTATCCCGTATTGACGCCGGGCAAGAGCAACTCGGTCGCCGCATACACTATTCTCAGAATGA
AAAGGTTACTACTCGTGAAAATTTCAAGACGATACACCGCGCCATAATAGGGCATAACTGCGGCCCGTTCTCGTTGAGCCAGCGGCGTATGTGATAAGAGTCTTACT

AmpR

2,900 2,920 2,940 2,960 2,980

CTTGGTTGAGTACTCACCAGTCACAGAAAAGCATCTTACGGATGGCATGACAGTAAGAGAATTATGCAGTGCTGCCATAACCATGAGTGATAACACTGCGGCCAACT
GAACCAACTCATGAGTGGTCAGTGTCTTTTCGTAGAATGCCTACCGTACTGTCATTCTCTTAATACGTCACGACGGTATTGGTACTCACTATTGTGACGCCGGTTGA

AmpR

3,000 3,020 3,040 3,060 3,080 3,100

TACTTCTGACAACGATCGGAGGACCGAAGGAGCTAACCGCTTTTTTGCACAACATGGGGGATCATGTAACTCGCCTTGATCGTTGGGAACCGGAGCTGAATGAAGCC
ATGAAGACTGTTGCTAGCCTCCTGGCTTCCTCGATTGGCGAAAAAACGTGTTGTACCCCCTAGTACATTGAGCGGAACTAGCAACCCTTGGCCTCGACTTACTTCGG

AmpR

3,120 3,140 3,160 3,180 3,200

ATACCAAACGACGAGCGTGACACCACGATGCCTGTAGCAATGGCAACAACGTTGCGCAAACTATTAACTGGCGAACTACTTACTCTAGCTTCCCGGCAACAATTAAT
TATGGTTTGCTGCTCGCACTGTGGTGCTACGGACATCGTTACCGTTGTTGCAACGCGTTTGATAATTGACCGCTTGATGAATGAGATCGAAGGGCCGTTGTTAATTA

AmpR

3,220 3,240 3,260 3,280 3,300

AGACTGGATGGAGGCGGATAAAGTTGCAGGACCACTTCTGCGCTCGGCCCTTCCGGCTGGCTGGTTTATTGCTGATAAATCTGGAGCCGGTGAGCGTGGGTCTCGCG
TCTGACCTACCTCCGCCTATTTCAACGTCCTGGTGAAGACGCGAGCCGGGAAGGCCGACCGACCAAATAACGACTATTTAGACCTCGGCCACTCGCACCCAGAGCGC

AmpR

3,320 3,340 3,360 3,380 3,400 3,420

GTATCATTGCAGCACTGGGGCCAGATGGTAAGCCCTCCCGTATCGTAGTTATCTACACGACGGGGAGTCAGGCAACTATGGATGAACGAAATAGACAGATCGCTGAG
CATAGTAACGTCGTGACCCCGGTCTACCATTCGGGAGGGCATAGCATCAATAGATGTGCTGCCCCTCAGTCCGTTGATACCTACTTGCTTTATCTGTCTAGCGACTC

AmpR

3,440 3,460 3,480 3,500 3,520

ATAGGTGCCTCACTGATTAAGCATTGGTAACTGTCAGACCAAGTTTACTCATATATACTTTAGATTGATTTAAAACTTCATTTTTAATTTAAAAGGATCTAGGTGAA
TATCCACGGAGTGACTAATTCGTAACCATTGACAGTCTGGTTCAAATGAGTATATATGAAATCTAACTAAATTTTGAAGTAAAAATTAAATTTTCCTAGATCCACTT

AmpR

3,540 3,560 3,580 3,600 3,620

```
GATCCTTTTTGATAATCTCATGACCAAAATCCCTTAACGTGAGTTTTCGTTCCACTGAGCGTCAGACCCCGTAGAAAAGATCAAAGGATCTTCTTGAGATCCTTTTT
CTAGGAAAAACTATTAGAGTACTGGTTTTAGGGAATTGCACTCAAAAGCAAGGTGACTCGCAGTCTGGGGCATCTTTTCTAGTTTCCTAGAAGAACTCTAGGAAAAA
                          ColE1 origin
3,640    3,660    3,680    3,700    3,720    3,740

TTCTGCGCGTAATCTGCTGCTTGCAAACAAAAAAACCACCGCTACCAGCGGTGGTTTGTTTGCCGGATCAAGAGCTACCAACTCTTTTTCCGAAGGTAACTGGCTTC
AAGACGCGCATTAGACGACGAACGTTTGTTTTTTTGGTGGCGATGGTCGCCACCAAACAAACGGCCTAGTTCTCGATGGTTGAGAAAAAGGCTTCCATTGACCGAAG
                          ColE1 origin
3,760    3,780    3,800    3,820    3,840

AGCAGAGCGCAGATACCAAATACTGTCCTTCTAGTGTAGCCGTAGTTAGGCCACCACTTCAAGAACTCTGTAGCACCGCCTACATACCTCGCTCTGCTAATCCTGTT
TCGTCTCGCGTCTATGGTTTATGACAGGAAGATCACATCGGCATCAATCCGGTGGTGAAGTTCTTGAGACATCGTGGCGGATGTATGGAGCGAGACGATTAGGACAA
                          ColE1 origin
3,860    3,880    3,900    3,920    3,940

ACCAGTGGCTGCTGCCAGTGGCGATAAGTCGTGTCTTACCGGGTTGGACTCAAGACGATAGTTACCGGATAAGGCGCAGCGGTCGGGCTGAACGGGGGGTTCGTGCA
TGGTCACCGACGACGGTCACCGCTATTCAGCACAGAATGGCCCAACCTGAGTTCTGCTATCAATGGCCTATTCCGCGTCGCCAGCCCGACTTGCCCCCCAAGCACGT
                          ColE1 origin
3,960    3,980    4,000    4,020    4,040    4,060

CACAGCCCAGCTTGGAGCGAACGACCTACACCGAACTGAGATACCTACAGCGTGAGCTATGAGAAAGCGCCACGCTTCCCGAAGGGAGAAAGGCGGACAGGTATCCG
GTGTCGGGTCGAACCTCGCTTGCTGGATGTGGCTTGACTCTATGGATGTCGCACTCGATACTCTTTCGCGGTGCGAAGGGCTTCCCTCTTTCCGCCTGTCCATAGGC
                          ColE1 origin
4,080    4,100    4,120    4,140    4,160

GTAAGCGGCAGGGTCGGAACAGGAGAGCGCACGAGGGAGCTTCCAGGGGGAAACGCCTGGTATCTTTATAGTCCTGTCGGGTTTCGCCACCTCTGACTTGAGCGTCG
CATTCGCCGTCCCAGCCTTGTCCTCTCGCGTGCTCCCTCGAAGGTCCCCCTTTGCGGACCATAGAAATATCAGGACAGCCCAAAGCGGTGGAGACTGAACTCGCAGC
                          ColE1 origin
4,180    4,200    4,220    4,240    4,260    4,280

ATTTTTGTGATGCTCGTCAGGGGGGCGGAGCCTATGGAAAAACGCCAGCAACGCGGCCTTTTTACGGTTCCTGGCCTTTTGCTGGCCTTTTGCTCACATGTTCTTTC
TAAAAACACTACGAGCAGTCCCCCCGCCTCGGATACCTTTTTGCGGTCGTTGCGCCGGAAAAATGCCAAGGACCGGAAAACGACCGGAAAACGAGTGTACAAGAAAG
             ColE1 origin
4,300    4,320    4,340    4,360    4,380

CTGCGTTATCCCCTGATTCTGTGGATAACCGTATTACCGCCTTTGAGTGAGCTGATACCGCTCGCCGCAGCCGAACGACCGAGCGCAGCGAGTCAGTGAGCGAGGAA
GACGCAATAGGGGACTAAGACACCTATTGGCATAATGGCGGAAACTCACTCGACTATGGCGAGCGGCGTCGGCTTGCTGGCTCGCGTCGCTCAGTCACTCGCTCCTT
4,400    4,420    4,440    4,460    4,480

GCGGAAGAGCGCCCAATACGCAAACCGCCTCTCCCCGCGCGTTGGCCGATTCATTAATGCAGCTGGCACGACAGGTTTCCCGACTGGAAAGCGGGCAGTGAGCGCAA
CGCCTTCTCGCGGGTTATGCGTTTGGCGGAGAGGGGCGCGCAACCGGCTAAGTAATTACGTCGACCGTGCTGTCCAAAGGGCTGACCTTTCGCCCGTCACTCGCGTT
4,500    4,520    4,540    4,560    4,580    4,600
```

CGCAATTAATGTGAGTTAGCTCACTCATTAGGCACCCCAGGCTTTACACTTTATGCTTCCGGCTCGTATGTTGTGTGGAATTGTGAGCGGATAACAATTTCACACAG
GCGTTAATTACACTCAATCGAGTGAGTAATCCGTGGGGTCCGAAATGTGAAATACGAAGGCCGAGCATACAACACACCTTAACACTCGCCTATTGTTAAAGTGTGTC

lac promoter LacO

4,620 4,640 4,660 4,680 4,700

GAAACAGCTATGACCATGATTACGCCAAGCTTACCAGTTCTCACACGGAACACCACTAATGGACACAAATTCGAAATACTTTGACCCTATTTTCGAGGACCTTGTCA
CTTTGTCGATACTGGTACTAATGCGGTTCGAATGGTCAAGAGTGTGCCTTGTGGTGATTACCTGTGTTTAAGCTTTATGAAACTGGGATAAAAGCTCCTGGAACAGT

M13-rev GAPDH promoter

4,720 4,740 4,760 4,780 4,800

CCTTGAGCCCAAGAGAGCCAAGATTTAAATTTTCCTATGACTTGATGCAAATTCCCAAAGCTAATAACATGCAAGACACGTACGGTCAAGAAGACATATTTGACCTC
GGAACTCGGGTTCTCTCGGTTCTAAATTTAAAAGGATACTGAACTACGTTTAAGGGTTTCGATTATTGTACGTTCTGTGCATGCCAGTTCTTCTGTATAAACTGGAG

GAPDH promoter

4,820 4,840 4,860 4,880 4,900 4,920

TTAACAGGTTCAGACGCGACTGCCTCATCAGTAAGACCCGTTGAAAAGAACTTACCTGAAAAAAACGAATATATACTAGCGTTGAATGTTAGCGTCAACAACAAGAA
AATTGTCCAAGTCTGCGCTGACGGAGTAGTCATTCTGGGCAACTTTTCTTGAATGGACTTTTTTTGCTTATATATGATCGCAACTTACAATCGCAGTTGTTGTTCTT

GAPDH promoter

4,940 4,960 4,980 5,000 5,020

GTTTAATGACGCGGAGGCCAAGGCAAAAAGATTCCTTGATTACGTAAGGGAGTTAGAATCATTTTGAATAAAAAACACGCTTTTTCAGTTCGAGTTTATCATTATCA
CAAATTACTGCGCCTCCGGTTCCGTTTTTCTAAGGAACTAATGCATTCCCTCAATCTTAGTAAAACTTATTTTTTGTGCGAAAAAGTCAAGCTCAAATAGTAATAGT

GAPDH promoter

5,040 5,060 5,080 5,100 5,120

ATACTGCCATTTCAAAGAATACGTAAATAATTAATAGTAGTGATTTTCCTAACTTTATTTAGTCAAAAAATTAGCCTTTTAATTCTGCTGTAACCCGTACATGCCCA
TATGACGGTAAAGTTTCTTATGCATTTATTAATTATCATCACTAAAAGGATTGAAATAAATCAGTTTTTTAATCGGAAAATTAAGACGACATTGGGCATGTACGGGT

GAPDH promoter

5,140 5,160 5,180 5,200 5,220 5,240

AAATAGGGGGCGGGTTACACAGAATATATAACATCGTAGGTGTCTGGGTGAACAGTTTATTCCTGGCATCCACTAAATATAATGGAGCCCGCTTTTTAAGCTGGCAT
TTTATCCCCCGCCCAATGTGTCTTATATATTGTAGCATCCACAGACCCACTTGTCAAATAAGGACCGTAGGTGATTTATATTACCTCGGGCGAAAAATTCGACCGTA

GAPDH promoter

5,260 5,280 5,300 5,320 5,340

CCAGAAAAAAAAAGAATCCCAGCACCAAAATATTGTTTTCTTCACCAACCATCAGTTCATAGGTCCATTCTCTTAGCGCAACTACAGAGAACAGGGGCACAAACAGG
GGTCTTTTTTTTTCTTAGGGTCGTGGTTTTATAACAAAAGAAGTGGTTGGTAGTCAAGTATCCAGGTAAGAGAATCGCGTTGATGTCTCTTGTCCCCGTGTTTGTCC

GAPDH promoter

5,360 5,380 5,400 5,420 5,440

CAAAAAACGGGCACAACCTCAATGGAGTGATGCAACCTGCCTGGAGTAAATGATGACACAAGGCAATTGACCCACGCATGTATCTATCTCATTTTCTTACACCTTCT
GTTTTTTGCCCGTGTTGGAGTTACCTCACTACGTTGGACGGACCTCATTTACTACTGTGTTCCGTTAACTGGGTGCGTACATAGATAGAGTAAAAGAATGTGGAAGA

GAPDH promoter

5,460 5,480 5,500 5,520 5,540 5,560

ATTACCTTCTGCTCTCTCTGATTTGGAAAAAGCTGAAAAAAAAAGGTTGAAACCAGTTCCCTGAAATTATTCCCCTACTTGACTAATAAGTATATAAAGACGGTAGGT
TAATGGAAGACGAGAGAGACTAAACCTTTTTCGACTTTTTTTTTCCAACTTTGGTCAAGGGACTTTAATAAGGGGATGAACTGATTATTCATATATTTCTGCCATCCA

GAPDH promoter

5,580 5,600 5,620 5,640 5,660

ATTGATTGTAATTCTGTAAATCTATTTCTTAAACTTCTTAAATTCTACTTTTATAGTTAGTCTTTTTTTTAGTTTTAAAACACCAAGAACTTAGTTTCGAATAAACA
TAACTAACATTAAGACATTTAGATAAAGAATTTGAAGAATTTAAGATGAAAATATCAATCAGAAAAAAAATCAAAATTTTGTGGTTCTTGAATCAAAGCTTATTTGT

GAPDH promoter

5,680 5,700 5,720 5,740 5,760

CACATAAACAGAATTCATGAGATTCCCATCTATCTTCACCGCTGTTTTGTTCGCTGCTTCTTCTGCTTTGGCTGCTCCAGCTAACACCACCACCGAAGACGAAACCG
GTGTATTTGTCTTAAGTACTCTAAGGGTAGATAGAAGTGGCGACAAAACAAGCGACGAAGAAGACGAAACCGACGAGGTCGATTGTGGTGGTGGCTTCTGCTTTGGC

1 2 3 4 5 6 7 8 9 10 11 12 13 14 15 16 17 18 19 20 21 22 23 24 25 26 27 28 29 30
M R F P S I F T A V L F A A S S A L A A P A N T T T E D E T

Mating factor alpha leader

5,780 5,800 5,820 5,840 5,860 5,880

CTCAAATCCCAGCTGAAGCTGTTATCGACTACTCTGACTTGGAAGGTGACTTCGACGCTGCTGCTTTGCCATTGTCTAACTCTACCAACAACGGTTTGTCTTCTACC
GAGTTTAGGGTCGACTTCGACAATAGCTGATGAGACTGAACCTTCCACTGAAGCTGCGACGACGAAACGGTAACAGATTGAGATGGTTGTTGCCAAACAGAAGATGG

31 32 33 34 35 36 37 38 39 40 41 42 43 44 45 46 47 48 49 50 51 52 53 54 55 56 57 58 59 60 61 62 63 64 65 66
A Q I P A E A V I D Y S D L E G D F D A A A L P L S N S T N N G L S S T

Mating factor alpha leader

5,900 5,920 5,940 5,960 5,980

AACACCACCATCGCTTCTATCGCTGCTAAGGAAGAAGGTGTTCAATTGGACAAGAGAGAAagatctggcggccgcgcatgcgctagcctcgagGGTGGATCTGATTA
TTGTGGTGGTAGCGAAGATAGCGACGATTCCTTCTTCCACAAGTTAACCTGTTCTCTCTTtctagaccgccggcgcgtacgcgatcggagctcCCACCTAGACTAAT

67 68 69 70 71 72 73 74 75 76 77 78 79 80 81 82 83 84 85 86
N T T I A S I A A K E E G V Q L D K R E

Mating factor alpha leader FLAG

6,000 6,020 6,040 6,060 6,080

CAAGGATGACGATGACAAGGCTGGAGGTTCGAGCGCCAAAAGCTCTTTTATCTCAACCACTACTACTGATTTAACAAGTATAAACACTAGTGCGTATTCCACTGGAT
GTTCCTACTGCTACTGTTCCGACCTCCAAGCTCGCGGTTTTCGAGAAAATAGAGTTGGTGATGATGACTAAATTGTTCATATTTGTGATCACGCATAAGGTGACCTA

2 4 6 8 10 12 14 16 18 20 22 24
S A K S S F I S T T T T D L T S I N T S A Y S T G

FLAG 3' Half of α-agglutinin

6,100 6,120 6,140 6,160 6,180 6,200

CCATTTCCACAGTAGAAACAGGCAATCGAACTACATCAGAAGTGATCAGTCATGTGGTGACTACCAGCACAAAACTGTCTCCAACTGCTACTACCAGCCTGACAATT
GGTAAAGGTGTCATCTTTGTCCGTTAGCTTGATGTAGTCTTCACTAGTCAGTACACCACTGATGGTCGTGTTTTGACAGAGGTTGACGATGATGGTCGGACTGTTAA

26 28 30 32 34 36 38 40 42 44 46 48 50 52 54 56 58 60

S I S T V E T G N R T T S E V I S H V V T T S T K L S P T A T T S L T I

3' Half of α-agglutinin

6,220 6,240 6,260 6,280 6,300

GCACAAACCAGTATCTATTCTACTGACTCATATATCACAGTAGGAACAGATATTCACACCACATCAGAAGTGATTAGTGATGTGGAAACCATTAGCAGAGAAACAGC
CGTGTTTGGTCATAGATAAGATGACTGAGTATATAGTGTCATCCTTGTCTATAAGTGTGGTGTAGTCTTCACTAATCACTACACCTTTGGTAATCGTCTCTTTGTCG

62 64 66 68 70 72 74 76 78 80 82 84 86 88 90 92 94 96

A Q T S I Y S T D S Y I T V G T D I H T T S E V I S D V E T I S R E T A

3' Half of α-agglutinin

6,320 6,340 6,360 6,380 6,400 6,420

TTCGACCGTTGTAGCCGCTCCAACCTCAACAACTGGATGGACAGGCGCTATGAATACTTACATCTCGCAATTTACATCCTCTTCTTTCGCAACAATCAACAGCACAC
AAGCTGGCAACATCGGCGAGGTTGGAGTTGTTGACCTACCTGTCCGCGATACTTATGAATGTAGAGCGTTAAATGTAGGAGAAGAAAGCGTTGTTAGTTGTCGTGTG

98 100 102 104 106 108 110 112 114 116 118 120 122 124 126 128 130 132

S T V V A A P T S T T G W T G A M N T Y I S Q F T S S S F A T I N S T

3' Half of α-agglutinin

6,440 6,460 6,480 6,500 6,520

CAATAATCTCTTCATCAGCAGTATTTGAAACCTCAGATGCTTCAATTGTCAATGTGCACACTGAAAATATCACGAATACTGCTGCTGTTCCATCTGAAGAGCCCACT
GTTATTAGAGAAGTAGTCGTCATAAACTTTGGAGTCTACGAAGTTAACAGTTACACGTGTGACTTTTATAGTGCTTATGACGACGACAAGGTAGACTTCTCGGGTGA

134 136 138 140 142 144 146 148 150 152 154 156 158 160 162 164 166 168

P I I S S S A V F E T S D A S I V N V H T E N I T N T A A V P S E E P T

3' Half of α-agglutinin

6,540 6,560 6,580 6,600 6,620

TTTGTAAATGCCACGAGAAACTCCTTAAATTCCTTCTGCAGCAGCAAACAGCCATCCAGTCCCTCATCTTATACGTCTTCCCCACTCGTATCGTCCCTCTCCGTAAG
AAACATTTACGGTGCTCTTTGAGGAATTTAAGGAAGACGTCGTCGTTTGTCGGTAGGTCAGGGAGTAGAATATGCAGAAGGGGTGAGCATAGCAGGGAGAGGCATTC

170 172 174 176 178 180 182 184 186 188 190 192 194 196 198 200 202 204

F V N A T R N S L N S F C S S K Q P S S P S S Y T S S P L V S S L S V S

3' Half of α-agglutinin

6,640 6,660 6,680 6,700 6,720 6,740

CAAAACATTACTAAGCACCAGTTTTACGCCTTCTGTGCCAACATCTAATACATATATCAAAACGAAAAATACGGGTTACTTTGAGCACACGGCTTTGACAACATCTT
GTTTTGTAATGATTCGTGGTCAAAATGCGGAAGACACGGTTGTAGATTATGTATATAGTTTTGCTTTTTATGCCCAATGAAACTCGTGTGCCGAAACTGTTGTAGAA

206 208 210 212 214 216 218 220 222 224 226 228 230 232 234 236 238

K T L L S T S F T P S V P T S N T Y I K T K N T G Y F E H T A L T T S

3' Half of α-agglutinin

6,760 6,780 6,800 6,820 6,840

```
CAGTTGGCCTTAATTCTTTTAGTGAAACAGCAGTCTCATCTCAGGGAACGAAAATTGACACCTTTTTAGTGTCATCCTTGATCGCATATCCTTCTTCTGCATCAGGA
GTCAACCGGAATTAAGAAAATCACTTTGTCGTCAGAGTAGAGTCCCTTGCTTTTAACTGTGGAAAAATCACAGTAGGAACTAGCGTATAGGAAGAAGACGTAGTCCT
240 242 244 246 248 250 252 254 256 258 260 262 264 266 268 270 272 274
S V G L N S F S E T A V S S Q G T K I D T F L V S S L I A Y P S S A S G
3' Half of α-agglutinin
6,860 6,880 6,900 6,920 6,940

AGCCAATTGTCCGGTATCCAACAGAATTTCACATCAACTTCTCTCATGATTTCAACCTATGAAGGTAAAGCGTCTATATTTTTCTCAGCTGAGCTCGGTTCGATCAT
TCGGTTAACAGGCCATAGGTTGTCTTAAAGTGTAGTTGAAGAGAGTACTAAAGTTGGATACTTCCATTTCGCAGATATAAAAAGAGTCGACTCGAGCCAAGCTAGTA
276 278 280 282 284 286 288 290 292 294 296 298 300 302 304 306 308 310
S Q L S G I Q Q N F T S T S L M I S T Y E G K A S I F F S A E L G S I I
3' Half of α-agglutinin
6,960 6,980 7,000 7,020 7,040 7,060

TTTTCTGCTTTTGTCGTACCTGCTATTCTAAAACGGGTACTGTACAGTTAGTACATTGAGTCGAAATATACGAAATTATTGTTCATAATTTTCATCCTGGCTCTTTT
AAAAGACGAAAACAGCATGGACGATAAGATTTTGCCCATGACATGTCAATCATGTAACTCAGCTTTATATGCTTTAATAACAAGTATTAAAAGTAGGACCGAGAAAA
312 314 316 318 320
F L L L S Y L L F *
3' Half of α-agglutinin
7,080 7,100 7,120 7,140 7,160

TTTCTTCAACCATAGTTAAATGGACAGTTCATATCTTAACTCTAATAATACTTTTCTAGTTCTTATCCTTTTCCGTCTCACCGCAGATTTTATCATAGTATTAAATT
AAAGAAGTTGGTATCAATTTACCTGTCAAGTATAGAATTGAGATTATTATGAAAAGATCAAGAATAGGAAAAGGCAGAGTGGCGTCTAAAATAGTATCATAATTTAA
3' Half of α-agglutinin
7,180 7,200 7,220 7,240 7,260

TATATTTTGTTCGTAAAAAGAAAAATTTGTGAGCGTTACCGCTCGTTTCATTACCCGAAGGCTGTTTCAGTAGACCACTGATTAAGTAAGTAGATGAAAAAATTTCA
ATATAAAACAAGCATTTTTCTTTTTAAACACTCGCAATGGCGAGCAAAGTAATGGGCTTCCGACAAAGTCATCTGGTGACTAATTCATTCATCTACTTTTTTAAAGT
3' Half of α-agglutinin
7,280 7,300 7,320 7,340 7,360 7,380

TCACCATGAAAGAGTTCGATGAGAGCTACTTTTTCAAATGCTTAACAGCTAACCGCCATTCAATAATGTTACGCTCTCTTCATTCTGCGGCTACGTTATCTAACAAG
AGTGGTACTTTCTCAAGCTACTCTCGATGAAAAAGTTTACGAATTGTCGATTGGCGGTAAGTTATTACAATGCGAGAGAAGTAAGACGCCGATGCAATAGATTGTTC
3' Half of α-agglutinin
7,400 7,420 7,440 7,460 7,480

AGGTTTTACTCTCTCATATCTCATTCAAATAGAAAGAACATAATCAAAGGTACCGCGATGTAGTAAAACTAGCTAGACCGAGAAAGAGACTAGAAATGCAAAAGGCA
TCCAAAATGAGAGAGTATAGAGTAAGTTTATCTTTCTTGTATTAGTTTCCATGGCGCTACATCATTTTGATCGATCTGGCTCTTTCTCTGATCTTTACGTTTTCCGT
3' Half of α-agglutinin
7,500 7,520 7,540 7,560 7,580
```

CTTCTACAATGGCTGCCATCATTATTATCCGATGTGACGCTGCATTTTTTTTTTTTTTTTTTTTTTTTTTTTTTTTTTTTTTTTTTTTTTTTTTTTTGTACAAATATCATAAA
GAAGATGTTACCGACGGTAGTAATAATAGGCTACACTGCGACGTAAAAAAAAAAAAAAAAAAAAAAAAAAAAAAAAAAAAAAAAAAAAAAACATGTTTATAGTATTT

7,600 7,620 7,640 7,660 7,680 7,700

AAAAGAGAATCTTTTTAAGCAAGGATTTTCTTAACTTCTTCGGCGACAGCATCACCGACTTCGGTGGTACTGTTGGAACCACCTAAATCACCAGTTCTGATACCTGC
TTTTCTCTTAGAAAAATTCGTTCCTAAAAGAATTGAAGAAGCCGCTGTCGTAGTGGCTGAAGCCACCATGACAACCTTGGTGGATTTAGTGGTCAAGACTATGGACG

7,720 7,740 7,760 7,780 7,800

ATCCAAAACCTTTTTAACTGCATCTTCAATGGCCTTACCTTCTTCAGGCAAGTTCAATGACAATTTCAACATCATTGCAGCAGACAAGATAGTGGCGATAGGGTCAA
TAGGTTTTGGAAAAATTGACGTAGAAGTTACCGGAATGGAAGAAGTCCGTTCAAGTTACTGTTAAAGTTGTAGTAACGTCGTCTGTTCTATCACCGCTATCCCAGTT

7,820 7,840 7,860 7,880 7,900

CCTTATTCTTTGGCAAATCTGGAGCAGAACCGTGGCATGGTTCGTACAAACCAAATGCGGTGTTCTTGTCTGGCAAAGAGGCCAAGGACGCAGATGGCAACAAACCC
GGAATAAGAAACCGTTTAGACCTCGTCTTGGCACCGTACCAAGCATGTTTGGTTTACGCCACAAGAACAGACCGTTTCTCCGGTTCCTGCGTCTACCGTTGTTTGGG

7,920 7,940 7,960 7,980 8,000 8,020

AAGGAACCTGGGATAACGGAGGCTTCATCGGAGATGATATCACCAAACATGTTGCTGGTGATTATAATACCATTTAGGTGGGTTGGGTTCTTAACTAGGATCATGGC
TTCCTTGGACCCTATTGCCTCCGAAGTAGCCTCTACTATAGTGGTTTGTACAACGACCACTAATATTATGGTAAATCCACCCAACCCAAGAATTGATCCTAGTACCG

8,040 8,060 8,080 8,100 8,120

GGCAGAATCAATCAATTGATGTTGAACCTTCAATGTAGGAAATTCGTTCTTGATGGTTTCCTCCACAGTTTTTCTCCATAATCTTGAAGAGGCCAAAACATTAGCTT
CCGTCTTAGTTAGTTAACTACAACTTGGAAGTTACATCCTTTAAGCAAGAACTACCAAAGGAGGTGTCAAAAAGAGGTATTAGAACTTCTCCGGTTTTGTAATCGAA

8,140 8,160 8,180 8,200 8,220

TATCCAAGGACCAAATAGGCAATGGTGGCTCATGTTGTAGGGCCATGAAAGCGGCCATTCTTGTGATTCTTTGCACTTCTGGAACGGTGTATTGTTCACTATCCCAA
ATAGGTTCCTGGTTTATCCGTTACCACCGAGTACAACATCCCGGTACTTTCGCCGGTAAGAACACTAAGAAACGTGAAGACCTTGCCACATAACAAGTGATAGGGTT

8,240 8,260 8,280 8,300 8,320 8,340

GCGACACCATCACCATCGTCTTCCTTTCTCTTACCAAAGTAAATACCTCCCACTAATTCTCTGACAACAACGAAGTCAGTACCTTTAGCAAATTGTGGCTTGATTGG
CGCTGTGGTAGTGGTAGCAGAAGGAAAGAGAATGGTTTCATTTATGGAGGGTGATTAAGAGACTGTTGTTGCTTCAGTCATGGAAATCGTTTAACACCGAACTAACC

8,360 8,380 8,400 8,420 8,440

AGATAAGTCTAAAAGAGAGTCGGATGCAAAGTTACATGGTCTTAAGTTGGCGTACAATTGAAGTTCTTTACGGATTTTTAGTAAACCTTGTTCAGGTCTAACACTAC
TCTATTCAGATTTTCTCTCAGCCTACGTTTCAATGTACCAGAATTCAACCGCATGTTAACTTCAAGAAATGCCTAAAAATCATTTGGAACAAGTCCAGATTGTGATG

8,460 8,480 8,500 8,520 8,540 8,560

CTGTACCCCATTTAGGACCACCCACAGCACCTAACAAAACGGCATCAACCTTCTTGGAGGCTTCCAGCGCCTCATCTGGAAGTGGGACACCTGTAGCATCGATAGCA
GACATGGGGTAAATCCTGGTGGGTGTCGTGGATTGTTTTGCCGTAGTTGGAAGAACCTCCGAAGGTCGCGGAGTAGACCTTCACCCTGTGGACATCGTAGCTATCGT

8,580 8,600 8,620 8,640 8,660

GCACCACCAATTAAATGATTTTCGAAATCGAACTTGACATTGGAACGAACATCAGAAATAGCTTTAAGAACCTTAATGGCTTCGGCTGTGATTTCTTGACCAACGTG
CGTGGTGGTTAATTTACTAAAAGCTTTAGCTTGAACTGTAACCTTGCTTGTAGTCTTTATCGAAATTCTTGGAATTACCGAAGCCGACACTAAAGAACTGGTTGCAC

8,680 8,700 8,720 8,740 8,760

GTCACCTGGCAAAACGACGATCTTCTTAGGGGCAGACATTACAATGGTATATCCTTGAAATATATATAAAAAAAAAAAAAAAAAAAAAAAAAAAAAAATGCAGCTTC
CAGTGGACCGTTTTGCTGCTAGAAGAATCCCCGTCTGTAATGTTACCATATAGGAACTTTATATATATTTTTTTTTTTTTTTTTTTTTTTTTTTTTTTACGTCGAAG

8,780 8,800 8,820 8,840 8,860 8,880

TCAATGATATTCGAATACGCTTTGAGGAGATACAGCCTAATATCCGACAAACTGTTTTACAGATTTACGATCGTACTTGTTACCCATCATTGAATTTTGAACATCCG
AGTTACTATAAGCTTATGCGAAACTCCTCTATGTCGGATTATAGGCTGTTTGACAAAATGTCTAAATGCTAGCATGAACAATGGGTAGTAACTTAAAACTTGTAGGC

8,900 8,920 8,940 8,960 8,980

AACCTGGGAGTTTTCCCTGAAACAGATAGTATATTTGAACCTGTATAATAATATATAGTCTAGCGCTTTACGGAAGACAATGTATGTATTTCGGTTCCTGGAGAAAC
TTGGACCCTCAAAAGGGACTTTGTCTATCATATAAACTTGGACATATTATTATATATCAGATCGCGAAATGCCTTCTGTTACATACATAAAGCCAAGGACCTCTTTG

9,000 9,020 9,040 9,060 9,080

TATTGCATCTATTGCATAGGTAATCTTGCACGTCGCATCCCCGGTTCATTTTCTGCGTTTCCATCTTGCACTTCAATAGCATATCTTT
ATAACGTAGATAACGTATCCATTAGAACGTGCAGCGTAGGGGCCAAGTAAAAGACGCAAAGGTAGAACGTGAAGTTATCGTATAGAAA

9,100 9,110 9,120 9,130 9,140 9,150 9,160 9,170 9,180

System 3 anti-lys Nb strain (10529 bp)

GTTAACGAAGCATCTGTGCTTCATTTTGTAGAACAAAAATGCAACGCGAGAGCGCTAATTTTTCAAACAAAGAATCTGAGCTGCATTTTTACAGAACAGAAATGCAA
CAATTGCTTCGTAGACACGAAGTAAAACATCTTGTTTTTACGTTGCGCTCTCGCGATTAAAAAGTTTGTTTCTTAGACTCGACGTAAAAATGTCTTGTCTTTACGTT

2 micron origin

20 40 60 80 100

CGCGAAAGCGCTATTTTACCAACGAAGAATCTGTGCTTCATTTTTGTAAAACAAAAATGCAACGCGAGAGCGCTAATTTTTCAAACAAAGAATCTGAGCTGCATTTT
GCGCTTTCGCGATAAAATGGTTGCTTCTTAGACACGAAGTAAAAACATTTTGTTTTTACGTTGCGCTCTCGCGATTAAAAAGTTTGTTTCTTAGACTCGACGTAAAA

2 micron origin

120 140 160 180 200

TACAGAACAGAAATGCAACGCGAGAGCGCTATTTTACCAACAAAGAATCTATACTTCTTTTTTGTTCTACAAAAATGCATCCCGAGAGCGCTATTTTTCTAACAAAG
ATGTCTTGTCTTTACGTTGCGCTCTCGCGATAAAATGGTTGTTTCTTAGATATGAAGAAAAAACAAGATGTTTTTACGTAGGGCTCTCGCGATAAAAAGATTGTTTC

2 micron origin

220 240 260 280 300 320

CATCTTAGATTACTTTTTTTCTCCTTTGTGCGCTCTATAATGCAGTCTCTTGATAACTTTTTGCACTGTAGGTCCGTTAAGGTTAGAAGAAGGCTACTTTGGTGTCT
GTAGAATCTAATGAAAAAAAGAGGAAACACGCGAGATATTACGTCAGAGAACTATTGAAAAACGTGACATCCAGGCAATTCCAATCTTCTTCCGATGAAACCACAGA

2 micron origin

340 360 380 400 420

ATTTTCTCTTCCATAAAAAAAGCCTGACTCCACTTCCCGCGTTTACTGATTACTAGCGAAGCTGCGGGTGCATTTTTTCAAGATAAAGGCATCCCCGATTATATTCT
TAAAAGAGAAGGTATTTTTTTCGGACTGAGGTGAAGGGCGCAAATGACTAATGATCGCTTCGACGCCCACGTAAAAAAGTTCTATTTCCGTAGGGGCTAATATAAGA

2 micron origin

440 460 480 500 520

ATACCGATGTGGATTGCGCATACTTTGTGAACAGAAAGTGATAGCGTTGATGATTCTTCATTGGTCAGAAAATTATGAACGGTTTCTTCTATTTTGTCTCTATATAC
TATGGCTACACCTAACGCGTATGAAACACTTGTCTTTCACTATCGCAACTACTAAGAAGTAACCAGTCTTTTAATACTTGCCAAAGAAGATAAAACAGAGATATATG

2 micron origin

540 560 580 600 620 640

TACGTATAGGAAATGTTTACATTTTCGTATTGTTTTCGATTCACTCTATGAATAGTTCTTACTACAATTTTTTTGTCTAAAGAGTAATACTAGAGATAAACATAAAA
ATGCATATCCTTTACAAATGTAAAAGCATAACAAAAGCTAAGTGAGATACTTATCAAGAATGATGTTAAAAAAACAGATTTCTCATTATGATCTCTATTTGTATTTT

2 micron origin

660 680 700 720 740

AATGTAGAGGTCGAGTTTAGATGCAAGTTCAAGGAGCGAAAGGTGGATGGGTAGGTTATATAGGGATATAGCACAGAGATATATAGCAAAGAGATACTTTTGAGCAA
TTACATCTCCAGCTCAAATCTACGTTCAAGTTCCTCGCTTTCCACCTACCCATCCAATATATCCCTATATCGTGTCTCTATATATCGTTTCTCTATGAAAACTCGTT

2 micron origin

760 780 800 820 840

TGTTTGTGGAAGCGGTATTCGCAATATTTTAGTAGCTCGTTACAGTCCGGTGCGTTTTTGGTTTTTTGAAAGTGCGTCTTCAGAGCGCTTTTGGTTTTCAAAAGCGC
ACAAACACCTTCGCCATAAGCGTTATAAAATCATCGAGCAATGTCAGGCCACGCAAAAACCAAAAAACTTTCACGCAGAAGTCTCGCGAAAACCAAAAGTTTTCGCG

2 micron origin

860 880 900 920 940 960

TCTGAAGTTCCTATACTTTCTAGCTAGAGAATAGGAACTTCGGAATAGGAACTTCAAAGCGTTTCCGAAAACGAGCGCTTCCGAAAATGCAACGCGAGCTGCGCACA
AGACTTCAAGGATATGAAAGATCGATCTCTTATCCTTGAAGCCTTATCCTTGAAGTTTCGCAAAGGCTTTTGCTCGCGAAGGCTTTTACGTTGCGCTCGACGCGTGT

2 micron origin

980 1,000 1,020 1,040 1,060

TACAGCTCACTGTTCACGTCGCACCTATATCTGCGTGTTGCCTGTATATATATATACATGAGAAGAACGGCATAGTGCGTGTTTATGCTTAAATGCGTACTTATATG
ATGTCGAGTGACAAGTGCAGCGTGGATATAGACGCACAACGGACATATATATATATGTACTCTTCTTGCCGTATCACGCACAAATACGAATTTACGCATGAATATAC

2 micron origin

1,080 1,100 1,120 1,140 1,160

CGTCTATTTATGTAGGATGAAAGGTAGTCTAGTACCTCCTGTGATATTATCCCATTCCATGCGGGGTATCGTATGCTTCCTTCAGCACTACCCTTTAGCTGTTCTAT
GCAGATAAATACATCCTACTTTCCATCAGATCATGGAGGACACTATAATAGGGTAAGGTACGCCCCATAGCATACGAAGGAAGTCGTGATGGGAAATCGACAAGATA

2 micron origin

1,180 1,200 1,220 1,240 1,260 1,280

ATGCTGCCACTCCTCAATTGGATTAGTCTCATCCTTCAATGCTATCATTTCCTTTGATATTGGATCGATCCGATGATAAGCTGTCAAACATGAGAATTGGGTAATAA
TACGACGGTGAGGAGTTAACCTAATCAGAGTAGGAAGTTACGATAGTAAAGGAAACTATAACCTAGCTAGGCTACTATTCGACAGTTTGTACTCTTAACCCATTATT

2 micron origin URA3

1,300 1,320 1,340 1,360 1,380

CTGATATAATTAAATTGAAGCTCTAATTTGTGAGTTTAGTATACATGCATTTACTTATAATACAGTTTTTTAGTTTTGCTGGCCGCATCTTCTCAAATATGCTTCCC
GACTATATTAATTTAACTTCGAGATTAAACACTCAAATCATATGTACGTAAATGAATATTATGTCAAAAAATCAAAACGACCGGCGTAGAAGAGTTTATACGAAGGG

URA3

1,400 1,420 1,440 1,460 1,480

AGCCTGCTTTTCTGTAACGTTCACCCTCTACCTTAGCATCCCTTCCCTTTGCAAATAGTCCTCTTCCAACAATAATAATGTCAGATCCTGTAGAGACCACATCATCC
TCGGACGAAAAGACATTGCAAGTGGGAGATGGAATCGTAGGGAAGGGAAACGTTTATCAGGAGAAGGTTGTTATTATTACAGTCTAGGACATCTCTGGTGTAGTAGG

URA3

1,500 1,520 1,540 1,560 1,580 1,600

ACGGTTCTATACTGTTGACCCAATGCGTCTCCCTTGTCATCTAAACCCACACCGGGTGTCATAATCAACCAATCGTAACCTTCATCTCTTCCACCCATGTCTCTTTG
TGCCAAGATATGACAACTGGGTTACGCAGAGGGAACAGTAGATTTGGGTGTGGCCCACAGTATTAGTTGGTTAGCATTGGAAGTAGAGAAGGTGGGTACAGAGAAAC

URA3

1,620 1,640 1,660 1,680 1,700

AGCAATAAAGCCGATAACAAAATCTTTGTCGCTCTTCGCAATGTCAACAGTACCCTTAGTATATTCTCCAGTAGATAGGGAGCCCTTGCATGACAATTCTGCTAACA
TCGTTATTTCGGCTATTGTTTTAGAAACAGCGAGAAGCGTTACAGTTGTCATGGGAATCATATAAGAGGTCATCTATCCCTCGGGAACGTACTGTTAAGACGATTGT
URA3
1,720 1,740 1,760 1,780 1,800

TCAAAAGGCCTCTAGGTTCCTTTGTTACTTCTTCTGCCGCCTGCTTCAAACCGCTAACAATACCTGGGCCCACCACACCGTGTGCATTCGTAATGTCTGCCCATTCT
AGTTTTCCGGAGATCCAAGGAAACAATGAAGAAGACGGCGGACGAAGTTTGGCGATTGTTATGGACCCGGGTGGTGTGGCACACGTAAGCATTACAGACGGGTAAGA
URA3
1,820 1,840 1,860 1,880 1,900 1,920

GCTATTCTGTATACACCCGCAGAGTACTGCAATTTGACTGTATTACCAATGTCAGCAAATTTTCTGTCTTCGAAGAGTAAAAAATTGTACTTGGCGGATAATGCCTT
CGATAAGACATATGTGGGCGTCTCATGACGTTAAACTGACATAATGGTTACAGTCGTTTAAAAGACAGAAGCTTCTCATTTTTTAACATGAACCGCCTATTACGGAA
URA3
1,940 1,960 1,980 2,000 2,020

TAGCGGCTTAACTGTGCCCTCCATGGAAAAATCAGTCAAGATATCCACATGTGTTTTTAGTAAACAAATTTTGGGACCTAATGCTTCAACTAACTCCAGTAATTCCT
ATCGCCGAATTGACACGGGAGGTACCTTTTTAGTCAGTTCTATAGGTGTACACAAAAATCATTTGTTTAAAACCCTGGATTACGAAGTTGATTGAGGTCATTAAGGA
URA3
2,040 2,060 2,080 2,100 2,120 2,140

TGGTGGTACGAACATCCAATGAAGCACACAAGTTTGTTTGCTTTTCGTGCATGATATTAAATAGCTTGGCAGCAACAGGACTAGGATGAGTAGCAGCACGTTCCTTA
ACCACCATGCTTGTAGGTTACTTCGTGTGTTCAAACAAACGAAAAGCACGTACTATAATTTATCGAACCGTCGTTGTCCTGATCCTACTCATCGTCGTGCAAGGAAT
URA3
2,160 2,180 2,200 2,220 2,240

TATGTAGCTTTCGACATGATTTATCTTCGTTTCCTGCATGTTTTTGTTCTGTGCAGTTGGGTTAAGAATACTGGGCAATTTCATGTTTCTTCAACACTACATATGCG
ATACATCGAAAGCTGTACTAAATAGAAGCAAAGGACGTACAAAAACAAGACACGTCAACCCAATTCTTATGACCCGTTAAAGTACAAAGAAGTTGTGATGTATACGC
URA3
2,260 2,280 2,300 2,320 2,340

TATATATACCAATCTAAGTCTGTGCTCCTTCCTTCGTTCTTCCTTCTGTTCGGAGATTACCGAATCAAAAAAATTTCAAAGAAACCGAAATCAAAAAAAAGAATAAA
ATATATATGGTTAGATTCAGACACGAGGAAGGAAGCAAGAAGGAAGACAAGCCTCTAATGGCTTAGTTTTTTTAAAGTTTCTTTGGCTTTAGTTTTTTTTCTTATTT
URA3
2,360 2,380 2,400 2,420 2,440 2,460

AAAAAAATGATGAATTGAATTGAAAAGCTAATTCTTGAAGACGAAAGGGCCTCGTGATACGCCTATTTTTATAGGTTAATGTCATGATAATAATGGTTTCTTAGACG
TTTTTTTACTACTTAACTTAACTTTTCGATTAAGAACTTCTGCTTTCCCGGAGCACTATGCGGATAAAAATATCCAATTACAGTACTATTATTACCAAAGAATCTGC
URA3
2,480 2,500 2,520 2,540 2,560

TCAGGTGGCACTTTTCGGGGAAATGTGCGCGGAACCCCTATTTGTTTATTTTTCTAAATACATTCAAATATGTATCCGCTCATGAGACAATAACCCTGATAAATGCT
AGTCCACCGTGAAAAGCCCCTTTACACGCGCCTTGGGGATAAACAAATAAAAAGATTTATGTAAGTTTATACATAGGCGAGTACTCTGTTATTGGGACTATTTACGA
2,580 2,600 2,620 2,640 2,660

TCAATAATATTGAAAAAGGAAGAGTATGAGTATTCAACATTTCCGTGTCGCCCTTATTCCCTTTTTTGCGGCATTTTGCCTTCCTGTTTTTGCTCACCCAGAAACGC
AGTTATTATAACTTTTTCCTTCTCATACTCATAAGTTGTAAAGGCACAGCGGGAATAAGGGAAAAAACGCCGTAAAACGGAAGGACAAAAACGAGTGGGTCTTTGCG

2,680 2,700 2,720 2,740 2,760 2,780

TGGTGAAAGTAAAAGATGCTGAAGATCAGTTGGGTGCACGAGTGGGTTACATCGAACTGGATCTCAACAGCGGTAAGATCCTTGAGAGTTTTCGCCCCGAAGAACGT
ACCACTTTCATTTTCTACGACTTCTAGTCAACCCACGTGCTCACCCAATGTAGCTTGACCTAGAGTTGTCGCCATTCTAGGAACTCTCAAAAGCGGGGCTTCTTGCA

2,800 2,820 2,840 2,860 2,880

TTTCCAATGATGAGCACTTTTAAAGTTCTGCTATGTGGCGCGGTATTATCCCGTATTGACGCCGGGCAAGAGCAACTCGGTCGCCGCATACACTATTCTCAGAATGA
AAAGGTTACTACTCGTGAAAATTTCAAGACGATACACCGCGCCATAATAGGGCATAACTGCGGCCCGTTCTCGTTGAGCCAGCGGCGTATGTGATAAGAGTCTTACT

AmpR

2,900 2,920 2,940 2,960 2,980

CTTGGTTGAGTACTCACCAGTCACAGAAAAGCATCTTACGGATGGCATGACAGTAAGAGAATTATGCAGTGCTGCCATAACCATGAGTGATAACACTGCGGCCAACT
GAACCAACTCATGAGTGGTCAGTGTCTTTTCGTAGAATGCCTACCGTACTGTCATTCTCTTAATACGTCACGACGGTATTGGTACTCACTATTGTGACGCCGGTTGA

AmpR

3,000 3,020 3,040 3,060 3,080 3,100

TACTTCTGACAACGATCGGAGGACCGAAGGAGCTAACCGCTTTTTTGCACAACATGGGGGATCATGTAACTCGCCTTGATCGTTGGGAACCGGAGCTGAATGAAGCC
ATGAAGACTGTTGCTAGCCTCCTGGCTTCCTCGATTGGCGAAAAAACGTGTTGTACCCCCTAGTACATTGAGCGGAACTAGCAACCCTTGGCCTCGACTTACTTCGG

AmpR

3,120 3,140 3,160 3,180 3,200

ATACCAAACGACGAGCGTGACACCACGATGCCTGTAGCAATGGCAACAACGTTGCGCAAACTATTAACTGGCGAACTACTTACTCTAGCTTCCCGGCAACAATTAAT
TATGGTTTGCTGCTCGCACTGTGGTGCTACGGACATCGTTACCGTTGTTGCAACGCGTTTGATAATTGACCGCTTGATGAATGAGATCGAAGGGCCGTTGTTAATTA

AmpR

3,220 3,240 3,260 3,280 3,300

AGACTGGATGGAGGCGGATAAAGTTGCAGGACCACTTCTGCGCTCGGCCCTTCCGGCTGGCTGGTTTATTGCTGATAAATCTGGAGCCGGTGAGCGTGGGTCTCGCG
TCTGACCTACCTCCGCCTATTTCAACGTCCTGGTGAAGACGCGAGCCGGGAAGGCCGACCGACCAAATAACGACTATTTAGACCTCGGCCACTCGCACCCAGAGCGC

AmpR

3,320 3,340 3,360 3,380 3,400 3,420

GTATCATTGCAGCACTGGGGCCAGATGGTAAGCCCTCCCGTATCGTAGTTATCTACACGACGGGGAGTCAGGCAACTATGGATGAACGAAATAGACAGATCGCTGAG
CATAGTAACGTCGTGACCCCGGTCTACCATTCGGGAGGGCATAGCATCAATAGATGTGCTGCCCCTCAGTCCGTTGATACCTACTTGCTTTATCTGTCTAGCGACTC

AmpR

3,440 3,460 3,480 3,500 3,520

ATAGGTGCCTCACTGATTAAGCATTGGTAACTGTCAGACCAAGTTTACTCATATATACTTTAGATTGATTTAAAACTTCATTTTTAATTTAAAAGGATCTAGGTGAA
TATCCACGGAGTGACTAATTCGTAACCATTGACAGTCTGGTTCAAATGAGTATATATGAAATCTAACTAAATTTTGAAGTAAAAATTAAATTTTCCTAGATCCACTT

AmpR

3,540 3,560 3,580 3,600 3,620

GATCCTTTTTGATAATCTCATGACCAAAATCCCTTAACGTGAGTTTTCGTTCCACTGAGCGTCAGACCCCGTAGAAAAGATCAAAGGATCTTCTTGAGATCCTTTTT
CTAGGAAAAACTATTAGAGTACTGGTTTTAGGGAATTGCACTCAAAAGCAAGGTGACTCGCAGTCTGGGGCATCTTTTCTAGTTTCCTAGAAGAACTCTAGGAAAAA

ColE1 origin

3,640 3,660 3,680 3,700 3,720 3,740

TTCTGCGCGTAATCTGCTGCTTGCAAACAAAAAAACCACCGCTACCAGCGGTGGTTTGTTTGCCGGATCAAGAGCTACCAACTCTTTTTCCGAAGGTAACTGGCTTC
AAGACGCGCATTAGACGACGAACGTTTGTTTTTTTGGTGGCGATGGTCGCCACCAAACAAACGGCCTAGTTCTCGATGGTTGAGAAAAAGGCTTCCATTGACCGAAG

ColE1 origin

3,760 3,780 3,800 3,820 3,840

AGCAGAGCGCAGATACCAAATACTGTCCTTCTAGTGTAGCCGTAGTTAGGCCACCACTTCAAGAACTCTGTAGCACCGCCTACATACCTCGCTCTGCTAATCCTGTT
TCGTCTCGCGTCTATGGTTTATGACAGGAAGATCACATCGGCATCAATCCGGTGGTGAAGTTCTTGAGACATCGTGGCGGATGTATGGAGCGAGACGATTAGGACAA

ColE1 origin

3,860 3,880 3,900 3,920 3,940

ACCAGTGGCTGCTGCCAGTGGCGATAAGTCGTGTCTTACCGGGTTGGACTCAAGACGATAGTTACCGGATAAGGCGCAGCGGTCGGGCTGAACGGGGGGTTCGTGCA
TGGTCACCGACGACGGTCACCGCTATTCAGCACAGAATGGCCCAACCTGAGTTCTGCTATCAATGGCCTATTCCGCGTCGCCAGCCCGACTTGCCCCCCAAGCACGT

ColE1 origin

3,960 3,980 4,000 4,020 4,040 4,060

CACAGCCCAGCTTGGAGCGAACGACCTACACCGAACTGAGATACCTACAGCGTGAGCTATGAGAAAGCGCCACGCTTCCCGAAGGGAGAAAGGCGGACAGGTATCCG
GTGTCGGGTCGAACCTCGCTTGCTGGATGTGGCTTGACTCTATGGATGTCGCACTCGATACTCTTTCGCGGTGCGAAGGGCTTCCCTCTTTCCGCCTGTCCATAGGC

ColE1 origin

4,080 4,100 4,120 4,140 4,160

GTAAGCGGCAGGGTCGGAACAGGAGAGCGCACGAGGGAGCTTCCAGGGGGAAACGCCTGGTATCTTTATAGTCCTGTCGGGTTTCGCCACCTCTGACTTGAGCGTCG
CATTCGCCGTCCCAGCCTTGTCCTCTCGCGTGCTCCCTCGAAGGTCCCCCTTTGCGGACCATAGAAATATCAGGACAGCCCAAAGCGGTGGAGACTGAACTCGCAGC

ColE1 origin

4,180 4,200 4,220 4,240 4,260 4,280

ATTTTTGTGATGCTCGTCAGGGGGGCGGAGCCTATGGAAAAACGCCAGCAACGCGGCCTTTTTACGGTTCCTGGCCTTTTGCTGGCCTTTTGCTCACATGTTCTTTC
TAAAAACACTACGAGCAGTCCCCCCGCCTCGGATACCTTTTTGCGGTCGTTGCGCCGGAAAAATGCCAAGGACCGGAAAACGACCGGAAAACGAGTGTACAAGAAAG

ColE1 origin

4,300 4,320 4,340 4,360 4,380

CTGCGTTATCCCCTGATTCTGTGGATAACCGTATTACCGCCTTTGAGTGAGCTGATACCGCTCGCCGCAGCCGAACGACCGAGCGCAGCGAGTCAGTGAGCGAGGAA
GACGCAATAGGGGACTAAGACACCTATTGGCATAATGGCGGAAACTCACTCGACTATGGCGAGCGGCGTCGGCTTGCTGGCTCGCGTCGCTCAGTCACTCGCTCCTT

4,400 4,420 4,440 4,460 4,480

GCGGAAGAGCGCCCAATACGCAAACCGCCTCTCCCCGCGCGTTGGCCGATTCATTAATGCAGCTGGCACGACAGGTTTCCCGACTGGAAAGCGGGCAGTGAGCGCAA
CGCCTTCTCGCGGGTTATGCGTTTGGCGGAGAGGGGCGCGCAACCGGCTAAGTAATTACGTCGACCGTGCTGTCCAAAGGGCTGACCTTTCGCCCGTCACTCGCGTT

4,500 4,520 4,540 4,560 4,580 4,600

CGCAATTAATGTGAGTTAGCTCACTCATTAGGCACCCCAGGCTTTACACTTTATGCTTCCGGCTCGTATGTTGTGTGGAATTGTGAGCGGATAACAATTTCACACAG
GCGTTAATTACACTCAATCGAGTGAGTAATCCGTGGGGTCCGAAATGTGAAATACGAAGGCCGAGCATACAACACACCTTAACACTCGCCTATTGTTAAAGTGTGTC

lac promoter LacO

4,620 4,640 4,660 4,680 4,700

GAAACAGCTATGACCATGATTACGCCAAGCTTACCAGTTCTCACACGGAACACCACTAATGGACACAAATTCGAAATACTTTGACCCTATTTTCGAGGACCTTGTCA
CTTTGTCGATACTGGTACTAATGCGGTTCGAATGGTCAAGAGTGTGCCTTGTGGTGATTACCTGTGTTTAAGCTTTATGAAACTGGGATAAAAGCTCCTGGAACAGT

M13-rev GAPDH promoter

4,720 4,740 4,760 4,780 4,800

CCTTGAGCCCAAGAGAGCCAAGATTTAAATTTTCCTATGACTTGATGCAAATTCCCAAAGCTAATAACATGCAAGACACGTACGGTCAAGAAGACATATTTGACCTC
GGAACTCGGGTTCTCTCGGTTCTAAATTTAAAAGGATACTGAACTACGTTTAAGGGTTTCGATTATTGTACGTTCTGTGCATGCCAGTTCTTCTGTATAAACTGGAG

GAPDH promoter

4,820 4,840 4,860 4,880 4,900 4,920

TTAACAGGTTCAGACGCGACTGCCTCATCAGTAAGACCCGTTGAAAAGAACTTACCTGAAAAAAACGAATATATACTAGCGTTGAATGTTAGCGTCAACAACAAGAA
AATTGTCCAAGTCTGCGCTGACGGAGTAGTCATTCTGGGCAACTTTTCTTGAATGGACTTTTTTTGCTTATATATGATCGCAACTTACAATCGCAGTTGTTGTTCTT

GAPDH promoter

4,940 4,960 4,980 5,000 5,020

GTTTAATGACGCGGAGGCCAAGGCAAAAAGATTCCTTGATTACGTAAGGGAGTTAGAATCATTTTGAATAAAAAACACGCTTTTTCAGTTCGAGTTTATCATTATCA
CAAATTACTGCGCCTCCGGTTCCGTTTTTCTAAGGAACTAATGCATTCCCTCAATCTTAGTAAAACTTATTTTTTGTGCGAAAAAGTCAAGCTCAAATAGTAATAGT

GAPDH promoter

5,040 5,060 5,080 5,100 5,120

ATACTGCCATTTCAAAGAATACGTAAATAATTAATAGTAGTGATTTTCCTAACTTTATTTAGTCAAAAAATTAGCCTTTTAATTCTGCTGTAACCCGTACATGCCCA
TATGACGGTAAAGTTTCTTATGCATTTATTAATTATCATCACTAAAAGGATTGAAATAAATCAGTTTTTTAATCGGAAAATTAAGACGACATTGGGCATGTACGGGT

GAPDH promoter

5,140 5,160 5,180 5,200 5,220 5,240

AAATAGGGGGCGGGTTACACAGAATATATAACATCGTAGGTGTCTGGGTGAACAGTTTATTCCTGGCATCCACTAAATATAATGGAGCCCGCTTTTTAAGCTGGCAT
TTTATCCCCCGCCCAATGTGTCTTATATATTGTAGCATCCACAGACCCACTTGTCAAATAAGGACCGTAGGTGATTTATATTACCTCGGGCGAAAAATTCGACCGTA

GAPDH promoter

5,260 5,280 5,300 5,320 5,340

CCAGAAAAAAAAAGAATCCCAGCACCAAAATATTGTTTTCTTCACCAACCATCAGTTCATAGGTCCATTCTCTTAGCGCAACTACAGAGAACAGGGGCACAAACAGG
GGTCTTTTTTTTTCTTAGGGTCGTGGTTTTATAACAAAAGAAGTGGTTGGTAGTCAAGTATCCAGGTAAGAGAATCGCGTTGATGTCTCTTGTCCCCGTGTTTGTCC

GAPDH promoter

5,360 5,380 5,400 5,420 5,440

CAAAAAACGGGCACAACCTCAATGGAGTGATGCAACCTGCCTGGAGTAAATGATGACACAAGGCAATTGACCCACGCATGTATCTATCTCATTTTCTTACACCTTCT
GTTTTTTGCCCGTGTTGGAGTTACCTCACTACGTTGGACGGACCTCATTTACTACTGTGTTCCGTTAACTGGGTGCGTACATAGATAGAGTAAAAGAATGTGGAAGA
GAPDH promoter
5,460 5,480 5,500 5,520 5,540 5,560

ATTACCTTCTGCTCTCTCTGATTTGGAAAAAGCTGAAAAAAAAGGTTGAAACCAGTTCCCTGAAATTATTCCCCTACTTGACTAATAAGTATATAAAGACGGTAGGT
TAATGGAAGACGAGAGAGACTAAACCTTTTTCGACTTTTTTTTCCAACTTTGGTCAAGGGACTTTAATAAGGGGATGAACTGATTATTCATATATTTCTGCCATCCA
GAPDH promoter
5,580 5,600 5,620 5,640 5,660

ATTGATTGTAATTCTGTAAATCTATTTCTTAAACTTCTTAAATTCTACTTTTATAGTTAGTCTTTTTTTTAGTTTTAAAACACCAAGAACTTAGTTTCGAATAAACA
TAACTAACATTAAGACATTTAGATAAAGAATTTGAAGAATTTAAGATGAAAATATCAATCAGAAAAAAAATCAAAATTTTGTGGTTCTTGAATCAAAGCTTATTTGT
GAPDH promoter
5,680 5,700 5,720 5,740 5,760

CACATAAACAGAATTCATGAGATTCCCATCTATCTTCACCGCTGTTTTGTTCGCTGCTTCTTCTGCTTTGGCTGCTCCAGCTAACACCACCACCGAAGACGAAACCG
GTGTATTTGTCTTAAGTACTCTAAGGGTAGATAGAAGTGGCGACAAAACAAGCGACGAAGAAGACGAAACCGACGAGGTCGATTGTGGTGGTGGCTTCTGCTTTGGC
1 2 3 4 5 6 7 8 9 10 11 12 13 14 15 16 17 18 19 20 21 22 23 24 25 26 27 28 29 30
M R F P S I F T A V L F A A S S A L A A P A N T T T E D E T
Mating factor alpha leader
5,780 5,800 5,820 5,840 5,860 5,880

CTCAAATCCCAGCTGAAGCTGTTATCGACTACTCTGACTTGGAAGGTGACTTCGACGCTGCTGCTTTGCCATTGTCTAACTCTACCAACAACGGTTTGTCTTCTACC
GAGTTTAGGGTCGACTTCGACAATAGCTGATGAGACTGAACCTTCCACTGAAGCTGCGACGACGAAACGGTAACAGATTGAGATGGTTGTTGCCAAACAGAAGATGG
31 32 33 34 35 36 37 38 39 40 41 42 43 44 45 46 47 48 49 50 51 52 53 54 55 56 57 58 59 60 61 62 63 64 65 66
A Q I P A E A V I D Y S D L E G D F D A A A L P L S N S T N N G L S S T
Mating factor alpha leader
5,900 5,920 5,940 5,960 5,980

AACACCACCATCGCTTCTATCGCTGCTAAGGAAGAAGGTGTTCAATTGGACAAGAGAGAAGCTAGCGCAGATGTGCAGTTGCAAGCATCCGGCGGCGGTTCTGTTCA
TTGTGGTGGTAGCGAAGATAGCGACGATTCCTTCTTCCACAAGTTAACCTGTTCTCTCTTCGATCGCGTCTACACGTCAACGTTCGTAGGCCGCCGCCAAGACAAGT
67 68 69 70 71 72 73 74 75 76 77 78 79 80 81 82 83 84 85 86
N T T I A S I A A K E E G V Q L D K R E
2 4 6 8 10 12
D V Q L Q A S G G G S V Q
Mating factor alpha leader
anti-lys Nb
6,000 6,020 6,040 6,060 6,080

GGCTGGTGGTTCTCTAAGATTAAGTTGTGCTGCTTCAGGTTATACCATCGGCCCATACTGCATGGGTTGGTTTCGTCAAGCTCCAGGCAAAGAAAGGGAGGGAGTCG
CCGACCACCAAGAGATTCTAATTCAACACGACGAAGTCCAATATGGTAGCCGGGTATGACGTACCCAACCAAAGCAGTTCGAGGTCCGTTTCTTTCCCTCCCTCAGC
14 16 18 20 22 24 26 28 30 32 34 36 38 40 42 44 46 48
A G G S L R L S C A A S G Y T I G P Y C M G W F R Q A P G K E R E G V
anti-lys Nb
6,100 6,120 6,140 6,160 6,180 6,200

CAGCCATTAACATGGGCGGCGGTATCACATATTATGCAGACTCCGTAAAAGGTAGATTTACTATCTCTCAGGACAATGCTAAGAACACGGTCTATCTGTTAATGAAT
GTCGGTAATTGTACCCGCCGCCATAGTGTATAATACGTCTGAGGCATTTTCCATCTAAATGATAGAGAGTCCTGTTACGATTCTTGTGCCAGATAGACAATTACTTA
50 52 54 56 58 60 62 64 66 68 70 72 74 76 78 80 82 84
A A I N M G G G I T Y Y A D S V K G R F T I S Q D N A K N T V Y L L M N
anti-lys Nb
6,220 6,240 6,260 6,280 6,300

TCATTGGAGCCCGAGGACACTGCCATATACTACTGTGCCGCAGATTCAACCATTTACGCTTCCTATTACGAATGCGGACACGGATTAAGTACTGGAGGTTACGGTTA
AGTAACCTCGGGCTCCTGTGACGGTATATGATGACACGGCGTCTAAGTTGGTAAATGCGAAGGATAATGCTTACGCCTGTGCCTAATTCATGACCTCCAATGCCAAT
86 88 90 92 94 96 98 100 102 104 106 108 110 112 114 116 118 120
S L E P E D T A I Y Y C A A D S T I Y A S Y Y E C G H G L S T G G Y G Y
anti-lys Nb
6,320 6,340 6,360 6,380 6,400 6,420

TGATTCCTGGGGTCAAGGCACACAAGTCACGGTATCATCAGGATCCTACCCATACGATGTTCCAGATTACGCTCAAACATCCTCACCCTCTACAGAAGCATCAGTAT
ACTAAGGACCCCAGTTCCGTGTGTTCAGTGCCATAGTAGTCCTAGGATGGGTATGCTACAAGGTCTAATGCGAGTTTGTAGGAGTGGGAGATGTCTTCGTAGTCATA
122 124 126 128 130 132 1 2 3 4 5 6 7 8 9 2 4 6 8 10
D S W G Q G T Q V T V S S Y P Y D V P D Y A Q T S S P S T E A S V
anti-lys Nb HA tag 649 stalk
6,440 6,460 6,480 6,500 6,520

CAACCTCTAGTACCTCTTCCTCTGCTTCACAATCATCTGACCCAACTACAACATCTTCGTCCAGTTCATCGTCTTCCCCATCTTCCCAATCTGAGGAAATTTCATCA
GTTGGAGATCATGGAGAAGGAGACGAAGTGTTAGTAGACTGGGTTGATGTTGTAGAAGCAGGTCAAGTAGCAGAAGGGGTAGAAGGGTTAGACTCCTTTAAAGTAGT
12 14 16 18 20 22 24 26 28 30 32 34 36 38 40 42 44 46
S T S S T S S S A S Q S S D P T T T S S S S S S S S P S S Q S E E I S S
649 stalk
6,540 6,560 6,580 6,600 6,620

TCACCGACGGTCTCAACTACACCATCAACTTCTTCATCATCTTCCTCAATGACTTCAACCACCACAACAAAGTCAATCTCAACTTCCACTACAAGTTCAGCTCCAGT
AGTGGCTGCCAGAGTTGATGTGGTAGTTGAAGAAGTAGTAGAAGGAGTTACTGAAGTTGGTGGTGTTGTTTCAGTTAGAGTTGAAGGTGATGTTCAAGTCGAGGTCA
48 50 52 54 56 58 60 62 64 66 68 70 72 74 76 78 80 82
S P T V S T T P S T S S S S S S M T S T T T T K S I S T S T T S S A P V
649 stalk
6,640 6,660 6,680 6,700 6,720 6,740

TACAGATGTGACAGTTTCCTCATCGCCTAGTAAATCTACCTCTACTTCGACAAGTACAGAAACATCTAAAACACCTACTTCAATGACAGAGTATACATCTAGTACAT
ATGTCTACACTGTCAAAGGAGTAGCGGATCATTTAGATGGAGATGAAGCTGTTCATGTCTTTGTAGATTTTGTGGATGAAGTTACTGTCTCATATGTAGATCATGTA
84 86 88 90 92 94 96 98 100 102 104 106 108 110 112 114 116 118
T D V T V S S S P S K S T S T S T S T E T S K T P T S M T E Y T S S T
649 stalk
6,760 6,780 6,800 6,820 6,840

CGATAATTTCGACTCCAGTTAGTCACTCGCAGACAGGTTTGTCGGCTTCATCAAGTTCATCATCTACAACATCCGGTTCTTCGTCCACTAAATCAGAAAGTTCGACA
GCTATTAAAGCTGAGGTCAATCAGTGAGCGTCTGTCCAAACAGCCGAAGTAGTTCAAGTAGTAGATGTTGTAGGCCAAGAAGCAGGTGATTTAGTCTTTCAAGCTGT
120 122 124 126 128 130 132 134 136 138 140 142 144 146 148 150 152 154
S I I S T P V S H S Q T G L S A S S S S S S T T S G S S S T K S E S S T

649 stalk

6,860 6,880 6,900 6,920 6,940

ACATCTGGCTCTTCCCAGTCCGTGGAATCAACCTCCAGCCACGCCACTGTTCTTGCTAATTCCGCAGAAATGGTCACAACATCCTCTAGTTCATCCTCAACATCCGA
TGTAGACCGAGAAGGGTCAGGCACCTTAGTTGGAGGTCGGTGCGGTGACAAGAACGATTAAGGCGTCTTTACCAGTGTTGTAGGAGATCAAGTAGGAGTTGTAGGCT
156 158 160 162 164 166 168 170 172 174 176 178 180 182 184 186 188 190
T S G S S Q S V E S T S S H A T V L A N S A E M V T T S S S S S S T S E

649 stalk

6,960 6,980 7,000 7,020 7,040 7,060

AATGTCATTAACTAGTACTGCTACCAGTGTACCAGTCTCATCTAGTAGCAGTACGACATATTCTACTAGCGCATCTACACAAGCCGTCACTACAACATCTTCTTCCA
TTACAGTAATTGATCATGACGATGGTCACATGGTCAGAGTAGATCATCGTCATGCTGTATAAGATGATCGCGTAGATGTGTTCGGCAGTGATGTTGTAGAAGAAGGT
192 194 196 198 200 202 204 206 208 210 212 214 216 218 220 222 224
M S L T S T A T S V P V S S S S S T T Y S T S A S T Q A V T T T S S S

649 stalk

7,080 7,100 7,120 7,140 7,160

CTGTATCTACAACTTCTTCTAGTACAACGTTAACAAGCGCATTCACACATTCTTCAACCACATCGTCCGACCAGCCACCCAGCGACACTACAAGTCCATCTACGACA
GACATAGATGTTGAAGAAGATCATGTTGCAATTGTTCGCGTAAGTGTGTAAGAAGTTGGTGTAGCAGGCTGGTCGGTGGGTCGCTGTGATGTTCAGGTAGATGCTGT
226 228 230 232 234 236 238 240 242 244 246 248 250 252 254 256 258 260
T V S T T S S S T T L T S A F T H S S T T S S D Q P P S D T T S P S T T

649 stalk

7,180 7,200 7,220 7,240 7,260

CACGAACCTCATGTAACCACTCAGACGTCATCAGAAACATCTTCTTCTAAGTCATCTTCTACTTCTTCTTCAAGTACATCTCAAACCTCTGAGTCTGCAACACCATC
GTGCTTGGAGTACATTGGTGAGTCTGCAGTAGTCTTTGTAGAAGAAGATTCAGTAGAAGATGAAGAAGAAGTTCATGTAGAGTTTGGAGACTCAGACGTTGTGGTAG
262 264 266 268 270 272 274 276 278 280 282 284 286 288 290 292 294 296
H E P H V T T Q T S S E T S S S K S S S T S S S S T S Q T S E S A T P S

649 stalk

7,280 7,300 7,320 7,340 7,360 7,380

CGATTCCGTATCACCTGGAAGTTCTACATCAACATCTTCTAGTAGCACTTCTACTTCCACTTCTATTTCCAGTGGAGAAACGACAACTTCTTCTTCTTCATCATCTG
GCTAAGGCATAGTGGACCTTCAAGATGTAGTTGTAGAAGATCATCGTGAAGATGAAGGTGAAGATAAAGGTCACCTCTTTGCTGTTGAAGAAGAAGAAGTAGTAGAC
298 300 302 304 306 308 310 312 314 316 318 320 322 324 326 328 330 332
D S V S P G S S T S T S S S S T S T S T S I S S G E T T T S S S S S S

649 stalk

7,400 7,420 7,440 7,460 7,480

CCACGACCACTTCTAACAGCGCAACCTTGTCAGTCTCTACCACACAAACTTCGATTGAAGCCAGTTCATCTACTACATCTACATCTAGTTCAACAATTACAACTTCA

GGTGCTGGTGAAGATTGTCGCGTTGGAACAGTCAGAGATGGTGTGTTTGAAGCTAACTTCGGTCAAGTAGATGATGTAGATGTAGATCAAGTTGTTAATGTTGAAGT

334 336 338 340 342 344 346 348 350 352 354 356 358 360 362 364 366 368

A T T T S N S A T L S V S T T Q T S I E A S S S T T S T S S S T I T T S

649 stalk

7,500 7,520 7,540 7,560 7,580

AGTAGTAGCGCTCACATATCGTCGAAATCTCAATCTAGTATTACCTATCCCTCTTCCTCGACATCTTCATCTACATCGTCCTCAATTTCTAGCGAATCTGAAAGTTT

TCATCATCGCGAGTGTATAGCAGCTTTAGAGTTAGATCATAATGGATAGGGAGAAGGAGCTGTAGAAGTAGATGTAGCAGGAGTTAAAGATCGCTTAGACTTTCAAA

370 372 374 376 378 380 382 384 386 388 390 392 394 396 398 400 402 404

S S S A H I S S K S Q S S I T Y P S S S T S S S T S S S I S S E S E S F

649 stalk

7,600 7,620 7,640 7,660 7,680 7,700

TGAATCGACATCAGCAGAAGATGCTCCATCAACAGCACCTTCATCAAGTGTCTCTTCTAAGAGTTCTACCTCTACAACATCAAGCACATCGACATCTTCAAGCACTC

ACTTAGCTGTAGTCGTCTTCTACGAGGTAGTTGTCGTGGAAGTAGTTCACAGAGAAGATTCTCAAGATGGAGATGTTGTAGTTCGTGTAGCTGTAGAAGTTCGTGAG

406 408 410 412 414 416 418 420 422 424 426 428 430 432 434 436 438

E S T S A E D A P S T A P S S S V S S K S S T S T T S S T S T S S S T

649 stalk

7,720 7,740 7,760 7,780 7,800

CATCTCCATCACCATCTTCCGTGAGTTCTTCCTCCACCAGCTCATTGACAACTTCTGCTGTATCAACACCAGCTACCTCTCATTCTCAAAGTACTGTAGTAACCACC

GTAGAGGTAGTGGTAGAAGGCACTCAAGAAGGAGGTGGTCGAGTAACTGTTGAAGACGACATAGTTGTGGTCGATGGAGAGTAAGAGTTTCATGACATCATTGGTGG

440 442 444 446 448 450 452 454 456 458 460 462 464 466 468 470 472 474

P S P S P S S V S S S S T S S L T T S A V S T P A T S H S Q S T V V T T

649 stalk

7,820 7,840 7,860 7,880 7,900

ACTACTATTACTACATCAACAGGTCCAGTGATGTCTACGACAACAGCTTATTCTTCTAGTTCTACTAGCAGCTCGGAATCTTCTGAGGTTCAGTCTGTCATGTCATC

TGATGATAATGATGTAGTTGTCCAGGTCACTACAGATGCTGTTGTCGAATAAGAAGATCAAGATGATCGTCGAGCCTTAGAAGACTCCAAGTCAGACAGTACAGTAG

476 478 480 482 484 486 488 490 492 494 496 498 500 502 504 506 508 510

T T I T T S T G P V M S T T T A Y S S S S T S S S E S S E V Q S V M S S

649 stalk

7,920 7,940 7,960 7,980 8,000 8,020

TACGCCTAGTTCAACATCAACAACAACCAGTTCGGAATCTACTTCATCTAGCTCCACAGCTTCTACCTCACCATCAACCTCGCAAACTTTCGAAACTTCTCCTACTA

ATGCGGATCAAGTTGTAGTTGTTGTTGGTCAAGCCTTAGATGAAGTAGATCGAGGTGTCGAAGATGGAGTGGTAGTTGGAGCGTTTGAAAGCTTTGAAGAGGATGAT

512 514 516 518 520 522 524 526 528 530 532 534 536 538 540 542 544 546

T P S S T S T T T S S E S T S S S S T A S T S P S T S Q T F E T S P T

649 stalk

8,040 8,060 8,080 8,100 8,120

```
TAGGAGGTGTCCCCTCAACCACTTCATTTGTCTCTACGCCAACAACGAAATTGTCGCACACTACTTCCACTATGACAGCACAGTCCGATAGTAAGTCTACCCACTCC
ATCCTCCACAGGGGAGTTGGTGAAGTAAACAGAGATGCGGTTGTTGCTTTAACAGCGTGTGATGAAGGTGATACTGTCGTGTCAGGCTATCATTCAGATGGGTGAGG
  548  550  552  554  556  558  560  562  564  566  568  570  572  574  576  578  580  582
 I G G V P S T T S F V S T P T T K L S H T T S T M T A Q S D S K S T H S
649 stalk
8,140  8,160  8,180  8,200  8,220
```

```
TCAAGCACATCGACAGAAGATAAATCATCCACTGCTTCTGCAGTTGACGAAAGCACTACAACATCCACTTCCACGGAGTCTACTACATCAGTAACATCAGGCACCTC
AGTTCGTGTAGCTGTCTTCTATTTAGTAGGTGACGAAGACGTCAACTGCTTTCGTGATGTTGTAGGTGAAGGTGCCTCAGATGATGTAGTCATTGTAGTCCGTGGAG
  584  586  588  590  592  594  596  598  600  602  604  606  608  610  612  614  616  618
 S S T S T E D K S S T A S A V D E S T T T S T S T E S T T S V T S G T S
649 stalk
8,240  8,260  8,280  8,300  8,320  8,340
```

```
CCATTCCGCTAAAGAATCTTCGTCAAATTCTAAGGTGTATAGTTCACAGACAGCACACTCATCCATAAGTGTTGCATCATCACCTAGTACAAAGGGCGCCCAAATCC
GGTAAGGCGATTTCTTAGAAGCAGTTTAAGATTCCACATATCAAGTGTCTGTCGTGTGAGTAGGTATTCACAACGTAGTAGTGGATCATGTTTCCCGCGGGTTTAGG
  620  622  624  626  628  630  632  634  636  638  640  642  644  646  648  650  652
 H S A K E S S S N S K V Y S S Q T A H S S I S V A S S P S T K G A Q I
649 stalk
8,360  8,380  8,400  8,420  8,440
```

```
AATCTTCTATGGTTGAAATCTCTACCTACGCTGGTTCTGCTAACTCTGTTAACGCTGGTGCTGGTGCTGGTGCTTTGTTCTTGTTGTTGTCTTTGGCTATCATCTAA
TTAGAAGATACCAACTTTAGAGATGGATGCGACCAAGACGATTGAGACAATTGCGACCACGACCACGACCACGAAACAAGAACAACAACAGAAACCGATAGTAGATT
654  656  658  660  662  664  666  668  670  672  674  676  678  680  682  684  686  688 1
 Q S S M V E I S T Y A G S A N S V N A G A G A G A L F L L L S L A I I *
GPI anchor
8,460  8,480  8,500  8,520  8,540  8,560
```

```
TGATTAATTAACTCGAGATCTGATAACAACAGTGTAGATGTAACAAAATCGACTTTGTTCCCACTGTACTTTTAGCTCGTACAAAATACAATATACTTTTCATTTCT
ACTAATTAATTGAGCTCTAGACTATTGTTGTCACATCTACATTGTTTTAGCTGAAACAAGGGTGACATGAAAATCGAGCATGTTTTATGTTATATGAAAAGTAAAGA
 2 3 4 5 6 7
 * L I N S R
alpha factor terminator
8,580  8,600  8,620  8,640  8,660
```

```
CCGTAAACAACATGTTTTCCCATGTAATATCCTTTTCTATTTTTCGTTCCGTTACCAACTTTACACATACTTTATATAGCTATTCACTTCTATACACTAAAAAACTA
GGCATTTGTTGTACAAAAGGGTACATTATAGGAAAAGATAAAAAGCAAGGCAATGGTTGAAATGTGTATGAAATATATCGATAAGTGAAGATATGTGATTTTTTGAT
alpha factor terminator
8,680  8,700  8,720  8,740  8,760
```

```
AGACAATTTTAATTTTGCTGCCTGCCATATTTCAATTTGTTATAAATTCCTATAATTTATCCTATTAGTAGCTAAAAAAAGATGAATGTGAATCGAATCCTAAGAGA
TCTGTTAAAATTAAAACGACGGACGGTATAAAGTTAAACAATATTTAAGGATATTAAATAGGATAATCATCGATTTTTTTCTACTTACACTTAGCTTAGGATTCTCT
alpha factor terminator
8,780  8,800  8,820  8,840  8,860  8,880
```

ATTGGTACCGCGATGTAGTAAAACTAGCTAGACCGAGAAAGAGACTAGAAATGCAAAAGGCACTTCTACAATGGCTGCCATCATTATTATCCGATGTGACGCTGCAT
TAACCATGGCGCTACATCATTTTGATCGATCTGGCTCTTTCTCTGATCTTTACGTTTTCCGTGAAGATGTTACCGACGGTAGTAATAATAGGCTACACTGCGACGTA

8,900 8,920 8,940 8,960 8,980

TTTTTTTTTTTTTTTTTTTTTTTTTTTTTTTTTTTTTTTTTTTTTTGTACAAATATCATAAAAAAAGAGAATCTTTTTAAGCAAGGATTTTCTTAACTTCTTCGGCG
AAAAAAAAAAAAAAAAAAAAAAAAAAAAAAAAAAAAAAAAAAAAAACATGTTTATAGTATTTTTTTCTCTTAGAAAAATTCGTTCCTAAAAGAATTGAAGAAGCCGC

9,000 9,020 9,040 9,060 9,080

ACAGCATCACCGACTTCGGTGGTACTGTTGGAACCACCTAAATCACCAGTTCTGATACCTGCATCCAAAACCTTTTTAACTGCATCTTCAATGGCCTTACCTTCTTC
TGTCGTAGTGGCTGAAGCCACCATGACAACCTTGGTGGATTTAGTGGTCAAGACTATGGACGTAGGTTTTGGAAAAATTGACGTAGAAGTTACCGGAATGGAAGAAG

9,100 9,120 9,140 9,160 9,180 9,200

AGGCAAGTTCAATGACAATTTCAACATCATTGCAGCAGACAAGATAGTGGCGATAGGGTCAACCTTATTCTTTGGCAAATCTGGAGCAGAACCGTGGCATGGTTCGT
TCCGTTCAAGTTACTGTTAAAGTTGTAGTAACGTCGTCTGTTCTATCACCGCTATCCCAGTTGGAATAAGAAACCGTTTAGACCTCGTCTTGGCACCGTACCAAGCA

9,220 9,240 9,260 9,280 9,300

ACAAACCAAATGCGGTGTTCTTGTCTGGCAAAGAGGCCAAGGACGCAGATGGCAACAAACCCAAGGAACCTGGGATAACGGAGGCTTCATCGGAGATGATATCACCA
TGTTTGGTTTACGCCACAAGAACAGACCGTTTCTCCGGTTCCTGCGTCTACCGTTGTTTGGGTTCCTTGGACCCTATTGCCTCCGAAGTAGCCTCTACTATAGTGGT

9,320 9,340 9,360 9,380 9,400

AACATGTTGCTGGTGATTATAATACCATTTAGGTGGGTTGGGTTCTTAACTAGGATCATGGCGGCAGAATCAATCAATTGATGTTGAACCTTCAATGTAGGAAATTC
TTGTACAACGACCACTAATATTATGGTAAATCCACCCAACCCAAGAATTGATCCTAGTACCGCCGTCTTAGTTAGTTAACTACAACTTGGAAGTTACATCCTTTAAG

9,420 9,440 9,460 9,480 9,500 9,520

GTTCTTGATGGTTTCCTCCACAGTTTTTCTCCATAATCTTGAAGAGGCCAAAACATTAGCTTTATCCAAGGACCAAATAGGCAATGGTGGCTCATGTTGTAGGGCCA
CAAGAACTACCAAAGGAGGTGTCAAAAAGAGGTATTAGAACTTCTCCGGTTTTGTAATCGAAATAGGTTCCTGGTTTATCCGTTACCACCGAGTACAACATCCCGGT

9,540 9,560 9,580 9,600 9,620

TGAAAGCGGCCATTCTTGTGATTCTTTGCACTTCTGGAACGGTGTATTGTTCACTATCCCAAGCGACACCATCACCATCGTCTTCCTTTCTCTTACCAAAGTAAATA
ACTTTCGCCGGTAAGAACACTAAGAAACGTGAAGACCTTGCCACATAACAAGTGATAGGGTTCGCTGTGGTAGTGGTAGCAGAAGGAAAGAGAATGGTTTCATTTAT

9,640 9,660 9,680 9,700 9,720

CCTCCCACTAATTCTCTGACAACAACGAAGTCAGTACCTTTAGCAAATTGTGGCTTGATTGGAGATAAGTCTAAAAGAGAGTCGGATGCAAAGTTACATGGTCTTAA
GGAGGGTGATTAAGAGACTGTTGTTGCTTCAGTCATGGAAATCGTTTAACACCGAACTAACCTCTATTCAGATTTTCTCTCAGCCTACGTTTCAATGTACCAGAATT

9,740 9,760 9,780 9,800 9,820 9,840

GTTGGCGTACAATTGAAGTTCTTTACGGATTTTTAGTAAACCTTGTTCAGGTCTAACACTACCTGTACCCCATTTAGGACCACCCACAGCACCTAACAAAACGGCAT
CAACCGCATGTTAACTTCAAGAAATGCCTAAAAATCATTTGGAACAAGTCCAGATTGTGATGGACATGGGGTAAATCCTGGTGGGTGTCGTGGATTGTTTTGCCGTA

9,860 9,880 9,900 9,920 9,940

CAACCTTCTTGGAGGCTTCCAGCGCCTCATCTGGAAGTGGGACACCTGTAGCATCGATAGCAGCACCACCAATTAAATGATTTTCGAAATCGAACTTGACATTGGAA
GTTGGAAGAACCTCCGAAGGTCGCGGAGTAGACCTTCACCCTGTGGACATCGTAGCTATCGTCGTGGTGGTTAATTTACTAAAAGCTTTAGCTTGAACTGTAACCTT

9,960 9,980 10,000 10,020 10,040

CGAACATCAGAAATAGCTTTAAGAACCTTAATGGCTTCGGCTGTGATTTCTTGACCAACGTGGTCACCTGGCAAAACGACGATCTTCTTAGGGGCAGACATTACAAT
GCTTGTAGTCTTTATCGAAATTCTTGGAATTACCGAAGCCGACACTAAAGAACTGGTTGCACCAGTGGACCGTTTTGCTGCTAGAAGAATCCCCGTCTGTAATGTTA

10,060 10,080 10,100 10,120 10,140 10,160

GGTATATCCTTGAAATATATATAAAAAAAAAAAAAAAAAAAAAAAAAAAAAAAATGCAGCTTCTCAATGATATTCGAATACGCTTTGAGGAGATACAGCCTAATATCC
CCATATAGGAACTTTATATATATTTTTTTTTTTTTTTTTTTTTTTTTTTTTTTTACGTCGAAGAGTTACTATAAGCTTATGCGAAACTCCTCTATGTCGGATTATAGG

10,180 10,200 10,220 10,240 10,260

GACAAACTGTTTTACAGATTTACGATCGTACTTGTTACCCATCATTGAATTTTGAACATCCGAACCTGGGAGTTTTCCCTGAAACAGATAGTATATTTGAACCTGTA
CTGTTTGACAAAATGTCTAAATGCTAGCATGAACAATGGGTAGTAACTTAAAACTTGTAGGCTTGGACCCTCAAAAGGGACTTTGTCTATCATATAAACTTGGACAT

10,280 10,300 10,320 10,340 10,360

TAATAATATATAGTCTAGCGCTTTACGGAAGACAATGTATGTATTTCGGTTCCTGGAGAAACTATTGCATCTATTGCATAGGTAATCTTGCACGTCGCATCCCCGGT
ATTATTATATATCAGATCGCGAAATGCCTTCTGTTACATACATAAAGCCAAGGACCTCTTTGATAACGTAGATAACGTATCCATTAGAACGTGCAGCGTAGGGGCCA

10,400 10,420 10,440 10,460 10,480

TCATTTTCTGCGTTTCCATCTTGCACTTCAATAGCATATCTTT
AGTAAAAGACGCAAAGGTAGAACGTGAAGTTATCGTATAGAAA

10,490 10,500 10,510 10,520

System 3 control strain (10127 bp)

GTTAACGAAGCATCTGTGCTTCATTTTGTAGAACAAAAATGCAACGCGAGAGCGCTAATTTTTCAAACAAAGAATCTGAGCTGCATTTTTACAGAACAGAAATGCAA
CAATTGCTTCGTAGACACGAAGTAAAACATCTTGTTTTTACGTTGCGCTCTCGCGATTAAAAAGTTTGTTTCTTAGACTCGACGTAAAAATGTCTTGTCTTTACGTT
2 micron origin
20 40 60 80 100

CGCGAAAGCGCTATTTTACCAACGAAGAATCTGTGCTTCATTTTTGTAAAACAAAAATGCAACGCGAGAGCGCTAATTTTTCAAACAAAGAATCTGAGCTGCATTTT
GCGCTTTCGCGATAAAATGGTTGCTTCTTAGACACGAAGTAAAAACATTTTTGTTTTTACGTTGCGCTCTCGCGATTAAAAAGTTTGTTTCTTAGACTCGACGTAAAA
2 micron origin
120 140 160 180 200

TACAGAACAGAAATGCAACGCGAGAGCGCTATTTTACCAACAAAGAATCTATACTTCTTTTTTGTTCTACAAAAATGCATCCCGAGAGCGCTATTTTTCTAACAAAG
ATGTCTTGTCTTTACGTTGCGCTCTCGCGATAAAATGGTTGTTTCTTAGATATGAAGAAAAAACAAGATGTTTTTACGTAGGGCTCTCGCGATAAAAAGATTGTTTC
2 micron origin
220 240 260 280 300 320

CATCTTAGATTACTTTTTTTCTCCTTTGTGCGCTCTATAATGCAGTCTCTTGATAACTTTTTGCACTGTAGGTCCGTTAAGGTTAGAAGAAGGCTACTTTGGTGTCT
GTAGAATCTAATGAAAAAAAGAGGAAACACGCGAGATATTACGTCAGAGAACTATTGAAAAACGTGACATCCAGGCAATTCCAATCTTCTTCCGATGAAACCACAGA
2 micron origin
340 360 380 400 420

ATTTTCTCTTCCATAAAAAAAGCCTGACTCCACTTCCCGCGTTTACTGATTACTAGCGAAGCTGCGGGTGCATTTTTTCAAGATAAAGGCATCCCCGATTATATTCT
TAAAAGAGAAGGTATTTTTTTCGGACTGAGGTGAAGGGCGCAAATGACTAATGATCGCTTCGACGCCCACGTAAAAAAGTTCTATTTCCGTAGGGGCTAATATAAGA
2 micron origin
440 460 480 500 520

ATACCGATGTGGATTGCGCATACTTTGTGAACAGAAAGTGATAGCGTTGATGATTCTTCATTGGTCAGAAAATTATGAACGGTTTCTTCTATTTTGTCTCTATATAC
TATGGCTACACCTAACGCGTATGAAACACTTGTCTTTCACTATCGCAACTACTAAGAAGTAACCAGTCTTTTAATACTTGCCAAAGAAGATAAAACAGAGATATATG
2 micron origin
540 560 580 600 620 640

TACGTATAGGAAATGTTTACATTTTCGTATTGTTTTCGATTCACTCTATGAATAGTTCTTACTACAATTTTTTTGTCTAAAGAGTAATACTAGAGATAAACATAAAA
ATGCATATCCTTTACAAATGTAAAAGCATAACAAAAGCTAAGTGAGATACTTATCAAGAATGATGTTAAAAAAACAGATTTCTCATTATGATCTCTATTTGTATTTT
2 micron origin
660 680 700 720 740

AATGTAGAGGTCGAGTTTAGATGCAAGTTCAAGGAGCGAAAGGTGGATGGGTAGGTTATATAGGGATATAGCACAGAGATATATAGCAAAGAGATACTTTTGAGCAA
TTACATCTCCAGCTCAAATCTACGTTCAAGTTCCTCGCTTTCCACCTACCCATCCAATATATCCCTATATCGTGTCTCTATATATCGTTTCTCTATGAAAACTCGTT
2 micron origin
760 780 800 820 840

TGTTTGTGGAAGCGGTATTCGCAATATTTTAGTAGCTCGTTACAGTCCGGTGCGTTTTTGGTTTTTTGAAAGTGCGTCTTCAGAGCGCTTTTGGTTTTCAAAAGCGC
ACAAACACCTTCGCCATAAGCGTTATAAAATCATCGAGCAATGTCAGGCCACGCAAAAACCAAAAAACTTTCACGCAGAAGTCTCGCGAAAACCAAAAGTTTTCGCG

2 micron origin

860 880 900 920 940 960

TCTGAAGTTCCTATACTTTCTAGCTAGAGAATAGGAACTTCGGAATAGGAACTTCAAAGCGTTTCCGAAAACGAGCGCTTCCGAAAATGCAACGCGAGCTGCGCACA
AGACTTCAAGGATATGAAAGATCGATCTCTTATCCTTGAAGCCTTATCCTTGAAGTTTCGCAAAGGCTTTTGCTCGCGAAGGCTTTTACGTTGCGCTCGACGCGTGT

2 micron origin

980 1,000 1,020 1,040 1,060

TACAGCTCACTGTTCACGTCGCACCTATATCTGCGTGTTGCCTGTATATATATATACATGAGAAGAACGGCATAGTGCGTGTTTATGCTTAAATGCGTACTTATATG
ATGTCGAGTGACAAGTGCAGCGTGGATATAGACGCACAACGGACATATATATATATGTACTCTTCTTGCCGTATCACGCACAAATACGAATTTACGCATGAATATAC

2 micron origin

1,080 1,100 1,120 1,140 1,160

CGTCTATTTATGTAGGATGAAAGGTAGTCTAGTACCTCCTGTGATATTATCCCATTCCATGCGGGGTATCGTATGCTTCCTTCAGCACTACCCTTTAGCTGTTCTAT
GCAGATAAATACATCCTACTTTCCATCAGATCATGGAGGACACTATAATAGGGTAAGGTACGCCCCATAGCATACGAAGGAAGTCGTGATGGGAAATCGACAAGATA

2 micron origin

1,180 1,200 1,220 1,240 1,260 1,280

ATGCTGCCACTCCTCAATTGGATTAGTCTCATCCTTCAATGCTATCATTTCCTTTGATATTGGATCGATCCGATGATAAGCTGTCAAACATGAGAATTGGGTAATAA
TACGACGGTGAGGAGTTAACCTAATCAGAGTAGGAAGTTACGATAGTAAAGGAAACTATAACCTAGCTAGGCTACTATTCGACAGTTTGTACTCTTAACCCATTATT

2 micron origin URA3

1,300 1,320 1,340 1,360 1,380

CTGATATAATTAAATTGAAGCTCTAATTTGTGAGTTTAGTATACATGCATTTACTTATAATACAGTTTTTTAGTTTTGCTGGCCGCATCTTCTCAAATATGCTTCCC
GACTATATTAATTTAACTTCGAGATTAAACACTCAAATCATATGTACGTAAATGAATATTATGTCAAAAAATCAAAACGACCGGCGTAGAAGAGTTTATACGAAGGG

URA3

1,400 1,420 1,440 1,460 1,480

AGCCTGCTTTTCTGTAACGTTCACCCTCTACCTTAGCATCCCTTCCCTTTGCAAATAGTCCTCTTCCAACAATAATAATGTCAGATCCTGTAGAGACCACATCATCC
TCGGACGAAAAGACATTGCAAGTGGGAGATGGAATCGTAGGGAAGGGAAACGTTTATCAGGAGAAGGTTGTTATTATTACAGTCTAGGACATCTCTGGTGTAGTAGG

URA3

1,500 1,520 1,540 1,560 1,580 1,600

ACGGTTCTATACTGTTGACCCAATGCGTCTCCCTTGTCATCTAAACCCACACCGGGTGTCATAATCAACCAATCGTAACCTTCATCTCTTCCACCCATGTCTCTTTG
TGCCAAGATATGACAACTGGGTTACGCAGAGGGAACAGTAGATTTGGGTGTGGCCCACAGTATTAGTTGGTTAGCATTGGAAGTAGAGAAGGTGGGTACAGAGAAAC

URA3

1,620 1,640 1,660 1,680 1,700

AGCAATAAAGCCGATAACAAAATCTTTGTCGCTCTTCGCAATGTCAACAGTACCCTTAGTATATTCTCCAGTAGATAGGGAGCCCTTGCATGACAATTCTGCTAACA
TCGTTATTTCGGCTATTGTTTTAGAAACAGCGAGAAGCGTTACAGTTGTCATGGGAATCATATAAGAGGTCATCTATCCCTCGGGAACGTACTGTTAAGACGATTGT

URA3

1,720 1,740 1,760 1,780 1,800

TCAAAAGGCCTCTAGGTTCCTTTGTTACTTCTTCTGCCGCCTGCTTCAAACCGCTAACAATACCTGGGCCCACCACACCGTGTGCATTCGTAATGTCTGCCCATTCT
AGTTTTCCGGAGATCCAAGGAAACAATGAAGAAGACGGCGGACGAAGTTTGGCGATTGTTATGGACCCGGGTGGTGTGGCACACGTAAGCATTACAGACGGGTAAGA

URA3

1,820 1,840 1,860 1,880 1,900 1,920

GCTATTCTGTATACACCCGCAGAGTACTGCAATTTGACTGTATTACCAATGTCAGCAAATTTTCTGTCTTCGAAGAGTAAAAAATTGTACTTGGCGGATAATGCCTT
CGATAAGACATATGTGGGCGTCTCATGACGTTAAACTGACATAATGGTTACAGTCGTTTAAAAGACAGAAGCTTCTCATTTTTTAACATGAACCGCCTATTACGGAA

URA3

1,940 1,960 1,980 2,000 2,020

TAGCGGCTTAACTGTGCCCTCCATGGAAAAATCAGTCAAGATATCCACATGTGTTTTTAGTAAACAAATTTTGGGACCTAATGCTTCAACTAACTCCAGTAATTCCT
ATCGCCGAATTGACACGGGAGGTACCTTTTTAGTCAGTTCTATAGGTGTACACAAAAATCATTTGTTTAAAACCCTGGATTACGAAGTTGATTGAGGTCATTAAGGA

URA3

2,040 2,060 2,080 2,100 2,120 2,140

TGGTGGTACGAACATCCAATGAAGCACACAAGTTTGTTTGCTTTTCGTGCATGATATTAAATAGCTTGGCAGCAACAGGACTAGGATGAGTAGCAGCACGTTCCTTA
ACCACCATGCTTGTAGGTTACTTCGTGTGTTCAAACAAACGAAAAGCACGTACTATAATTTATCGAACCGTCGTTGTCCTGATCCTACTCATCGTCGTGCAAGGAAT

URA3

2,160 2,180 2,200 2,220 2,240

TATGTAGCTTTCGACATGATTTATCTTCGTTTCCTGCATGTTTTTGTTCTGTGCAGTTGGGTTAAGAATACTGGGCAATTTCATGTTTCTTCAACACTACATATGCG
ATACATCGAAAGCTGTACTAAATAGAAGCAAAGGACGTACAAAAACAAGACACGTCAACCCAATTCTTATGACCCGTTAAAGTACAAAGAAGTTGTGATGTATACGC

URA3

2,260 2,280 2,300 2,320 2,340

TATATATACCAATCTAAGTCTGTGCTCCTTCCTTCGTTCTTCCTTCTGTTCGGAGATTACCGAATCAAAAAAATTTCAAAGAAACCGAAATCAAAAAAAAGAATAAA
ATATATATGGTTAGATTCAGACACGAGGAAGGAAGCAAGAAGGAAGACAAGCCTCTAATGGCTTAGTTTTTTTAAAGTTTCTTTGGCTTTAGTTTTTTTTCTTATTT

URA3

2,360 2,380 2,400 2,420 2,440 2,460

AAAAAAATGATGAATTGAATTGAAAAGCTAATTCTTGAAGACGAAAGGGCCTCGTGATACGCCTATTTTTATAGGTTAATGTCATGATAATAATGGTTTCTTAGACG
TTTTTTTACTACTTAACTTAACTTTTCGATTAAGAACTTCTGCTTTCCCGGAGCACTATGCGGATAAAAATATCCAATTACAGTACTATTATTACCAAAGAATCTGC

URA3

2,480 2,500 2,520 2,540 2,560

TCAGGTGGCACTTTTCGGGGAAATGTGCGCGGAACCCCTATTTGTTTATTTTTCTAAATACATTCAAATATGTATCCGCTCATGAGACAATAACCCTGATAAATGCT
AGTCCACCGTGAAAAGCCCCTTTACACGCGCCTTGGGGATAAACAAATAAAAAGATTTATGTAAGTTTATACATAGGCGAGTACTCTGTTATTGGGACTATTTACGA

2,580 2,600 2,620 2,640 2,660

TCAATAATATTGAAAAAGGAAGAGTATGAGTATTCAACATTTCCGTGTCGCCCTTATTCCCTTTTTTGCGGCATTTTGCCTTCCTGTTTTTGCTCACCCAGAAACGC
AGTTATTATAACTTTTTCCTTCTCATACTCATAAGTTGTAAAGGCACAGCGGGAATAAGGGAAAAAACGCCGTAAAACGGAAGGACAAAAACGAGTGGGTCTTTGCG

2,680 2,700 2,720 2,740 2,760 2,780

TGGTGAAAGTAAAAGATGCTGAAGATCAGTTGGGTGCACGAGTGGGTTACATCGAACTGGATCTCAACAGCGGTAAGATCCTTGAGAGTTTTCGCCCCGAAGAACGT
ACCACTTTCATTTTCTACGACTTCTAGTCAACCCACGTGCTCACCCAATGTAGCTTGACCTAGAGTTGTCGCCATTCTAGGAACTCTCAAAAGCGGGGCTTCTTGCA

2,800 2,820 2,840 2,860 2,880

TTTCCAATGATGAGCACTTTTAAAGTTCTGCTATGTGGCGCGGTATTATCCCGTATTGACGCCGGGCAAGAGCAACTCGGTCGCCGCATACACTATTCTCAGAATGA
AAAGGTTACTACTCGTGAAAATTTCAAGACGATACACCGCGCCATAATAGGGCATAACTGCGGCCCGTTCTCGTTGAGCCAGCGGCGTATGTGATAAGAGTCTTACT

AmpR

2,900 2,920 2,940 2,960 2,980

CTTGGTTGAGTACTCACCAGTCACAGAAAAGCATCTTACGGATGGCATGACAGTAAGAGAATTATGCAGTGCTGCCATAACCATGAGTGATAACACTGCGGCCAACT
GAACCAACTCATGAGTGGTCAGTGTCTTTTCGTAGAATGCCTACCGTACTGTCATTCTCTTAATACGTCACGACGGTATTGGTACTCACTATTGTGACGCCGGTTGA

AmpR

3,000 3,020 3,040 3,060 3,080 3,100

TACTTCTGACAACGATCGGAGGACCGAAGGAGCTAACCGCTTTTTTGCACAACATGGGGGATCATGTAACTCGCCTTGATCGTTGGGAACCGGAGCTGAATGAAGCC
ATGAAGACTGTTGCTAGCCTCCTGGCTTCCTCGATTGGCGAAAAAACGTGTTGTACCCCCTAGTACATTGAGCGGAACTAGCAACCCTTGGCCTCGACTTACTTCGG

AmpR

3,120 3,140 3,160 3,180 3,200

ATACCAAACGACGAGCGTGACACCACGATGCCTGTAGCAATGGCAACAACGTTGCGCAAACTATTAACTGGCGAACTACTTACTCTAGCTTCCCGGCAACAATTAAT
TATGGTTTGCTGCTCGCACTGTGGTGCTACGGACATCGTTACCGTTGTTGCAACGCGTTTGATAATTGACCGCTTGATGAATGAGATCGAAGGGCCGTTGTTAATTA

AmpR

3,220 3,240 3,260 3,280 3,300

AGACTGGATGGAGGCGGATAAAGTTGCAGGACCACTTCTGCGCTCGGCCCTTCCGGCTGGCTGGTTTATTGCTGATAAATCTGGAGCCGGTGAGCGTGGGTCTCGCG
TCTGACCTACCTCCGCCTATTTCAACGTCCTGGTGAAGACGCGAGCCGGGAAGGCCGACCGACCAAATAACGACTATTTAGACCTCGGCCACTCGCACCCAGAGCGC

AmpR

3,320 3,340 3,360 3,380 3,400 3,420

GTATCATTGCAGCACTGGGGCCAGATGGTAAGCCCTCCCGTATCGTAGTTATCTACACGACGGGGAGTCAGGCAACTATGGATGAACGAAATAGACAGATCGCTGAG
CATAGTAACGTCGTGACCCCGGTCTACCATTCGGGAGGGCATAGCATCAATAGATGTGCTGCCCCTCAGTCCGTTGATACCTACTTGCTTTATCTGTCTAGCGACTC

AmpR

3,440 3,460 3,480 3,500 3,520

ATAGGTGCCTCACTGATTAAGCATTGGTAACTGTCAGACCAAGTTTACTCATATATACTTTAGATTGATTTAAAACTTCATTTTTAATTTAAAAGGATCTAGGTGAA
TATCCACGGAGTGACTAATTCGTAACCATTGACAGTCTGGTTCAAATGAGTATATATGAAATCTAACTAAATTTTGAAGTAAAAATTAAATTTTCCTAGATCCACTT

AmpR

3,540 3,560 3,580 3,600 3,620

GATCCTTTTTGATAATCTCATGACCAAAATCCCTTAACGTGAGTTTTCGTTCCACTGAGCGTCAGACCCCGTAGAAAAGATCAAAGGATCTTCTTGAGATCCTTTTT
CTAGGAAAAACTATTAGAGTACTGGTTTTAGGGAATTGCACTCAAAAGCAAGGTGACTCGCAGTCTGGGGCATCTTTTCTAGTTTCCTAGAAGAACTCTAGGAAAAA
ColE1 origin
3,640 3,660 3,680 3,700 3,720 3,740

TTCTGCGCGTAATCTGCTGCTTGCAAACAAAAAAACCACCGCTACCAGCGGTGGTTTGTTTGCCGGATCAAGAGCTACCAACTCTTTTTCCGAAGGTAACTGGCTTC
AAGACGCGCATTAGACGACGAACGTTTGTTTTTTTGGTGGCGATGGTCGCCACCAAACAAACGGCCTAGTTCTCGATGGTTGAGAAAAAGGCTTCCATTGACCGAAG
ColE1 origin
3,760 3,780 3,800 3,820 3,840

AGCAGAGCGCAGATACCAAATACTGTCCTTCTAGTGTAGCCGTAGTTAGGCCACCACTTCAAGAACTCTGTAGCACCGCCTACATACCTCGCTCTGCTAATCCTGTT
TCGTCTCGCGTCTATGGTTTATGACAGGAAGATCACATCGGCATCAATCCGGTGGTGAAGTTCTTGAGACATCGTGGCGGATGTATGGAGCGAGACGATTAGGACAA
ColE1 origin
3,860 3,880 3,900 3,920 3,940

ACCAGTGGCTGCTGCCAGTGGCGATAAGTCGTGTCTTACCGGGTTGGACTCAAGACGATAGTTACCGGATAAGGCGCAGCGGTCGGGCTGAACGGGGGGTTCGTGCA
TGGTCACCGACGACGGTCACCGCTATTCAGCACAGAATGGCCCAACCTGAGTTCTGCTATCAATGGCCTATTCCGCGTCGCCAGCCCGACTTGCCCCCCAAGCACGT
ColE1 origin
3,960 3,980 4,000 4,020 4,040 4,060

CACAGCCCAGCTTGGAGCGAACGACCTACACCGAACTGAGATACCTACAGCGTGAGCTATGAGAAAGCGCCACGCTTCCCGAAGGGAGAAAGGCGGACAGGTATCCG
GTGTCGGGTCGAACCTCGCTTGCTGGATGTGGCTTGACTCTATGGATGTCGCACTCGATACTCTTTCGCGGTGCGAAGGGCTTCCCTCTTTCCGCCTGTCCATAGGC
ColE1 origin
4,080 4,100 4,120 4,140 4,160

GTAAGCGGCAGGGTCGGAACAGGAGAGCGCACGAGGGAGCTTCCAGGGGGAAACGCCTGGTATCTTTATAGTCCTGTCGGGTTTCGCCACCTCTGACTTGAGCGTCG
CATTCGCCGTCCCAGCCTTGTCCTCTCGCGTGCTCCCTCGAAGGTCCCCCTTTGCGGACCATAGAAATATCAGGACAGCCCAAAGCGGTGGAGACTGAACTCGCAGC
ColE1 origin
4,180 4,200 4,220 4,240 4,260 4,280

ATTTTTGTGATGCTCGTCAGGGGGGCGGAGCCTATGGAAAAACGCCAGCAACGCGGCCTTTTTACGGTTCCTGGCCTTTTGCTGGCCTTTTGCTCACATGTTCTTTC
TAAAAACACTACGAGCAGTCCCCCCGCCTCGGATACCTTTTTGCGGTCGTTGCGCCGGAAAAATGCCAAGGACCGGAAAACGACCGGAAAACGAGTGTACAAGAAAG
ColE1 origin
4,300 4,320 4,340 4,360 4,380

CTGCGTTATCCCCTGATTCTGTGGATAACCGTATTACCGCCTTTGAGTGAGCTGATACCGCTCGCCGCAGCCGAACGACCGAGCGCAGCGAGTCAGTGAGCGAGGAA
GACGCAATAGGGGACTAAGACACCTATTGGCATAATGGCGGAAACTCACTCGACTATGGCGAGCGGCGTCGGCTTGCTGGCTCGCGTCGCTCAGTCACTCGCTCCTT
4,400 4,420 4,440 4,460 4,480

GCGGAAGAGCGCCCAATACGCAAACCGCCTCTCCCCGCGCGTTGGCCGATTCATTAATGCAGCTGGCACGACAGGTTTCCCGACTGGAAAGCGGGCAGTGAGCGCAA
CGCCTTCTCGCGGGTTATGCGTTTGGCGGAGAGGGGCGCGCAACCGGCTAAGTAATTACGTCGACCGTGCTGTCCAAAGGGCTGACCTTTCGCCCGTCACTCGCGTT
4,500 4,520 4,540 4,560 4,580 4,600

CGCAATTAATGTGAGTTAGCTCACTCATTAGGCACCCCAGGCTTTACACTTTATGCTTCCGGCTCGTATGTTGTGTGGAATTGTGAGCGGATAACAATTTCACACAG
GCGTTAATTACACTCAATCGAGTGAGTAATCCGTGGGGTCCGAAATGTGAAATACGAAGGCCGAGCATACAACACACCTTAACACTCGCCTATTGTTAAAGTGTGTC

lac promoter LacO

4,620 4,640 4,660 4,680 4,700

GAAACAGCTATGACCATGATTACGCCAAGCTTACCAGTTCTCACACGGAACACCACTAATGGACACAAATTCGAAATACTTTGACCCTATTTTCGAGGACCTTGTCA
CTTTGTCGATACTGGTACTAATGCGGTTCGAATGGTCAAGAGTGTGCCTTGTGGTGATTACCTGTGTTTAAGCTTTATGAAACTGGGATAAAAGCTCCTGGAACAGT

M13-rev GAPDH promoter

4,720 4,740 4,760 4,780 4,800

CCTTGAGCCCAAGAGAGCCAAGATTTAAATTTTCCTATGACTTGATGCAAATTCCCAAAGCTAATAACATGCAAGACACGTACGGTCAAGAAGACATATTTGACCTC
GGAACTCGGGTTCTCTCGGTTCTAAATTTAAAAGGATACTGAACTACGTTTAAGGGTTTCGATTATTGTACGTTCTGTGCATGCCAGTTCTTCTGTATAAACTGGAG

GAPDH promoter

4,820 4,840 4,860 4,880 4,900 4,920

TTAACAGGTTCAGACGCGACTGCCTCATCAGTAAGACCCGTTGAAAAGAACTTACCTGAAAAAAACGAATATATACTAGCGTTGAATGTTAGCGTCAACAACAAGAA
AATTGTCCAAGTCTGCGCTGACGGAGTAGTCATTCTGGGCAACTTTTCTTGAATGGACTTTTTTTGCTTATATATGATCGCAACTTACAATCGCAGTTGTTGTTCTT

GAPDH promoter

4,940 4,960 4,980 5,000 5,020

GTTTAATGACGCGGAGGCCAAGGCAAAAAGATTCCTTGATTACGTAAGGGAGTTAGAATCATTTTGAATAAAAAACACGCTTTTTCAGTTCGAGTTTATCATTATCA
CAAATTACTGCGCCTCCGGTTCCGTTTTTCTAAGGAACTAATGCATTCCCTCAATCTTAGTAAAACTTATTTTTTGTGCGAAAAAGTCAAGCTCAAATAGTAATAGT

GAPDH promoter

5,040 5,060 5,080 5,100 5,120

ATACTGCCATTTCAAAGAATACGTAAATAATTAATAGTAGTGATTTTCCTAACTTTATTTAGTCAAAAAATTAGCCTTTTAATTCTGCTGTAACCCGTACATGCCCA
TATGACGGTAAAGTTTCTTATGCATTTATTAATTATCATCACTAAAAGGATTGAAATAAATCAGTTTTTTAATCGGAAAATTAAGACGACATTGGGCATGTACGGGT

GAPDH promoter

5,140 5,160 5,180 5,200 5,220 5,240

AAATAGGGGGCGGGTTACACAGAATATATAACATCGTAGGTGTCTGGGTGAACAGTTTATTCCTGGCATCCACTAAATATAATGGAGCCCGCTTTTTAAGCTGGCAT
TTTATCCCCCGCCCAATGTGTCTTATATATTGTAGCATCCACAGACCCACTTGTCAAATAAGGACCGTAGGTGATTTATATTACCTCGGGCGAAAAATTCGACCGTA

GAPDH promoter

5,260 5,280 5,300 5,320 5,340

CCAGAAAAAAAAAGAATCCCAGCACCAAAATATTGTTTTCTTCACCAACCATCAGTTCATAGGTCCATTCTCTTAGCGCAACTACAGAGAACAGGGGCACAAACAGG
GGTCTTTTTTTTTCTTAGGGTCGTGGTTTTATAACAAAAGAAGTGGTTGGTAGTCAAGTATCCAGGTAAGAGAATCGCGTTGATGTCTCTTGTCCCCGTGTTTGTCC

GAPDH promoter

5,360 5,380 5,400 5,420 5,440

```
CAAAAAACGGGCACAACCTCAATGGAGTGATGCAACCTGCCTGGAGTAAATGATGACACAAGGCAATTGACCCACGCATGTATCTATCTCATTTTCTTACACCTTCT
GTTTTTTGCCCGTGTTGGAGTTACCTCACTACGTTGGACGGACCTCATTTACTACTGTGTTCCGTTAACTGGGTGCGTACATAGATAGAGTAAAAGAATGTGGAAGA
GAPDH promoter
5,460  5,480  5,500  5,520  5,540  5,560

ATTACCTTCTGCTCTCTCTGATTTGGAAAAAGCTGAAAAAAAAGGTTGAAACCAGTTCCCTGAAATTATTCCCCTACTTGACTAATAAGTATATAAAGACGGTAGGT
TAATGGAAGACGAGAGAGACTAAACCTTTTTCGACTTTTTTTTCCAACTTTGGTCAAGGGACTTTAATAAGGGGATGAACTGATTATTCATATATTTCTGCCATCCA
GAPDH promoter
5,580  5,600  5,620  5,640  5,660

ATTGATTGTAATTCTGTAAATCTATTTCTTAAACTTCTTAAATTCTACTTTTATAGTTAGTCTTTTTTTTAGTTTTAAAACACCAAGAACTTAGTTTCGAATAAACA
TAACTAACATTAAGACATTTAGATAAAGAATTTGAAGAATTTAAGATGAAAATATCAATCAGAAAAAAAATCAAAATTTTGTGGTTCTTGAATCAAAGCTTATTTGT
GAPDH promoter
5,680  5,700  5,720  5,740  5,760

CACATAAACAGAATTCATGAGATTCCCATCTATCTTCACCGCTGTTTTGTTCGCTGCTTCTTCTGCTTTGGCTGCTCCAGCTAACACCACCACCGAAGACGAAACCG
GTGTATTTGTCTTAAGTACTCTAAGGGTAGATAGAAGTGGCGACAAAACAAGCGACGAAGAAGACGAAACCGACGAGGTCGATTGTGGTGGTGGCTTCTGCTTTGGC
1  2  3  4  5  6  7  8  9  10 11 12 13 14 15 16 17 18 19 20 21 22 23 24 25 26 27 28 29 30
M  R  F  P  S  I  F  T  A  V  L  F  A  A  S  S  A  L  A  A  P  A  N  T  T  T  E  D  E  T
Mating factor alpha leader
5,780  5,800  5,820  5,840  5,860  5,880

CTCAAATCCCAGCTGAAGCTGTTATCGACTACTCTGACTTGGAAGGTGACTTCGACGCTGCTGCTTTGCCATTGTCTAACTCTACCAACAACGGTTTGTCTTCTACC
GAGTTTAGGGTCGACTTCGACAATAGCTGATGAGACTGAACCTTCCACTGAAGCTGCGACGACGAAACGGTAACAGATTGAGATGGTTGTTGCCAAACAGAAGATGG
31 32 33 34 35 36 37 38 39 40 41 42 43 44 45 46 47 48 49 50 51 52 53 54 55 56 57 58 59 60 61 62 63 64 65 66
A  Q  I  P  A  E  A  V  I  D  Y  S  D  L  E  G  D  F  D  A  A  A  L  P  L  S  N  S  T  N  N  G  L  S  S  T
Mating factor alpha leader
5,900  5,920  5,940  5,960  5,980

AACACCACCATCGCTTCTATCGCTGCTAAGGAAGAAGGTGTTCAATTGGACAAGAGAGAAGCTAGCGCAGGATCCGATTACAAGGATGACGATGACAAGCAAACATC
TTGTGGTGGTAGCGAAGATAGCGACGATTCCTTCTTCCACAAGTTAACCTGTTCTCTCTTCGATCGCGTCCTAGGCTAATGTTCCTACTGCTACTGTTCGTTTGTAG
67 68 69 70 71 72 73 74 75 76 77 78 79 80 81 82 83 84 85 86 1  2  3  4  5  1  2  3  4  5  6  7  8     2
N  T  T  I  A  S  I  A  A  K  E  E  G  V  Q  L  D  K  R  E  A  S  A  G  S  D  Y  K  D  D  D  D  K  Q  T  S
Mating factor alpha leader    FLAG
6,000  6,020  6,040  6,060  6,080

CTCACCCTCTACAGAAGCATCAGTATCAACCTCTAGTACCTCTTCCTCTGCTTCACAATCATCTGACCCAACTACAACATCTTCGTCCAGTTCATCGTCTTCCCCAT
GAGTGGGAGATGTCTTCGTAGTCATAGTTGGAGATCATGGAGAAGGAGACGAAGTGTTAGTAGACTGGGTTGATGTTGTAGAAGCAGGTCAAGTAGCAGAAGGGGTA
   4     6     8     10    12    14    16    18    20    22    24    26    28    30    32    34    36    38
S  P  S  T  E  A  S  V  S  T  S  S  T  S  S  S  A  S  Q  S  S  D  P  T  T  T  S  S  S  S  S  S  S  S  P
649 stalk
6,100  6,120  6,140  6,160  6,180  6,200
```

CTTCCCAATCTGAGGAAATTTCATCATCACCGACGGTCTCAACTACACCATCAACTTCTTCATCATCTTCCTCAATGACTTCAACCACCACAACAAAGTCAATCTCA
GAAGGGTTAGACTCCTTTAAAGTAGTAGTGGCTGCCAGAGTTGATGTGGTAGTTGAAGAAGTAGTAGAAGGAGTTACTGAAGTTGGTGGTGTTGTTTCAGTTAGAGT

40 42 44 46 48 50 52 54 56 58 60 62 64 66 68 70 72 74

S S Q S E E I S S S P T V S T T P S T S S S S S M T S T T T T K S I S

649 stalk

6,220 6,240 6,260 6,280 6,300

ACTTCCACTACAAGTTCAGCTCCAGTTACAGATGTGACAGTTTCCTCATCGCCTAGTAAATCTACCTCTACTTCGACAAGTACAGAAACATCTAAAACACCTACTTC
TGAAGGTGATGTTCAAGTCGAGGTCAATGTCTACACTGTCAAAGGAGTAGCGGATCATTTAGATGGAGATGAAGCTGTTCATGTCTTTGTAGATTTTGTGGATGAAG

76 78 80 82 84 86 88 90 92 94 96 98 100 102 104 106 108 110

T S T T S S A P V T D V T V S S S P S K S T S T S T S T E T S K T P T S

649 stalk

6,320 6,340 6,360 6,380 6,400 6,420

AATGACAGAGTATACATCTAGTACATCGATAATTTCGACTCCAGTTAGTCACTCGCAGACAGGTTTGTCGGCTTCATCAAGTTCATCATCTACAACATCCGGTTCTT
TTACTGTCTCATATGTAGATCATGTAGCTATTAAAGCTGAGGTCAATCAGTGAGCGTCTGTCCAAACAGCCGAAGTAGTTCAAGTAGTAGATGTTGTAGGCCAAGAA

112 114 116 118 120 122 124 126 128 130 132 134 136 138 140 142 144

M T E Y T S S T S I I S T P V S H S Q T G L S A S S S S S S T T S G S

649 stalk

6,440 6,460 6,480 6,500 6,520

CGTCCACTAAATCAGAAAGTTCGACAACATCTGGCTCTTCCCAGTCCGTGGAATCAACCTCCAGCCACGCCACTGTTCTTGCTAATTCCGCAGAAATGGTCACAACA
GCAGGTGATTTAGTCTTTCAAGCTGTTGTAGACCGAGAAGGGTCAGGCACCTTAGTTGGAGGTCGGTGCGGTGACAAGAACGATTAAGGCGTCTTTACCAGTGTTGT

146 148 150 152 154 156 158 160 162 164 166 168 170 172 174 176 178 180

S S T K S E S S T T S G S S Q S V E S T S S H A T V L A N S A E M V T T

649 stalk

6,540 6,560 6,580 6,600 6,620

TCCTCTAGTTCATCCTCAACATCCGAAATGTCATTAACTAGTACTGCTACCAGTGTACCAGTCTCATCTAGTAGCAGTACGACATATTCTACTAGCGCATCTACACA
AGGAGATCAAGTAGGAGTTGTAGGCTTTACAGTAATTGATCATGACGATGGTCACATGGTCAGAGTAGATCATCGTCATGCTGTATAAGATGATCGCGTAGATGTGT

182 184 186 188 190 192 194 196 198 200 202 204 206 208 210 212 214 216

S S S S S S T S E M S L T S T A T S V P V S S S S S T T Y S T S A S T Q

649 stalk

6,640 6,660 6,680 6,700 6,720 6,740

AGCCGTCACTACAACATCTTCTTCCACTGTATCTACAACTTCTTCTAGTACAACGTTAACAAGCGCATTCACACATTCTTCAACCACATCGTCCGACCAGCCACCCA
TCGGCAGTGATGTTGTAGAAGAAGGTGACATAGATGTTGAAGAAGATCATGTTGCAATTGTTCGCGTAAGTGTGTAAGAAGTTGGTGTAGCAGGCTGGTCGGTGGGT

218 220 222 224 226 228 230 232 234 236 238 240 242 244 246 248 250 252

A V T T T S S S T V S T T S S S T T L T S A F T H S S T T S S D Q P P

649 stalk

6,760 6,780 6,800 6,820 6,840

GCGACACTACAAGTCCATCTACGACACACGAACCTCATGTAACCACTCAGACGTCATCAGAAACATCTTCTTCTAAGTCATCTTCTACTTCTTCTTCAAGTACATCT
CGCTGTGATGTTCAGGTAGATGCTGTGTGCTTGGAGTACATTGGTGAGTCTGCAGTAGTCTTTGTAGAAGAAGATTCAGTAGAAGATGAAGAAGAAGTTCATGTAGA
254 256 258 260 262 264 266 268 270 272 274 276 278 280 282 284 286 288
S D T T S P S T T H E P H V T T Q T S S E T S S S K S S S T S S S S T S

649 stalk

6,860 6,880 6,900 6,920 6,940

CAAACCTCTGAGTCTGCAACACCATCCGATTCCGTATCACCTGGAAGTTCTACATCAACATCTTCTAGTAGCACTTCTACTTCCACTTCTATTTCCAGTGGAGAAAC
GTTTGGAGACTCAGACGTTGTGGTAGGCTAAGGCATAGTGGACCTTCAAGATGTAGTTGTAGAAGATCATCGTGAAGATGAAGGTGAAGATAAAGGTCACCTCTTTG
290 292 294 296 298 300 302 304 306 308 310 312 314 316 318 320 322 324
Q T S E S A T P S D S V S P G S S T S T S S S S T S T S T S I S S G E T

649 stalk

6,960 6,980 7,000 7,020 7,040 7,060

GACAACTTCTTCTTCTTCATCATCTGCCACGACCACTTCTAACAGCGCAACCTTGTCAGTCTCTACCACACAAACTTCGATTGAAGCCAGTTCATCTACTACATCTA
CTGTTGAAGAAGAAGAAGTAGTAGACGGTGCTGGTGAAGATTGTCGCGTTGGAACAGTCAGAGATGGTGTGTTTGAAGCTAACTTCGGTCAAGTAGATGATGTAGAT
326 328 330 332 334 336 338 340 342 344 346 348 350 352 354 356 358
T T S S S S S S A T T T S N S A T L S V S T T Q T S I E A S S S T T S

649 stalk

7,080 7,100 7,120 7,140 7,160

CATCTAGTTCAACAATTACAACTTCAAGTAGTAGCGCTCACATATCGTCGAAATCTCAATCTAGTATTACCTATCCCTCTTCCTCGACATCTTCATCTACATCGTCC
GTAGATCAAGTTGTTAATGTTGAAGTTCATCATCGCGAGTGTATAGCAGCTTTAGAGTTAGATCATAATGGATAGGGAGAAGGAGCTGTAGAAGTAGATGTAGCAGG
360 362 364 366 368 370 372 374 376 378 380 382 384 386 388 390 392 394
T S S S T I T T S S S S A H I S S K S Q S S I T Y P S S S T S S S T S S

649 stalk

7,180 7,200 7,220 7,240 7,260

TCAATTTCTAGCGAATCTGAAAGTTTTGAATCGACATCAGCAGAAGATGCTCCATCAACAGCACCTTCATCAAGTGTCTCTTCTAAGAGTTCTACCTCTACAACATC
AGTTAAAGATCGCTTAGACTTTCAAAACTTAGCTGTAGTCGTCTTCTACGAGGTAGTTGTCGTGGAAGTAGTTCACAGAGAAGATTCTCAAGATGGAGATGTTGTAG
396 398 400 402 404 406 408 410 412 414 416 418 420 422 424 426 428 430
S I S S E S E S F E S T S A E D A P S T A P S S S V S S K S S T S T T S

649 stalk

7,280 7,300 7,320 7,340 7,360 7,380

AAGCACATCGACATCTTCAAGCACTCCATCTCCATCACCATCTTCCGTGAGTTCTTCCTCCACCAGCTCATTGACAACTTCTGCTGTATCAACACCAGCTACCTCTC
TTCGTGTAGCTGTAGAAGTTCGTGAGGTAGAGGTAGTGGTAGAAGGCACTCAAGAAGGAGGTGGTCGAGTAACTGTTGAAGACGACATAGTTGTGGTCGATGGAGAG
432 434 436 438 440 442 444 446 448 450 452 454 456 458 460 462 464 466
S T S T S S S T P S P S P S S V S S S S T S S L T T S A V S T P A T S

649 stalk

7,400 7,420 7,440 7,460 7,480

ATTCTCAAAGTACTGTAGTAACCACCACTACTATTACTACATCAACAGGTCCAGTGATGTCTACGACAACAGCTTATTCTTCTAGTTCTACTAGCAGCTCGGAATCT
TAAGAGTTTCATGACATCATTGGTGGTGATGATAATGATGTAGTTGTCCAGGTCACTACAGATGCTGTTGTCGAATAAGAAGATCAAGATGATCGTCGAGCCTTAGA
468 470 472 474 476 478 480 482 484 486 488 490 492 494 496 498 500 502
H S Q S T V V T T T T I T T S T G P V M S T T T A Y S S S S T S S S E S

649 stalk

7,500 7,520 7,540 7,560 7,580

TCTGAGGTTCAGTCTGTCATGTCATCTACGCCTAGTTCAACATCAACAACAACCAGTTCGGAATCTACTTCATCTAGCTCCACAGCTTCTACCTCACCATCAACCTC
AGACTCCAAGTCAGACAGTACAGTAGATGCGGATCAAGTTGTAGTTGTTGTTGGTCAAGCCTTAGATGAAGTAGATCGAGGTGTCGAAGATGGAGTGGTAGTTGGAG
504 506 508 510 512 514 516 518 520 522 524 526 528 530 532 534 536 538
S E V Q S V M S S T P S S T S T T T S S E S T S S S S T A S T S P S T S

649 stalk

7,600 7,620 7,640 7,660 7,680 7,700

GCAAACTTTCGAAACTTCTCCTACTATAGGAGGTGTCCCCTCAACCACTTCATTTGTCTCTACGCCAACAACGAAATTGTCGCACACTACTTCCACTATGACAGCAC
CGTTTGAAAGCTTTGAAGAGGATGATATCCTCCACAGGGGAGTTGGTGAAGTAAACAGAGATGCGGTTGTTGCTTTAACAGCGTGTGATGAAGGTGATACTGTCGTG
540 542 544 546 548 550 552 554 556 558 560 562 564 566 568 570 572
Q T F E T S P T I G G V P S T T S F V S T P T T K L S H T T S T M T A

649 stalk

7,720 7,740 7,760 7,780 7,800

AGTCCGATAGTAAGTCTACCCACTCCTCAAGCACATCGACAGAAGATAAATCATCCACTGCTTCTGCAGTTGACGAAAGCACTACAACATCCACTTCCACGGAGTCT
TCAGGCTATCATTCAGATGGGTGAGGAGTTCGTGTAGCTGTCTTCTATTTAGTAGGTGACGAAGACGTCAACTGCTTTCGTGATGTTGTAGGTGAAGGTGCCTCAGA
574 576 578 580 582 584 586 588 590 592 594 596 598 600 602 604 606 608
Q S D S K S T H S S S T S T E D K S S T A S A V D E S T T T S T S T E S

649 stalk

7,820 7,840 7,860 7,880 7,900

ACTACATCAGTAACATCAGGCACCTCCCATTCCGCTAAAGAATCTTCGTCAAATTCTAAGGTGTATAGTTCACAGACAGCACACTCATCCATAAGTGTTGCATCATC
TGATGTAGTCATTGTAGTCCGTGGAGGGTAAGGCGATTTCTTAGAAGCAGTTTAAGATTCCACATATCAAGTGTCTGTCGTGTGAGTAGGTATTCACAACGTAGTAG
610 612 614 616 618 620 622 624 626 628 630 632 634 636 638 640 642 644
T T S V T S G T S H S A K E S S S N S K V Y S S Q T A H S S I S V A S S

649 stalk

7,920 7,940 7,960 7,980 8,000 8,020

ACCTAGTACAAAGGGCGCCCAAATCCAATCTTCTATGGTTGAAATCTCTACCTACGCTGGTTCTGCTAACTCTGTTAACGCTGGTGCTGGTGCTGGTGCTTTGTTCT
TGGATCATGTTTCCCGCGGGTTTAGGTTAGAAGATACCAACTTTAGAGATGGATGCGACCAAGACGATTGAGACAATTGCGACCACGACCACGACCACGAAACAAGA
646 648 650 652 654 656 658 660 662 664 666 668 670 672 674 676 678 680
P S T K G A Q I Q S S M V E I S T Y A G S A N S V N A G A G A G A L F

GPI anchor

8,040 8,060 8,080 8,100 8,120

TGTTGTTGTCTTTGGCTATCATCTAATGATTAATTAACTCGAGATCTGATAACAACAGTGTAGATGTAACAAAATCGACTTTGTTCCCACTGTACTTTTAGCTCGTA
ACAACAACAGAAACCGATAGTAGATTACTAATTAATTGAGCTCTAGACTATTGTTGTCACATCTACATTGTTTTAGCTGAAACAAGGGTGACATGAAAATCGAGCAT

682 684 686 688 1 2 3 4 5 6 7
L L L S L A I I * * L I N S R

GPI anchor
alpha factor terminator

8,140 8,160 8,180 8,200 8,220

CAAAATACAATATACTTTTCATTTCTCCGTAAACAACATGTTTTCCCATGTAATATCCTTTTCTATTTTTCGTTCCGTTACCAACTTTACACATACTTTATATAGCT
GTTTTATGTTATATGAAAAGTAAAGAGGCATTTGTTGTACAAAAGGGTACATTATAGGAAAAGATAAAAAGCAAGGCAATGGTTGAAATGTGTATGAAATATATCGA

alpha factor terminator

8,240 8,260 8,280 8,300 8,320 8,340

ATTCACTTCTATACACTAAAAAACTAAGACAATTTTAATTTTGCTGCCTGCCATATTTCAATTTGTTATAAATTCCTATAATTTATCCTATTAGTAGCTAAAAAAAG
TAAGTGAAGATATGTGATTTTTTGATTCTGTTAAAATTAAAACGACGGACGGTATAAAGTTAAACAATATTTAAGGATATTAAATAGGATAATCATCGATTTTTTTC

alpha factor terminator

8,360 8,380 8,400 8,420 8,440

ATGAATGTGAATCGAATCCTAAGAGAATTGGTACCGCGATGTAGTAAAACTAGCTAGACCGAGAAAGAGACTAGAAATGCAAAAGGCACTTCTACAATGGCTGCCAT
TACTTACACTTAGCTTAGGATTCTCTTAACCATGGCGCTACATCATTTTGATCGATCTGGCTCTTTCTCTGATCTTTACGTTTTCCGTGAAGATGTTACCGACGGTA

alpha factor terminator

8,460 8,480 8,500 8,520 8,540 8,560

CATTATTATCCGATGTGACGCTGCATTTTTTTTTTTTTTTTTTTTTTTTTTTTTTTTTTTTTTTTTTTTTTTGTACAAATATCATAAAAAAAGAGAATCTTTTTAAG
GTAATAATAGGCTACACTGCGACGTAAAAAAAAAAAAAAAAAAAAAAAAAAAAAAAAAAAAAAAAAAAAAAACATGTTTATAGTATTTTTTTCTCTTAGAAAAATTC

8,580 8,600 8,620 8,640 8,660

CAAGGATTTTCTTAACTTCTTCGGCGACAGCATCACCGACTTCGGTGGTACTGTTGGAACCACCTAAATCACCAGTTCTGATACCTGCATCCAAAACCTTTTTAACT
GTTCCTAAAAGAATTGAAGAAGCCGCTGTCGTAGTGGCTGAAGCCACCATGACAACCTTGGTGGATTTAGTGGTCAAGACTATGGACGTAGGTTTTGGAAAAATTGA

8,680 8,700 8,720 8,740 8,760

GCATCTTCAATGGCCTTACCTTCTTCAGGCAAGTTCAATGACAATTTCAACATCATTGCAGCAGACAAGATAGTGGCGATAGGGTCAACCTTATTCTTTGGCAAATC
CGTAGAAGTTACCGGAATGGAAGAAGTCCGTTCAAGTTACTGTTAAAGTTGTAGTAACGTCGTCTGTTCTATCACCGCTATCCCAGTTGGAATAAGAAACCGTTTAG

8,780 8,800 8,820 8,840 8,860 8,880

TGGAGCAGAACCGTGGCATGGTTCGTACAAACCAAATGCGGTGTTCTTGTCTGGCAAAGAGGCCAAGGACGCAGATGGCAACAAACCCAAGGAACCTGGGATAACGG
ACCTCGTCTTGGCACCGTACCAAGCATGTTTGGTTTACGCCACAAGAACAGACCGTTTCTCCGGTTCCTGCGTCTACCGTTGTTTGGGTTCCTTGGACCCTATTGCC

8,900 8,920 8,940 8,960 8,980

AGGCTTCATCGGAGATGATATCACCAAACATGTTGCTGGTGATTATAATACCATTTAGGTGGGTTGGGTTCTTAACTAGGATCATGGCGGCAGAATCAATCAATTGA
TCCGAAGTAGCCTCTACTATAGTGGTTTGTACAACGACCACTAATATTATGGTAAATCCACCCAACCCAAGAATTGATCCTAGTACCGCCGTCTTAGTTAGTTAACT

9,000 9,020 9,040 9,060 9,080

TGTTGAACCTTCAATGTAGGAAATTCGTTCTTGATGGTTTCCTCCACAGTTTTTCTCCATAATCTTGAAGAGGCCAAAACATTAGCTTTATCCAAGGACCAAATAGG
ACAACTTGGAAGTTACATCCTTTAAGCAAGAACTACCAAAGGAGGTGTCAAAAAGAGGTATTAGAACTTCTCCGGTTTTGTAATCGAAATAGGTTCCTGGTTTATCC
9,100 9,120 9,140 9,160 9,180 9,200

CAATGGTGGCTCATGTTGTAGGGCCATGAAAGCGGCCATTCTTGTGATTCTTTGCACTTCTGGAACGGTGTATTGTTCACTATCCCAAGCGACACCATCACCATCGT
GTTACCACCGAGTACAACATCCCGGTACTTTCGCCGGTAAGAACACTAAGAAACGTGAAGACCTTGCCACATAACAAGTGATAGGGTTCGCTGTGGTAGTGGTAGCA
9,220 9,240 9,260 9,280 9,300

CTTCCTTTCTCTTACCAAAGTAAATACCTCCCACTAATTCTCTGACAACAACGAAGTCAGTACCTTTAGCAAATTGTGGCTTGATTGGAGATAAGTCTAAAAGAGAG
GAAGGAAAGAGAATGGTTTCATTTATGGAGGGTGATTAAGAGACTGTTGTTGCTTCAGTCATGGAAATCGTTTAACACCGAACTAACCTCTATTCAGATTTTCTCTC
9,320 9,340 9,360 9,380 9,400

TCGGATGCAAAGTTACATGGTCTTAAGTTGGCGTACAATTGAAGTTCTTTACGGATTTTTAGTAAACCTTGTTCAGGTCTAACACTACCTGTACCCCATTTAGGACC
AGCCTACGTTTCAATGTACCAGAATTCAACCGCATGTTAACTTCAAGAAATGCCTAAAAATCATTTGGAACAAGTCCAGATTGTGATGGACATGGGGTAAATCCTGG
9,420 9,440 9,460 9,480 9,500 9,520

ACCCACAGCACCTAACAAAACGGCATCAACCTTCTTGGAGGCTTCCAGCGCCTCATCTGGAAGTGGGACACCTGTAGCATCGATAGCAGCACCACCAATTAAATGAT
TGGGTGTCGTGGATTGTTTTGCCGTAGTTGGAAGAACCTCCGAAGGTCGCGGAGTAGACCTTCACCCTGTGGACATCGTAGCTATCGTCGTGGTGGTTAATTTACTA
9,540 9,560 9,580 9,600 9,620

TTTCGAAATCGAACTTGACATTGGAACGAACATCAGAAATAGCTTTAAGAACCTTAATGGCTTCGGCTGTGATTTCTTGACCAACGTGGTCACCTGGCAAAACGACG
AAAGCTTTAGCTTGAACTGTAACCTTGCTTGTAGTCTTTATCGAAATTCTTGGAATTACCGAAGCCGACACTAAAGAACTGGTTGCACCAGTGGACCGTTTTGCTGC
9,640 9,660 9,680 9,700 9,720

ATCTTCTTAGGGGCAGACATTACAATGGTATATCCTTGAAATATATATAAAAAAAAAAAAAAAAAAAAAAAAAAAAAAATGCAGCTTCTCAATGATATTCGAATACG
TAGAAGAATCCCCGTCTGTAATGTTACCATATAGGAACTTTATATATATTTTTTTTTTTTTTTTTTTTTTTTTTTTTTTACGTCGAAGAGTTACTATAAGCTTATGC
9,740 9,760 9,780 9,800 9,820 9,840

CTTTGAGGAGATACAGCCTAATATCCGACAAACTGTTTTACAGATTTACGATCGTACTTGTTACCCATCATTGAATTTTGAACATCCGAACCTGGGAGTTTTCCCTG
GAAACTCCTCTATGTCGGATTATAGGCTGTTTGACAAAATGTCTAAATGCTAGCATGAACAATGGGTAGTAACTTAAAACTTGTAGGCTTGGACCCTCAAAAGGGAC
9,860 9,880 9,900 9,920 9,940

AAACAGATAGTATATTTGAACCTGTATAATAATATATAGTCTAGCGCTTTACGGAAGACAATGTATGTATTTCGGTTCCTGGAGAAACTATTGCATCTATTGCATAG
TTTGTCTATCATATAAACTTGGACATATTATTATATATCAGATCGCGAAATGCCTTCTGTTACATACATAAAGCCAAGGACCTCTTTGATAACGTAGATAACGTATC
9,960 9,980 10,000 10,020 10,040

GTAATCTTGCACGTCGCATCCCCGGTTCATTTTCTGCGTTTCCATCTTGCACTTCAATAGCATATCTTT
CATTAGAACGTGCAGCGTAGGGGCCAAGTAAAAGACGCAAAGGTAGAACGTGAAGTTATCGTATAGAAA
10,060 10,070 10,080 10,090 10,100 10,110 10,120

System 4 anti-lys Nb strain (9015 bp)

GTTAACGAAGCATCTGTGCTTCATTTTGTAGAACAAAAATGCAACGCGAGAGCGCTAATTTTTCAAACAAAGAATCTGAGCTGCATTTTTACAGAACAGAAATGCAA
CAATTGCTTCGTAGACACGAAGTAAAACATCTTGTTTTTACGTTGCGCTCTCGCGATTAAAAAGTTTGTTTCTTAGACTCGACGTAAAAATGTCTTGTCTTTACGTT
2 micron origin
20 40 60 80 100

CGCGAAAGCGCTATTTTACCAACGAAGAATCTGTGCTTCATTTTTGTAAAACAAAAATGCAACGCGAGAGCGCTAATTTTTCAAACAAAGAATCTGAGCTGCATTTT
GCGCTTTCGCGATAAAATGGTTGCTTCTTAGACACGAAGTAAAAACATTTTGTTTTTACGTTGCGCTCTCGCGATTAAAAAGTTTGTTTCTTAGACTCGACGTAAAA
2 micron origin
120 140 160 180 200

TACAGAACAGAAATGCAACGCGAGAGCGCTATTTTACCAACAAAGAATCTATACTTCTTTTTTGTTCTACAAAAATGCATCCCGAGAGCGCTATTTTTCTAACAAAG
ATGTCTTGTCTTTACGTTGCGCTCTCGCGATAAAATGGTTGTTTCTTAGATATGAAGAAAAAACAAGATGTTTTTACGTAGGGCTCTCGCGATAAAAAGATTGTTTC
2 micron origin
220 240 260 280 300 320

CATCTTAGATTACTTTTTTTCTCCTTTGTGCGCTCTATAATGCAGTCTCTTGATAACTTTTTGCACTGTAGGTCCGTTAAGGTTAGAAGAAGGCTACTTTGGTGTCT
GTAGAATCTAATGAAAAAAAGAGGAAACACGCGAGATATTACGTCAGAGAACTATTGAAAAACGTGACATCCAGGCAATTCCAATCTTCTTCCGATGAAACCACAGA
2 micron origin
340 360 380 400 420

ATTTTCTCTTCCATAAAAAAAGCCTGACTCCACTTCCCGCGTTTACTGATTACTAGCGAAGCTGCGGGTGCATTTTTTCAAGATAAAGGCATCCCCGATTATATTCT
TAAAAGAGAAGGTATTTTTTTCGGACTGAGGTGAAGGGCGCAAATGACTAATGATCGCTTCGACGCCCACGTAAAAAAGTTCTATTTCCGTAGGGGCTAATATAAGA
2 micron origin
440 460 480 500 520

ATACCGATGTGGATTGCGCATACTTTGTGAACAGAAAGTGATAGCGTTGATGATTCTTCATTGGTCAGAAAATTATGAACGGTTTCTTCTATTTTGTCTCTATATAC
TATGGCTACACCTAACGCGTATGAAACACTTGTCTTTCACTATCGCAACTACTAAGAAGTAACCAGTCTTTTAATACTTGCCAAAGAAGATAAAACAGAGATATATG
2 micron origin
540 560 580 600 620 640

TACGTATAGGAAATGTTTACATTTTCGTATTGTTTTCGATTCACTCTATGAATAGTTCTTACTACAATTTTTTTGTCTAAAGAGTAATACTAGAGATAAACATAAAA
ATGCATATCCTTTACAAATGTAAAAGCATAACAAAAGCTAAGTGAGATACTTATCAAGAATGATGTTAAAAAAACAGATTTCTCATTATGATCTCTATTTGTATTTT
2 micron origin
660 680 700 720 740

AATGTAGAGGTCGAGTTTAGATGCAAGTTCAAGGAGCGAAAGGTGGATGGGTAGGTTATATAGGGATATAGCACAGAGATATATAGCAAAGAGATACTTTTGAGCAA
TTACATCTCCAGCTCAAATCTACGTTCAAGTTCCTCGCTTTCCACCTACCCATCCAATATATCCCTATATCGTGTCTCTATATATCGTTTCTCTATGAAAACTCGTT
2 micron origin
760 780 800 820 840

TGTTTGTGGAAGCGGTATTCGCAATATTTTAGTAGCTCGTTACAGTCCGGTGCGTTTTTGGTTTTTTGAAAGTGCGTCTTCAGAGCGCTTTTGGTTTTCAAAAGCGC
ACAAACACCTTCGCCATAAGCGTTATAAAATCATCGAGCAATGTCAGGCCACGCAAAAACCAAAAAACTTTCACGCAGAAGTCTCGCGAAAACCAAAAGTTTTCGCG

2 micron origin

860 880 900 920 940 960

TCTGAAGTTCCTATACTTTCTAGCTAGAGAATAGGAACTTCGGAATAGGAACTTCAAAGCGTTTCCGAAAACGAGCGCTTCCGAAAATGCAACGCGAGCTGCGCACA
AGACTTCAAGGATATGAAAGATCGATCTCTTATCCTTGAAGCCTTATCCTTGAAGTTTCGCAAAGGCTTTTGCTCGCGAAGGCTTTTACGTTGCGCTCGACGCGTGT

2 micron origin

980 1,000 1,020 1,040 1,060

TACAGCTCACTGTTCACGTCGCACCTATATCTGCGTGTTGCCTGTATATATATATACATGAGAAGAACGGCATAGTGCGTGTTTATGCTTAAATGCGTACTTATATG
ATGTCGAGTGACAAGTGCAGCGTGGATATAGACGCACAACGGACATATATATATATGTACTCTTCTTGCCGTATCACGCACAAATACGAATTTACGCATGAATATAC

2 micron origin

1,080 1,100 1,120 1,140 1,160

CGTCTATTTATGTAGGATGAAAGGTAGTCTAGTACCTCCTGTGATATTATCCCATTCCATGCGGGGTATCGTATGCTTCCTTCAGCACTACCCTTTAGCTGTTCTAT
GCAGATAAATACATCCTACTTTCCATCAGATCATGGAGGACACTATAATAGGGTAAGGTACGCCCCATAGCATACGAAGGAAGTCGTGATGGGAAATCGACAAGATA

2 micron origin

1,180 1,200 1,220 1,240 1,260 1,280

ATGCTGCCACTCCTCAATTGGATTAGTCTCATCCTTCAATGCTATCATTTCCTTTGATATTGGATCGATCCGATGATAAGCTGTCAAACATGAGAATTGGGTAATAA
TACGACGGTGAGGAGTTAACCTAATCAGAGTAGGAAGTTACGATAGTAAAGGAAACTATAACCTAGCTAGGCTACTATTCGACAGTTTGTACTCTTAACCCATTATT

2 micron origin URA3

1,300 1,320 1,340 1,360 1,380

CTGATATAATTAAATTGAAGCTCTAATTTGTGAGTTTAGTATACATGCATTTACTTATAATACAGTTTTTTAGTTTTGCTGGCCGCATCTTCTCAAATATGCTTCCC
GACTATATTAATTTAACTTCGAGATTAAACACTCAAATCATATGTACGTAAATGAATATTATGTCAAAAAATCAAAACGACCGGCGTAGAAGAGTTTATACGAAGGG

URA3

1,400 1,420 1,440 1,460 1,480

AGCCTGCTTTTCTGTAACGTTCACCCTCTACCTTAGCATCCCTTCCCTTTGCAAATAGTCCTCTTCCAACAATAATAATGTCAGATCCTGTAGAGACCACATCATCC
TCGGACGAAAAGACATTGCAAGTGGGAGATGGAATCGTAGGGAAGGGAAACGTTTATCAGGAGAAGGTTGTTATTATTACAGTCTAGGACATCTCTGGTGTAGTAGG

URA3

1,500 1,520 1,540 1,560 1,580 1,600

ACGGTTCTATACTGTTGACCCAATGCGTCTCCCTTGTCATCTAAACCCACACCGGGTGTCATAATCAACCAATCGTAACCTTCATCTCTTCCACCCATGTCTCTTTG
TGCCAAGATATGACAACTGGGTTACGCAGAGGGAACAGTAGATTTGGGTGTGGCCCACAGTATTAGTTGGTTAGCATTGGAAGTAGAGAAGGTGGGTACAGAGAAAC

URA3

1,620 1,640 1,660 1,680 1,700

AGCAATAAAGCCGATAACAAAATCTTTGTCGCTCTTCGCAATGTCAACAGTACCCTTAGTATATTCTCCAGTAGATAGGGAGCCCTTGCATGACAATTCTGCTAACA
TCGTTATTTCGGCTATTGTTTTAGAAACAGCGAGAAGCGTTACAGTTGTCATGGGAATCATATAAGAGGTCATCTATCCCTCGGGAACGTACTGTTAAGACGATTGT
URA3
1,720 1,740 1,760 1,780 1,800

TCAAAAGGCCTCTAGGTTCCTTTGTTACTTCTTCTGCCGCCTGCTTCAAACCGCTAACAATACCTGGGCCCACCACACCGTGTGCATTCGTAATGTCTGCCCATTCT
AGTTTTCCGGAGATCCAAGGAAACAATGAAGAAGACGGCGGACGAAGTTTGGCGATTGTTATGGACCCGGGTGGTGTGGCACACGTAAGCATTACAGACGGGTAAGA
URA3
1,820 1,840 1,860 1,880 1,900 1,920

GCTATTCTGTATACACCCGCAGAGTACTGCAATTTGACTGTATTACCAATGTCAGCAAATTTTCTGTCTTCGAAGAGTAAAAAATTGTACTTGGCGGATAATGCCTT
CGATAAGACATATGTGGGCGTCTCATGACGTTAAACTGACATAATGGTTACAGTCGTTTAAAAGACAGAAGCTTCTCATTTTTTAACATGAACCGCCTATTACGGAA
URA3
1,940 1,960 1,980 2,000 2,020

TAGCGGCTTAACTGTGCCCTCCATGGAAAAATCAGTCAAGATATCCACATGTGTTTTTAGTAAACAAATTTTGGGACCTAATGCTTCAACTAACTCCAGTAATTCCT
ATCGCCGAATTGACACGGGAGGTACCTTTTTAGTCAGTTCTATAGGTGTACACAAAAATCATTTGTTTAAAACCCTGGATTACGAAGTTGATTGAGGTCATTAAGGA
URA3
2,040 2,060 2,080 2,100 2,120 2,140

TGGTGGTACGAACATCCAATGAAGCACACAAGTTTGTTTGCTTTTCGTGCATGATATTAAATAGCTTGGCAGCAACAGGACTAGGATGAGTAGCAGCACGTTCCTTA
ACCACCATGCTTGTAGGTTACTTCGTGTGTTCAAACAAACGAAAAGCACGTACTATAATTTATCGAACCGTCGTTGTCCTGATCCTACTCATCGTCGTGCAAGGAAT
URA3
2,160 2,180 2,200 2,220 2,240

TATGTAGCTTTCGACATGATTTATCTTCGTTTCCTGCATGTTTTTGTTCTGTGCAGTTGGGTTAAGAATACTGGGCAATTTCATGTTTCTTCAACACTACATATGCG
ATACATCGAAAGCTGTACTAAATAGAAGCAAAGGACGTACAAAAACAAGACACGTCAACCCAATTCTTATGACCCGTTAAAGTACAAAGAAGTTGTGATGTATACGC
URA3
2,260 2,280 2,300 2,320 2,340

TATATATACCAATCTAAGTCTGTGCTCCTTCCTTCGTTCTTCCTTCTGTTCGGAGATTACCGAATCAAAAAAATTTCAAAGAAACCGAAATCAAAAAAAAGAATAAA
ATATATATGGTTAGATTCAGACACGAGGAAGGAAGCAAGAAGGAAGACAAGCCTCTAATGGCTTAGTTTTTTTAAAGTTTCTTTGGCTTTAGTTTTTTTTCTTATTT
URA3
2,360 2,380 2,400 2,420 2,440 2,460

AAAAAAATGATGAATTGAATTGAAAAGCTAATTCTTGAAGACGAAAGGGCCTCGTGATACGCCTATTTTTATAGGTTAATGTCATGATAATAATGGTTTCTTAGACG
TTTTTTTACTACTTAACTTAACTTTTCGATTAAGAACTTCTGCTTTCCCGGAGCACTATGCGGATAAAAATATCCAATTACAGTACTATTATTACCAAAGAATCTGC
URA3
2,480 2,500 2,520 2,540 2,560

TCAGGTGGCACTTTTCGGGGAAATGTGCGCGGAACCCCTATTTGTTTATTTTTCTAAATACATTCAAATATGTATCCGCTCATGAGACAATAACCCTGATAAATGCT
AGTCCACCGTGAAAAGCCCCTTTACACGCGCCTTGGGGATAAACAAATAAAAAGATTTATGTAAGTTTATACATAGGCGAGTACTCTGTTATTGGGACTATTTACGA
2,580 2,600 2,620 2,640 2,660

TCAATAATATTGAAAAAGGAAGAGTATGAGTATTCAACATTTCCGTGTCGCCCTTATTCCCTTTTTTGCGGCATTTTGCCTTCCTGTTTTTGCTCACCCAGAAACGC
AGTTATTATAACTTTTTCCTTCTCATACTCATAAGTTGTAAAGGCACAGCGGGAATAAGGGAAAAAACGCCGTAAAACGGAAGGACAAAAACGAGTGGGTCTTTGCG

2,680 2,700 2,720 2,740 2,760 2,780

TGGTGAAAGTAAAAGATGCTGAAGATCAGTTGGGTGCACGAGTGGGTTACATCGAACTGGATCTCAACAGCGGTAAGATCCTTGAGAGTTTTCGCCCCGAAGAACGT
ACCACTTTCATTTTCTACGACTTCTAGTCAACCCACGTGCTCACCCAATGTAGCTTGACCTAGAGTTGTCGCCATTCTAGGAACTCTCAAAAGCGGGGCTTCTTGCA

2,800 2,820 2,840 2,860 2,880

TTTCCAATGATGAGCACTTTTAAAGTTCTGCTATGTGGCGCGGTATTATCCCGTATTGACGCCGGGCAAGAGCAACTCGGTCGCCGCATACACTATTCTCAGAATGA
AAAGGTTACTACTCGTGAAAATTTCAAGACGATACACCGCGCCATAATAGGGCATAACTGCGGCCCGTTCTCGTTGAGCCAGCGGCGTATGTGATAAGAGTCTTACT

AmpR

2,900 2,920 2,940 2,960 2,980

CTTGGTTGAGTACTCACCAGTCACAGAAAAGCATCTTACGGATGGCATGACAGTAAGAGAATTATGCAGTGCTGCCATAACCATGAGTGATAACACTGCGGCCAACT
GAACCAACTCATGAGTGGTCAGTGTCTTTTCGTAGAATGCCTACCGTACTGTCATTCTCTTAATACGTCACGACGGTATTGGTACTCACTATTGTGACGCCGGTTGA

AmpR

3,000 3,020 3,040 3,060 3,080 3,100

TACTTCTGACAACGATCGGAGGACCGAAGGAGCTAACCGCTTTTTTGCACAACATGGGGGATCATGTAACTCGCCTTGATCGTTGGGAACCGGAGCTGAATGAAGCC
ATGAAGACTGTTGCTAGCCTCCTGGCTTCCTCGATTGGCGAAAAAACGTGTTGTACCCCCTAGTACATTGAGCGGAACTAGCAACCCTTGGCCTCGACTTACTTCGG

AmpR

3,120 3,140 3,160 3,180 3,200

ATACCAAACGACGAGCGTGACACCACGATGCCTGTAGCAATGGCAACAACGTTGCGCAAACTATTAACTGGCGAACTACTTACTCTAGCTTCCCGGCAACAATTAAT
TATGGTTTGCTGCTCGCACTGTGGTGCTACGGACATCGTTACCGTTGTTGCAACGCGTTTGATAATTGACCGCTTGATGAATGAGATCGAAGGGCCGTTGTTAATTA

AmpR

3,220 3,240 3,260 3,280 3,300

AGACTGGATGGAGGCGGATAAAGTTGCAGGACCACTTCTGCGCTCGGCCCTTCCGGCTGGCTGGTTTATTGCTGATAAATCTGGAGCCGGTGAGCGTGGGTCTCGCG
TCTGACCTACCTCCGCCTATTTCAACGTCCTGGTGAAGACGCGAGCCGGGAAGGCCGACCGACCAAATAACGACTATTTAGACCTCGGCCACTCGCACCCAGAGCGC

AmpR

3,320 3,340 3,360 3,380 3,400 3,420

GTATCATTGCAGCACTGGGGCCAGATGGTAAGCCCTCCCGTATCGTAGTTATCTACACGACGGGGAGTCAGGCAACTATGGATGAACGAAATAGACAGATCGCTGAG
CATAGTAACGTCGTGACCCCGGTCTACCATTCGGGAGGGCATAGCATCAATAGATGTGCTGCCCCTCAGTCCGTTGATACCTACTTGCTTTATCTGTCTAGCGACTC

AmpR

3,440 3,460 3,480 3,500 3,520

ATAGGTGCCTCACTGATTAAGCATTGGTAACTGTCAGACCAAGTTTACTCATATATACTTTAGATTGATTTAAAACTTCATTTTTAATTTAAAAGGATCTAGGTGAA
TATCCACGGAGTGACTAATTCGTAACCATTGACAGTCTGGTTCAAATGAGTATATATGAAATCTAACTAAATTTTGAAGTAAAAATTAAATTTTCCTAGATCCACTT

AmpR

3,540 3,560 3,580 3,600 3,620

GATCCTTTTTGATAATCTCATGACCAAAATCCCTTAACGTGAGTTTTCGTTCCACTGAGCGTCAGACCCCGTAGAAAAGATCAAAGGATCTTCTTGAGATCCTTTTT
CTAGGAAAAACTATTAGAGTACTGGTTTTAGGGAATTGCACTCAAAAGCAAGGTGACTCGCAGTCTGGGGCATCTTTTCTAGTTTCCTAGAAGAACTCTAGGAAAAA
ColE1 origin
3,640 3,660 3,680 3,700 3,720 3,740

TTCTGCGCGTAATCTGCTGCTTGCAAACAAAAAAACCACCGCTACCAGCGGTGGTTTGTTTGCCGGATCAAGAGCTACCAACTCTTTTTCCGAAGGTAACTGGCTTC
AAGACGCGCATTAGACGACGAACGTTTGTTTTTTTGGTGGCGATGGTCGCCACCAAACAAACGGCCTAGTTCTCGATGGTTGAGAAAAAGGCTTCCATTGACCGAAG
ColE1 origin
3,760 3,780 3,800 3,820 3,840

AGCAGAGCGCAGATACCAAATACTGTCCTTCTAGTGTAGCCGTAGTTAGGCCACCACTTCAAGAACTCTGTAGCACCGCCTACATACCTCGCTCTGCTAATCCTGTT
TCGTCTCGCGTCTATGGTTTATGACAGGAAGATCACATCGGCATCAATCCGGTGGTGAAGTTCTTGAGACATCGTGGCGGATGTATGGAGCGAGACGATTAGGACAA
ColE1 origin
3,860 3,880 3,900 3,920 3,940

ACCAGTGGCTGCTGCCAGTGGCGATAAGTCGTGTCTTACCGGGTTGGACTCAAGACGATAGTTACCGGATAAGGCGCAGCGGTCGGGCTGAACGGGGGGTTCGTGCA
TGGTCACCGACGACGGTCACCGCTATTCAGCACAGAATGGCCCAACCTGAGTTCTGCTATCAATGGCCTATTCCGCGTCGCCAGCCCGACTTGCCCCCCAAGCACGT
ColE1 origin
3,960 3,980 4,000 4,020 4,040 4,060

CACAGCCCAGCTTGGAGCGAACGACCTACACCGAACTGAGATACCTACAGCGTGAGCTATGAGAAAGCGCCACGCTTCCCGAAGGGAGAAAGGCGGACAGGTATCCG
GTGTCGGGTCGAACCTCGCTTGCTGGATGTGGCTTGACTCTATGGATGTCGCACTCGATACTCTTTCGCGGTGCGAAGGGCTTCCCTCTTTCCGCCTGTCCATAGGC
ColE1 origin
4,080 4,100 4,120 4,140 4,160

GTAAGCGGCAGGGTCGGAACAGGAGAGCGCACGAGGGAGCTTCCAGGGGGAAACGCCTGGTATCTTTATAGTCCTGTCGGGTTTCGCCACCTCTGACTTGAGCGTCG
CATTCGCCGTCCCAGCCTTGTCCTCTCGCGTGCTCCCTCGAAGGTCCCCCTTTGCGGACCATAGAAATATCAGGACAGCCCAAAGCGGTGGAGACTGAACTCGCAGC
ColE1 origin
4,180 4,200 4,220 4,240 4,260 4,280

ATTTTTGTGATGCTCGTCAGGGGGGCGGAGCCTATGGAAAAACGCCAGCAACGCGGCCTTTTTACGGTTCCTGGCCTTTTGCTGGCCTTTTGCTCACATGTTCTTTC
TAAAAACACTACGAGCAGTCCCCCCGCCTCGGATACCTTTTTGCGGTCGTTGCGCCGGAAAAATGCCAAGGACCGGAAAACGACCGGAAAACGAGTGTACAAGAAAG
ColE1 origin
4,300 4,320 4,340 4,360 4,380

CTGCGTTATCCCCTGATTCTGTGGATAACCGTATTACCGCCTTTGAGTGAGCTGATACCGCTCGCCGCAGCCGAACGACCGAGCGCAGCGAGTCAGTGAGCGAGGAA
GACGCAATAGGGGACTAAGACACCTATTGGCATAATGGCGGAAACTCACTCGACTATGGCGAGCGGCGTCGGCTTGCTGGCTCGCGTCGCTCAGTCACTCGCTCCTT
4,400 4,420 4,440 4,460 4,480

GCGGAAGAGCGCCCAATACGCAAACCGCCTCTCCCCGCGCGTTGGCCGATTCATTAATGCAGCTGGCACGACAGGTTTCCCGACTGGAAAGCGGGCAGTGAGCGCAA
CGCCTTCTCGCGGGTTATGCGTTTGGCGGAGAGGGGCGCGCAACCGGCTAAGTAATTACGTCGACCGTGCTGTCCAAAGGGCTGACCTTTCGCCCGTCACTCGCGTT
4,500 4,520 4,540 4,560 4,580 4,600

CGCAATTAATGTGAGTTAGCTCACTCATTAGGCACCCCAGGCTTTACACTTTATGCTTCCGGCTCGTATGTTGTGTGGAATTGTGAGCGGATAACAATTTCACACAG
GCGTTAATTACACTCAATCGAGTGAGTAATCCGTGGGGTCCGAAATGTGAAATACGAAGGCCGAGCATACAACACACCTTAACACTCGCCTATTGTTAAAGTGTGTC

lac promoter | LacO

4,620 4,640 4,660 4,680 4,700

GAAACAGCTATGACCATGATTACGCCAAGCTTCGGATTAGAAGCCGCCGAGCGGGTGACAGCCCTCCGAAGGAAGACTCTCCTCCGTGCGTCCTCGTCTTCACCGGT
CTTTGTCGATACTGGTACTAATGCGGTTCGAAGCCTAATCTTCGGCGGCTCGCCCACTGTCGGGAGGCTTCCTTCTGAGAGGAGGCACGCAGGAGCAGAAGTGGCCA

M13-rev | GAL1 promoter

4,720 4,740 4,760 4,780 4,800

CGCGTTCCTGAAACGCAGATGTGCCTCGCGCCGCACTGCTCCGAACAATAAAGATTCTACAATACTAGCTTTTATGGTTATGAAGAGGAAAAATTGGCAGTAACCTG
GCGCAAGGACTTTGCGTCTACACGGAGCGCGGCGTGACGAGGCTTGTTATTTCTAAGATGTTATGATCGAAAATACCAATACTTCTCCTTTTTAACCGTCATTGGAC

GAL1 promoter

4,820 4,840 4,860 4,880 4,900 4,920

GCCCCACAAACCTTCAAATGAACGAATCAAATTAACAACCATAGGATGATAATGCGATTAGTTTTTTAGCCTTATTTCTGGGGTAATTAATCAGCGAAGCGATGATT
CGGGGTGTTTGGAAGTTTACTTGCTTAGTTTAATTGTTGGTATCCTACTATTACGCTAATCAAAAAATCGGAATAAAGACCCCATTAATTAGTCGCTTCGCTACTAA

GAL1 promoter

4,940 4,960 4,980 5,000 5,020

TTTGATCTATTAACAGATATATAAATGCAAAAACTGCATAACCACTTTAACTAATACTTTCAACATTTTCGGTTTGTATTACTTCTTATTCAAATGTAATAAAAGTA
AAACTAGATAATTGTCTATATATTTACGTTTTTGACGTATTGGTGAAATTGATTATGAAAGTTGTAAAAGCCAAACATAATGAAGAATAAGTTTACATTATTTTCAT

GAL1 promoter

5,040 5,060 5,080 5,100 5,120

TCAACAAAAAATTGTTAATATACCTCTATACTTTAACGTCAAGGAGAAAAAACCCCGGATCGAATTCAACCCTCACTAAAGGGCGGCCGCCATGAGATTCCCATCTA
AGTTGTTTTTTAACAATTATATGGAGATATGAAATTGCAGTTCCTCTTTTTTGGGGCCTAGCTTAAGTTGGGAGTGATTTCCCGCCGGCGGTACTCTAAGGGTAGAT

1 2 3 4 5
M R F P S

GAL1 promoter

5,140 5,160 5,180 5,200 5,220 5,240

TCTTCACCGCTGTTTTGTTCGCTGCTTCTTCTGCTTTGGCTGCTCCAGCTAACACCACCACCGAAGACGAAACCGCTCAAATCCCAGCTGAAGCTGTTATCGACTAC
AGAAGTGGCGACAAAACAAGCGACGAAGAAGACGAAACCGACGAGGTCGATTGTGGTGGTGGCTTCTGCTTTGGCGAGTTTAGGGTCGACTTCGACAATAGCTGATG

6 7 8 9 10 11 12 13 14 15 16 17 18 19 20 21 22 23 24 25 26 27 28 29 30 31 32 33 34 35 36 37 38 39 40 41
I F T A V L F A A S S A L A A P A N T T T E D E T A Q I P A E A V I D Y

Mating factor alpha leader

5,260 5,280 5,300 5,320 5,340

TCTGACTTGGAAGGTGACTTCGACGCTGCTGCTTTGCCATTGTCTAACTCTACCAACAACGGTTTGTCTTCTACCAACACCACCATCGCTTCTATCGCTGCTAAGGA
AGACTGAACCTTCCACTGAAGCTGCGACGACGAAACGGTAACAGATTGAGATGGTTGTTGCCAAACAGAAGATGGTTGTGGTGGTAGCGAAGATAGCGACGATTCCT
42 43 44 45 46 47 48 49 50 51 52 53 54 55 56 57 58 59 60 61 62 63 64 65 66 67 68 69 70 71 72 73 74 75 76 77
S D L E G D F D A A A L P L S N S T N N G L S S T N T T I A S I A A K E

Mating factor alpha leader

5,360 5,380 5,400 5,420 5,440

AGAAGGTGTTCAATTGGACAAGAGAGAAagatctggcggccgcgcatgcgctagcctcgagGATGTGCAGTTGCAAGCATCCGGCGGCGGTTCTGTTCAGGCTGGTG
TCTTCCACAAGTTAACCTGTTCTCTCTTtctagaccgccggcgcgtacgcgatcggagctcCTACACGTCAACGTTCGTAGGCCGCCGCCAAGACAAGTCCGACCAC
78 79 80 81 82 83 84 85 86 ... 2 4 6 8 10 12 14
E G V Q L D K R E ... D V Q L Q A S G G G S V Q A G

anti-lys Nb

5,460 5,480 5,500 5,520 5,540 5,560

GTTCTCTAAGATTAAGTTGTGCTGCTTCAGGTTATACCATCGGCCCATACTGCATGGGTTGGTTTCGTCAAGCTCCAGGCAAAGAAAGGGAGGGAGTCGCAGCCATT
CAAGAGATTCTAATTCAACACGACGAAGTCCAATATGGTAGCCGGGTATGACGTACCCAACCAAAGCAGTTCGAGGTCCGTTTCTTTCCCTCCCTCAGCGTCGGTAA
16 18 20 22 24 26 28 30 32 34 36 38 40 42 44 46 48 50
G S L R L S C A A S G Y T I G P Y C M G W F R Q A P G K E R E G V A A I

anti-lys Nb

5,580 5,600 5,620 5,640 5,660

AACATGGGCGGCGGTATCACATATTATGCAGACTCCGTAAAAGGTAGATTTACTATCTCTCAGGACAATGCTAAGAACACGGTCTATCTGTTAATGAATTCATTGGA
TTGTACCCGCCGCCATAGTGTATAATACGTCTGAGGCATTTTCCATCTAAATGATAGAGAGTCCTGTTACGATTCTTGTGCCAGATAGACAATTACTTAAGTAACCT
52 54 56 58 60 62 64 66 68 70 72 74 76 78 80 82 84 86
N M G G G I T Y Y A D S V K G R F T I S Q D N A K N T V Y L L M N S L E

anti-lys Nb

5,680 5,700 5,720 5,740 5,760

GCCCGAGGACACTGCCATATACTACTGTGCCGCAGATTCAACCATTTACGCTTCCTATTACGAATGCGGACACGGATTAAGTACTGGAGGTTACGGTTATGATTCCT
CGGGCTCCTGTGACGGTATATGATGACACGGCGTCTAAGTTGGTAAATGCGAAGGATAATGCTTACGCCTGTGCCTAATTCATGACCTCCAATGCCAATACTAAGGA
88 90 92 94 96 98 100 102 104 106 108 110 112 114 116 118 120 122
P E D T A I Y Y C A A D S T I Y A S Y Y E C G H G L S T G G Y G Y D S

anti-lys Nb

5,780 5,800 5,820 5,840 5,860 5,880

GGGGTCAAGGCACACAAGTCACGGTATCATCAgaatccTACCCATACGATGTTCCAGATTACGCTGCTGGAGGTTCGAGCGCCAAAAGCTCTTTTATCTCAACCACT
CCCCAGTTCCGTGTGTTCAGTGCCATAGTAGTcttaggATGGGTATGCTACAAGGTCTAATGCGACGACCTCCAAGCTCGCGGTTTTCGAGAAAATAGAGTTGGTGA
124 126 128 130 132 ... 2 4 6 8 10
W G Q G T Q V T V S S ... S A K S S F I S T T

anti-lys Nb — HA tag — 3' Half of α-agglutinin

5,900 5,920 5,940 5,960 5,980

ACTACTGATTTAACAAGTATAAACACTAGTGCGTATTCCACTGGATCCATTTCCACAGTAGAAACAGGCAATCGAACTACATCAGAAGTGATCAGTCATGTGGTGAC
TGATGACTAAATTGTTCATATTTGTGATCACGCATAAGGTGACCTAGGTAAAGGTGTCATCTTTGTCCGTTAGCTTGATGTAGTCTTCACTAGTCAGTACACCACTG
12 14 16 18 20 22 24 26 28 30 32 34 36 38 40 42 44 46
T T D L T S I N T S A Y S T G S I S T V E T G N R T T S E V I S H V V T
3' Half of α-agglutinin
6,000 6,020 6,040 6,060 6,080

TACCAGCACAAAACTGTCTCCAACTGCTACTACCAGCCTGACAATTGCACAAACCAGTATCTATTCTACTGACTCATATATCACAGTAGGAACAGATATTCACACCA
ATGGTCGTGTTTTGACAGAGGTTGACGATGATGGTCGGACTGTTAACGTGTTTGGTCATAGATAAGATGACTGAGTATATAGTGTCATCCTTGTCTATAAGTGTGGT
48 50 52 54 56 58 60 62 64 66 68 70 72 74 76 78 80
T S T K L S P T A T T S L T I A Q T S I Y S T D S Y I T V G T D I H T
3' Half of α-agglutinin
6,100 6,120 6,140 6,160 6,180 6,200

CATCAGAAGTGATTAGTGATGTGGAAACCATTAGCAGAGAAACAGCTTCGACCGTTGTAGCCGCTCCAACCTCAACAACTGGATGGACAGGCGCTATGAATACTTAC
GTAGTCTTCACTAATCACTACACCTTTGGTAATCGTCTCTTTGTCGAAGCTGGCAACATCGGCGAGGTTGGAGTTGTTGACCTACCTGTCCGCGATACTTATGAATG
82 84 86 88 90 92 94 96 98 100 102 104 106 108 110 112 114 116
T S E V I S D V E T I S R E T A S T V V A A P T S T T G W T G A M N T Y
3' Half of α-agglutinin
6,220 6,240 6,260 6,280 6,300

ATCTCGCAATTTACATCCTCTTCTTTCGCAACAATCAACAGCACACCAATAATCTCTTCATCAGCAGTATTTGAAACCTCAGATGCTTCAATTGTCAATGTGCACAC
TAGAGCGTTAAATGTAGGAGAAGAAAGCGTTGTTAGTTGTCGTGTGGTTATTAGAGAAGTAGTCGTCATAAACTTTGGAGTCTACGAAGTTAACAGTTACACGTGTG
118 120 122 124 126 128 130 132 134 136 138 140 142 144 146 148 150 152
I S Q F T S S S F A T I N S T P I I S S S A V F E T S D A S I V N V H T
3' Half of α-agglutinin
6,320 6,340 6,360 6,380 6,400 6,420

TGAAAATATCACGAATACTGCTGCTGTTCCATCTGAAGAGCCCACTTTTGTAAATGCCACGAGAAACTCCTTAAATTCCTTCTGCAGCAGCAAACAGCCATCCAGTC
ACTTTTATAGTGCTTATGACGACGACAAGGTAGACTTCTCGGGTGAAAACATTTACGGTGCTCTTTGAGGAATTTAAGGAAGACGTCGTCGTTTGTCGGTAGGTCAG
154 156 158 160 162 164 166 168 170 172 174 176 178 180 182 184 186 188
E N I T N T A A V P S E E P T F V N A T R N S L N S F C S S K Q P S S
3' Half of α-agglutinin
6,440 6,460 6,480 6,500 6,520

CCTCATCTTATACGTCTTCCCCACTCGTATCGTCCCTCTCCGTAAGCAAAACATTACTAAGCACCAGTTTTACGCCTTCTGTGCCAACATCTAATACATATATCAAA
GGAGTAGAATATGCAGAAGGGGTGAGCATAGCAGGGAGAGGCATTCGTTTTGTAATGATTCGTGGTCAAAATGCGGAAGACACGGTTGTAGATTATGTATATAGTTT
190 192 194 196 198 200 202 204 206 208 210 212 214 216 218 220 222 224
P S S Y T S S P L V S S L S V S K T L L S T S F T P S V P T S N T Y I K
3' Half of α-agglutinin
6,540 6,560 6,580 6,600 6,620

```
ACGAAAAATACGGGTTACTTTGAGCACACGGCTTTGACAACATCTTCAGTTGGCCTTAATTCTTTTAGTGAAACAGCAGTCTCATCTCAGGGAACGAAAATTGACAC
TGCTTTTTATGCCCAATGAAACTCGTGTGCCGAAACTGTTGTAGAAGTCAACCGGAATTAAGAAAATCACTTTGTCGTCAGAGTAGAGTCCCTTGCTTTTAACTGTG
    226    228    230    232    234    236    238    240    242    244    246    248    250    252    254    256    258    260
 T  K  N  T  G  Y  F  E  H  T  A  L  T  T  S  S  V  G  L  N  S  F  S  E  T  A  V  S  S  Q  G  T  K  I  D  T
```

3′ Half of α-agglutinin

6,640 6,660 6,680 6,700 6,720 6,740

```
CTTTTTAGTGTCATCCTTGATCGCATATCCTTCTTCTGCATCAGGAAGCCAATTGTCCGGTATCCAACAGAATTTCACATCAACTTCTCTCATGATTTCAACCTATG
GAAAAATCACAGTAGGAACTAGCGTATAGGAAGAAGACGTAGTCCTTCGGTTAACAGGCCATAGGTTGTCTTAAAGTGTAGTTGAAGAGAGTACTAAAGTTGGATAC
     262    264    266    268    270    272    274    276    278    280    282    284    286    288    290    292    294
  F  L  V  S  S  L  I  A  Y  P  S  S  A  S  G  S  Q  L  S  G  I  Q  Q  N  F  T  S  T  S  L  M  I  S  T  Y
```

3′ Half of α-agglutinin

6,760 6,780 6,800 6,820 6,840

```
AAGGTAAAGCGTCTATATTTTTCTCAGCTGAGCTCGGTTCGATCATTTTTCTGCTTTTGTCGTACCTGCTATTCTAAAACGGGTACTGTACAGTTAGTACATTGAGT
TTCCATTTCGCAGATATAAAAAGAGTCGACTCGAGCCAAGCTAGTAAAAAGACGAAAACAGCATGGACGATAAGATTTTGCCCATGACATGTCAATCATGTAACTCA
296    298    300    302    304    306    308    310    312    314    316    318    320
 E  G  K  A  S  I  F  F  S  A  E  L  G  S  I  I  F  L  L  L  S  Y  L  L  F  *
```

3′ Half of α-agglutinin

6,860 6,880 6,900 6,920 6,940

```
CGAAATATACGAAATTATTGTTCATAATTTTCATCCTGGCTCTTTTTTTCTTCAACCATAGTTAAATGGACAGTTCATATCTTAACTCTAATAATACTTTTCTAGTT
GCTTTATATGCTTTAATAACAAGTATTAAAAGTAGGACCGAGAAAAAAAGAAGTTGGTATCAATTTACCTGTCAAGTATAGAATTGAGATTATTATGAAAAGATCAA
```

3′ Half of α-agglutinin

6,960 6,980 7,000 7,020 7,040 7,060

```
CTTATCCTTTTCCGTCTCACCGCAGATTTTATCATAGTATTAAATTTATATTTTGTTCGTAAAAAGAAAAATTTGTGAGCGTTACCGCTCGTTTCATTACCCGAAGG
GAATAGGAAAAGGCAGAGTGGCGTCTAAAATAGTATCATAATTTAAATATAAAACAAGCATTTTTCTTTTTAAACACTCGCAATGGCGAGCAAAGTAATGGGCTTCC
```

3′ Half of α-agglutinin

7,080 7,100 7,120 7,140 7,160

```
CTGTTTCAGTAGACCACTGATTAAGTAAGTAGATGAAAAAATTTCATCACCATGAAAGAGTTCGATGAGAGCTACTTTTTCAAATGCTTAACAGCTAACCGCCATTC
GACAAAGTCATCTGGTGACTAATTCATTCATCTACTTTTTTAAAGTAGTGGTACTTTCTCAAGCTACTCTCGATGAAAAAGTTTACGAATTGTCGATTGGCGGTAAG
```

3′ Half of α-agglutinin

7,180 7,200 7,220 7,240 7,260

```
AATAATGTTACGCTCTCTTCATTCTGCGGCTACGTTATCTAACAAGAGGTTTTACTCTCTCATATCTCATTCAAATAGAAAGAACATAATCAAAGGTACCGCGATGT
TTATTACAATGCGAGAGAAGTAAGACGCCGATGCAATAGATTGTTCTCCAAAATGAGAGAGTATAGAGTAAGTTTATCTTTCTTGTATTAGTTTCCATGGCGCTACA
```

3′ Half of α-agglutinin

7,280 7,300 7,320 7,340 7,360 7,380

AGTAAAACTAGCTAGACCGAGAAAGAGACTAGAAATGCAAAAGGCACTTCTACAATGGCTGCCATCATTATTATCCGATGTGACGCTGCATTTTTTTTTTTTTTTTT
TCATTTTGATCGATCTGGCTCTTTCTCTGATCTTTACGTTTTCCGTGAAGATGTTACCGACGGTAGTAATAATAGGCTACACTGCGACGTAAAAAAAAAAAAAAAAA

7,400 7,420 7,440 7,460 7,480

TTTTTTTTTTTTTTTTTTTTTTTTTTTTTTGTACAAATATCATAAAAAAAGAGAATCTTTTTAAGCAAGGATTTTCTTAACTTCTTCGGCGACAGCATCACCGACTT
AAAAAAAAAAAAAAAAAAAAAAAAAAAAAACATGTTTATAGTATTTTTTTCTCTTAGAAAAATTCGTTCCTAAAAGAATTGAAGAAGCCGCTGTCGTAGTGGCTGAA

7,500 7,520 7,540 7,560 7,580

CGGTGGTACTGTTGGAACCACCTAAATCACCAGTTCTGATACCTGCATCCAAAACCTTTTTAACTGCATCTTCAATGGCCTTACCTTCTTCAGGCAAGTTCAATGAC
GCCACCATGACAACCTTGGTGGATTTAGTGGTCAAGACTATGGACGTAGGTTTTGGAAAAATTGACGTAGAAGTTACCGGAATGGAAGAAGTCCGTTCAAGTTACTG

7,600 7,620 7,640 7,660 7,680 7,700

AATTTCAACATCATTGCAGCAGACAAGATAGTGGCGATAGGGTCAACCTTATTCTTTGGCAAATCTGGAGCAGAACCGTGGCATGGTTCGTACAAACCAAATGCGGT
TTAAAGTTGTAGTAACGTCGTCTGTTCTATCACCGCTATCCCAGTTGGAATAAGAAACCGTTTAGACCTCGTCTTGGCACCGTACCAAGCATGTTTGGTTTACGCCA

7,720 7,740 7,760 7,780 7,800

GTTCTTGTCTGGCAAAGAGGCCAAGGACGCAGATGGCAACAAACCCAAGGAACCTGGGATAACGGAGGCTTCATCGGAGATGATATCACCAAACATGTTGCTGGTGA
CAAGAACAGACCGTTTCTCCGGTTCCTGCGTCTACCGTTGTTTGGGTTCCTTGGACCCTATTGCCTCCGAAGTAGCCTCTACTATAGTGGTTTGTACAACGACCACT

7,820 7,840 7,860 7,880 7,900

TTATAATACCATTTAGGTGGGTTGGGTTCTTAACTAGGATCATGGCGGCAGAATCAATCAATTGATGTTGAACCTTCAATGTAGGAAATTCGTTCTTGATGGTTTCC
AATATTATGGTAAATCCACCCAACCCAAGAATTGATCCTAGTACCGCCGTCTTAGTTAGTTAACTACAACTTGGAAGTTACATCCTTTAAGCAAGAACTACCAAAGG

7,920 7,940 7,960 7,980 8,000 8,020

TCCACAGTTTTTCTCCATAATCTTGAAGAGGCCAAAACATTAGCTTTATCCAAGGACCAAATAGGCAATGGTGGCTCATGTTGTAGGGCCATGAAAGCGGCCATTCT
AGGTGTCAAAAAGAGGTATTAGAACTTCTCCGGTTTTGTAATCGAAATAGGTTCCTGGTTTATCCGTTACCACCGAGTACAACATCCCGGTACTTTCGCCGGTAAGA

8,040 8,060 8,080 8,100 8,120

TGTGATTCTTTGCACTTCTGGAACGGTGTATTGTTCACTATCCCAAGCGACACCATCACCATCGTCTTCCTTTCTCTTACCAAAGTAAATACCTCCCACTAATTCTC
ACACTAAGAAACGTGAAGACCTTGCCACATAACAAGTGATAGGGTTCGCTGTGGTAGTGGTAGCAGAAGGAAAGAGAATGGTTTCATTTATGGAGGGTGATTAAGAG

8,140 8,160 8,180 8,200 8,220

TGACAACAACGAAGTCAGTACCTTTAGCAAATTGTGGCTTGATTGGAGATAAGTCTAAAAGAGAGTCGGATGCAAAGTTACATGGTCTTAAGTTGGCGTACAATTGA
ACTGTTGTTGCTTCAGTCATGGAAATCGTTTAACACCGAACTAACCTCTATTCAGATTTTCTCTCAGCCTACGTTTCAATGTACCAGAATTCAACCGCATGTTAACT

8,240 8,260 8,280 8,300 8,320 8,340

AGTTCTTTACGGATTTTTAGTAAACCTTGTTCAGGTCTAACACTACCTGTACCCCATTTAGGACCACCCACAGCACCTAACAAAACGGCATCAACCTTCTTGGAGGC
TCAAGAAATGCCTAAAAATCATTTGGAACAAGTCCAGATTGTGATGGACATGGGGTAAATCCTGGTGGGTGTCGTGGATTGTTTTGCCGTAGTTGGAAGAACCTCCG

8,360 8,380 8,400 8,420 8,440

TTCCAGCGCCTCATCTGGAAGTGGGACACCTGTAGCATCGATAGCAGCACCACCAATTAAATGATTTTCGAAATCGAACTTGACATTGGAACGAACATCAGAAATAG
AAGGTCGCGGAGTAGACCTTCACCCTGTGGACATCGTAGCTATCGTCGTGGTGGTTAATTTACTAAAAGCTTTAGCTTGAACTGTAACCTTGCTTGTAGTCTTTATC

8,460 8,480 8,500 8,520 8,540 8,560

```
CTTTAAGAACCTTAATGGCTTCGGCTGTGATTTCTTGACCAACGTGGTCACCTGGCAAAACGACGATCTTCTTAGGGGCAGACATTACAATGGTATATCCTTGAAAT
GAAATTCTTGGAATTACCGAAGCCGACACTAAAGAACTGGTTGCACCAGTGGACCGTTTTGCTGCTAGAAGAATCCCCGTCTGTAATGTTACCATATAGGAACTTTA
8,580 8,600 8,620 8,640 8,660

ATATATAAAAAAAAAAAAAAAAAAAAAAAAAAAAAAATGCAGCTTCTCAATGATATTCGAATACGCTTTGAGGAGATACAGCCTAATATCCGACAAACTGTTTTACA
TATATATTTTTTTTTTTTTTTTTTTTTTTTTTTTTTTACGTCGAAGAGTTACTATAAGCTTATGCGAAACTCCTCTATGTCGGATTATAGGCTGTTTGACAAAATGT
8,680 8,700 8,720 8,740 8,760

GATTTACGATCGTACTTGTTACCCATCATTGAATTTTGAACATCCGAACCTGGGAGTTTTCCCTGAAACAGATAGTATATTTGAACCTGTATAATAATATATAGTCT
CTAAATGCTAGCATGAACAATGGGTAGTAACTTAAAACTTGTAGGCTTGGACCCTCAAAAGGGACTTTGTCTATCATATAAACTTGGACATATTATTATATATCAGA
8,780 8,800 8,820 8,840 8,860 8,880

AGCGCTTTACGGAAGACAATGTATGTATTTCGGTTCCTGGAGAAACTATTGCATCTATTGCATAGGTAATCTTGCACGTCGCATCCCCGGTTCATTTTCTGCGTTTC
TCGCGAAATGCCTTCTGTTACATACATAAAGCCAAGGACCTCTTTGATAACGTAGATAACGTATCCATTAGAACGTGCAGCGTAGGGGCCAAGTAAAAGACGCAAAG
8,900 8,920 8,940 8,960 8,980

CATCTTGCACTTCAATAGCATATCTTT
GTAGAACGTGAAGTTATCGTATAGAAA
8,990 9,000 9,010
```

System 4 control strain (8616 bp)

GTTAACGAAGCATCTGTGCTTCATTTTGTAGAACAAAAATGCAACGCGAGAGCGCTAATTTTTCAAACAAAGAATCTGAGCTGCATTTTTACAGAACAGAAATGCAA
CAATTGCTTCGTAGACACGAAGTAAAACATCTTGTTTTTACGTTGCGCTCTCGCGATTAAAAAGTTTGTTTCTTAGACTCGACGTAAAAATGTCTTGTCTTTACGTT

2 micron origin

20 40 60 80 100

CGCGAAAGCGCTATTTTACCAACGAAGAATCTGTGCTTCATTTTTGTAAAACAAAAATGCAACGCGAGAGCGCTAATTTTTCAAACAAAGAATCTGAGCTGCATTTT
GCGCTTTCGCGATAAAATGGTTGCTTCTTAGACACGAAGTAAAAACATTTTGTTTTTACGTTGCGCTCTCGCGATTAAAAAGTTTGTTTCTTAGACTCGACGTAAAA

2 micron origin

120 140 160 180 200

TACAGAACAGAAATGCAACGCGAGAGCGCTATTTTACCAACAAAGAATCTATACTTCTTTTTTGTTCTACAAAAATGCATCCCGAGAGCGCTATTTTTCTAACAAAG
ATGTCTTGTCTTTACGTTGCGCTCTCGCGATAAAATGGTTGTTTCTTAGATATGAAGAAAAAACAAGATGTTTTTACGTAGGGCTCTCGCGATAAAAAGATTGTTTC

2 micron origin

220 240 260 280 300 320

CATCTTAGATTACTTTTTTTCTCCTTTGTGCGCTCTATAATGCAGTCTCTTGATAACTTTTTGCACTGTAGGTCCGTTAAGGTTAGAAGAAGGCTACTTTGGTGTCT
GTAGAATCTAATGAAAAAAAGAGGAAACACGCGAGATATTACGTCAGAGAACTATTGAAAAACGTGACATCCAGGCAATTCCAATCTTCTTCCGATGAAACCACAGA

2 micron origin

340 360 380 400 420

ATTTTCTCTTCCATAAAAAAAGCCTGACTCCACTTCCCGCGTTTACTGATTACTAGCGAAGCTGCGGGTGCATTTTTTCAAGATAAAGGCATCCCCGATTATATTCT
TAAAAGAGAAGGTATTTTTTTCGGACTGAGGTGAAGGGCGCAAATGACTAATGATCGCTTCGACGCCCACGTAAAAAAGTTCTATTTCCGTAGGGGCTAATATAAGA

2 micron origin

440 460 480 500 520

ATACCGATGTGGATTGCGCATACTTTGTGAACAGAAAGTGATAGCGTTGATGATTCTTCATTGGTCAGAAAATTATGAACGGTTTCTTCTATTTTGTCTCTATATAC
TATGGCTACACCTAACGCGTATGAAACACTTGTCTTTCACTATCGCAACTACTAAGAAGTAACCAGTCTTTTAATACTTGCCAAAGAAGATAAAACAGAGATATATG

2 micron origin

540 560 580 600 620 640

TACGTATAGGAAATGTTTACATTTTCGTATTGTTTTCGATTCACTCTATGAATAGTTCTTACTACAATTTTTTTGTCTAAAGAGTAATACTAGAGATAAACATAAAA
ATGCATATCCTTTACAAATGTAAAAGCATAACAAAAGCTAAGTGAGATACTTATCAAGAATGATGTTAAAAAAACAGATTTCTCATTATGATCTCTATTTGTATTTT

2 micron origin

660 680 700 720 740

AATGTAGAGGTCGAGTTTAGATGCAAGTTCAAGGAGCGAAAGGTGGATGGGTAGGTTATATAGGGATATAGCACAGAGATATATAGCAAAGAGATACTTTTGAGCAA
TTACATCTCCAGCTCAAATCTACGTTCAAGTTCCTCGCTTTCCACCTACCCATCCAATATATCCCTATATCGTGTCTCTATATATCGTTTCTCTATGAAAACTCGTT

2 micron origin

760 780 800 820 840

TGTTTGTGGAAGCGGTATTCGCAATATTTTAGTAGCTCGTTACAGTCCGGTGCGTTTTTGGTTTTTTGAAAGTGCGTCTTCAGAGCGCTTTTGGTTTTCAAAAGCGC
ACAAACACCTTCGCCATAAGCGTTATAAAATCATCGAGCAATGTCAGGCCACGCAAAAACCAAAAAACTTTCACGCAGAAGTCTCGCGAAAACCAAAAGTTTTCGCG

2 micron origin

860 880 900 920 940 960

TCTGAAGTTCCTATACTTTCTAGCTAGAGAATAGGAACTTCGGAATAGGAACTTCAAAGCGTTTCCGAAAACGAGCGCTTCCGAAAATGCAACGCGAGCTGCGCACA
AGACTTCAAGGATATGAAAGATCGATCTCTTATCCTTGAAGCCTTATCCTTGAAGTTTCGCAAAGGCTTTTGCTCGCGAAGGCTTTTACGTTGCGCTCGACGCGTGT

2 micron origin

980 1,000 1,020 1,040 1,060

TACAGCTCACTGTTCACGTCGCACCTATATCTGCGTGTTGCCTGTATATATATATACATGAGAAGAACGGCATAGTGCGTGTTTATGCTTAAATGCGTACTTATATG
ATGTCGAGTGACAAGTGCAGCGTGGATATAGACGCACAACGGACATATATATATATGTACTCTTCTTGCCGTATCACGCACAAATACGAATTTACGCATGAATATAC

2 micron origin

1,080 1,100 1,120 1,140 1,160

CGTCTATTTATGTAGGATGAAAGGTAGTCTAGTACCTCCTGTGATATTATCCCATTCCATGCGGGGTATCGTATGCTTCCTTCAGCACTACCCTTTAGCTGTTCTAT
GCAGATAAATACATCCTACTTTCCATCAGATCATGGAGGACACTATAATAGGGTAAGGTACGCCCCATAGCATACGAAGGAAGTCGTGATGGGAAATCGACAAGATA

2 micron origin

1,180 1,200 1,220 1,240 1,260 1,280

ATGCTGCCACTCCTCAATTGGATTAGTCTCATCCTTCAATGCTATCATTTCCTTTGATATTGGATCGATCCGATGATAAGCTGTCAAACATGAGAATTGGGTAATAA
TACGACGGTGAGGAGTTAACCTAATCAGAGTAGGAAGTTACGATAGTAAAGGAAACTATAACCTAGCTAGGCTACTATTCGACAGTTTGTACTCTTAACCCATTATT

2 micron origin URA3

1,300 1,320 1,340 1,360 1,380

CTGATATAATTAAATTGAAGCTCTAATTTGTGAGTTTAGTATACATGCATTTACTTATAATACAGTTTTTTAGTTTTGCTGGCCGCATCTTCTCAAATATGCTTCCC
GACTATATTAATTTAACTTCGAGATTAAACACTCAAATCATATGTACGTAAATGAATATTATGTCAAAAAATCAAAACGACCGGCGTAGAAGAGTTTATACGAAGGG

URA3

1,400 1,420 1,440 1,460 1,480

AGCCTGCTTTTCTGTAACGTTCACCCTCTACCTTAGCATCCCTTCCCTTTGCAAATAGTCCTCTTCCAACAATAATAATGTCAGATCCTGTAGAGACCACATCATCC
TCGGACGAAAAGACATTGCAAGTGGGAGATGGAATCGTAGGGAAGGGAAACGTTTATCAGGAGAAGGTTGTTATTATTACAGTCTAGGACATCTCTGGTGTAGTAGG

URA3

1,500 1,520 1,540 1,560 1,580 1,600

ACGGTTCTATACTGTTGACCCAATGCGTCTCCCTTGTCATCTAAACCCACACCGGGTGTCATAATCAACCAATCGTAACCTTCATCTCTTCCACCCATGTCTCTTTG
TGCCAAGATATGACAACTGGGTTACGCAGAGGGAACAGTAGATTTGGGTGTGGCCCACAGTATTAGTTGGTTAGCATTGGAAGTAGAGAAGGTGGGTACAGAGAAAC

URA3

1,620 1,640 1,660 1,680 1,700

AGCAATAAAGCCGATAACAAAATCTTTGTCGCTCTTCGCAATGTCAACAGTACCCTTAGTATATTCTCCAGTAGATAGGGAGCCCTTGCATGACAATTCTGCTAACA
TCGTTATTTCGGCTATTGTTTTAGAAACAGCGAGAAGCGTTACAGTTGTCATGGGAATCATATAAGAGGTCATCTATCCCTCGGGAACGTACTGTTAAGACGATTGT
URA3
1,720 1,740 1,760 1,780 1,800

TCAAAAGGCCTCTAGGTTCCTTTGTTACTTCTTCTGCCGCCTGCTTCAAACCGCTAACAATACCTGGGCCCACCACACCGTGTGCATTCGTAATGTCTGCCCATTCT
AGTTTTCCGGAGATCCAAGGAAACAATGAAGAAGACGGCGGACGAAGTTTGGCGATTGTTATGGACCCGGGTGGTGTGGCACACGTAAGCATTACAGACGGGTAAGA
URA3
1,820 1,840 1,860 1,880 1,900 1,920

GCTATTCTGTATACACCCGCAGAGTACTGCAATTTGACTGTATTACCAATGTCAGCAAATTTTCTGTCTTCGAAGAGTAAAAAATTGTACTTGGCGGATAATGCCTT
CGATAAGACATATGTGGGCGTCTCATGACGTTAAACTGACATAATGGTTACAGTCGTTTAAAAGACAGAAGCTTCTCATTTTTTAACATGAACCGCCTATTACGGAA
URA3
1,940 1,960 1,980 2,000 2,020

TAGCGGCTTAACTGTGCCCTCCATGGAAAAATCAGTCAAGATATCCACATGTGTTTTTAGTAAACAAATTTTGGGACCTAATGCTTCAACTAACTCCAGTAATTCCT
ATCGCCGAATTGACACGGGAGGTACCTTTTTAGTCAGTTCTATAGGTGTACACAAAAATCATTTGTTTAAAACCCTGGATTACGAAGTTGATTGAGGTCATTAAGGA
URA3
2,040 2,060 2,080 2,100 2,120 2,140

TGGTGGTACGAACATCCAATGAAGCACACAAGTTTGTTTGCTTTTCGTGCATGATATTAAATAGCTTGGCAGCAACAGGACTAGGATGAGTAGCAGCACGTTCCTTA
ACCACCATGCTTGTAGGTTACTTCGTGTGTTCAAACAAACGAAAAGCACGTACTATAATTTATCGAACCGTCGTTGTCCTGATCCTACTCATCGTCGTGCAAGGAAT
URA3
2,160 2,180 2,200 2,220 2,240

TATGTAGCTTTCGACATGATTTATCTTCGTTTCCTGCATGTTTTTGTTCTGTGCAGTTGGGTTAAGAATACTGGGCAATTTCATGTTTCTTCAACACTACATATGCG
ATACATCGAAAGCTGTACTAAATAGAAGCAAAGGACGTACAAAAACAAGACACGTCAACCCAATTCTTATGACCCGTTAAAGTACAAAGAAGTTGTGATGTATACGC
URA3
2,260 2,280 2,300 2,320 2,340

TATATATACCAATCTAAGTCTGTGCTCCTTCCTTCGTTCTTCCTTCTGTTCGGAGATTACCGAATCAAAAAAATTTCAAAGAAACCGAAATCAAAAAAAAGAATAAA
ATATATATGGTTAGATTCAGACACGAGGAAGGAAGCAAGAAGGAAGACAAGCCTCTAATGGCTTAGTTTTTTTAAAGTTTCTTTGGCTTTAGTTTTTTTTCTTATTT
URA3
2,360 2,380 2,400 2,420 2,440 2,460

AAAAAAATGATGAATTGAATTGAAAAGCTAATTCTTGAAGACGAAAGGGCCTCGTGATACGCCTATTTTTATAGGTTAATGTCATGATAATAATGGTTTCTTAGACG
TTTTTTTACTACTTAACTTAACTTTTCGATTAAGAACTTCTGCTTTCCCGGAGCACTATGCGGATAAAAATATCCAATTACAGTACTATTATTACCAAAGAATCTGC
URA3
2,480 2,500 2,520 2,540 2,560

TCAGGTGGCACTTTTCGGGGAAATGTGCGCGGAACCCCTATTTGTTTATTTTTCTAAATACATTCAAATATGTATCCGCTCATGAGACAATAACCCTGATAAATGCT
AGTCCACCGTGAAAAGCCCCTTTACACGCGCCTTGGGGATAAACAAATAAAAAGATTTATGTAAGTTTATACATAGGCGAGTACTCTGTTATTGGGACTATTTACGA
2,580 2,600 2,620 2,640 2,660

TCAATAATATTGAAAAAGGAAGAGTATGAGTATTCAACATTTCCGTGTCGCCCTTATTCCCTTTTTTGCGGCATTTTGCCTTCCTGTTTTTGCTCACCCAGAAACGC
AGTTATTATAACTTTTTCCTTCTCATACTCATAAGTTGTAAAGGCACAGCGGGAATAAGGGAAAAAACGCCGTAAAACGGAAGGACAAAAACGAGTGGGTCTTTGCG

2,680 2,700 2,720 2,740 2,760 2,780

TGGTGAAAGTAAAAGATGCTGAAGATCAGTTGGGTGCACGAGTGGGTTACATCGAACTGGATCTCAACAGCGGTAAGATCCTTGAGAGTTTTCGCCCCGAAGAACGT
ACCACTTTCATTTTCTACGACTTCTAGTCAACCCACGTGCTCACCCAATGTAGCTTGACCTAGAGTTGTCGCCATTCTAGGAACTCTCAAAAGCGGGGCTTCTTGCA

2,800 2,820 2,840 2,860 2,880

TTTCCAATGATGAGCACTTTTAAAGTTCTGCTATGTGGCGCGGTATTATCCCGTATTGACGCCGGGCAAGAGCAACTCGGTCGCCGCATACACTATTCTCAGAATGA
AAAGGTTACTACTCGTGAAAATTTCAAGACGATACACCGCGCCATAATAGGGCATAACTGCGGCCCGTTCTCGTTGAGCCAGCGGCGTATGTGATAAGAGTCTTACT

AmpR

2,900 2,920 2,940 2,960 2,980

CTTGGTTGAGTACTCACCAGTCACAGAAAAGCATCTTACGGATGGCATGACAGTAAGAGAATTATGCAGTGCTGCCATAACCATGAGTGATAACACTGCGGCCAACT
GAACCAACTCATGAGTGGTCAGTGTCTTTTCGTAGAATGCCTACCGTACTGTCATTCTCTTAATACGTCACGACGGTATTGGTACTCACTATTGTGACGCCGGTTGA

AmpR

3,000 3,020 3,040 3,060 3,080 3,100

TACTTCTGACAACGATCGGAGGACCGAAGGAGCTAACCGCTTTTTTGCACAACATGGGGGATCATGTAACTCGCCTTGATCGTTGGGAACCGGAGCTGAATGAAGCC
ATGAAGACTGTTGCTAGCCTCCTGGCTTCCTCGATTGGCGAAAAAACGTGTTGTACCCCCTAGTACATTGAGCGGAACTAGCAACCCTTGGCCTCGACTTACTTCGG

AmpR

3,120 3,140 3,160 3,180 3,200

ATACCAAACGACGAGCGTGACACCACGATGCCTGTAGCAATGGCAACAACGTTGCGCAAACTATTAACTGGCGAACTACTTACTCTAGCTTCCCGGCAACAATTAAT
TATGGTTTGCTGCTCGCACTGTGGTGCTACGGACATCGTTACCGTTGTTGCAACGCGTTTGATAATTGACCGCTTGATGAATGAGATCGAAGGGCCGTTGTTAATTA

AmpR

3,220 3,240 3,260 3,280 3,300

AGACTGGATGGAGGCGGATAAAGTTGCAGGACCACTTCTGCGCTCGGCCCTTCCGGCTGGCTGGTTTATTGCTGATAAATCTGGAGCCGGTGAGCGTGGGTCTCGCG
TCTGACCTACCTCCGCCTATTTCAACGTCCTGGTGAAGACGCGAGCCGGGAAGGCCGACCGACCAAATAACGACTATTTAGACCTCGGCCACTCGCACCCAGAGCGC

AmpR

3,320 3,340 3,360 3,380 3,400 3,420

GTATCATTGCAGCACTGGGGCCAGATGGTAAGCCCTCCCGTATCGTAGTTATCTACACGACGGGGAGTCAGGCAACTATGGATGAACGAAATAGACAGATCGCTGAG
CATAGTAACGTCGTGACCCCGGTCTACCATTCGGGAGGGCATAGCATCAATAGATGTGCTGCCCCTCAGTCCGTTGATACCTACTTGCTTTATCTGTCTAGCGACTC

AmpR

3,440 3,460 3,480 3,500 3,520

ATAGGTGCCTCACTGATTAAGCATTGGTAACTGTCAGACCAAGTTTACTCATATATACTTTAGATTGATTTAAAACTTCATTTTTAATTTAAAAGGATCTAGGTGAA
TATCCACGGAGTGACTAATTCGTAACCATTGACAGTCTGGTTCAAATGAGTATATATGAAATCTAACTAAATTTTGAAGTAAAAATTAAATTTTCCTAGATCCACTT

AmpR

3,540 3,560 3,580 3,600 3,620

GATCCTTTTTGATAATCTCATGACCAAAATCCCTTAACGTGAGTTTTCGTTCCACTGAGCGTCAGACCCCGTAGAAAAGATCAAAGGATCTTCTTGAGATCCTTTTT
CTAGGAAAAACTATTAGAGTACTGGTTTTAGGGAATTGCACTCAAAAGCAAGGTGACTCGCAGTCTGGGGCATCTTTTCTAGTTTCCTAGAAGAACTCTAGGAAAAA

ColE1 origin

3,640 3,660 3,680 3,700 3,720 3,740

TTCTGCGCGTAATCTGCTGCTTGCAAACAAAAAAACCACCGCTACCAGCGGTGGTTTGTTTGCCGGATCAAGAGCTACCAACTCTTTTTCCGAAGGTAACTGGCTTC
AAGACGCGCATTAGACGACGAACGTTTGTTTTTTTGGTGGCGATGGTCGCCACCAAACAAACGGCCTAGTTCTCGATGGTTGAGAAAAAGGCTTCCATTGACCGAAG

ColE1 origin

3,760 3,780 3,800 3,820 3,840

AGCAGAGCGCAGATACCAAATACTGTCCTTCTAGTGTAGCCGTAGTTAGGCCACCACTTCAAGAACTCTGTAGCACCGCCTACATACCTCGCTCTGCTAATCCTGTT
TCGTCTCGCGTCTATGGTTTATGACAGGAAGATCACATCGGCATCAATCCGGTGGTGAAGTTCTTGAGACATCGTGGCGGATGTATGGAGCGAGACGATTAGGACAA

ColE1 origin

3,860 3,880 3,900 3,920 3,940

ACCAGTGGCTGCTGCCAGTGGCGATAAGTCGTGTCTTACCGGGTTGGACTCAAGACGATAGTTACCGGATAAGGCGCAGCGGTCGGGCTGAACGGGGGGTTCGTGCA
TGGTCACCGACGACGGTCACCGCTATTCAGCACAGAATGGCCCAACCTGAGTTCTGCTATCAATGGCCTATTCCGCGTCGCCAGCCCGACTTGCCCCCCAAGCACGT

ColE1 origin

3,960 3,980 4,000 4,020 4,040 4,060

CACAGCCCAGCTTGGAGCGAACGACCTACACCGAACTGAGATACCTACAGCGTGAGCTATGAGAAAGCGCCACGCTTCCCGAAGGGAGAAAGGCGGACAGGTATCCG
GTGTCGGGTCGAACCTCGCTTGCTGGATGTGGCTTGACTCTATGGATGTCGCACTCGATACTCTTTCGCGGTGCGAAGGGCTTCCCTCTTTCCGCCTGTCCATAGGC

ColE1 origin

4,080 4,100 4,120 4,140 4,160

GTAAGCGGCAGGGTCGGAACAGGAGAGCGCACGAGGGAGCTTCCAGGGGGAAACGCCTGGTATCTTTATAGTCCTGTCGGGTTTCGCCACCTCTGACTTGAGCGTCG
CATTCGCCGTCCCAGCCTTGTCCTCTCGCGTGCTCCCTCGAAGGTCCCCCTTTGCGGACCATAGAAATATCAGGACAGCCCAAAGCGGTGGAGACTGAACTCGCAGC

ColE1 origin

4,180 4,200 4,220 4,240 4,260 4,280

ATTTTTGTGATGCTCGTCAGGGGGGCGGAGCCTATGGAAAAACGCCAGCAACGCGGCCTTTTTACGGTTCCTGGCCTTTTGCTGGCCTTTTGCTCACATGTTCTTTC
TAAAAACACTACGAGCAGTCCCCCCGCCTCGGATACCTTTTTGCGGTCGTTGCGCCGGAAAAATGCCAAGGACCGGAAAACGACCGGAAAACGAGTGTACAAGAAAG

ColE1 origin

4,300 4,320 4,340 4,360 4,380

CTGCGTTATCCCCTGATTCTGTGGATAACCGTATTACCGCCTTTGAGTGAGCTGATACCGCTCGCCGCAGCCGAACGACCGAGCGCAGCGAGTCAGTGAGCGAGGAA
GACGCAATAGGGGACTAAGACACCTATTGGCATAATGGCGGAAACTCACTCGACTATGGCGAGCGGCGTCGGCTTGCTGGCTCGCGTCGCTCAGTCACTCGCTCCTT

4,400 4,420 4,440 4,460 4,480

GCGGAAGAGCGCCCAATACGCAAACCGCCTCTCCCCGCGCGTTGGCCGATTCATTAATGCAGCTGGCACGACAGGTTTCCCGACTGGAAAGCGGGCAGTGAGCGCAA
CGCCTTCTCGCGGGTTATGCGTTTGGCGGAGAGGGGCGCGCAACCGGCTAAGTAATTACGTCGACCGTGCTGTCCAAAGGGCTGACCTTTCGCCCGTCACTCGCGTT

4,500 4,520 4,540 4,560 4,580 4,600

CGCAATTAATGTGAGTTAGCTCACTCATTAGGCACCCCAGGCTTTACACTTTATGCTTCCGGCTCGTATGTTGTGTGGAATTGTGAGCGGATAACAATTTCACACAG
GCGTTAATTACACTCAATCGAGTGAGTAATCCGTGGGGTCCGAAATGTGAAATACGAAGGCCGAGCATACAACACACCTTAACACTCGCCTATTGTTAAAGTGTGTC

lac promoter LacO

4,620 4,640 4,660 4,680 4,700

GAAACAGCTATGACCATGATTACGCCAAGCTTCGGATTAGAAGCCGCCGAGCGGGTGACAGCCCTCCGAAGGAAGACTCTCCTCCGTGCGTCCTCGTCTTCACCGGT
CTTTGTCGATACTGGTACTAATGCGGTTCGAAGCCTAATCTTCGGCGGCTCGCCCACTGTCGGGAGGCTTCCTTCTGAGAGGAGGCACGCAGGAGCAGAAGTGGCCA

M13-rev GAL1 promoter

4,720 4,740 4,760 4,780 4,800

CGCGTTCCTGAAACGCAGATGTGCCTCGCGCCGCACTGCTCCGAACAATAAAGATTCTACAATACTAGCTTTTATGGTTATGAAGAGGAAAAATTGGCAGTAACCTG
GCGCAAGGACTTTGCGTCTACACGGAGCGCGGCGTGACGAGGCTTGTTATTTCTAAGATGTTATGATCGAAAATACCAATACTTCTCCTTTTTAACCGTCATTGGAC

GAL1 promoter

4,820 4,840 4,860 4,880 4,900 4,920

GCCCCACAAACCTTCAAATGAACGAATCAAATTAACAACCATAGGATGATAATGCGATTAGTTTTTTAGCCTTATTTCTGGGGTAATTAATCAGCGAAGCGATGATT
CGGGGTGTTTGGAAGTTTACTTGCTTAGTTTAATTGTTGGTATCCTACTATTACGCTAATCAAAAAATCGGAATAAAGACCCCATTAATTAGTCGCTTCGCTACTAA

GAL1 promoter

4,940 4,960 4,980 5,000 5,020

TTTGATCTATTAACAGATATATAAATGCAAAAACTGCATAACCACTTTAACTAATACTTTCAACATTTTCGGTTTGTATTACTTCTTATTCAAATGTAATAAAAGTA
AAACTAGATAATTGTCTATATATTTACGTTTTTGACGTATTGGTGAAATTGATTATGAAAGTTGTAAAAGCCAAACATAATGAAGAATAAGTTTACATTATTTTCAT

GAL1 promoter

5,040 5,060 5,080 5,100 5,120

TCAACAAAAAATTGTTAATATACCTCTATACTTTAACGTCAAGGAGAAAAAACCCCGGATCGAATTCAACCCTCACTAAAGGGCGGCCGCCATGAGATTCCCATCTA
AGTTGTTTTTTAACAATTATATGGAGATATGAAATTGCAGTTCCTCTTTTTTGGGGCCTAGCTTAAGTTGGGAGTGATTTCCCGCCGGCGGTACTCTAAGGGTAGAT

1 2 3 4 5
M R F P S

GAL1 promoter

5,140 5,160 5,180 5,200 5,220 5,240

TCTTCACCGCTGTTTTGTTCGCTGCTTCTTCTGCTTTGGCTGCTCCAGCTAACACCACCACCGAAGACGAAACCGCTCAAATCCCAGCTGAAGCTGTTATCGACTAC
AGAAGTGGCGACAAAACAAGCGACGAAGAAGACGAAACCGACGAGGTCGATTGTGGTGGTGGCTTCTGCTTTGGCGAGTTTAGGGTCGACTTCGACAATAGCTGATG

6 7 8 9 10 11 12 13 14 15 16 17 18 19 20 21 22 23 24 25 26 27 28 29 30 31 32 33 34 35 36 37 38 39 40 41
I F T A V L F A A S S A L A A P A N T T T E D E T A Q I P A E A V I D Y

Mating factor alpha leader

5,260 5,280 5,300 5,320 5,340

```
TCTGACTTGGAAGGTGACTTCGACGCTGCTGCTTTGCCATTGTCTAACTCTACCAACAACGGTTTGTCTTCTACCAACACCACCATCGCTTCTATCGCTGCTAAGGA
AGACTGAACCTTCCACTGAAGCTGCGACGACGAAACGGTAACAGATTGAGATGGTTGTTGCCAAACAGAAGATGGTTGTGGTGGTAGCGAAGATAGCGACGATTCCT
 42 43 44 45 46 47 48 49 50 51 52 53 54 55 56 57 58 59 60 61 62 63 64 65 66 67 68 69 70 71 72 73 74 75 76 77
  S  D  L  E  G  D  F  D  A  A  A  L  P  L  S  N  S  T  N  N  G  L  S  S  T  N  T  T  I  A  S  I  A  A  K  E
Mating factor alpha leader
5,360  5,380  5,400  5,420  5,440
```

```
AGAAGGTGTTCAATTGGACAAGAGAGAAagatctggcggccgcgcatgcgctagcctcgagGGTGGATCTGATTACAAGGATGACGATGACAAGGCTGGAGGTTCGA
TCTTCCACAAGTTAACCTGTTCTCTCTTtctagaccgccggcgcgtacgcgatcggagctcCCACCTAGACTAATGTTCCTACTGCTACTGTTCCGACCTCCAAGCT
  78 79 80 81 82 83 84 85 86
   E  G  V  Q  L  D  K  R  E
FLAG
5,460  5,480  5,500  5,520  5,540  5,560
```

```
GCGCCAAAAGCTCTTTTATCTCAACCACTACTACTGATTTAACAAGTATAAACACTAGTGCGTATTCCACTGGATCCATTTCCACAGTAGAAACAGGCAATCGAACT
CGCGGTTTTCGAGAAAATAGAGTTGGTGATGATGACTAAATTGTTCATATTTGTGATCACGCATAAGGTGACCTAGGTAAAGGTGTCATCTTTGTCCGTTAGCTTGA
   2   4   6   8   10   12   14   16   18   20   22   24   26   28   30   32   34   36
S A K S S F I S T T T T D L T S I N T S A Y S T G S I S T V E T G N R T
3' Half of α-agglutinin
5,580  5,600  5,620  5,640  5,660
```

```
ACATCAGAAGTGATCAGTCATGTGGTGACTACCAGCACAAAACTGTCTCCAACTGCTACTACCAGCCTGACAATTGCACAAACCAGTATCTATTCTACTGACTCATA
TGTAGTCTTCACTAGTCAGTACACCACTGATGGTCGTGTTTTGACAGAGGTTGACGATGATGGTCGGACTGTTAACGTGTTTGGTCATAGATAAGATGACTGAGTAT
   38   40   42   44   46   48   50   52   54   56   58   60   62   64   66   68   70   72
T S E V I S H V V T T S T K L S P T A T T S L T I A Q T S I Y S T D S Y
3' Half of α-agglutinin
5,680  5,700  5,720  5,740  5,760
```

```
TATCACAGTAGGAACAGATATTCACACCACATCAGAAGTGATTAGTGATGTGGAAACCATTAGCAGAGAAACAGCTTCGACCGTTGTAGCCGCTCCAACCTCAACAA
ATAGTGTCATCCTTGTCTATAAGTGTGGTGTAGTCTTCACTAATCACTACACCTTTGGTAATCGTCTCTTTGTCGAAGCTGGCAACATCGGCGAGGTTGGAGTTGTT
   74   76   78   80   82   84   86   88   90   92   94   96   98   100   102   104   106
I T V G T D I H T T S E V I S D V E T I S R E T A S T V V A A P T S T
3' Half of α-agglutinin
5,780  5,800  5,820  5,840  5,860  5,880
```

```
CTGGATGGACAGGCGCTATGAATACTTACATCTCGCAATTTACATCCTCTTCTTTCGCAACAATCAACAGCACACCAATAATCTCTTCATCAGCAGTATTTGAAACC
GACCTACCTGTCCGCGATACTTATGAATGTAGAGCGTTAAATGTAGGAGAAGAAAGCGTTGTTAGTTGTCGTGTGGTTATTAGAGAAGTAGTCGTCATAAACTTTGG
108   110   112   114   116   118   120   122   124   126   128   130   132   134   136   138   140   142
T G W T G A M N T Y I S Q F T S S S F A T I N S T P I I S S S A V F E T
3' Half of α-agglutinin
5,900  5,920  5,940  5,960  5,980
```

```
TCAGATGCTTCAATTGTCAATGTGCACACTGAAAATATCACGAATACTGCTGCTGTTCCATCTGAAGAGCCCACTTTTGTAAATGCCACGAGAAACTCCTTAAATTC
AGTCTACGAAGTTAACAGTTACACGTGTGACTTTTATAGTGCTTATGACGACGACAAGGTAGACTTCTCGGGTGAAAACATTTACGGTGCTCTTTGAGGAATTTAAG
144   146   148   150   152   154   156   158   160   162   164   166   168   170   172   174   176   178
S  D  A  S  I  V  N  V  H  T  E  N  I  T  N  T  A  A  V  P  S  E  E  P  T  F  V  N  A  T  R  N  S  L  N  S
3' Half of α-agglutinin
6,000   6,020   6,040   6,060   6,080

CTTCTGCAGCAGCAAACAGCCATCCAGTCCCTCATCTTATACGTCTTCCCCACTCGTATCGTCCCTCTCCGTAAGCAAAACATTACTAAGCACCAGTTTTACGCCTT
GAAGACGTCGTCGTTTGTCGGTAGGTCAGGGAGTAGAATATGCAGAAGGGGTGAGCATAGCAGGGAGAGGCATTCGTTTTGTAATGATTCGTGGTCAAAATGCGGAA
180   182   184   186   188   190   192   194   196   198   200   202   204   206   208   210   212   214
F  C  S  S  K  Q  P  S  S  P  S  S  Y  T  S  S  P  L  V  S  S  L  S  V  S  K  T  L  L  S  T  S  F  T  P
3' Half of α-agglutinin
6,100   6,120   6,140   6,160   6,180   6,200

CTGTGCCAACATCTAATACATATATCAAAACGAAAAATACGGGTTACTTTGAGCACACGGCTTTGACAACATCTTCAGTTGGCCTTAATTCTTTTAGTGAAACAGCA
GACACGGTTGTAGATTATGTATATAGTTTTGCTTTTTATGCCCAATGAAACTCGTGTGCCGAAACTGTTGTAGAAGTCAACCGGAATTAAGAAAATCACTTTGTCGT
216   218   220   222   224   226   228   230   232   234   236   238   240   242   244   246   248   250
S  V  P  T  S  N  T  Y  I  K  T  K  N  T  G  Y  F  E  H  T  A  L  T  T  S  S  V  G  L  N  S  F  S  E  T  A
3' Half of α-agglutinin
6,220   6,240   6,260   6,280   6,300

GTCTCATCTCAGGGAACGAAAATTGACACCTTTTTAGTGTCATCCTTGATCGCATATCCTTCTTCTGCATCAGGAAGCCAATTGTCCGGTATCCAACAGAATTTCAC
CAGAGTAGAGTCCCTTGCTTTTAACTGTGGAAAAATCACAGTAGGAACTAGCGTATAGGAAGAAGACGTAGTCCTTCGGTTAACAGGCCATAGGTTGTCTTAAAGTG
252   254   256   258   260   262   264   266   268   270   272   274   276   278   280   282   284   286
V  S  S  Q  G  T  K  I  D  T  F  L  V  S  S  L  I  A  Y  P  S  S  A  S  G  S  Q  L  S  G  I  Q  Q  N  F  T
3' Half of α-agglutinin
6,320   6,340   6,360   6,380   6,400   6,420

ATCAACTTCTCTCATGATTTCAACCTATGAAGGTAAAGCGTCTATATTTTTCTCAGCTGAGCTCGGTTCGATCATTTTTCTGCTTTTGTCGTACCTGCTATTCTAAA
TAGTTGAAGAGAGTACTAAAGTTGGATACTTCCATTTCGCAGATATAAAAAGAGTCGACTCGAGCCAAGCTAGTAAAAAGACGAAAACAGCATGGACGATAAGATTT
288   290   292   294   296   298   300   302   304   306   308   310   312   314   316   318   320
S  T  S  L  M  I  S  T  Y  E  G  K  A  S  I  F  F  S  A  E  L  G  S  I  I  F  L  L  L  S  Y  L  L  F  *
3' Half of α-agglutinin
6,440   6,460   6,480   6,500   6,520

ACGGGTACTGTACAGTTAGTACATTGAGTCGAAATATACGAAATTATTGTTCATAATTTTCATCCTGGCTCTTTTTTTCTTCAACCATAGTTAAATGGACAGTTCAT
TGCCCATGACATGTCAATCATGTAACTCAGCTTTATATGCTTTAATAACAAGTATTAAAAGTAGGACCGAGAAAAAAAGAAGTTGGTATCAATTTACCTGTCAAGTA
3' Half of α-agglutinin
6,540   6,560   6,580   6,600   6,620

ATCTTAACTCTAATAATACTTTTCTAGTTCTTATCCTTTTCCGTCTCACCGCAGATTTTATCATAGTATTAAATTTATATTTTGTTCGTAAAAAGAAAAATTTGTGA
TAGAATTGAGATTATTATGAAAAGATCAAGAATAGGAAAAGGCAGAGTGGCGTCTAAAATAGTATCATAATTTAAATATAAAACAAGCATTTTTCTTTTTAAACACT
3' Half of α-agglutinin
6,640   6,660   6,680   6,700   6,720   6,740
```

GCGTTACCGCTCGTTTCATTACCCGAAGGCTGTTTCAGTAGACCACTGATTAAGTAAGTAGATGAAAAAATTTCATCACCATGAAAGAGTTCGATGAGAGCTACTTT
CGCAATGGCGAGCAAAGTAATGGGCTTCCGACAAAGTCATCTGGTGACTAATTCATTCATCTACTTTTTTAAAGTAGTGGTACTTTCTCAAGCTACTCTCGATGAAA

3′ Half of α-agglutinin

6,760 6,780 6,800 6,820 6,840

TTCAAATGCTTAACAGCTAACCGCCATTCAATAATGTTACGCTCTCTTCATTCTGCGGCTACGTTATCTAACAAGAGGTTTTACTCTCTCATATCTCATTCAAATAG
AAGTTTACGAATTGTCGATTGGCGGTAAGTTATTACAATGCGAGAGAAGTAAGACGCCGATGCAATAGATTGTTCTCCAAAATGAGAGAGTATAGAGTAAGTTTATC

3′ Half of α-agglutinin

6,860 6,880 6,900 6,920 6,940

AAAGAACATAATCAAAGGTACCGCGATGTAGTAAAACTAGCTAGACCGAGAAAGAGACTAGAAATGCAAAAGGCACTTCTACAATGGCTGCCATCATTATTATCCGA
TTTCTTGTATTAGTTTCCATGGCGCTACATCATTTTGATCGATCTGGCTCTTTCTCTGATCTTTACGTTTTCCGTGAAGATGTTACCGACGGTAGTAATAATAGGCT

6,960 6,980 7,000 7,020 7,040 7,060

TGTGACGCTGCATTTTTTTTTTTTTTTTTTTTTTTTTTTTTTTTTTTTTTTTTTTTTTGTACAAATATCATAAAAAAAGAGAATCTTTTTAAGCAAGGATTTTCTT
ACACTGCGACGTAAAAAAAAAAAAAAAAAAAAAAAAAAAAAAAAAAAAAAAAAAAAAAACATGTTTATAGTATTTTTTTCTCTTAGAAAAATTCGTTCCTAAAAGAA

7,080 7,100 7,120 7,140 7,160

AACTTCTTCGGCGACAGCATCACCGACTTCGGTGGTACTGTTGGAACCACCTAAATCACCAGTTCTGATACCTGCATCCAAAACCTTTTTAACTGCATCTTCAATGG
TTGAAGAAGCCGCTGTCGTAGTGGCTGAAGCCACCATGACAACCTTGGTGGATTTAGTGGTCAAGACTATGGACGTAGGTTTTGGAAAAATTGACGTAGAAGTTACC

7,180 7,200 7,220 7,240 7,260

CCTTACCTTCTTCAGGCAAGTTCAATGACAATTTCAACATCATTGCAGCAGACAAGATAGTGGCGATAGGGTCAACCTTATTCTTTGGCAAATCTGGAGCAGAACCG
GGAATGGAAGAAGTCCGTTCAAGTTACTGTTAAAGTTGTAGTAACGTCGTCTGTTCTATCACCGCTATCCCAGTTGGAATAAGAAACCGTTTAGACCTCGTCTTGGC

7,280 7,300 7,320 7,340 7,360 7,380

TGGCATGGTTCGTACAAACCAAATGCGGTGTTCTTGTCTGGCAAAGAGGCCAAGGACGCAGATGGCAACAAACCCAAGGAACCTGGGATAACGGAGGCTTCATCGGA
ACCGTACCAAGCATGTTTGGTTTACGCCACAAGAACAGACCGTTTCTCCGGTTCCTGCGTCTACCGTTGTTTGGGTTCCTTGGACCCTATTGCCTCCGAAGTAGCCT

7,400 7,420 7,440 7,460 7,480

GATGATATCACCAAACATGTTGCTGGTGATTATAATACCATTTAGGTGGGTTGGGTTCTTAACTAGGATCATGGCGGCAGAATCAATCAATTGATGTTGAACCTTCA
CTACTATAGTGGTTTGTACAACGACCACTAATATTATGGTAAATCCACCCAACCCAAGAATTGATCCTAGTACCGCCGTCTTAGTTAGTTAACTACAACTTGGAAGT

7,500 7,520 7,540 7,560 7,580

ATGTAGGAAATTCGTTCTTGATGGTTTCCTCCACAGTTTTTCTCCATAATCTTGAAGAGGCCAAAACATTAGCTTTATCCAAGGACCAAATAGGCAATGGTGGCTCA
TACATCCTTTAAGCAAGAACTACCAAAGGAGGTGTCAAAAAGAGGTATTAGAACTTCTCCGGTTTTGTAATCGAAATAGGTTCCTGGTTTATCCGTTACCACCGAGT

7,600 7,620 7,640 7,660 7,680 7,700

TGTTGTAGGGCCATGAAAGCGGCCATTCTTGTGATTCTTTGCACTTCTGGAACGGTGTATTGTTCACTATCCCAAGCGACACCATCACCATCGTCTTCCTTTCTCTT
ACAACATCCCGGTACTTTCGCCGGTAAGAACACTAAGAAACGTGAAGACCTTGCCACATAACAAGTGATAGGGTTCGCTGTGGTAGTGGTAGCAGAAGGAAAGAGAA

7,720 7,740 7,760 7,780 7,800

ACCAAAGTAAATACCTCCCACTAATTCTCTGACAACAACGAAGTCAGTACCTTTAGCAAATTGTGGCTTGATTGGAGATAAGTCTAAAAGAGAGTCGGATGCAAAGT
TGGTTTCATTTATGGAGGGTGATTAAGAGACTGTTGTTGCTTCAGTCATGGAAATCGTTTAACACCGAACTAACCTCTATTCAGATTTTCTCTCAGCCTACGTTTCA

7,820 7,840 7,860 7,880 7,900

TACATGGTCTTAAGTTGGCGTACAATTGAAGTTCTTTACGGATTTTTAGTAAACCTTGTTCAGGTCTAACACTACCTGTACCCCATTTAGGACCACCCACAGCACCT
ATGTACCAGAATTCAACCGCATGTTAACTTCAAGAAATGCCTAAAAATCATTTGGAACAAGTCCAGATTGTGATGGACATGGGGTAAATCCTGGTGGGTGTCGTGGA

7,920 7,940 7,960 7,980 8,000 8,020

AACAAAACGGCATCAACCTTCTTGGAGGCTTCCAGCGCCTCATCTGGAAGTGGGACACCTGTAGCATCGATAGCAGCACCACCAATTAAATGATTTTCGAAATCGAA
TTGTTTTGCCGTAGTTGGAAGAACCTCCGAAGGTCGCGGAGTAGACCTTCACCCTGTGGACATCGTAGCTATCGTCGTGGTGGTTAATTTACTAAAAGCTTTAGCTT

8,040 8,060 8,080 8,100 8,120

CTTGACATTGGAACGAACATCAGAAATAGCTTTAAGAACCTTAATGGCTTCGGCTGTGATTTCTTGACCAACGTGGTCACCTGGCAAAACGACGATCTTCTTAGGGG
GAACTGTAACCTTGCTTGTAGTCTTTATCGAAATTCTTGGAATTACCGAAGCCGACACTAAAGAACTGGTTGCACCAGTGGACCGTTTTGCTGCTAGAAGAATCCCC

8,140 8,160 8,180 8,200 8,220

CAGACATTACAATGGTATATCCTTGAAATATATATAAAAAAAAAAAAAAAAAAAAAAAAAAAAAAATGCAGCTTCTCAATGATATTCGAATACGCTTTGAGGAGATA
GTCTGTAATGTTACCATATAGGAACTTTATATATATTTTTTTTTTTTTTTTTTTTTTTTTTTTTTTTACGTCGAAGAGTTACTATAAGCTTATGCGAAACTCCTCTAT

8,240 8,260 8,280 8,300 8,320 8,340

CAGCCTAATATCCGACAAACTGTTTTACAGATTTACGATCGTACTTGTTACCCATCATTGAATTTTGAACATCCGAACCTGGGAGTTTTCCCTGAAACAGATAGTAT
GTCGGATTATAGGCTGTTTGACAAAATGTCTAAATGCTAGCATGAACAATGGGTAGTAACTTAAAACTTGTAGGCTTGGACCCTCAAAAGGGACTTTGTCTATCATA

8,360 8,380 8,400 8,420 8,440

ATTTGAACCTGTATAATAATATATAGTCTAGCGCTTTACGGAAGACAATGTATGTATTTCGGTTCCTGGAGAAACTATTGCATCTATTGCATAGGTAATCTTGCACG
TAAACTTGGACATATTATTATATATCAGATCGCGAAATGCCTTCTGTTACATACATAAAGCCAAGGACCTCTTTGATAACGTAGATAACGTATCCATTAGAACGTGC

8,460 8,480 8,500 8,520 8,540 8,560

TCGCATCCCCGGTTCATTTTCTGCGTTTCCATCTTGCACTTCAATAGCATATCTTT
AGCGTAGGGGCCAAGTAAAAGACGCAAAGGTAGAACGTGAAGTTATCGTATAGAAA

8,570 8,580 8,590 8,600 8,610

System 5 anti-lys Nb strain (9962 bp)

```
GTTAACGAAGCATCTGTGCTTCATTTTGTAGAACAAAAATGCAACGCGAGAGCGCTAATTTTTCAAACAAAGAATCTGAGCTGCATTTTTACAGAACAGAAATGCAA
CAATTGCTTCGTAGACACGAAGTAAAACATCTTGTTTTTACGTTGCGCTCTCGCGATTAAAAAGTTTGTTTCTTAGACTCGACGTAAAAATGTCTTGTCTTTACGTT
2 micron origin
20 40 60 80 100

CGCGAAAGCGCTATTTTACCAACGAAGAATCTGTGCTTCATTTTTGTAAAACAAAAATGCAACGCGAGAGCGCTAATTTTTCAAACAAAGAATCTGAGCTGCATTTT
GCGCTTTCGCGATAAAATGGTTGCTTCTTAGACACGAAGTAAAAACATTTTGTTTTTACGTTGCGCTCTCGCGATTAAAAAGTTTGTTTCTTAGACTCGACGTAAAA
2 micron origin
120 140 160 180 200

TACAGAACAGAAATGCAACGCGAGAGCGCTATTTTACCAACAAAGAATCTATACTTCTTTTTTGTTCTACAAAAATGCATCCCGAGAGCGCTATTTTTCTAACAAAG
ATGTCTTGTCTTTACGTTGCGCTCTCGCGATAAAATGGTTGTTTCTTAGATATGAAGAAAAAACAAGATGTTTTTACGTAGGGCTCTCGCGATAAAAAGATTGTTTC
2 micron origin
220 240 260 280 300 320

CATCTTAGATTACTTTTTTTCTCCTTTGTGCGCTCTATAATGCAGTCTCTTGATAACTTTTTGCACTGTAGGTCCGTTAAGGTTAGAAGAAGGCTACTTTGGTGTCT
GTAGAATCTAATGAAAAAAAGAGGAAACACGCGAGATATTACGTCAGAGAACTATTGAAAAACGTGACATCCAGGCAATTCCAATCTTCTTCCGATGAAACCACAGA
2 micron origin
340 360 380 400 420

ATTTTCTCTTCCATAAAAAAAGCCTGACTCCACTTCCCGCGTTTACTGATTACTAGCGAAGCTGCGGGTGCATTTTTTCAAGATAAAGGCATCCCCGATTATATTCT
TAAAAGAGAAGGTATTTTTTTCGGACTGAGGTGAAGGGCGCAAATGACTAATGATCGCTTCGACGCCCACGTAAAAAAGTTCTATTTCCGTAGGGGCTAATATAAGA
2 micron origin
440 460 480 500 520

ATACCGATGTGGATTGCGCATACTTTGTGAACAGAAAGTGATAGCGTTGATGATTCTTCATTGGTCAGAAAATTATGAACGGTTTCTTCTATTTTGTCTCTATATAC
TATGGCTACACCTAACGCGTATGAAACACTTGTCTTTCACTATCGCAACTACTAAGAAGTAACCAGTCTTTTAATACTTGCCAAAGAAGATAAAACAGAGATATATG
2 micron origin
540 560 580 600 620 640

TACGTATAGGAAATGTTTACATTTTCGTATTGTTTTCGATTCACTCTATGAATAGTTCTTACTACAATTTTTTTGTCTAAAGAGTAATACTAGAGATAAACATAAAA
ATGCATATCCTTTACAAATGTAAAAGCATAACAAAAGCTAAGTGAGATACTTATCAAGAATGATGTTAAAAAAACAGATTTCTCATTATGATCTCTATTTGTATTTT
2 micron origin
660 680 700 720 740

AATGTAGAGGTCGAGTTTAGATGCAAGTTCAAGGAGCGAAAGGTGGATGGGTAGGTTATATAGGGATATAGCACAGAGATATATAGCAAAGAGATACTTTTGAGCAA
TTACATCTCCAGCTCAAATCTACGTTCAAGTTCCTCGCTTTCCACCTACCCATCCAATATATCCCTATATCGTGTCTCTATATATCGTTTCTCTATGAAAACTCGTT
2 micron origin
760 780 800 820 840
```

TGTTTGTGGAAGCGGTATTCGCAATATTTTAGTAGCTCGTTACAGTCCGGTGCGTTTTTGGTTTTTTGAAAGTGCGTCTTCAGAGCGCTTTTGGTTTTCAAAAGCGC
ACAAACACCTTCGCCATAAGCGTTATAAAATCATCGAGCAATGTCAGGCCACGCAAAAACCAAAAAACTTTCACGCAGAAGTCTCGCGAAAACCAAAAGTTTTCGCG
2 micron origin
860 880 900 920 940 960

TCTGAAGTTCCTATACTTTCTAGCTAGAGAATAGGAACTTCGGAATAGGAACTTCAAAGCGTTTCCGAAAACGAGCGCTTCCGAAAATGCAACGCGAGCTGCGCACA
AGACTTCAAGGATATGAAAGATCGATCTCTTATCCTTGAAGCCTTATCCTTGAAGTTTCGCAAAGGCTTTTGCTCGCGAAGGCTTTTACGTTGCGCTCGACGCGTGT
2 micron origin
980 1,000 1,020 1,040 1,060

TACAGCTCACTGTTCACGTCGCACCTATATCTGCGTGTTGCCTGTATATATATATACATGAGAAGAACGGCATAGTGCGTGTTTATGCTTAAATGCGTACTTATATG
ATGTCGAGTGACAAGTGCAGCGTGGATATAGACGCACAACGGACATATATATATATGTACTCTTCTTGCCGTATCACGCACAAATACGAATTTACGCATGAATATAC
2 micron origin
1,080 1,100 1,120 1,140 1,160

CGTCTATTTATGTAGGATGAAAGGTAGTCTAGTACCTCCTGTGATATTATCCCATTCCATGCGGGGTATCGTATGCTTCCTTCAGCACTACCCTTTAGCTGTTCTAT
GCAGATAAATACATCCTACTTTCCATCAGATCATGGAGGACACTATAATAGGGTAAGGTACGCCCCATAGCATACGAAGGAAGTCGTGATGGGAAATCGACAAGATA
2 micron origin
1,180 1,200 1,220 1,240 1,260 1,280

ATGCTGCCACTCCTCAATTGGATTAGTCTCATCCTTCAATGCTATCATTTCCTTTGATATTGGATCGATCCGATGATAAGCTGTCAAACATGAGAATTGGGTAATAA
TACGACGGTGAGGAGTTAACCTAATCAGAGTAGGAAGTTACGATAGTAAAGGAAACTATAACCTAGCTAGGCTACTATTCGACAGTTTGTACTCTTAACCCATTATT
2 micron origin URA3
1,300 1,320 1,340 1,360 1,380

CTGATATAATTAAATTGAAGCTCTAATTTGTGAGTTTAGTATACATGCATTTACTTATAATACAGTTTTTTAGTTTTGCTGGCCGCATCTTCTCAAATATGCTTCCC
GACTATATTAATTTAACTTCGAGATTAAACACTCAAATCATATGTACGTAAATGAATATTATGTCAAAAAATCAAAACGACCGGCGTAGAAGAGTTTATACGAAGGG
URA3
1,400 1,420 1,440 1,460 1,480

AGCCTGCTTTTCTGTAACGTTCACCCTCTACCTTAGCATCCCTTCCCTTTGCAAATAGTCCTCTTCCAACAATAATAATGTCAGATCCTGTAGAGACCACATCATCC
TCGGACGAAAAGACATTGCAAGTGGGAGATGGAATCGTAGGGAAGGGAAACGTTTATCAGGAGAAGGTTGTTATTATTACAGTCTAGGACATCTCTGGTGTAGTAGG
URA3
1,500 1,520 1,540 1,560 1,580 1,600

ACGGTTCTATACTGTTGACCCAATGCGTCTCCCTTGTCATCTAAACCCACACCGGGTGTCATAATCAACCAATCGTAACCTTCATCTCTTCCACCCATGTCTCTTTG
TGCCAAGATATGACAACTGGGTTACGCAGAGGGAACAGTAGATTTGGGTGTGGCCCACAGTATTAGTTGGTTAGCATTGGAAGTAGAGAAGGTGGGTACAGAGAAAC
URA3
1,620 1,640 1,660 1,680 1,700

AGCAATAAAGCCGATAACAAAATCTTTGTCGCTCTTCGCAATGTCAACAGTACCCTTAGTATATTCTCCAGTAGATAGGGAGCCCTTGCATGACAATTCTGCTAACA
TCGTTATTTCGGCTATTGTTTTAGAAACAGCGAGAAGCGTTACAGTTGTCATGGGAATCATATAAGAGGTCATCTATCCCTCGGGAACGTACTGTTAAGACGATTGT

URA3

1,720 1,740 1,760 1,780 1,800

TCAAAAGGCCTCTAGGTTCCTTTGTTACTTCTTCTGCCGCCTGCTTCAAACCGCTAACAATACCTGGGCCCACCACACCGTGTGCATTCGTAATGTCTGCCCATTCT
AGTTTTCCGGAGATCCAAGGAAACAATGAAGAAGACGGCGGACGAAGTTTGGCGATTGTTATGGACCCGGGTGGTGTGGCACACGTAAGCATTACAGACGGGTAAGA

URA3

1,820 1,840 1,860 1,880 1,900 1,920

GCTATTCTGTATACACCCGCAGAGTACTGCAATTTGACTGTATTACCAATGTCAGCAAATTTTCTGTCTTCGAAGAGTAAAAAATTGTACTTGGCGGATAATGCCTT
CGATAAGACATATGTGGGCGTCTCATGACGTTAAACTGACATAATGGTTACAGTCGTTTAAAAGACAGAAGCTTCTCATTTTTTAACATGAACCGCCTATTACGGAA

URA3

1,940 1,960 1,980 2,000 2,020

TAGCGGCTTAACTGTGCCCTCCATGGAAAAATCAGTCAAGATATCCACATGTGTTTTTAGTAAACAAATTTTGGGACCTAATGCTTCAACTAACTCCAGTAATTCCT
ATCGCCGAATTGACACGGGAGGTACCTTTTTAGTCAGTTCTATAGGTGTACACAAAAATCATTTGTTTAAAACCCTGGATTACGAAGTTGATTGAGGTCATTAAGGA

URA3

2,040 2,060 2,080 2,100 2,120 2,140

TGGTGGTACGAACATCCAATGAAGCACACAAGTTTGTTTGCTTTTCGTGCATGATATTAAATAGCTTGGCAGCAACAGGACTAGGATGAGTAGCAGCACGTTCCTTA
ACCACCATGCTTGTAGGTTACTTCGTGTGTTCAAACAAACGAAAAGCACGTACTATAATTTATCGAACCGTCGTTGTCCTGATCCTACTCATCGTCGTGCAAGGAAT

URA3

2,160 2,180 2,200 2,220 2,240

TATGTAGCTTTCGACATGATTTATCTTCGTTTCCTGCATGTTTTTGTTCTGTGCAGTTGGGTTAAGAATACTGGGCAATTTCATGTTTCTTCAACACTACATATGCG
ATACATCGAAAGCTGTACTAAATAGAAGCAAAGGACGTACAAAAACAAGACACGTCAACCCAATTCTTATGACCCGTTAAAGTACAAAGAAGTTGTGATGTATACGC

URA3

2,260 2,280 2,300 2,320 2,340

TATATATACCAATCTAAGTCTGTGCTCCTTCCTTCGTTCTTCCTTCTGTTCGGAGATTACCGAATCAAAAAAATTTCAAAGAAACCGAAATCAAAAAAAAGAATAAA
ATATATATGGTTAGATTCAGACACGAGGAAGGAAGCAAGAAGGAAGACAAGCCTCTAATGGCTTAGTTTTTTTAAAGTTTCTTTGGCTTTAGTTTTTTTTCTTATTT

URA3

2,360 2,380 2,400 2,420 2,440 2,460

AAAAAAATGATGAATTGAATTGAAAAGCTAATTCTTGAAGACGAAAGGGCCTCGTGATACGCCTATTTTTATAGGTTAATGTCATGATAATAATGGTTTCTTAGACG
TTTTTTTACTACTTAACTTAACTTTTCGATTAAGAACTTCTGCTTTCCCGGAGCACTATGCGGATAAAAATATCCAATTACAGTACTATTATTACCAAAGAATCTGC

URA3

2,480 2,500 2,520 2,540 2,560

TCAGGTGGCACTTTTCGGGGAAATGTGCGCGGAACCCCTATTTGTTTATTTTTCTAAATACATTCAAATATGTATCCGCTCATGAGACAATAACCCTGATAAATGCT
AGTCCACCGTGAAAAGCCCCTTTACACGCGCCTTGGGGATAAACAAATAAAAAGATTTATGTAAGTTTATACATAGGCGAGTACTCTGTTATTGGGACTATTTACGA

2,580 2,600 2,620 2,640 2,660

TCAATAATATTGAAAAAGGAAGAGTATGAGTATTCAACATTTCCGTGTCGCCCTTATTCCCTTTTTTGCGGCATTTTGCCTTCCTGTTTTTGCTCACCCAGAAACGC
AGTTATTATAACTTTTTCCTTCTCATACTCATAAGTTGTAAAGGCACAGCGGGAATAAGGGAAAAAACGCCGTAAAACGGAAGGACAAAAACGAGTGGGTCTTTGCG

2,680 2,700 2,720 2,740 2,760 2,780

TGGTGAAAGTAAAAGATGCTGAAGATCAGTTGGGTGCACGAGTGGGTTACATCGAACTGGATCTCAACAGCGGTAAGATCCTTGAGAGTTTTCGCCCCGAAGAACGT
ACCACTTTCATTTTCTACGACTTCTAGTCAACCCACGTGCTCACCCAATGTAGCTTGACCTAGAGTTGTCGCCATTCTAGGAACTCTCAAAAGCGGGGCTTCTTGCA

2,800 2,820 2,840 2,860 2,880

TTTCCAATGATGAGCACTTTTAAAGTTCTGCTATGTGGCGCGGTATTATCCCGTATTGACGCCGGGCAAGAGCAACTCGGTCGCCGCATACACTATTCTCAGAATGA
AAAGGTTACTACTCGTGAAAATTTCAAGACGATACACCGCGCCATAATAGGGCATAACTGCGGCCCGTTCTCGTTGAGCCAGCGGCGTATGTGATAAGAGTCTTACT

AmpR

2,900 2,920 2,940 2,960 2,980

CTTGGTTGAGTACTCACCAGTCACAGAAAAGCATCTTACGGATGGCATGACAGTAAGAGAATTATGCAGTGCTGCCATAACCATGAGTGATAACACTGCGGCCAACT
GAACCAACTCATGAGTGGTCAGTGTCTTTTCGTAGAATGCCTACCGTACTGTCATTCTCTTAATACGTCACGACGGTATTGGTACTCACTATTGTGACGCCGGTTGA

AmpR

3,000 3,020 3,040 3,060 3,080 3,100

TACTTCTGACAACGATCGGAGGACCGAAGGAGCTAACCGCTTTTTTGCACAACATGGGGGATCATGTAACTCGCCTTGATCGTTGGGAACCGGAGCTGAATGAAGCC
ATGAAGACTGTTGCTAGCCTCCTGGCTTCCTCGATTGGCGAAAAAACGTGTTGTACCCCCTAGTACATTGAGCGGAACTAGCAACCCTTGGCCTCGACTTACTTCGG

AmpR

3,120 3,140 3,160 3,180 3,200

ATACCAAACGACGAGCGTGACACCACGATGCCTGTAGCAATGGCAACAACGTTGCGCAAACTATTAACTGGCGAACTACTTACTCTAGCTTCCCGGCAACAATTAAT
TATGGTTTGCTGCTCGCACTGTGGTGCTACGGACATCGTTACCGTTGTTGCAACGCGTTTGATAATTGACCGCTTGATGAATGAGATCGAAGGGCCGTTGTTAATTA

AmpR

3,220 3,240 3,260 3,280 3,300

AGACTGGATGGAGGCGGATAAAGTTGCAGGACCACTTCTGCGCTCGGCCCTTCCGGCTGGCTGGTTTATTGCTGATAAATCTGGAGCCGGTGAGCGTGGGTCTCGCG
TCTGACCTACCTCCGCCTATTTCAACGTCCTGGTGAAGACGCGAGCCGGGAAGGCCGACCGACCAAATAACGACTATTTAGACCTCGGCCACTCGCACCCAGAGCGC

AmpR

3,320 3,340 3,360 3,380 3,400 3,420

GTATCATTGCAGCACTGGGGCCAGATGGTAAGCCCTCCCGTATCGTAGTTATCTACACGACGGGGAGTCAGGCAACTATGGATGAACGAAATAGACAGATCGCTGAG
CATAGTAACGTCGTGACCCCGGTCTACCATTCGGGAGGGCATAGCATCAATAGATGTGCTGCCCCTCAGTCCGTTGATACCTACTTGCTTTATCTGTCTAGCGACTC

AmpR

3,440 3,460 3,480 3,500 3,520

ATAGGTGCCTCACTGATTAAGCATTGGTAACTGTCAGACCAAGTTTACTCATATATACTTTAGATTGATTTAAAACTTCATTTTTAATTTAAAAGGATCTAGGTGAA
TATCCACGGAGTGACTAATTCGTAACCATTGACAGTCTGGTTCAAATGAGTATATATGAAATCTAACTAAATTTTGAAGTAAAAATTAAATTTTCCTAGATCCACTT

AmpR

3,540 3,560 3,580 3,600 3,620

GATCCTTTTTGATAATCTCATGACCAAAATCCCTTAACGTGAGTTTTCGTTCCACTGAGCGTCAGACCCCGTAGAAAAGATCAAAGGATCTTCTTGAGATCCTTTTT
CTAGGAAAAACTATTAGAGTACTGGTTTTAGGGAATTGCACTCAAAAGCAAGGTGACTCGCAGTCTGGGGCATCTTTTCTAGTTTCCTAGAAGAACTCTAGGAAAAA
ColE1 origin
3,640 3,660 3,680 3,700 3,720 3,740

TTCTGCGCGTAATCTGCTGCTTGCAAACAAAAAAACCACCGCTACCAGCGGTGGTTTGTTTGCCGGATCAAGAGCTACCAACTCTTTTTCCGAAGGTAACTGGCTTC
AAGACGCGCATTAGACGACGAACGTTTGTTTTTTTGGTGGCGATGGTCGCCACCAAACAAACGGCCTAGTTCTCGATGGTTGAGAAAAAGGCTTCCATTGACCGAAG
ColE1 origin
3,760 3,780 3,800 3,820 3,840

AGCAGAGCGCAGATACCAAATACTGTCCTTCTAGTGTAGCCGTAGTTAGGCCACCACTTCAAGAACTCTGTAGCACCGCCTACATACCTCGCTCTGCTAATCCTGTT
TCGTCTCGCGTCTATGGTTTATGACAGGAAGATCACATCGGCATCAATCCGGTGGTGAAGTTCTTGAGACATCGTGGCGGATGTATGGAGCGAGACGATTAGGACAA
ColE1 origin
3,860 3,880 3,900 3,920 3,940

ACCAGTGGCTGCTGCCAGTGGCGATAAGTCGTGTCTTACCGGGTTGGACTCAAGACGATAGTTACCGGATAAGGCGCAGCGGTCGGGCTGAACGGGGGGTTCGTGCA
TGGTCACCGACGACGGTCACCGCTATTCAGCACAGAATGGCCCAACCTGAGTTCTGCTATCAATGGCCTATTCCGCGTCGCCAGCCCGACTTGCCCCCCAAGCACGT
ColE1 origin
3,960 3,980 4,000 4,020 4,040 4,060

CACAGCCCAGCTTGGAGCGAACGACCTACACCGAACTGAGATACCTACAGCGTGAGCTATGAGAAAGCGCCACGCTTCCCGAAGGGAGAAAGGCGGACAGGTATCCG
GTGTCGGGTCGAACCTCGCTTGCTGGATGTGGCTTGACTCTATGGATGTCGCACTCGATACTCTTTCGCGGTGCGAAGGGCTTCCCTCTTTCCGCCTGTCCATAGGC
ColE1 origin
4,080 4,100 4,120 4,140 4,160

GTAAGCGGCAGGGTCGGAACAGGAGAGCGCACGAGGGAGCTTCCAGGGGGAAACGCCTGGTATCTTTATAGTCCTGTCGGGTTTCGCCACCTCTGACTTGAGCGTCG
CATTCGCCGTCCCAGCCTTGTCCTCTCGCGTGCTCCCTCGAAGGTCCCCCTTTGCGGACCATAGAAATATCAGGACAGCCCAAAGCGGTGGAGACTGAACTCGCAGC
ColE1 origin
4,180 4,200 4,220 4,240 4,260 4,280

ATTTTTGTGATGCTCGTCAGGGGGGCGGAGCCTATGGAAAAACGCCAGCAACGCGGCCTTTTTACGGTTCCTGGCCTTTTGCTGGCCTTTTGCTCACATGTTCTTTC
TAAAAACACTACGAGCAGTCCCCCCGCCTCGGATACCTTTTTGCGGTCGTTGCGCCGGAAAAATGCCAAGGACCGGAAAACGACCGGAAAACGAGTGTACAAGAAAG
ColE1 origin
4,300 4,320 4,340 4,360 4,380

CTGCGTTATCCCCTGATTCTGTGGATAACCGTATTACCGCCTTTGAGTGAGCTGATACCGCTCGCCGCAGCCGAACGACCGAGCGCAGCGAGTCAGTGAGCGAGGAA
GACGCAATAGGGGACTAAGACACCTATTGGCATAATGGCGGAAACTCACTCGACTATGGCGAGCGGCGTCGGCTTGCTGGCTCGCGTCGCTCAGTCACTCGCTCCTT
4,400 4,420 4,440 4,460 4,480

GCGGAAGAGCGCCCAATACGCAAACCGCCTCTCCCCGCGCGTTGGCCGATTCATTAATGCAGCTGGCACGACAGGTTTCCCGACTGGAAAGCGGGCAGTGAGCGCAA
CGCCTTCTCGCGGGTTATGCGTTTGGCGGAGAGGGGCGCGCAACCGGCTAAGTAATTACGTCGACCGTGCTGTCCAAAGGGCTGACCTTTCGCCCGTCACTCGCGTT
4,500 4,520 4,540 4,560 4,580 4,600

CGCAATTAATGTGAGTTAGCTCACTCATTAGGCACCCCAGGCTTTACACTTTATGCTTCCGGCTCGTATGTTGTGTGGAATTGTGAGCGGATAACAATTTCACACAG
GCGTTAATTACACTCAATCGAGTGAGTAATCCGTGGGGTCCGAAATGTGAAATACGAAGGCCGAGCATACAACACACCTTAACACTCGCCTATTGTTAAAGTGTGTC

lac promoter | LacO

4,620 4,640 4,660 4,680 4,700

GAAACAGCTATGACCATGATTACGCCAAGCTTCGGATTAGAAGCCGCCGAGCGGGTGACAGCCCTCCGAAGGAAGACTCTCCTCCGTGCGTCCTCGTCTTCACCGGT
CTTTGTCGATACTGGTACTAATGCGGTTCGAAGCCTAATCTTCGGCGGCTCGCCCACTGTCGGGAGGCTTCCTTCTGAGAGGAGGCACGCAGGAGCAGAAGTGGCCA

M13-rev | GAL1 promoter

4,720 4,740 4,760 4,780 4,800

CGCGTTCCTGAAACGCAGATGTGCCTCGCGCCGCACTGCTCCGAACAATAAAGATTCTACAATACTAGCTTTTATGGTTATGAAGAGGAAAAATTGGCAGTAACCTG
GCGCAAGGACTTTGCGTCTACACGGAGCGCGGCGTGACGAGGCTTGTTATTTCTAAGATGTTATGATCGAAAATACCAATACTTCTCCTTTTTAACCGTCATTGGAC

GAL1 promoter

4,820 4,840 4,860 4,880 4,900 4,920

GCCCCACAAACCTTCAAATGAACGAATCAAATTAACAACCATAGGATGATAATGCGATTAGTTTTTTAGCCTTATTTCTGGGGTAATTAATCAGCGAAGCGATGATT
CGGGGTGTTTGGAAGTTTACTTGCTTAGTTTAATTGTTGGTATCCTACTATTACGCTAATCAAAAAATCGGAATAAAGACCCCATTAATTAGTCGCTTCGCTACTAA

GAL1 promoter

4,940 4,960 4,980 5,000 5,020

TTTGATCTATTAACAGATATATAAATGCAAAAACTGCATAACCACTTTAACTAATACTTTCAACATTTTCGGTTTGTATTACTTCTTATTCAAATGTAATAAAAGTA
AAACTAGATAATTGTCTATATATTTACGTTTTTGACGTATTGGTGAAATTGATTATGAAAGTTGTAAAAGCCAAACATAATGAAGAATAAGTTTACATTATTTTCAT

GAL1 promoter

5,040 5,060 5,080 5,100 5,120

TCAACAAAAAATTGTTAATATACCTCTATACTTTAACGTCAAGGAGAAAAAACCCCGGATCGAATTCAACCCTCACTAAAGGGCGGCCGCCATGAGATTCCCATCTA
AGTTGTTTTTTAACAATTATATGGAGATATGAAATTGCAGTTCCTCTTTTTTGGGGCCTAGCTTAAGTTGGGAGTGATTTCCCGCCGGCGGTACTCTAAGGGTAGAT

1 2 3 4 5
M R F P S

GAL1 promoter

5,140 5,160 5,180 5,200 5,220 5,240

TCTTCACCGCTGTTTTGTTCGCTGCTTCTTCTGCTTTGGCTGCTCCAGCTAACACCACCACCGAAGACGAAACCGCTCAAATCCCAGCTGAAGCTGTTATCGACTAC
AGAAGTGGCGACAAAACAAGCGACGAAGAAGACGAAACCGACGAGGTCGATTGTGGTGGTGGCTTCTGCTTTGGCGAGTTTAGGGTCGACTTCGACAATAGCTGATG

6 7 8 9 10 11 12 13 14 15 16 17 18 19 20 21 22 23 24 25 26 27 28 29 30 31 32 33 34 35 36 37 38 39 40 41
I F T A V L F A A S S A L A A P A N T T T E D E T A Q I P A E A V I D Y

Mating factor alpha leader

5,260 5,280 5,300 5,320 5,340

TCTGACTTGGAAGGTGACTTCGACGCTGCTGCTTTGCCATTGTCTAACTCTACCAACAACGGTTTGTCTTCTACCAACACCACCATCGCTTCTATCGCTGCTAAGGA
AGACTGAACCTTCCACTGAAGCTGCGACGACGAAACGGTAACAGATTGAGATGGTTGTTGCCAAACAGAAGATGGTTGTGGTGGTAGCGAAGATAGCGACGATTCCT
42 43 44 45 46 47 48 49 50 51 52 53 54 55 56 57 58 59 60 61 62 63 64 65 66 67 68 69 70 71 72 73 74 75 76 77
S D L E G D F D A A A L P L S N S T N N G L S S T N T T I A S I A A K E

Mating factor alpha leader

5,360 5,380 5,400 5,420 5,440

AGAAGGTGTTCAATTGGACAAGAGAGAAGCTAGCGCAGATGTGCAGTTGCAAGCATCCGGCGGCGGTTCTGTTCAGGCTGGTGGTTCTCTAAGATTAAGTTGTGCTG
TCTTCCACAAGTTAACCTGTTCTCTCTTCGATCGCGTCTACACGTCAACGTTCGTAGGCCGCCGCCAAGACAAGTCCGACCACCAAGAGATTCTAATTCAACACGAC
78 79 80 81 82 83 84 85 86 2 4 6 8 10 12 14 16 18 20 22
E G V Q L D K R E D V Q L Q A S G G G S V Q A G G S L R L S C A

anti-lys Nb

5,460 5,480 5,500 5,520 5,540 5,560

CTTCAGGTTATACCATCGGCCCATACTGCATGGGTTGGTTTCGTCAAGCTCCAGGCAAAGAAAGGGAGGGAGTCGCAGCCATTAACATGGGCGGCGGTATCACATAT
GAAGTCCAATATGGTAGCCGGGTATGACGTACCCAACCAAAGCAGTTCGAGGTCCGTTTCTTTCCCTCCCTCAGCGTCGGTAATTGTACCCGCCGCCATAGTGTATA
24 26 28 30 32 34 36 38 40 42 44 46 48 50 52 54 56 58
A S G Y T I G P Y C M G W F R Q A P G K E R E G V A A I N M G G G I T Y

anti-lys Nb

5,580 5,600 5,620 5,640 5,660

TATGCAGACTCCGTAAAAGGTAGATTTACTATCTCTCAGGACAATGCTAAGAACACGGTCTATCTGTTAATGAATTCATTGGAGCCCGAGGACACTGCCATATACTA
ATACGTCTGAGGCATTTTCCATCTAAATGATAGAGAGTCCTGTTACGATTCTTGTGCCAGATAGACAATTACTTAAGTAACCTCGGGCTCCTGTGACGGTATATGAT
60 62 64 66 68 70 72 74 76 78 80 82 84 86 88 90 92 94
Y A D S V K G R F T I S Q D N A K N T V Y L L M N S L E P E D T A I Y Y

anti-lys Nb

5,680 5,700 5,720 5,740 5,760

CTGTGCCGCAGATTCAACCATTTACGCTTCCTATTACGAATGCGGACACGGATTAAGTACTGGAGGTTACGGTTATGATTCCTGGGGTCAAGGCACACAAGTCACGG
GACACGGCGTCTAAGTTGGTAAATGCGAAGGATAATGCTTACGCCTGTGCCTAATTCATGACCTCCAATGCCAATACTAAGGACCCCAGTTCCGTGTGTTCAGTGCC
96 98 100 102 104 106 108 110 112 114 116 118 120 122 124 126 128 130
C A A D S T I Y A S Y Y E C G H G L S T G G Y G Y D S W G Q G T Q V T

anti-lys Nb

5,780 5,800 5,820 5,840 5,860 5,880

TATCATCAGGATCCTACCCATACGATGTTCCAGATTACGCTCAAACATCCTCACCCTCTACAGAAGCATCAGTATCAACCTCTAGTACCTCTTCCTCTGCTTCACAA
ATAGTAGTCCTAGGATGGGTATGCTACAAGGTCTAATGCGAGTTTGTAGGAGTGGGAGATGTCTTCGTAGTCATAGTTGGAGATCATGGAGAAGGAGACGAAGTGTT
132 1 2 3 4 5 6 7 8 9 2 4 6 8 10 12 14 16 18 20 22
V S S Y P Y D V P D Y A Q T S S P S T E A S V S T S S T S S S A S Q

HA tag 649 stalk

5,900 5,920 5,940 5,960 5,980

```
TCATCTGACCCAACTACAACATCTTCGTCCAGTTCATCGTCTTCCCCATCTTCCCAATCTGAGGAAATTTCATCATCACCGACGGTCTCAACTACACCATCAACTTC
AGTAGACTGGGTTGATGTTGTAGAAGCAGGTCAAGTAGCAGAAGGGGTAGAAGGGTTAGACTCCTTTAAAGTAGTAGTGGCTGCCAGAGTTGATGTGGTAGTTGAAG
     24      26      28      30      32      34      36      38      40      42      44      46      48      50      52      54      56      58
 S  S  D  P  T  T  T  S  S  S  S  S  S  S  P  S  S  Q  S  E  E  I  S  S  S  P  T  V  S  T  T  P  S  T  S
649 stalk
     6,000     6,020     6,040     6,060     6,080

TTCATCATCTTCCTCAATGACTTCAACCACCACAACAAAGTCAATCTCAACTTCCACTACAAGTTCAGCTCCAGTTACAGATGTGACAGTTTCCTCATCGCCTAGTA
AAGTAGTAGAAGGAGTTACTGAAGTTGGTGGTGTTGTTTCAGTTAGAGTTGAAGGTGATGTTCAAGTCGAGGTCAATGTCTACACTGTCAAAGGAGTAGCGGATCAT
     60      62      64      66      68      70      72      74      76      78      80      82      84      86      88      90      92
 S  S  S  S  S  M  T  S  T  T  T  T  K  S  I  S  T  S  T  T  S  S  A  P  V  T  D  V  T  V  S  S  S  P  S
649 stalk
6,100     6,120     6,140     6,160     6,180     6,200

AATCTACCTCTACTTCGACAAGTACAGAAACATCTAAAACACCTACTTCAATGACAGAGTATACATCTAGTACATCGATAATTTCGACTCCAGTTAGTCACTCGCAG
TTAGATGGAGATGAAGCTGTTCATGTCTTTGTAGATTTTGTGGATGAAGTTACTGTCTCATATGTAGATCATGTAGCTATTAAAGCTGAGGTCAATCAGTGAGCGTC
94      96      98      100      102      104      106      108      110      112      114      116      118      120      122      124      126      128
K  S  T  S  T  S  T  S  T  E  T  S  K  T  P  T  S  M  T  E  Y  T  S  S  T  S  I  I  S  T  P  V  S  H  S  Q
649 stalk
     6,220     6,240     6,260     6,280     6,300

ACAGGTTTGTCGGCTTCATCAAGTTCATCATCTACAACATCCGGTTCTTCGTCCACTAAATCAGAAAGTTCGACAACATCTGGCTCTTCCCAGTCCGTGGAATCAAC
TGTCCAAACAGCCGAAGTAGTTCAAGTAGTAGATGTTGTAGGCCAAGAAGCAGGTGATTTAGTCTTTCAAGCTGTTGTAGACCGAGAAGGGTCAGGCACCTTAGTTG
130      132      134      136      138      140      142      144      146      148      150      152      154      156      158      160      162      164
T  G  L  S  A  S  S  S  S  S  S  T  T  S  G  S  S  S  T  K  S  E  S  S  T  T  S  G  S  S  Q  S  V  E  S  T
649 stalk
     6,320     6,340     6,360     6,380     6,400     6,420

CTCCAGCCACGCCACTGTTCTTGCTAATTCCGCAGAAATGGTCACAACATCCTCTAGTTCATCCTCAACATCCGAAATGTCATTAACTAGTACTGCTACCAGTGTAC
GAGGTCGGTGCGGTGACAAGAACGATTAAGGCGTCTTTACCAGTGTTGTAGGAGATCAAGTAGGAGTTGTAGGCTTTACAGTAATTGATCATGACGATGGTCACATG
 166      168      170      172      174      176      178      180      182      184      186      188      190      192      194      196      198      200
 S  S  H  A  T  V  L  A  N  S  A  E  M  V  T  T  S  S  S  S  S  S  T  S  E  M  S  L  T  S  T  A  T  S  V
649 stalk
          6,440     6,460     6,480     6,500     6,520

CAGTCTCATCTAGTAGCAGTACGACATATTCTACTAGCGCATCTACACAAGCCGTCACTACAACATCTTCTTCCACTGTATCTACAACTTCTTCTAGTACAACGTTA
GTCAGAGTAGATCATCGTCATGCTGTATAAGATGATCGCGTAGATGTGTTCGGCAGTGATGTTGTAGAAGAAGGTGACATAGATGTTGAAGAAGATCATGTTGCAAT
 202      204      206      208      210      212      214      216      218      220      222      224      226      228      230      232      234      236
P  V  S  S  S  S  S  T  T  Y  S  T  S  A  S  T  Q  A  V  T  T  T  S  S  S  T  V  S  T  T  S  S  S  T  T  L
649 stalk
     6,540     6,560     6,580     6,600     6,620
```

```
ACAAGCGCATTCACACATTCTTCAACCACATCGTCCGACCAGCCACCCAGCGACACTACAAGTCCATCTACGACACACGAACCTCATGTAACCACTCAGACGTCATC
TGTTCGCGTAAGTGTGTAAGAAGTTGGTGTAGCAGGCTGGTCGGTGGGTCGCTGTGATGTTCAGGTAGATGCTGTGTGCTTGGAGTACATTGGTGAGTCTGCAGTAG
   238    240    242    244    246    248    250    252    254    256    258    260    262    264    266    268    270    272
T S A F T H S S T T S S D Q P P S D T T S P S T T H E P H V T T Q T S S
649 stalk
6,640 6,660 6,680 6,700 6,720 6,740
```

```
AGAAACATCTTCTTCTAAGTCATCTTCTACTTCTTCTTCAAGTACATCTCAAACCTCTGAGTCTGCAACACCATCCGATTCCGTATCACCTGGAAGTTCTACATCAA
TCTTTGTAGAAGAAGATTCAGTAGAAGATGAAGAAGAAGTTCATGTAGAGTTTGGAGACTCAGACGTTGTGGTAGGCTAAGGCATAGTGGACCTTCAAGATGTAGTT
   274    276    278    280    282    284    286    288    290    292    294    296    298    300    302    304    306
E T S S S K S S S T S S S S T S Q T S E S A T P S D S V S P G S S T S
649 stalk
6,760 6,780 6,800 6,820 6,840
```

```
CATCTTCTAGTAGCACTTCTACTTCCACTTCTATTTCCAGTGGAGAAACGACAACTTCTTCTTCTTCATCATCTGCCACGACCACTTCTAACAGCGCAACCTTGTCA
GTAGAAGATCATCGTGAAGATGAAGGTGAAGATAAAGGTCACCTCTTTGCTGTTGAAGAAGAAGAAGTAGTAGACGGTGCTGGTGAAGATTGTCGCGTTGGAACAGT
308    310    312    314    316    318    320    322    324    326    328    330    332    334    336    338    340    342
T S S S S T S T S T S I S S G E T T T S S S S S S A T T T S N S A T L S
649 stalk
6,860 6,880 6,900 6,920 6,940
```

```
GTCTCTACCACACAAACTTCGATTGAAGCCAGTTCATCTACTACATCTACATCTAGTTCAACAATTACAACTTCAAGTAGTAGCGCTCACATATCGTCGAAATCTCA
CAGAGATGGTGTGTTTGAAGCTAACTTCGGTCAAGTAGATGATGTAGATGTAGATCAAGTTGTTAATGTTGAAGTTCATCATCGCGAGTGTATAGCAGCTTTAGAGT
344    346    348    350    352    354    356    358    360    362    364    366    368    370    372    374    376    378
V S T T Q T S I E A S S S T T S T S S S T I T T S S S S A H I S S K S Q
649 stalk
6,960 6,980 7,000 7,020 7,040 7,060
```

```
ATCTAGTATTACCTATCCCTCTTCCTCGACATCTTCATCTACATCGTCCTCAATTTCTAGCGAATCTGAAAGTTTTGAATCGACATCAGCAGAAGATGCTCCATCAA
TAGATCATAATGGATAGGGAGAAGGAGCTGTAGAAGTAGATGTAGCAGGAGTTAAAGATCGCTTAGACTTTCAAAACTTAGCTGTAGTCGTCTTCTACGAGGTAGTT
 380    382    384    386    388    390    392    394    396    398    400    402    404    406    408    410    412    414
S S I T Y P S S S T S S S T S S S I S S E S E S F E S T S A E D A P S
649 stalk
7,080 7,100 7,120 7,140 7,160
```

```
CAGCACCTTCATCAAGTGTCTCTTCTAAGAGTTCTACCTCTACAACATCAAGCACATCGACATCTTCAAGCACTCCATCTCCATCACCATCTTCCGTGAGTTCTTCC
GTCGTGGAAGTAGTTCACAGAGAAGATTCTCAAGATGGAGATGTTGTAGTTCGTGTAGCTGTAGAAGTTCGTGAGGTAGAGGTAGTGGTAGAAGGCACTCAAGAAGG
  416    418    420    422    424    426    428    430    432    434    436    438    440    442    444    446    448    450
T A P S S S V S S K S S T S T T S S T S T S S S T P S P S P S S V S S S
649 stalk
7,180 7,200 7,220 7,240 7,260
```

TCCACCAGCTCATTGACAACTTCTGCTGTATCAACACCAGCTACCTCTCATTCTCAAAGTACTGTAGTAACCACCACTACTATTACTACATCAACAGGTCCAGTGAT
AGGTGGTCGAGTAACTGTTGAAGACGACATAGTTGTGGTCGATGGAGAGTAAGAGTTTCATGACATCATTGGTGGTGATGATAATGATGTAGTTGTCCAGGTCACTA
452 454 456 458 460 462 464 466 468 470 472 474 476 478 480 482 484 486
S T S S L T T S A V S T P A T S H S Q S T V V T T T T I T T S T G P V M
649 stalk
7,280 7,300 7,320 7,340 7,360 7,380

GTCTACGACAACAGCTTATTCTTCTAGTTCTACTAGCAGCTCGGAATCTTCTGAGGTTCAGTCTGTCATGTCATCTACGCCTAGTTCAACATCAACAACAACCAGTT
CAGATGCTGTTGTCGAATAAGAAGATCAAGATGATCGTCGAGCCTTAGAAGACTCCAAGTCAGACAGTACAGTAGATGCGGATCAAGTTGTAGTTGTTGTTGGTCAA
488 490 492 494 496 498 500 502 504 506 508 510 512 514 516 518 520
S T T T A Y S S S S T S S S E S S E V Q S V M S S T P S S T S T T T S
649 stalk
7,400 7,420 7,440 7,460 7,480

CGGAATCTACTTCATCTAGCTCCACAGCTTCTACCTCACCATCAACCTCGCAAACTTTCGAAACTTCTCCTACTATAGGAGGTGTCCCCTCAACCACTTCATTTGTC
GCCTTAGATGAAGTAGATCGAGGTGTCGAAGATGGAGTGGTAGTTGGAGCGTTTGAAAGCTTTGAAGAGGATGATATCCTCCACAGGGGAGTTGGTGAAGTAAACAG
522 524 526 528 530 532 534 536 538 540 542 544 546 548 550 552 554 556
S E S T S S S S T A S T S P S T S Q T F E T S P T I G G V P S T T S F V
649 stalk
7,500 7,520 7,540 7,560 7,580

TCTACGCCAACAACGAAATTGTCGCACACTACTTCCACTATGACAGCACAGTCCGATAGTAAGTCTACCCACTCCTCAAGCACATCGACAGAAGATAAATCATCCAC
AGATGCGGTTGTTGCTTTAACAGCGTGTGATGAAGGTGATACTGTCGTGTCAGGCTATCATTCAGATGGGTGAGGAGTTCGTGTAGCTGTCTTCTATTTAGTAGGTG
558 560 562 564 566 568 570 572 574 576 578 580 582 584 586 588 590 592
S T P T T K L S H T T S T M T A Q S D S K S T H S S S T S T E D K S S T
649 stalk
7,600 7,620 7,640 7,660 7,680 7,700

TGCTTCTGCAGTTGACGAAAGCACTACAACATCCACTTCCACGGAGTCTACTACATCAGTAACATCAGGCACCTCCCATTCCGCTAAAGAATCTTCGTCAAATTCTA
ACGAAGACGTCAACTGCTTTCGTGATGTTGTAGGTGAAGGTGCCTCAGATGATGTAGTCATTGTAGTCCGTGGAGGGTAAGGCGATTTCTTAGAAGCAGTTTAAGAT
594 596 598 600 602 604 606 608 610 612 614 616 618 620 622 624 626 628
A S A V D E S T T T S T S T E S T T S V T S G T S H S A K E S S S N S
649 stalk
7,720 7,740 7,760 7,780 7,800

AGGTGTATAGTTCACAGACAGCACACTCATCCATAAGTGTTGCATCATCACCTAGTACAAAGGGCGCCCAAATCCAATCTTCTATGGTTGAAATCTCTACCTACGCT
TCCACATATCAAGTGTCTGTCGTGTGAGTAGGTATTCACAACGTAGTAGTGGATCATGTTTCCCGCGGGTTTAGGTTAGAAGATACCAACTTTAGAGATGGATGCGA
630 632 634 636 638 640 642 644 646 648 650 652 654 656 658 660 662 664
K V Y S S Q T A H S S I S V A S S P S T K G A Q I Q S S M V E I S T Y A
649 stalk GPI anchor
7,820 7,840 7,860 7,880 7,900

GGTTCTGCTAACTCTGTTAACGCTGGTGCTGGTGCTGGTGCTTTGTTCTTGTTGTTGTCTTTGGCTATCATCTAATGATTAATTAACTCGAGATCTGATAACAACAG
CCAAGACGATTGAGACAATTGCGACCACGACCACGACCACGAAACAAGAACAACAACAGAAACCGATAGTAGATTACTAATTAATTGAGCTCTAGACTATTGTTGTC

666 668 670 672 674 676 678 680 682 684 686 688 1 2 3 4 5 6 7
G S A N S V N A G A G A G A L F L L L S L A I I * * L I N S R

GPI anchor

7,920 7,940 7,960 7,980 8,000 8,020

TGTAGATGTAACAAAATCGACTTTGTTCCCACTGTACTTTTAGCTCGTACAAAATACAATATACTTTTCATTTCTCCGTAAACAACATGTTTTCCCATGTAATATCC
ACATCTACATTGTTTTAGCTGAAACAAGGGTGACATGAAAATCGAGCATGTTTTATGTTATATGAAAAGTAAAGAGGCATTTGTTGTACAAAAGGGTACATTATAGG

alpha factor terminator

8,040 8,060 8,080 8,100 8,120

TTTTCTATTTTTCGTTCCGTTACCAACTTTACACATACTTTATATAGCTATTCACTTCTATACACTAAAAAACTAAGACAATTTTAATTTTGCTGCCTGCCATATTT
AAAAGATAAAAAGCAAGGCAATGGTTGAAATGTGTATGAAATATATCGATAAGTGAAGATATGTGATTTTTTGATTCTGTTAAAATTAAAACGACGGACGGTATAAA

alpha factor terminator

8,140 8,160 8,180 8,200 8,220

CAATTTGTTATAAATTCCTATAATTTATCCTATTAGTAGCTAAAAAAAGATGAATGTGAATCGAATCCTAAGAGAATTGGTACCGCGATGTAGTAAAACTAGCTAGA
GTTAAACAATATTTAAGGATATTAAATAGGATAATCATCGATTTTTTTCTACTTACACTTAGCTTAGGATTCTCTTAACCATGGCGCTACATCATTTTGATCGATCT

alpha factor terminator

8,240 8,260 8,280 8,300 8,320 8,340

CCGAGAAAGAGACTAGAAATGCAAAAGGCACTTCTACAATGGCTGCCATCATTATTATCCGATGTGACGCTGCATTTTTTTTTTTTTTTTTTTTTTTTTTTTTTTTT
GGCTCTTTCTCTGATCTTTACGTTTTCCGTGAAGATGTTACCGACGGTAGTAATAATAGGCTACACTGCGACGTAAAAAAAAAAAAAAAAAAAAAAAAAAAAAAAA

8,360 8,380 8,400 8,420 8,440

TTTTTTTTTTTTTTGTACAAATATCATAAAAAAAGAGAATCTTTTTAAGCAAGGATTTTCTTAACTTCTTCGGCGACAGCATCACCGACTTCGGTGGTACTGTTGGA
AAAAAAAAAAAAAACATGTTTATAGTATTTTTTTCTCTTAGAAAAATTCGTTCCTAAAAGAATTGAAGAAGCCGCTGTCGTAGTGGCTGAAGCCACCATGACAACCT

8,460 8,480 8,500 8,520 8,540 8,560

ACCACCTAAATCACCAGTTCTGATACCTGCATCCAAAACCTTTTTAACTGCATCTTCAATGGCCTTACCTTCTTCAGGCAAGTTCAATGACAATTTCAACATCATTG
TGGTGGATTTAGTGGTCAAGACTATGGACGTAGGTTTTGGAAAAATTGACGTAGAAGTTACCGGAATGGAAGAAGTCCGTTCAAGTTACTGTTAAAGTTGTAGTAAC

8,580 8,600 8,620 8,640 8,660

CAGCAGACAAGATAGTGGCGATAGGGTCAACCTTATTCTTTGGCAAATCTGGAGCAGAACCGTGGCATGGTTCGTACAAACCAAATGCGGTGTTCTTGTCTGGCAAA
GTCGTCTGTTCTATCACCGCTATCCCAGTTGGAATAAGAAACCGTTTAGACCTCGTCTTGGCACCGTACCAAGCATGTTTGGTTTACGCCACAAGAACAGACCGTTT

8,680 8,700 8,720 8,740 8,760

GAGGCCAAGGACGCAGATGGCAACAAACCCAAGGAACCTGGGATAACGGAGGCTTCATCGGAGATGATATCACCAAACATGTTGCTGGTGATTATAATACCATTTAG
CTCCGGTTCCTGCGTCTACCGTTGTTTGGGTTCCTTGGACCCTATTGCCTCCGAAGTAGCCTCTACTATAGTGGTTTGTACAACGACCACTAATATTATGGTAAATC

8,780 8,800 8,820 8,840 8,860 8,880

GTGGGTTGGGTTCTTAACTAGGATCATGGCGGCAGAATCAATCAATTGATGTTGAACCTTCAATGTAGGAAATTCGTTCTTGATGGTTTCCTCCACAGTTTTTCTCC
CACCCAACCCAAGAATTGATCCTAGTACCGCCGTCTTAGTTAGTTAACTACAACTTGGAAGTTACATCCTTTAAGCAAGAACTACCAAAGGAGGTGTCAAAAAGAGG

8,900 8,920 8,940 8,960 8,980

ATAATCTTGAAGAGGCCAAAACATTAGCTTTATCCAAGGACCAAATAGGCAATGGTGGCTCATGTTGTAGGGCCATGAAAGCGGCCATTCTTGTGATTCTTTGCACT
TATTAGAACTTCTCCGGTTTTGTAATCGAAATAGGTTCCTGGTTTATCCGTTACCACCGAGTACAACATCCCGGTACTTTCGCCGGTAAGAACACTAAGAAACGTGA

9,000 9,020 9,040 9,060 9,080

TCTGGAACGGTGTATTGTTCACTATCCCAAGCGACACCATCACCATCGTCTTCCTTTCTCTTACCAAAGTAAATACCTCCCACTAATTCTCTGACAACAACGAAGTC
AGACCTTGCCACATAACAAGTGATAGGGTTCGCTGTGGTAGTGGTAGCAGAAGGAAAGAGAATGGTTTCATTTATGGAGGGTGATTAAGAGACTGTTGTTGCTTCAG

9,100 9,120 9,140 9,160 9,180 9,200

AGTACCTTTAGCAAATTGTGGCTTGATTGGAGATAAGTCTAAAAGAGAGTCGGATGCAAAGTTACATGGTCTTAAGTTGGCGTACAATTGAAGTTCTTTACGGATTT
TCATGGAAATCGTTTAACACCGAACTAACCTCTATTCAGATTTTCTCTCAGCCTACGTTTCAATGTACCAGAATTCAACCGCATGTTAACTTCAAGAAATGCCTAAA

9,220 9,240 9,260 9,280 9,300

TTAGTAAACCTTGTTCAGGTCTAACACTACCTGTACCCCATTTAGGACCACCCACAGCACCTAACAAAACGGCATCAACCTTCTTGGAGGCTTCCAGCGCCTCATCT
AATCATTTGGAACAAGTCCAGATTGTGATGGACATGGGGTAAATCCTGGTGGGTGTCGTGGATTGTTTTGCCGTAGTTGGAAGAACCTCCGAAGGTCGCGGAGTAGA

9,320 9,340 9,360 9,380 9,400

GGAAGTGGGACACCTGTAGCATCGATAGCAGCACCACCAATTAAATGATTTTCGAAATCGAACTTGACATTGGAACGAACATCAGAAATAGCTTTAAGAACCTTAAT
CCTTCACCCTGTGGACATCGTAGCTATCGTCGTGGTGGTTAATTTACTAAAAGCTTTAGCTTGAACTGTAACCTTGCTTGTAGTCTTTATCGAAATTCTTGGAATTA

9,420 9,440 9,460 9,480 9,500 9,520

GGCTTCGGCTGTGATTTCTTGACCAACGTGGTCACCTGGCAAAACGACGATCTTCTTAGGGGCAGACATTACAATGGTATATCCTTGAAATATATATAAAAAAAAAA
CCGAAGCCGACACTAAAGAACTGGTTGCACCAGTGGACCGTTTTGCTGCTAGAAGAATCCCCGTCTGTAATGTTACCATATAGGAACTTTATATATATTTTTTTTTT

9,540 9,560 9,580 9,600 9,620

AAAAAAAAAAAAAAAAAAAAATGCAGCTTCTCAATGATATTCGAATACGCTTTGAGGAGATACAGCCTAATATCCGACAAACTGTTTTACAGATTTACGATCGTACT
TTTTTTTTTTTTTTTTTTTTTACGTCGAAGAGTTACTATAAGCTTATGCGAAACTCCTCTATGTCGGATTATAGGCTGTTTGACAAAATGTCTAAATGCTAGCATGA

9,640 9,660 9,680 9,700 9,720

TGTTACCCATCATTGAATTTTGAACATCCGAACCTGGGAGTTTTCCCTGAAACAGATAGTATATTTGAACCTGTATAATAATATATAGTCTAGCGCTTTACGGAAGA
ACAATGGGTAGTAACTTAAAACTTGTAGGCTTGGACCCTCAAAAGGGACTTTGTCTATCATATAAACTTGGACATATTATTATATATCAGATCGCGAAATGCCTTCT

9,740 9,760 9,780 9,800 9,820 9,840

CAATGTATGTATTTCGGTTCCTGGAGAAACTATTGCATCTATTGCATAGGTAATCTTGCACGTCGCATCCCCGGTTCATTTTCTGCGTTTCCATCTTGCACTTCAAT
GTTACATACATAAAGCCAAGGACCTCTTTGATAACGTAGATAACGTATCCATTAGAACGTGCAGCGTAGGGGCCAAGTAAAAGACGCAAAGGTAGAACGTGAAGTTA

9,860 9,880 9,900 9,920 9,940

AGCATATCTTT
TCGTATAGAAA

9,960

System 5 control strain (9563 bp)

GTTAACGAAGCATCTGTGCTTCATTTTGTAGAACAAAAATGCAACGCGAGAGCGCTAATTTTTCAAACAAAGAATCTGAGCTGCATTTTTACAGAACAGAAATGCAA
CAATTGCTTCGTAGACACGAAGTAAAACATCTTGTTTTTACGTTGCGCTCTCGCGATTAAAAAGTTTGTTTCTTAGACTCGACGTAAAAATGTCTTGTCTTTACGTT

2 micron origin

20 40 60 80 100

CGCGAAAGCGCTATTTTACCAACGAAGAATCTGTGCTTCATTTTTGTAAAACAAAAATGCAACGCGAGAGCGCTAATTTTTCAAACAAAGAATCTGAGCTGCATTTT
GCGCTTTCGCGATAAAATGGTTGCTTCTTAGACACGAAGTAAAAACATTTTGTTTTTACGTTGCGCTCTCGCGATTAAAAAGTTTGTTTCTTAGACTCGACGTAAAA

2 micron origin

120 140 160 180 200

TACAGAACAGAAATGCAACGCGAGAGCGCTATTTTACCAACAAAGAATCTATACTTCTTTTTTGTTCTACAAAAATGCATCCCGAGAGCGCTATTTTTCTAACAAAG
ATGTCTTGTCTTTACGTTGCGCTCTCGCGATAAAATGGTTGTTTCTTAGATATGAAGAAAAAACAAGATGTTTTTACGTAGGGCTCTCGCGATAAAAAGATTGTTTC

2 micron origin

220 240 260 280 300 320

CATCTTAGATTACTTTTTTTCTCCTTTGTGCGCTCTATAATGCAGTCTCTTGATAACTTTTTGCACTGTAGGTCCGTTAAGGTTAGAAGAAGGCTACTTTGGTGTCT
GTAGAATCTAATGAAAAAAAGAGGAAACACGCGAGATATTACGTCAGAGAACTATTGAAAAACGTGACATCCAGGCAATTCCAATCTTCTTCCGATGAAACCACAGA

2 micron origin

340 360 380 400 420

ATTTTCTCTTCCATAAAAAAAGCCTGACTCCACTTCCCGCGTTTACTGATTACTAGCGAAGCTGCGGGTGCATTTTTTCAAGATAAAGGCATCCCCGATTATATTCT
TAAAAGAGAAGGTATTTTTTTCGGACTGAGGTGAAGGGCGCAAATGACTAATGATCGCTTCGACGCCCACGTAAAAAAGTTCTATTTCCGTAGGGGCTAATATAAGA

2 micron origin

440 460 480 500 520

ATACCGATGTGGATTGCGCATACTTTGTGAACAGAAAGTGATAGCGTTGATGATTCTTCATTGGTCAGAAAATTATGAACGGTTTCTTCTATTTTGTCTCTATATAC
TATGGCTACACCTAACGCGTATGAAACACTTGTCTTTCACTATCGCAACTACTAAGAAGTAACCAGTCTTTTAATACTTGCCAAAGAAGATAAAACAGAGATATATG

2 micron origin

540 560 580 600 620 640

TACGTATAGGAAATGTTTACATTTTCGTATTGTTTTCGATTCACTCTATGAATAGTTCTTACTACAATTTTTTTGTCTAAAGAGTAATACTAGAGATAAACATAAAA
ATGCATATCCTTTACAAATGTAAAAGCATAACAAAAGCTAAGTGAGATACTTATCAAGAATGATGTTAAAAAAACAGATTTCTCATTATGATCTCTATTTGTATTTT

2 micron origin

660 680 700 720 740

AATGTAGAGGTCGAGTTTAGATGCAAGTTCAAGGAGCGAAAGGTGGATGGGTAGGTTATATAGGGATATAGCACAGAGATATATAGCAAAGAGATACTTTTGAGCAA
TTACATCTCCAGCTCAAATCTACGTTCAAGTTCCTCGCTTTCCACCTACCCATCCAATATATCCCTATATCGTGTCTCTATATATCGTTTCTCTATGAAAACTCGTT

2 micron origin

760 780 800 820 840

TGTTTGTGGAAGCGGTATTCGCAATATTTTAGTAGCTCGTTACAGTCCGGTGCGTTTTTGGTTTTTTGAAAGTGCGTCTTCAGAGCGCTTTTGGTTTTCAAAAGCGC
ACAAACACCTTCGCCATAAGCGTTATAAAATCATCGAGCAATGTCAGGCCACGCAAAAACCAAAAAACTTTCACGCAGAAGTCTCGCGAAAACCAAAAGTTTTCGCG

2 micron origin

860 880 900 920 940 960

TCTGAAGTTCCTATACTTTCTAGCTAGAGAATAGGAACTTCGGAATAGGAACTTCAAAGCGTTTCCGAAAACGAGCGCTTCCGAAAATGCAACGCGAGCTGCGCACA
AGACTTCAAGGATATGAAAGATCGATCTCTTATCCTTGAAGCCTTATCCTTGAAGTTTCGCAAAGGCTTTTGCTCGCGAAGGCTTTTACGTTGCGCTCGACGCGTGT

2 micron origin

980 1,000 1,020 1,040 1,060

TACAGCTCACTGTTCACGTCGCACCTATATCTGCGTGTTGCCTGTATATATATATATACATGAGAAGAACGGCATAGTGCGTGTTTATGCTTAAATGCGTACTTATATG
ATGTCGAGTGACAAGTGCAGCGTGGATATAGACGCACAACGGACATATATATATATATGTACTCTTCTTGCCGTATCACGCACAAATACGAATTTACGCATGAATATAC

2 micron origin

1,080 1,100 1,120 1,140 1,160

CGTCTATTTATGTAGGATGAAAGGTAGTCTAGTACCTCCTGTGATATTATCCCATTCCATGCGGGGTATCGTATGCTTCCTTCAGCACTACCCTTTAGCTGTTCTAT
GCAGATAAATACATCCTACTTTCCATCAGATCATGGAGGACACTATAATAGGGTAAGGTACGCCCCATAGCATACGAAGGAAGTCGTGATGGGAAATCGACAAGATA

2 micron origin

1,180 1,200 1,220 1,240 1,260 1,280

ATGCTGCCACTCCTCAATTGGATTAGTCTCATCCTTCAATGCTATCATTTCCTTTGATATTGGATCGATCCGATGATAAGCTGTCAAACATGAGAATTGGGTAATAA
TACGACGGTGAGGAGTTAACCTAATCAGAGTAGGAAGTTACGATAGTAAAGGAAACTATAACCTAGCTAGGCTACTATTCGACAGTTTGTACTCTTAACCCATTATT

2 micron origin URA3

1,300 1,320 1,340 1,360 1,380

CTGATATAATTAAATTGAAGCTCTAATTTGTGAGTTTAGTATACATGCATTTACTTATAATACAGTTTTTTAGTTTTGCTGGCCGCATCTTCTCAAATATGCTTCCC
GACTATATTAATTTAACTTCGAGATTAAACACTCAAATCATATGTACGTAAATGAATATTATGTCAAAAAATCAAAACGACCGGCGTAGAAGAGTTTATACGAAGGG

URA3

1,400 1,420 1,440 1,460 1,480

AGCCTGCTTTTCTGTAACGTTCACCCTCTACCTTAGCATCCCTTCCCTTTGCAAATAGTCCTCTTCCAACAATAATAATGTCAGATCCTGTAGAGACCACATCATCC
TCGGACGAAAAGACATTGCAAGTGGGAGATGGAATCGTAGGGAAGGGAAACGTTTATCAGGAGAAGGTTGTTATTATTACAGTCTAGGACATCTCTGGTGTAGTAGG

URA3

1,500 1,520 1,540 1,560 1,580 1,600

ACGGTTCTATACTGTTGACCCAATGCGTCTCCCTTGTCATCTAAACCCACACCGGGTGTCATAATCAACCAATCGTAACCTTCATCTCTTCCACCCATGTCTCTTTG
TGCCAAGATATGACAACTGGGTTACGCAGAGGGAACAGTAGATTTGGGTGTGGCCCACAGTATTAGTTGGTTAGCATTGGAAGTAGAGAAGGTGGGTACAGAGAAAC

URA3

1,620 1,640 1,660 1,680 1,700

AGCAATAAAGCCGATAACAAAATCTTTGTCGCTCTTCGCAATGTCAACAGTACCCTTAGTATATTCTCCAGTAGATAGGGAGCCCTTGCATGACAATTCTGCTAACA
TCGTTATTTCGGCTATTGTTTTAGAAACAGCGAGAAGCGTTACAGTTGTCATGGGAATCATATAAGAGGTCATCTATCCCTCGGGAACGTACTGTTAAGACGATTGT
URA3
1,720 1,740 1,760 1,780 1,800

TCAAAAGGCCTCTAGGTTCCTTTGTTACTTCTTCTGCCGCCTGCTTCAAACCGCTAACAATACCTGGGCCCACCACACCGTGTGCATTCGTAATGTCTGCCCATTCT
AGTTTTCCGGAGATCCAAGGAAACAATGAAGAAGACGGCGGACGAAGTTTGGCGATTGTTATGGACCCGGGTGGTGTGGCACACGTAAGCATTACAGACGGGTAAGA
URA3
1,820 1,840 1,860 1,880 1,900 1,920

GCTATTCTGTATACACCCGCAGAGTACTGCAATTTGACTGTATTACCAATGTCAGCAAATTTTCTGTCTTCGAAGAGTAAAAAATTGTACTTGGCGGATAATGCCTT
CGATAAGACATATGTGGGCGTCTCATGACGTTAAACTGACATAATGGTTACAGTCGTTTAAAAGACAGAAGCTTCTCATTTTTTAACATGAACCGCCTATTACGGAA
URA3
1,940 1,960 1,980 2,000 2,020

TAGCGGCTTAACTGTGCCCTCCATGGAAAAATCAGTCAAGATATCCACATGTGTTTTTAGTAAACAAATTTTGGGACCTAATGCTTCAACTAACTCCAGTAATTCCT
ATCGCCGAATTGACACGGGAGGTACCTTTTTAGTCAGTTCTATAGGTGTACACAAAAATCATTTGTTTAAAACCCTGGATTACGAAGTTGATTGAGGTCATTAAGGA
URA3
2,040 2,060 2,080 2,100 2,120 2,140

TGGTGGTACGAACATCCAATGAAGCACACAAGTTTGTTTGCTTTTCGTGCATGATATTAAATAGCTTGGCAGCAACAGGACTAGGATGAGTAGCAGCACGTTCCTTA
ACCACCATGCTTGTAGGTTACTTCGTGTGTTCAAACAAACGAAAAGCACGTACTATAATTTATCGAACCGTCGTTGTCCTGATCCTACTCATCGTCGTGCAAGGAAT
URA3
2,160 2,180 2,200 2,220 2,240

TATGTAGCTTTCGACATGATTTATCTTCGTTTCCTGCATGTTTTTGTTCTGTGCAGTTGGGTTAAGAATACTGGGCAATTTCATGTTTCTTCAACACTACATATGCG
ATACATCGAAAGCTGTACTAAATAGAAGCAAAGGACGTACAAAAACAAGACACGTCAACCCAATTCTTATGACCCGTTAAAGTACAAAGAAGTTGTGATGTATACGC
URA3
2,260 2,280 2,300 2,320 2,340

TATATATACCAATCTAAGTCTGTGCTCCTTCCTTCGTTCTTCCTTCTGTTCGGAGATTACCGAATCAAAAAAATTTCAAAGAAACCGAAATCAAAAAAAAGAATAAA
ATATATATGGTTAGATTCAGACACGAGGAAGGAAGCAAGAAGGAAGACAAGCCTCTAATGGCTTAGTTTTTTTAAAGTTTCTTTGGCTTTAGTTTTTTTTCTTATTT
URA3
2,360 2,380 2,400 2,420 2,440 2,460

AAAAAAATGATGAATTGAATTGAAAAGCTAATTCTTGAAGACGAAAGGGCCTCGTGATACGCCTATTTTTATAGGTTAATGTCATGATAATAATGGTTTCTTAGACG
TTTTTTTACTACTTAACTTAACTTTTCGATTAAGAACTTCTGCTTTCCCGGAGCACTATGCGGATAAAAATATCCAATTACAGTACTATTATTACCAAAGAATCTGC
URA3
2,480 2,500 2,520 2,540 2,560

TCAGGTGGCACTTTTCGGGGAAATGTGCGCGGAACCCCTATTTGTTTATTTTTCTAAATACATTCAAATATGTATCCGCTCATGAGACAATAACCCTGATAAATGCT
AGTCCACCGTGAAAAGCCCCTTTACACGCGCCTTGGGGATAAACAAATAAAAAGATTTATGTAAGTTTATACATAGGCGAGTACTCTGTTATTGGGACTATTTACGA
2,580 2,600 2,620 2,640 2,660

TCAATAATATTGAAAAAGGAAGAGTATGAGTATTCAACATTTCCGTGTCGCCCTTATTCCCTTTTTTGCGGCATTTTGCCTTCCTGTTTTTGCTCACCCAGAAACGC
AGTTATTATAACTTTTTCCTTCTCATACTCATAAGTTGTAAAGGCACAGCGGGAATAAGGGAAAAAACGCCGTAAAACGGAAGGACAAAAACGAGTGGGTCTTTGCG

2,680 2,700 2,720 2,740 2,760 2,780

TGGTGAAAGTAAAAGATGCTGAAGATCAGTTGGGTGCACGAGTGGGTTACATCGAACTGGATCTCAACAGCGGTAAGATCCTTGAGAGTTTTCGCCCCGAAGAACGT
ACCACTTTCATTTTCTACGACTTCTAGTCAACCCACGTGCTCACCCAATGTAGCTTGACCTAGAGTTGTCGCCATTCTAGGAACTCTCAAAAGCGGGGCTTCTTGCA

2,800 2,820 2,840 2,860 2,880

TTTCCAATGATGAGCACTTTTAAAGTTCTGCTATGTGGCGCGGTATTATCCCGTATTGACGCCGGGCAAGAGCAACTCGGTCGCCGCATACACTATTCTCAGAATGA
AAAGGTTACTACTCGTGAAAATTTCAAGACGATACACCGCGCCATAATAGGGCATAACTGCGGCCCGTTCTCGTTGAGCCAGCGGCGTATGTGATAAGAGTCTTACT

AmpR

2,900 2,920 2,940 2,960 2,980

CTTGGTTGAGTACTCACCAGTCACAGAAAAGCATCTTACGGATGGCATGACAGTAAGAGAATTATGCAGTGCTGCCATAACCATGAGTGATAACACTGCGGCCAACT
GAACCAACTCATGAGTGGTCAGTGTCTTTTCGTAGAATGCCTACCGTACTGTCATTCTCTTAATACGTCACGACGGTATTGGTACTCACTATTGTGACGCCGGTTGA

AmpR

3,000 3,020 3,040 3,060 3,080 3,100

TACTTCTGACAACGATCGGAGGACCGAAGGAGCTAACCGCTTTTTTGCACAACATGGGGGATCATGTAACTCGCCTTGATCGTTGGGAACCGGAGCTGAATGAAGCC
ATGAAGACTGTTGCTAGCCTCCTGGCTTCCTCGATTGGCGAAAAAACGTGTTGTACCCCCTAGTACATTGAGCGGAACTAGCAACCCTTGGCCTCGACTTACTTCGG

AmpR

3,120 3,140 3,160 3,180 3,200

ATACCAAACGACGAGCGTGACACCACGATGCCTGTAGCAATGGCAACAACGTTGCGCAAACTATTAACTGGCGAACTACTTACTCTAGCTTCCCGGCAACAATTAAT
TATGGTTTGCTGCTCGCACTGTGGTGCTACGGACATCGTTACCGTTGTTGCAACGCGTTTGATAATTGACCGCTTGATGAATGAGATCGAAGGGCCGTTGTTAATTA

AmpR

3,220 3,240 3,260 3,280 3,300

AGACTGGATGGAGGCGGATAAAGTTGCAGGACCACTTCTGCGCTCGGCCCTTCCGGCTGGCTGGTTTATTGCTGATAAATCTGGAGCCGGTGAGCGTGGGTCTCGCG
TCTGACCTACCTCCGCCTATTTCAACGTCCTGGTGAAGACGCGAGCCGGGAAGGCCGACCGACCAAATAACGACTATTTAGACCTCGGCCACTCGCACCCAGAGCGC

AmpR

3,320 3,340 3,360 3,380 3,400 3,420

GTATCATTGCAGCACTGGGGCCAGATGGTAAGCCCTCCCGTATCGTAGTTATCTACACGACGGGGAGTCAGGCAACTATGGATGAACGAAATAGACAGATCGCTGAG
CATAGTAACGTCGTGACCCCGGTCTACCATTCGGGAGGGCATAGCATCAATAGATGTGCTGCCCCTCAGTCCGTTGATACCTACTTGCTTTATCTGTCTAGCGACTC

AmpR

3,440 3,460 3,480 3,500 3,520

ATAGGTGCCTCACTGATTAAGCATTGGTAACTGTCAGACCAAGTTTACTCATATATACTTTAGATTGATTTAAAACTTCATTTTTAATTTAAAAGGATCTAGGTGAA
TATCCACGGAGTGACTAATTCGTAACCATTGACAGTCTGGTTCAAATGAGTATATATGAAATCTAACTAAATTTTGAAGTAAAAATTAAATTTTCCTAGATCCACTT

AmpR

3,540 3,560 3,580 3,600 3,620

GATCCTTTTTGATAATCTCATGACCAAAATCCCTTAACGTGAGTTTTCGTTCCACTGAGCGTCAGACCCCGTAGAAAAGATCAAAGGATCTTCTTGAGATCCTTTTT
CTAGGAAAAACTATTAGAGTACTGGTTTTAGGGAATTGCACTCAAAAGCAAGGTGACTCGCAGTCTGGGGCATCTTTTCTAGTTTCCTAGAAGAACTCTAGGAAAAA
ColE1 origin
3,640 3,660 3,680 3,700 3,720 3,740

TTCTGCGCGTAATCTGCTGCTTGCAAACAAAAAAACCACCGCTACCAGCGGTGGTTTGTTTGCCGGATCAAGAGCTACCAACTCTTTTTCCGAAGGTAACTGGCTTC
AAGACGCGCATTAGACGACGAACGTTTGTTTTTTTGGTGGCGATGGTCGCCACCAAACAAACGGCCTAGTTCTCGATGGTTGAGAAAAAGGCTTCCATTGACCGAAG
ColE1 origin
3,760 3,780 3,800 3,820 3,840

AGCAGAGCGCAGATACCAAATACTGTCCTTCTAGTGTAGCCGTAGTTAGGCCACCACTTCAAGAACTCTGTAGCACCGCCTACATACCTCGCTCTGCTAATCCTGTT
TCGTCTCGCGTCTATGGTTTATGACAGGAAGATCACATCGGCATCAATCCGGTGGTGAAGTTCTTGAGACATCGTGGCGGATGTATGGAGCGAGACGATTAGGACAA
ColE1 origin
3,860 3,880 3,900 3,920 3,940

ACCAGTGGCTGCTGCCAGTGGCGATAAGTCGTGTCTTACCGGGTTGGACTCAAGACGATAGTTACCGGATAAGGCGCAGCGGTCGGGCTGAACGGGGGGTTCGTGCA
TGGTCACCGACGACGGTCACCGCTATTCAGCACAGAATGGCCCAACCTGAGTTCTGCTATCAATGGCCTATTCCGCGTCGCCAGCCCGACTTGCCCCCCAAGCACGT
ColE1 origin
3,960 3,980 4,000 4,020 4,040 4,060

CACAGCCCAGCTTGGAGCGAACGACCTACACCGAACTGAGATACCTACAGCGTGAGCTATGAGAAAGCGCCACGCTTCCCGAAGGGAGAAAGGCGGACAGGTATCCG
GTGTCGGGTCGAACCTCGCTTGCTGGATGTGGCTTGACTCTATGGATGTCGCACTCGATACTCTTTCGCGGTGCGAAGGGCTTCCCTCTTTCCGCCTGTCCATAGGC
ColE1 origin
4,080 4,100 4,120 4,140 4,160

GTAAGCGGCAGGGTCGGAACAGGAGAGCGCACGAGGGAGCTTCCAGGGGGAAACGCCTGGTATCTTTATAGTCCTGTCGGGTTTCGCCACCTCTGACTTGAGCGTCG
CATTCGCCGTCCCAGCCTTGTCCTCTCGCGTGCTCCCTCGAAGGTCCCCCTTTGCGGACCATAGAAATATCAGGACAGCCCAAAGCGGTGGAGACTGAACTCGCAGC
ColE1 origin
4,180 4,200 4,220 4,240 4,260 4,280

ATTTTTGTGATGCTCGTCAGGGGGGCGGAGCCTATGGAAAAACGCCAGCAACGCGGCCTTTTTACGGTTCCTGGCCTTTTGCTGGCCTTTTGCTCACATGTTCTTTC
TAAAAACACTACGAGCAGTCCCCCCGCCTCGGATACCTTTTTGCGGTCGTTGCGCCGGAAAAATGCCAAGGACCGGAAAACGACCGGAAAACGAGTGTACAAGAAAG
ColE1 origin
4,300 4,320 4,340 4,360 4,380

CTGCGTTATCCCCTGATTCTGTGGATAACCGTATTACCGCCTTTGAGTGAGCTGATACCGCTCGCCGCAGCCGAACGACCGAGCGCAGCGAGTCAGTGAGCGAGGAA
GACGCAATAGGGGACTAAGACACCTATTGGCATAATGGCGGAAACTCACTCGACTATGGCGAGCGGCGTCGGCTTGCTGGCTCGCGTCGCTCAGTCACTCGCTCCTT
4,400 4,420 4,440 4,460 4,480

GCGGAAGAGCGCCCAATACGCAAACCGCCTCTCCCCGCGCGTTGGCCGATTCATTAATGCAGCTGGCACGACAGGTTTCCCGACTGGAAAGCGGGCAGTGAGCGCAA
CGCCTTCTCGCGGGTTATGCGTTTGGCGGAGAGGGGCGCGCAACCGGCTAAGTAATTACGTCGACCGTGCTGTCCAAAGGGCTGACCTTTCGCCCGTCACTCGCGTT
4,500 4,520 4,540 4,560 4,580 4,600

CGCAATTAATGTGAGTTAGCTCACTCATTAGGCACCCCAGGCTTTACACTTTATGCTTCCGGCTCGTATGTTGTGTGGAATTGTGAGCGGATAACAATTTCACACAG
GCGTTAATTACACTCAATCGAGTGAGTAATCCGTGGGGTCCGAAATGTGAAATACGAAGGCCGAGCATACAACACACCTTAACACTCGCCTATTGTTAAAGTGTGTC

lac promoter LacO

4,620 4,640 4,660 4,680 4,700

GAAACAGCTATGACCATGATTACGCCAAGCTTCGGATTAGAAGCCGCCGAGCGGGTGACAGCCCTCCGAAGGAAGACTCTCCTCCGTGCGTCCTCGTCTTCACCGGT
CTTTGTCGATACTGGTACTAATGCGGTTCGAAGCCTAATCTTCGGCGGCTCGCCCACTGTCGGGAGGCTTCCTTCTGAGAGGAGGCACGCAGGAGCAGAAGTGGCCA

M13-rev GAL1 promoter

4,720 4,740 4,760 4,780 4,800

CGCGTTCCTGAAACGCAGATGTGCCTCGCGCCGCACTGCTCCGAACAATAAAGATTCTACAATACTAGCTTTTATGGTTATGAAGAGGAAAAATTGGCAGTAACCTG
GCGCAAGGACTTTGCGTCTACACGGAGCGCGGCGTGACGAGGCTTGTTATTTCTAAGATGTTATGATCGAAAATACCAATACTTCTCCTTTTTAACCGTCATTGGAC

GAL1 promoter

4,820 4,840 4,860 4,880 4,900 4,920

GCCCCACAAACCTTCAAATGAACGAATCAAATTAACAACCATAGGATGATAATGCGATTAGTTTTTTAGCCTTATTTCTGGGGTAATTAATCAGCGAAGCGATGATT
CGGGGTGTTTGGAAGTTTACTTGCTTAGTTTAATTGTTGGTATCCTACTATTACGCTAATCAAAAAATCGGAATAAAGACCCCATTAATTAGTCGCTTCGCTACTAA

GAL1 promoter

4,940 4,960 4,980 5,000 5,020

TTTGATCTATTAACAGATATATAAATGCAAAAACTGCATAACCACTTTAACTAATACTTTCAACATTTTCGGTTTGTATTACTTCTTATTCAAATGTAATAAAAGTA
AAACTAGATAATTGTCTATATATTTACGTTTTTGACGTATTGGTGAAATTGATTATGAAAGTTGTAAAAGCCAAACATAATGAAGAATAAGTTTACATTATTTTCAT

GAL1 promoter

5,040 5,060 5,080 5,100 5,120

TCAACAAAAAATTGTTAATATACCTCTATACTTTAACGTCAAGGAGAAAAAACCCCGGATCGAATTCAACCCTCACTAAAGGGCGGCCGCCATGAGATTCCCATCTA
AGTTGTTTTTTAACAATTATATGGAGATATGAAATTGCAGTTCCTCTTTTTTGGGGCCTAGCTTAAGTTGGGAGTGATTTCCCGCCGGCGGTACTCTAAGGGTAGAT

1 2 3 4 5
M R F P S

GAL1 promoter

5,140 5,160 5,180 5,200 5,220 5,240

TCTTCACCGCTGTTTTGTTCGCTGCTTCTTCTGCTTTGGCTGCTCCAGCTAACACCACCACCGAAGACGAAACCGCTCAAATCCCAGCTGAAGCTGTTATCGACTAC
AGAAGTGGCGACAAAACAAGCGACGAAGAAGACGAAACCGACGAGGTCGATTGTGGTGGTGGCTTCTGCTTTGGCGAGTTTAGGGTCGACTTCGACAATAGCTGATG

6 7 8 9 10 11 12 13 14 15 16 17 18 19 20 21 22 23 24 25 26 27 28 29 30 31 32 33 34 35 36 37 38 39 40 41
I F T A V L F A A S S A L A A P A N T T T E D E T A Q I P A E A V I D Y

Mating factor alpha leader

5,260 5,280 5,300 5,320 5,340

TCTGACTTGGAAGGTGACTTCGACGCTGCTGCTTTGCCATTGTCTAACTCTACCAACAACGGTTTGTCTTCTACCAACACCACCATCGCTTCTATCGCTGCTAAGGA
AGACTGAACCTTCCACTGAAGCTGCGACGACGAAACGGTAACAGATTGAGATGGTTGTTGCCAAACAGAAGATGGTTGTGGTGGTAGCGAAGATAGCGACGATTCCT
42 43 44 45 46 47 48 49 50 51 52 53 54 55 56 57 58 59 60 61 62 63 64 65 66 67 68 69 70 71 72 73 74 75 76 77
S D L E G D F D A A A L P L S N S T N N G L S S T N T T I A S I A A K E
Mating factor alpha leader
5,360 5,380 5,400 5,420 5,440

AGAAGGTGTTCAATTGGACAAGAGAGAAGCTAGCGCAGGATCCTACCCATACGATGTTCCAGATTACGCTCAAACATCCTCACCCTCTACAGAAGCATCAGTATCAA
TCTTCCACAAGTTAACCTGTTCTCTCTTCGATCGCGTCCTAGGATGGGTATGCTACAAGGTCTAATGCGAGTTTGTAGGAGTGGGAGATGTCTTCGTAGTCATAGTT
78 79 80 81 82 83 84 85 86 1 2 3 4 5 1 2 3 4 5 6 7 8 9 2 4 6 8 10 12
E G V Q L D K R E A S A G S Y P Y D V P D Y A Q T S S P S T E A S V S
HA tag 649 stalk
5,460 5,480 5,500 5,520 5,540 5,560

CCTCTAGTACCTCTTCCTCTGCTTCACAATCATCTGACCCAACTACAACATCTTCGTCCAGTTCATCGTCTTCCCCATCTTCCCAATCTGAGGAAATTTCATCATCA
GGAGATCATGGAGAAGGAGACGAAGTGTTAGTAGACTGGGTTGATGTTGTAGAAGCAGGTCAAGTAGCAGAAGGGGTAGAAGGGTTAGACTCCTTTAAAGTAGTAGT
14 16 18 20 22 24 26 28 30 32 34 36 38 40 42 44 46 48
T S S T S S S A S Q S S D P T T T S S S S S S S S P S S Q S E E I S S S
649 stalk
5,580 5,600 5,620 5,640 5,660

CCGACGGTCTCAACTACACCATCAACTTCTTCATCATCTTCCTCAATGACTTCAACCACCACAACAAAGTCAATCTCAACTTCCACTACAAGTTCAGCTCCAGTTAC
GGCTGCCAGAGTTGATGTGGTAGTTGAAGAAGTAGTAGAAGGAGTTACTGAAGTTGGTGGTGTTGTTTCAGTTAGAGTTGAAGGTGATGTTCAAGTCGAGGTCAATG
50 52 54 56 58 60 62 64 66 68 70 72 74 76 78 80 82 84
P T V S T T P S T S S S S S S M T S T T T T K S I S T S T T S S A P V T
649 stalk
5,680 5,700 5,720 5,740 5,760

AGATGTGACAGTTTCCTCATCGCCTAGTAAATCTACCTCTACTTCGACAAGTACAGAAACATCTAAAACACCTACTTCAATGACAGAGTATACATCTAGTACATCGA
TCTACACTGTCAAAGGAGTAGCGGATCATTTAGATGGAGATGAAGCTGTTCATGTCTTTGTAGATTTTGTGGATGAAGTTACTGTCTCATATGTAGATCATGTAGCT
86 88 90 92 94 96 98 100 102 104 106 108 110 112 114 116 118
D V T V S S S P S K S T S T S T S T E T S K T P T S M T E Y T S S T S
649 stalk
5,780 5,800 5,820 5,840 5,860 5,880

TAATTTCGACTCCAGTTAGTCACTCGCAGACAGGTTTGTCGGCTTCATCAAGTTCATCATCTACAACATCCGGTTCTTCGTCCACTAAATCAGAAAGTTCGACAACA
ATTAAAGCTGAGGTCAATCAGTGAGCGTCTGTCCAAACAGCCGAAGTAGTTCAAGTAGTAGATGTTGTAGGCCAAGAAGCAGGTGATTTAGTCTTTCAAGCTGTTGT
120 122 124 126 128 130 132 134 136 138 140 142 144 146 148 150 152 154
I I S T P V S H S Q T G L S A S S S S S S T T S G S S S T K S E S S T T
649 stalk
5,900 5,920 5,940 5,960 5,980

TCTGGCTCTTCCCAGTCCGTGGAATCAACCTCCAGCCACGCCACTGTTCTTGCTAATTCCGCAGAAATGGTCACAACATCCTCTAGTTCATCCTCAACATCCGAAAT
AGACCGAGAAGGGTCAGGCACCTTAGTTGGAGGTCGGTGCGGTGACAAGAACGATTAAGGCGTCTTTACCAGTGTTGTAGGAGATCAAGTAGGAGTTGTAGGCTTTA
156 158 160 162 164 166 168 170 172 174 176 178 180 182 184 186 188 190
S G S S Q S V E S T S S H A T V L A N S A E M V T T S S S S S S T S E M
649 stalk
6,000 6,020 6,040 6,060 6,080

GTCATTAACTAGTACTGCTACCAGTGTACCAGTCTCATCTAGTAGCAGTACGACATATTCTACTAGCGCATCTACACAAGCCGTCACTACAACATCTTCTTCCACTG
CAGTAATTGATCATGACGATGGTCACATGGTCAGAGTAGATCATCGTCATGCTGTATAAGATGATCGCGTAGATGTGTTCGGCAGTGATGTTGTAGAAGAAGGTGAC
192 194 196 198 200 202 204 206 208 210 212 214 216 218 220 222 224 226
S L T S T A T S V P V S S S S S T T Y S T S A S T Q A V T T T S S S T
649 stalk
6,100 6,120 6,140 6,160 6,180 6,200

TATCTACAACTTCTTCTAGTACAACGTTAACAAGCGCATTCACACATTCTTCAACCACATCGTCCGACCAGCCACCCAGCGACACTACAAGTCCATCTACGACACAC
ATAGATGTTGAAGAAGATCATGTTGCAATTGTTCGCGTAAGTGTGTAAGAAGTTGGTGTAGCAGGCTGGTCGGTGGGTCGCTGTGATGTTCAGGTAGATGCTGTGTG
228 230 232 234 236 238 240 242 244 246 248 250 252 254 256 258 260 262
V S T T S S S T T L T S A F T H S S T T S S D Q P P S D T T S P S T T H
649 stalk
6,220 6,240 6,260 6,280 6,300

GAACCTCATGTAACCACTCAGACGTCATCAGAAACATCTTCTTCTAAGTCATCTTCTACTTCTTCTTCAAGTACATCTCAAACCTCTGAGTCTGCAACACCATCCGA
CTTGGAGTACATTGGTGAGTCTGCAGTAGTCTTTGTAGAAGAAGATTCAGTAGAAGATGAAGAAGAAGTTCATGTAGAGTTTGGAGACTCAGACGTTGTGGTAGGCT
264 266 268 270 272 274 276 278 280 282 284 286 288 290 292 294 296 298
E P H V T T Q T S S E T S S S K S S S T S S S S T S Q T S E S A T P S D
649 stalk
6,320 6,340 6,360 6,380 6,400 6,420

TTCCGTATCACCTGGAAGTTCTACATCAACATCTTCTAGTAGCACTTCTACTTCCACTTCTATTTCCAGTGGAGAAACGACAACTTCTTCTTCTTCATCATCTGCCA
AAGGCATAGTGGACCTTCAAGATGTAGTTGTAGAAGATCATCGTGAAGATGAAGGTGAAGATAAAGGTCACCTCTTTGCTGTTGAAGAAGAAGAAGTAGTAGACGGT
300 302 304 306 308 310 312 314 316 318 320 322 324 326 328 330 332
S V S P G S S T S T S S S S T S T S T S I S S G E T T T S S S S S S A
649 stalk
6,440 6,460 6,480 6,500 6,520

CGACCACTTCTAACAGCGCAACCTTGTCAGTCTCTACCACACAAACTTCGATTGAAGCCAGTTCATCTACTACATCTACATCTAGTTCAACAATTACAACTTCAAGT
GCTGGTGAAGATTGTCGCGTTGGAACAGTCAGAGATGGTGTGTTTGAAGCTAACTTCGGTCAAGTAGATGATGTAGATGTAGATCAAGTTGTTAATGTTGAAGTTCA
334 336 338 340 342 344 346 348 350 352 354 356 358 360 362 364 366 368
T T T S N S A T L S V S T T Q T S I E A S S S T T S T S S S T I T T S S
649 stalk
6,540 6,560 6,580 6,600 6,620

```
AGTAGCGCTCACATATCGTCGAAATCTCAATCTAGTATTACCTATCCCTCTTCCTCGACATCTTCATCTACATCGTCCTCAATTTCTAGCGAATCTGAAAGTTTTGA
TCATCGCGAGTGTATAGCAGCTTTAGAGTTAGATCATAATGGATAGGGAGAAGGAGCTGTAGAAGTAGATGTAGCAGGAGTTAAAGATCGCTTAGACTTTCAAAACT
370    372    374    376    378    380    382    384    386    388    390    392    394    396    398    400    402    404
 S  S  A  H  I  S  S  K  S  Q  S  S  I  T  Y  P  S  S  S  T  S  S  S  T  S  S  S  I  S  S  E  S  E  S  F  E
649 stalk
     6,640               6,660               6,680               6,700               6,720               6,740

ATCGACATCAGCAGAAGATGCTCCATCAACAGCACCTTCATCAAGTGTCTCTTCTAAGAGTTCTACCTCTACAACATCAAGCACATCGACATCTTCAAGCACTCCAT
TAGCTGTAGTCGTCTTCTACGAGGTAGTTGTCGTGGAAGTAGTTCACAGAGAAGATTCTCAAGATGGAGATGTTGTAGTTCGTGTAGCTGTAGAAGTTCGTGAGGTA
 406    408    410    412    414    416    418    420    422    424    426    428    430    432    434    436    438    440
 S  T  S  A  E  D  A  P  S  T  A  P  S  S  S  V  S  S  K  S  S  T  S  T  T  S  S  T  S  T  S  S  S  T  P
649 stalk
                 6,760               6,780               6,800               6,820               6,840

CTCCATCACCATCTTCCGTGAGTTCTTCCTCCACCAGCTCATTGACAACTTCTGCTGTATCAACACCAGCTACCTCTCATTCTCAAAGTACTGTAGTAACCACCACT
GAGGTAGTGGTAGAAGGCACTCAAGAAGGAGGTGGTCGAGTAACTGTTGAAGACGACATAGTTGTGGTCGATGGAGAGTAAGAGTTTCATGACATCATTGGTGGTGA
  442    444    446    448    450    452    454    456    458    460    462    464    466    468    470    472    474    476
 S  P  S  P  S  S  V  S  S  S  S  T  S  S  L  T  T  S  A  V  S  T  P  A  T  S  H  S  Q  S  T  V  V  T  T  T
649 stalk
            6,860               6,880               6,900               6,920               6,940

ACTATTACTACATCAACAGGTCCAGTGATGTCTACGACAACAGCTTATTCTTCTAGTTCTACTAGCAGCTCGGAATCTTCTGAGGTTCAGTCTGTCATGTCATCTAC
TGATAATGATGTAGTTGTCCAGGTCACTACAGATGCTGTTGTCGAATAAGAAGATCAAGATGATCGTCGAGCCTTAGAAGACTCCAAGTCAGACAGTACAGTAGATG
   478    480    482    484    486    488    490    492    494    496    498    500    502    504    506    508    510   512
 T  I  T  T  S  T  G  P  V  M  S  T  T  T  A  Y  S  S  S  S  T  S  S  S  E  S  S  E  V  Q  S  V  M  S  S  T
649 stalk
      6,960               6,980               7,000               7,020               7,040               7,060

GCCTAGTTCAACATCAACAACAACCAGTTCGGAATCTACTTCATCTAGCTCCACAGCTTCTACCTCACCATCAACCTCGCAAACTTTCGAAACTTCTCCTACTATAG
CGGATCAAGTTGTAGTTGTTGTTGGTCAAGCCTTAGATGAAGTAGATCGAGGTGTCGAAGATGGAGTGGTAGTTGGAGCGTTTGAAAGCTTTGAAGAGGATGATATC
    514    516    518    520    522    524    526    528    530    532    534    536    538    540    542    544    546
 P  S  S  T  S  T  T  T  S  S  E  S  T  S  S  S  S  T  A  S  T  S  P  S  T  S  Q  T  F  E  T  S  P  T  I
649 stalk
                 7,080               7,100               7,120               7,140               7,160

GAGGTGTCCCCTCAACCACTTCATTTGTCTCTACGCCAACAACGAAATTGTCGCACACTACTTCCACTATGACAGCACAGTCCGATAGTAAGTCTACCCACTCCTCA
CTCCACAGGGGAGTTGGTGAAGTAAACAGAGATGCGGTTGTTGCTTTAACAGCGTGTGATGAAGGTGATACTGTCGTGTCAGGCTATCATTCAGATGGGTGAGGAGT
548    550    552    554    556    558    560    562    564    566    568    570    572    574    576    578    580    582
 G  G  V  P  S  T  T  S  F  V  S  T  P  T  T  K  L  S  H  T  T  S  T  M  T  A  Q  S  D  S  K  S  T  H  S  S
649 stalk
          7,180               7,200               7,220               7,240               7,260
```

```
AGCACATCGACAGAAGATAAATCATCCACTGCTTCTGCAGTTGACGAAAGCACTACAACATCCACTTCCACGGAGTCTACTACATCAGTAACATCAGGCACCTCCCA
TCGTGTAGCTGTCTTCTATTTAGTAGGTGACGAAGACGTCAACTGCTTTCGTGATGTTGTAGGTGAAGGTGCCTCAGATGATGTAGTCATTGTAGTCCGTGGAGGGT
584   586   588   590   592   594   596   598   600   602   604   606   608   610   612   614   616   618
S T S T E D K S S T A S A V D E S T T T S T S T E S T T S V T S G T S H
649 stalk
7,280   7,300   7,320   7,340   7,360   7,380

TTCCGCTAAAGAATCTTCGTCAAATTCTAAGGTGTATAGTTCACAGACAGCACACTCATCCATAAGTGTTGCATCATCACCTAGTACAAAGGGCGCCCAAATCCAAT
AAGGCGATTTCTTAGAAGCAGTTTAAGATTCCACATATCAAGTGTCTGTCGTGTGAGTAGGTATTCACAACGTAGTAGTGGATCATGTTTCCCGCGGGTTTAGGTTA
620   622   624   626   628   630   632   634   636   638   640   642   644   646   648   650   652   654
S A K E S S S N S K V Y S S Q T A H S S I S V A S S P S T K G A Q I Q
649 stalk
7,400   7,420   7,440   7,460   7,480

CTTCTATGGTTGAAATCTCTACCTACGCTGGTTCTGCTAACTCTGTTAACGCTGGTGCTGGTGCTGGTGCTTTGTTCTTGTTGTTGTCTTTGGCTATCATCTAATGA
GAAGATACCAACTTTAGAGATGGATGCGACCAAGACGATTGAGACAATTGCGACCACGACCACGACCACGAAACAAGAACAACAACAGAAACCGATAGTAGATTACT
656   658   660   662   664   666   668   670   672   674   676   678   680   682   684   686   688 1   2
S S M V E I S T Y A G S A N S V N A G A G A G A L F L L L S L A I I * *
GPI anchor
7,500   7,520   7,540   7,560   7,580

TTAATTAACTCGAGATCTGATAACAACAGTGTAGATGTAACAAAATCGACTTTGTTCCCACTGTACTTTTAGCTCGTACAAAATACAATATACTTTTCATTTCTCCG
AATTAATTGAGCTCTAGACTATTGTTGTCACATCTACATTGTTTTAGCTGAAACAAGGGTGACATGAAAATCGAGCATGTTTTATGTTATATGAAAAGTAAAGAGGC
3 4 5 6 7
L I N S R
alpha factor terminator
7,600   7,620   7,640   7,660   7,680   7,700

TAAACAACATGTTTTCCCATGTAATATCCTTTTCTATTTTTCGTTCCGTTACCAACTTTACACATACTTTATATAGCTATTCACTTCTATACACTAAAAAACTAAGA
ATTTGTTGTACAAAAGGGTACATTATAGGAAAAGATAAAAAGCAAGGCAATGGTTGAAATGTGTATGAAATATATCGATAAGTGAAGATATGTGATTTTTTGATTCT
alpha factor terminator
7,720   7,740   7,760   7,780   7,800

CAATTTTAATTTTGCTGCCTGCCATATTTCAATTTGTTATAAATTCCTATAATTTATCCTATTAGTAGCTAAAAAAAGATGAATGTGAATCGAATCCTAAGAGAATT
GTTAAAATTAAAACGACGGACGGTATAAAGTTAAACAATATTTAAGGATATTAAATAGGATAATCATCGATTTTTTTCTACTTACACTTAGCTTAGGATTCTCTTAA
alpha factor terminator
7,820   7,840   7,860   7,880   7,900

GGTACCGCGATGTAGTAAAACTAGCTAGACCGAGAAAGAGACTAGAAATGCAAAAGGCACTTCTACAATGGCTGCCATCATTATTATCCGATGTGACGCTGCATTTT
CCATGGCGCTACATCATTTTGATCGATCTGGCTCTTTCTCTGATCTTTACGTTTTCCGTGAAGATGTTACCGACGGTAGTAATAATAGGCTACACTGCGACGTAAAA
7,920   7,940   7,960   7,980   8,000   8,020
```

```
TTTTTTTTTTTTTTTTTTTTTTTTTTTTTTTTTTTTTTTTTTGTACAAATATCATAAAAAAAGAGAATCTTTTTAAGCAAGGATTTTCTTAACTTCTTCGGCGACA
AAAAAAAAAAAAAAAAAAAAAAAAAAAAAAAAAAAAAAAAAACATGTTTATAGTATTTTTTTCTCTTAGAAAAATTCGTTCCTAAAAGAATTGAAGAAGCCGCTGT
        8,040       8,060       8,080       8,100       8,120

GCATCACCGACTTCGGTGGTACTGTTGGAACCACCTAAATCACCAGTTCTGATACCTGCATCCAAAACCTTTTTAACTGCATCTTCAATGGCCTTACCTTCTTCAGG
CGTAGTGGCTGAAGCCACCATGACAACCTTGGTGGATTTAGTGGTCAAGACTATGGACGTAGGTTTTGGAAAAATTGACGTAGAAGTTACCGGAATGGAAGAAGTCC
    8,140       8,160       8,180       8,200       8,220

CAAGTTCAATGACAATTTCAACATCATTGCAGCAGACAAGATAGTGGCGATAGGGTCAACCTTATTCTTTGGCAAATCTGGAGCAGAACCGTGGCATGGTTCGTACA
GTTCAAGTTACTGTTAAAGTTGTAGTAACGTCGTCTGTTCTATCACCGCTATCCCAGTTGGAATAAGAAACCGTTTAGACCTCGTCTTGGCACCGTACCAAGCATGT
8,240       8,260       8,280       8,300       8,320       8,340

AACCAAATGCGGTGTTCTTGTCTGGCAAAGAGGCCAAGGACGCAGATGGCAACAAACCCAAGGAACCTGGGATAACGGAGGCTTCATCGGAGATGATATCACCAAAC
TTGGTTTACGCCACAAGAACAGACCGTTTCTCCGGTTCCTGCGTCTACCGTTGTTTGGGTTCCTTGGACCCTATTGCCTCCGAAGTAGCCTCTACTATAGTGGTTTG
       8,360       8,380       8,400       8,420       8,440

ATGTTGCTGGTGATTATAATACCATTTAGGTGGGTTGGGTTCTTAACTAGGATCATGGCGGCAGAATCAATCAATTGATGTTGAACCTTCAATGTAGGAAATTCGTT
TACAACGACCACTAATATTATGGTAAATCCACCCAACCCAAGAATTGATCCTAGTACCGCCGTCTTAGTTAGTTAACTACAACTTGGAAGTTACATCCTTTAAGCAA
   8,460       8,480       8,500       8,520       8,540       8,560

CTTGATGGTTTCCTCCACAGTTTTTCTCCATAATCTTGAAGAGGCCAAAACATTAGCTTTATCCAAGGACCAAATAGGCAATGGTGGCTCATGTTGTAGGGCCATGA
GAACTACCAAAGGAGGTGTCAAAAAGAGGTATTAGAACTTCTCCGGTTTTGTAATCGAAATAGGTTCCTGGTTTATCCGTTACCACCGAGTACAACATCCCGGTACT
          8,580       8,600       8,620       8,640       8,660

AAGCGGCCATTCTTGTGATTCTTTGCACTTCTGGAACGGTGTATTGTTCACTATCCCAAGCGACACCATCACCATCGTCTTCCTTTCTCTTACCAAAGTAAATACCT
TTCGCCGGTAAGAACACTAAGAAACGTGAAGACCTTGCCACATAACAAGTGATAGGGTTCGCTGTGGTAGTGGTAGCAGAAGGAAAGAGAATGGTTTCATTTATGGA
      8,680       8,700       8,720       8,740       8,760

CCCACTAATTCTCTGACAACAACGAAGTCAGTACCTTTAGCAAATTGTGGCTTGATTGGAGATAAGTCTAAAAGAGAGTCGGATGCAAAGTTACATGGTCTTAAGTT
GGGTGATTAAGAGACTGTTGTTGCTTCAGTCATGGAAATCGTTTAACACCGAACTAACCTCTATTCAGATTTTCTCTCAGCCTACGTTTCAATGTACCAGAATTCAA
  8,780       8,800       8,820       8,840       8,860       8,880

GGCGTACAATTGAAGTTCTTTACGGATTTTTAGTAAACCTTGTTCAGGTCTAACACTACCTGTACCCCATTTAGGACCACCCACAGCACCTAACAAAACGGCATCAA
CCGCATGTTAACTTCAAGAAATGCCTAAAAATCATTTGGAACAAGTCCAGATTGTGATGGACATGGGGTAAATCCTGGTGGGTGTCGTGGATTGTTTTGCCGTAGTT
          8,900       8,920       8,940       8,960       8,980

CCTTCTTGGAGGCTTCCAGCGCCTCATCTGGAAGTGGGACACCTGTAGCATCGATAGCAGCACCACCAATTAAATGATTTTCGAAATCGAACTTGACATTGGAACGA
GGAAGAACCTCCGAAGGTCGCGGAGTAGACCTTCACCCTGTGGACATCGTAGCTATCGTCGTGGTGGTTAATTTACTAAAAGCTTTAGCTTGAACTGTAACCTTGCT
      9,000       9,020       9,040       9,060       9,080

ACATCAGAAATAGCTTTAAGAACCTTAATGGCTTCGGCTGTGATTTCTTGACCAACGTGGTCACCTGGCAAAACGACGATCTTCTTAGGGGCAGACATTACAATGGT
TGTAGTCTTTATCGAAATTCTTGGAATTACCGAAGCCGACACTAAAGAACTGGTTGCACCAGTGGACCGTTTTGCTGCTAGAAGAATCCCCGTCTGTAATGTTACCA
  9,100       9,120       9,140       9,160       9,180       9,200
```

ATATCCTTGAAATATATATAAAAAAAAAAAAAAAAAAAAAAAAAAAAAAAATGCAGCTTCTCAATGATATTCGAATACGCTTTGAGGAGATACAGCCTAATATCCGAC
TATAGGAACTTTATATATATTTTTTTTTTTTTTTTTTTTTTTTTTTTTTTTACGTCGAAGAGTTACTATAAGCTTATGCGAAACTCCTCTATGTCGGATTATAGGCTG

9,220 9,240 9,260 9,280 9,300

AAACTGTTTTACAGATTTACGATCGTACTTGTTACCCATCATTGAATTTTGAACATCCGAACCTGGGAGTTTTCCCTGAAACAGATAGTATATTTGAACCTGTATAA
TTTGACAAAATGTCTAAATGCTAGCATGAACAATGGGTAGTAACTTAAAACTTGTAGGCTTGGACCCTCAAAAGGGACTTTGTCTATCATATAAACTTGGACATATT

9,320 9,340 9,360 9,380 9,400

TAATATATAGTCTAGCGCTTTACGGAAGACAATGTATGTATTTCGGTTCCTGGAGAAACTATTGCATCTATTGCATAGGTAATCTTGCACGTCGCATCCCCGGTTCA
ATTATATATCAGATCGCGAAATGCCTTCTGTTACATACATAAAGCCAAGGACCTCTTTGATAACGTAGATAACGTATCCATTAGAACGTGCAGCGTAGGGGCCAAGT

9,420 9,440 9,460 9,480 9,500 9,520

TTTTCTGCGTTTCCATCTTGCACTTCAATAGCATATCTTT
AAAAGACGCAAAGGTAGAACGTGAAGTTATCGTATAGAAA

9,540 9,560
